# Functional expression of diverse post-translational peptide-modifying enzymes in *Escherichia coli* under uniform expression and purification conditions

**DOI:** 10.1371/journal.pone.0266488

**Published:** 2022-09-19

**Authors:** Emerson Glassey, Andrew M. King, Daniel A. Anderson, Zhengan Zhang, Christopher A. Voigt

**Affiliations:** Department of Biological Engineering, Synthetic Biology Center, Massachusetts Institute of Technology, Cambridge, MA, United States of America; Shenzhen Bay Laboratory, CHINA

## Abstract

RiPPs (ribosomally-synthesized and post-translationally modified peptides) are a class of pharmaceutically-relevant natural products expressed as precursor peptides before being enzymatically processed into their final functional forms. Bioinformatic methods have illuminated hundreds of thousands of RiPP enzymes in sequence databases and the number of characterized chemical modifications is growing rapidly; however, it remains difficult to functionally express them in a heterologous host. One challenge is peptide stability, which we addressed by designing a RiPP stabilization tag (RST) based on a small ubiquitin-like modifier (SUMO) domain that can be fused to the N- or C-terminus of the precursor peptide and proteolytically removed after modification. This is demonstrated to stabilize expression of eight RiPPs representative of diverse phyla. Further, using *Escherichia coli* for heterologous expression, we identify a common set of media and growth conditions where 24 modifying enzymes, representative of diverse chemistries, are functional. The high success rate and broad applicability of this system facilitates: (i) RiPP discovery through high-throughput “mining” and (ii) artificial combination of enzymes from different pathways to create a desired peptide.

## Introduction

Metagenomics has led to a deluge of microbial genomes, leading to high-throughput efforts to “mine” the molecules made by organisms by rebuilding pathways and screening for functions-of-interest [1–3]. Because they are gleaned from sequence databases, the organism or genomic DNA may not be available, thus necessitating the use of DNA synthesis and a heterologous host to obtain the chemical product [4–6]. RiPPs (ribosomally-synthesized and post-translationally modified peptides) are a potentially rich source of functional diversity that are encoded in gene clusters as a precursor peptide that is enzymatically modified before being proteolytically released [7–14]. Because the peptidic product is made by the ribosome, as opposed to a large megasynthase, the probability of successful heterologous expression is high. However, expressed peptides are often unstable *in vivo* and post-translational modifying enzymes may not function in new contexts [15–17]. As a result, only a small fraction of the thousands of known RiPP pathways have been explored [13].

RiPPs are classified by the chemical modifications made to the peptide. Some are defined by cyclization chemistry, including lanthipeptides (lanthionine macrocyclizations), thiopeptides ([4+2] cycloaddition of dehydrated serine/threonine), lasso peptides (N-terminal macrocyclization with asp/glu), graspetides (lactone/lactam macrocyclizations), bottromycin (macrolactamidine macrocyclization), ranthipeptides (Non-Cα thioether macrocyclizations), pantocins (glutamate crosslink), and sactipeptides (sactionine macrocyclizations) [7, 14]. Others by individual modifications present, such as glycocins (side chain glycosylation), microcin C (aminoacyl adenylation or cytidylation), comX (indole cyclization and prenylation), sulfatyrotide (tyrosine sulfation), spliceotide (β-amino acids from backbone splicing), and cyanobactins (N-terminal proteolysis). Precursor peptide organization varies between RiPP classes. Modifying enzymes can either bind to a leader/follower sequence in the precursor peptide or directly modify the core. The core consists of 2 to over 50 amino acids and there can be multiple cores in one precursor peptide [17–20]. Leader peptides range from 7 to over 80 amino acids and can recruit multiple modifying enzymes that can have overlapping binding sequences [21–23]. The diversity in chemistry and genetic encoding complicates the creation of general engineering tools that can be systematically used for mining efforts across RiPP classes.

There are examples from most RiPP classes have been successfully expressed in *E*. *coli* [16, 17, 20–22, 24–29]. Tools have been developed to aid heterologous production, including multi-plasmid inducible systems and exploration of *E*. *coli*, various *Streptomyces* strains and *Microvirgula aerodentrificans* as expression hosts [17, 30–33]. *In vitro* methods have also been used to engineer production of new molecules or study biosynthesis [34–37]. Gene cluster regulation may not function properly in a new host. To overcome this, the precursor peptide and modifying enzymes can be cloned and expressed separately [17, 33]. However, precursor peptides have been observed to often be unstable due to host proteases, thus necessitating the use of stabilization tags [15, 16, 24]. Large tags need to be removed before peptide modifications can be observed by mass spectrometry, as is the case for maltose binding protein (MBP, 45 kD), green fluorescent protein (GFP, 27 kD) or glutathione-S-transferase (GST, 26 kD) [38]. In contrast, the small ubiquitin-like modifier tag (SUMO, 12 kD) is smaller, thus allowing modifications to be observed prior to its removal. Further, it can be removed using SUMO protease immediately after purification without desalting [39], which simplifies its use in high-throughput formats. SUMO has been used for expression of both eukaryotic and prokaryotic antimicrobial peptides in *E*. *coli* [40–42] as well as a post-translationally modified lanthipeptide from *Lactococcus* [43] and a xenorceptide from *Xenorhabdus* [44].

Here, we develop a method for the expression, modification and purification of diverse RiPPs under uniform conditions in order to inform high-throughput mining efforts. We use a SUMO tag and add affinity tags, cleavage sites and linkers, which we refer to as “RiPP Stabilization Tag” (RST). This SUMO-based tag is demonstrated to work with diverse RiPP classes and modifying enzymes. Inducible systems from *E*. *coli* Marionette [45] are used to express the tagged precursor peptide and modifying enzymes from separate plasmids. These enzymes are all expressed in the same heterologous host (*E*. *coli*) under uniform culture conditions and induction times. We tested 50 precursor peptides with 46 modifying enzymes and identify 39 peptides that express as RST fusions, of which 24 were able to be modified with the SUMO-based tag attached. This provides a method that can be used for high-throughput RiPP discovery out of metagenomics data and for selecting enzymes that can be combined to build pathways to new-to-nature RiPPs.

## Results

### Expression system for modified peptides

**Fig 1:**
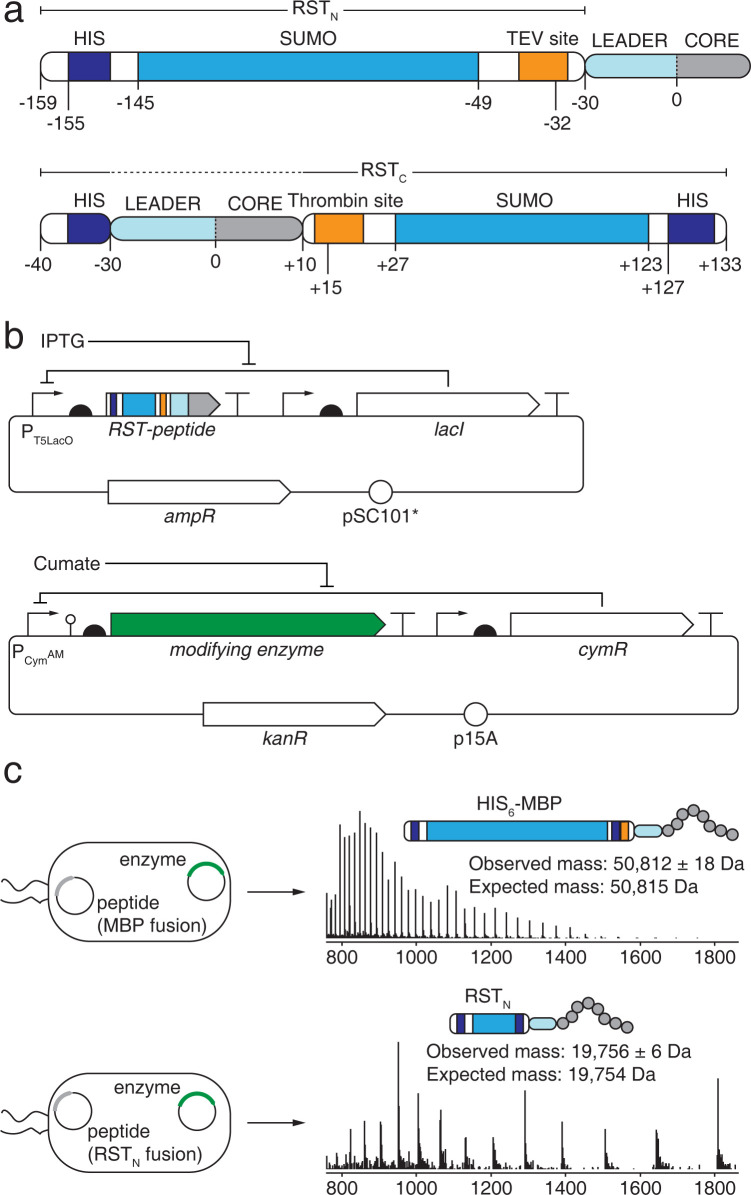
Expression system for producing modified peptides. **a** Schematic of the RSTs. Sequences for the backbones are in S8 Table, part sequences in S7 Table. **b** The two-plasmid system used to express the RiPP peptide and modifying enzyme. The genetic part sequences are provided in S7 Table. **c** The extracted peptides were analyzed using LC-MS to observe the mass shift associated with the modification. The tags used are shown above the spectra. The observed masses were calculated from 5 consecutive *m/z* peaks in the spectrum (Methods and S1 Table) and the expected masses are the molar mass of the peptide, calculated from the primary sequences. The initially designed RST_N_ variant with Link-1 was used in panel c. The spectra in c were collected from single experiments.

Two versions of the RST were designed so that it can be fused to either the N- or C-terminus (RST_N_ or RST_C_) of a precursor peptide (Fig 1A). Typically, the N-terminal version was used, but having a C-terminal version is convenient when either there is a modification at the N-terminus or the leader peptide is removed during modification. For purification, a HIS_6_ tag was included at the terminus of the RST. Small Ubiquitin-like Modifier (SUMO) was used as the stabilizing domain, with a linker sequence between SUMO and the precursor peptide. The linker was adapted from a recombinant protein expression system [46] and includes protease cleavage sites: TEV (N-terminal) or Thrombin (C-terminal). After purification, the RST can be removed using either these proteases or SUMO protease (for RST_N_). Treatment with TEV leaves a GC scar at the N-terminus, where the cysteine is included for SAMDI (self-assembled monolayers on gold for matrix-assisted laser desorption/ionization) mass spectroscopy [47, 48].

A two-plasmid system was used to separately express the precursor peptide and modifying enzyme, thus enabling combinations to be tested rapidly through co-transformations (Figs 1B and S1). The inducible system for the precursor peptide was selected to maximize its expression. To this end, we used the IPTG-inducible P_T5LacO_ promoter [45] and a strong ribosome binding site (RBS) designed using the RBS Calculator [49, 50] (Methods). For the modifying enzyme, we used the cumate-inducible P_CymR*_ or ahl-inducible P_LuxB_ because of their high dynamic range (low off and high on) [45]. A different RBS is calculated for each modifying enzyme to maximize the probability of successful expression and bias toward similar expression levels.

Expression and purification protocols were first developed for low-throughput growth in 250 ml flasks in LB media (Methods). The tagged precursor peptide and modifying enzyme were induced simultaneously. After induction with 1 mM IPTG and 200 μM cumate (for P_CymR*_) or 10 μM 3OC6-AHL (for P_LuxB_), cultures were grown at 18°C for 20 hours with shaking. Then, the peptide was purified using immobilized metal affinity chromatography (IMAC) and analyzed using LC-MS.

An example of the production of a modified peptide in flasks is shown in Fig 1C using a variant of the trunkamide precursor peptide (TruE*) and cognate modifying enzyme TruD. Two samples were prepared: 1. the TruE* peptide fused to an initial version of RST_N_ that used Link-1 (instead of Link-2) co-transformed with the plasmid containing P_LuxB_-controlled *truD* (pEG1128), 2. the TruE* peptide expressed as an MBP fusion also co-transformed with pEG1128. From the LC-MS spectra, we calculated the observed mass for each of the peptides, and the expected error given the resolution of the mass spectrometer (Methods). TruD catalyzes the formation of a thiazoline from cysteine, causing a loss of water and a corresponding mass shift of -18 Da. The larger MBP obfuscates the observation of this expected mass shift because the resolution of the mass spectrometer is too low to reliably resolve modified versus unmodified MBP-tagged peptide and the isotope distributions between modified and unmodified MBP-tagged peptides overlap. In contrast, the expected mass spectrometer resolution of the RST_N_ fusion is 6 Da (standard deviation), and the expected and observed masses match (Fig 1C). Therefore, we conclude that we can observe the mass shift that occurs due to post-translational modification without removing the RST_N_, even using a low-resolution quadrupole mass spectrometer.

**Fig 2:**
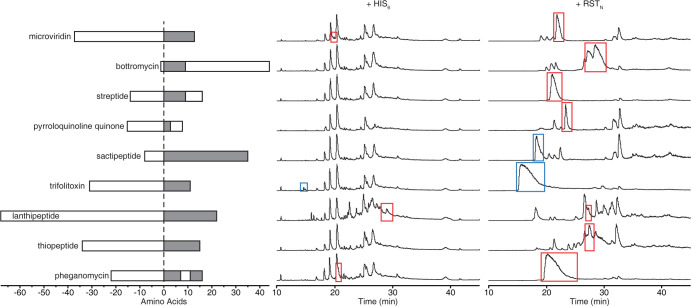
RST_N_ stabilization of unmodified peptides from diverse RiPP classes. The peptide schematics show precursor peptide structural elements (leader/follower, white box) and core (grey box). From top to bottom, the gene names (species) are: *mdnA*, *bmbC*, *strA*, *pqqA*, *sboA*, *tfxA*, *procA1*.*7*, *tbtA*, *pgm2*. The LC-MS total ion chromatograms are shown (Methods). Peaks boxed in red match peptide expected mass, blue match the expected mass of a peptide cleavage product. Masses are provided in S1 Table. In +HIS_6_ column, top-to-bottom, extracts are 192, 193, 194, 195, 196, 198, 199, 200, and 202. In +RST_N_ column, top-to-bottom, extracts are 216, 220, 219, 218, 217, 221, 222, 223, and 225. For these experiments, the initially designed RST_N_ variant with Link-1 was used. The chromatograms are representative of two replicates that yielded similar results (the second performed with the high-throughput expression/purification protocol, Methods).

Next, we tested RST_N_ stabilization of diverse precursor peptides across RiPP classes (Fig 2). The following examples from each class were selected: microviridin L from graspetides, bottromycin from bottromycins, streptide from streptides, PQQ from pyrroloquinoline quinones, subtilosin A from sactipeptides, trifolitoxin from linear azole peptides, prochlorosin from lanthipeptides, thiomuracin from thiopeptides, and pheganomycin from guanidinotides [14, 20, 29, 51–57]. This set encompasses a wide range of lengths, amino acid compositions, number of modifying enzyme binding sites, N- and C-terminal leaders/followers, and pheganomycin has two cores.

We tested the ability for RST_N_ to stabilize the unmodified peptides. Expression was measured in the absence of modifying enzymes to abrogate any stabilization affect that arises from peptide modification. Expression and purification were performed at the 250 mL flask scale, as described above (Methods). First, we evaluated precursor peptide expression when fused only to a N-terminal HIS_6_ tag [16]. This tag led to only three of nine peptides being detected by LC-MS (Fig 2). In contrast, when the precursor peptides were fused to an initial version of RST_N_ (using Link-1), large peaks appeared for all of the peptides. In summary, two of the nine peptides expressed as HIS_6_ fusions and seven of the nine peptides expressed as RST fusions were successfully expressed and purified as full-length peptides with the expected mass (S1 Table). One of the nine peptides (trifolitoxin) expressed as a HIS_6_ fusion and two of the nine (trifolitoxin and subtilosin A) expressed as RST fusions were expressed and purified, but the masses observed matched peptides cleaved by site-specific *E*. *coli* proteases at internal cleavage sites (S1 Table).

### Production of active haloduracin

**Fig 3:**
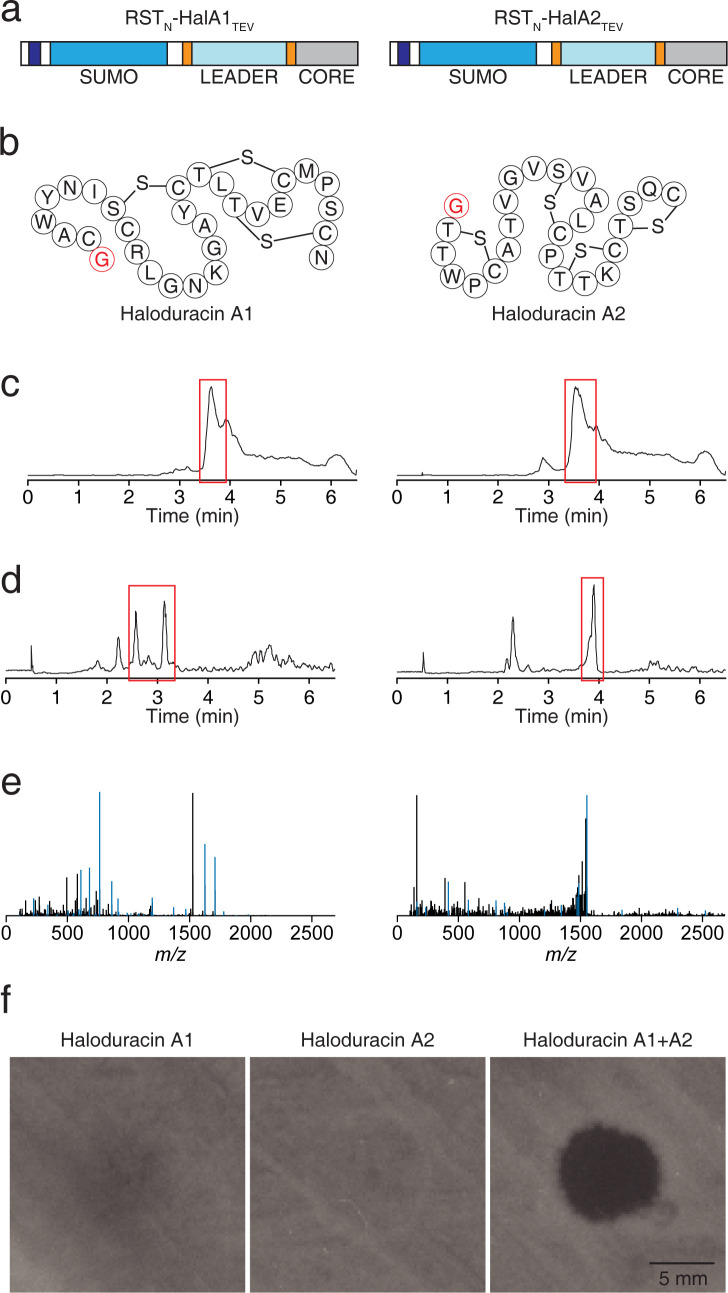
Production of active haloduracin when fused to RST_N_. **a** Schematic of peptides from the haloduracin cluster, engineered to have TEV cleavage sites in between the leader and core peptides. **b** The expected structures after TEV cleavage are shown, with the glycine residue left by TEV colored red. **c** LC-MS TICs of haloduracins A1 and A2 after affinity purification, with peak corresponding to SUMO-tagged peptides boxed in red. **d** LC-MS TICs of haloduracins A1 and A2 after cleavage with TEV protease and purification, with peaks corresponding to peptides boxed in red. **e** LC-MS/MS fragmentation spectra of cleaved haloduracins A1 and A2, with masses that match fragments of structure in b, highlighted in blue (full annotation is in S2–S5 Notes). **f** Halo-assay of haloduracin A1, A2, and A1+A2 antimicrobial activity, using *B*. *subtilis* as the reporter strain (solvent negative control in S2 Fig). Data in panels c/d has been collected twice with similar results (once via “haloduracin production and purification” method and once via “peptide purification for MS/MS data analysis” method). MS/MS data in panel e represents a single replicate and halo-assay data in panel f is a representative example of data collected in triplicate (S2 Fig).

We then validated the production of a biologically-active product using our expression system. Modifications are directed at an RST-fused peptide, after which the tag is cleaved and activity tested. We selected haloduracin, which is a two-component lanthipeptide that had been previously expressed and purified from *E*. *coli* and shown to have antibiotic activity [34]. We synthesized the genes for the haloduracin A1 and haloduracin A2 peptides (fused to the final RST_N_ version with Link-2) and corresponding HalM1 and HalM2 modifying enzymes from *Bacillus subtilis* (Fig 3). An additional TEV protease cleavage site was added between the leader and core regions of the precursor peptide (Fig 3A) so that the core could be cleaved and recovered as the active product (Fig 3B). This leaves a single N-terminal glycine on the released core sequence. The peptide and enzyme genes were cloned into the two-plasmid system (Fig 1B) and co-transformed into *E*. *coli* NEB Express (Methods).

We developed a high-throughput 96-well system for expression and purification, which we tested using haloduracin (Methods). Cultures were grown in 2 mL of TB media in deep well plates (two 1 mL wells for each peptide), where they were each induced with 1 mM IPTG/200 μM cumate for 20 hours at 30°C with shaking. The cells were lysed, affinity-purified, and desalted using solid phase extraction, all in 96-well format. Then, the samples were treated with TEV protease to remove RST_N_ and the leader peptide and desalted again to concentrate the core peptide (Fig 3D) (Methods). The presence of the cleaved cores was verified by LC-MS (Fig 3D) and LC-MS/MS to investigate if SUMO disrupted or altered the lanthionine macrocyclizations present in both molecules (Fig 3E and S2–S3 Notes) (Methods). For both, the predicted structures were in close agreement with previously reported structural data [34, 58, 59]. For HalA2, seven of eight Ser/Thr residues were dehydrated and LC-MS/MS-based assignment of the single unmodified residue has been previously localized to Thr18, Thr22, or Ser23 [34, 58, 59]. We observed the correct number of dehydrations, and fragmentation that supported dehydration of Thr18, with Thr22 or Ser23 left unmodified. A previous report showed that the mutation S23A did not change the number of dehydrations [58], which agrees with our data, noting that we were not able to fully verify this structure through LC-MS/MS.

For the antimicrobial assay, the cleaved and desalted core peptides were resuspended in 50 μL 1:1 methanol:water. *Bacillus subtilis* PY79 was used as indicator strain and was spread on a LB-agar surface, on which 5 μL of either or 2.5 μL of both haloduracins (Figs 3F and S2) or a solvent control was added (S2 Fig) (Methods). Individually, they showed limited activity, but combined they form a clear halo of growth inhibition, suggesting that both peptides were properly modified and cyclized.

### High-throughput assay of diverse modifying enzymes

**Fig 4:**
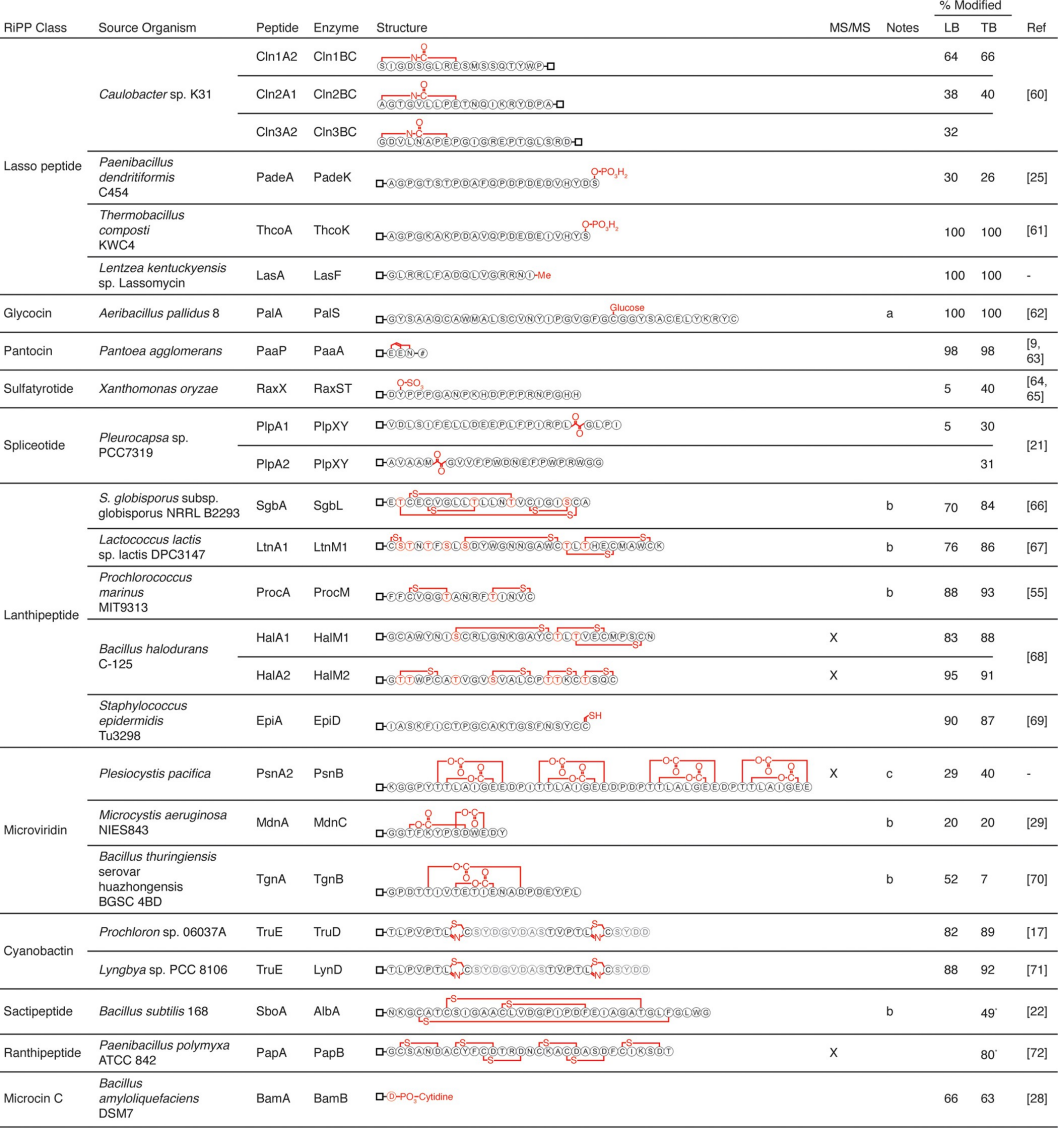
Successfully enzyme-modified peptides. RiPP class, source, gene names, and previously published structure for successfully observed modifications. Modified peptides analyzed with MS/MS are demarcated with an “X” in the MS/MS column. Potential structural ambiguity is listed in the Notes column: Blank—the collected LC-MS or LC-MS/MS data confirms the published structure or is consistent with published data for the structure, a–multiple cysteines present could be glycosylated and cannot be differentiated by LC-MS, b–cross-linking pattern was not validated and could differ from wild-type structure shown, c–MS/MS shows four distinct intra-modified cores, but does not validate cross-linking pattern within each core. Mean percent modified is listed for each modification in LB and TB medias, with individual replicates plotted in S4 Fig. Fraction modified for ThcoA/K is the sum of mono- and di-phosphorylated peptide. Percentages marked with an asterisk (*) have a modification with a small mass shift–percent modified may be inaccurate due to isotope overlap between unmodified/modified peptides (SboA/AlbA cleaved spectrum validation in S5 Fig; PapA/PapB in S5 Note). References are listed for modifications that have previously been shown in *E*. *coli*.

We collated a set of 46 modifying enzymes and their cognate 50 precursor peptides from the literature. The complete list of pathways and enzymes is provided in S2–S3 Tables whereas the subset we ultimately found to be active are in Fig 4. These are representative of 13 bacterial RiPP classes from diverse genera and catalyze 22 different chemical transformations, including glycosylation, radical carbon-carbon bond formation and cysteine heterocyclization (S3 Table). The precursor peptide and modifying enzyme genes were codon optimized for *E*. *coli* and synthesized, or amplified when the source DNA was available, and cloned into the two-plasmid system (Methods). The precursor peptides were tagged with RST_N_, except for macrocyclization of lasso peptides, which were fused to RST_C_. The plasmids containing the modifying enzymes and precursor peptides were co-transformed into *E*. *coli* NEB Express.

The cultures were grown following the high-throughput protocol in 96-well plates (Methods). Both TB media and LB media have been used to functionally express RiPPs in *E*. *coli*. The choice of media can impact the function of an enzyme; for example, radical S-adenosyl-L-methionine (rSAM) enzymes are more active in TB than LB, the latter requiring a reduction in shake speed and/or increased iron-sulfur cluster biosynthesis [22, 60, 61]. For applications requiring the high-throughput mining or the artificial combination of RiPP enzymes, it is desirable to have a single set of culture conditions. To this end, we evaluated the ability for the enzymes to modify their precursor peptides following the same culture conditions either in LB or TB (Fig 4). Other conditions, such as temperature, dissolved O_2_ and incubation time are known to affect enzyme activity and specificity, but we sought here to survey enzyme activity across a common set of conditions for recombinant expression.

All of the enzymes and precursor peptides were expressed in 1 mL of media in deep-well plates with shaking (Methods). Induction by 1 mM IPTG and 200 μM cumate was performed for 20 hours at 30°C, after which the modified peptide was purified. In all cases, the modification could be observed with LC-MS without cleaving RST_N_. In total, 24/46 (52%) of the enzymes tested were active against at least one peptide in one of the medias tested, two of which had not previously been reported expressed in *E*. *coli*. The “% modified” values shown in Fig 4 were calculated from the extracted compound chromatograms (ECCs) based on the expected charge state *m/z*’s for unmodified, partially modified (if relevant) and modified peptide molar masses (S3 Fig). The ECC peaks were fit with a Gaussian and the areas under the curve calculated. The “% modified” was calculated by dividing the area of the peak associated with the modification by the total areas of modified and unmodified (and partially modified, if relevant) (Methods). More enzymes (24) had activity in TB media than LB media (20) and, on average, the % modified is higher. As expected, rSAM enzymes (AlbA, PapB, PlpXY) are more active in TB media and several only have activity in this media. Similarly, RaxST is a sulfur-requiring enzyme that is more active in TB media. Previous expression of this enzyme in LB has relied on co-expression of RaxP and RaxQ to biosynthesize the sulfur donor 3’-phosphoadenosine 5’-phosphosulfate (PAPS) [62].

The 25 modified peptides shown in Fig 4 showed the exact mass change that is expected to result from the modification. The amino acid specificities of some of the enzymes restrict the mass change to only one possible modified peptide product; there are 14 in this category (S4 Table) [21, 25, 28, 63–69]. However, some modifications could occur at different positions than the wild-type modification, leading to a different peptide with the same mass [19, 22, 29, 55, 70–75]. When multiple products are possible, the addition of an RST could change where the modification occurs (Fig 4). To test for this outcome, we selected several modifications from different classes to evaluate by LC-MS/MS (Methods). In addition to HalA1/HalA2 investigated above, the following were selected for structural annotation: PsnA2 macrolactonization by PsnB and PapA sactionine macrocyclization by PapB. The precursor peptides were modified to contain a TEV cleavage site between the leader and core peptides. The modifying enzymes and precursor peptides were expressed following the high-throughput protocol, the RST_N_ and leader peptide removed using TEV protease, and the modified core analyzed with LC-MS/MS (Methods). Fragmentation of PsnA2 was observed between the core repeats, with each core repeat fragment mass corresponding to two lactone macrocyclizations per repeat, in agreement with previously published results [19]. Similarly, fragmentation of PapA was observed between CxxxD motifs in the core, indicating that the correct CxxxD cycles have formed rather than other cyclization patterns.

**Fig 5:**
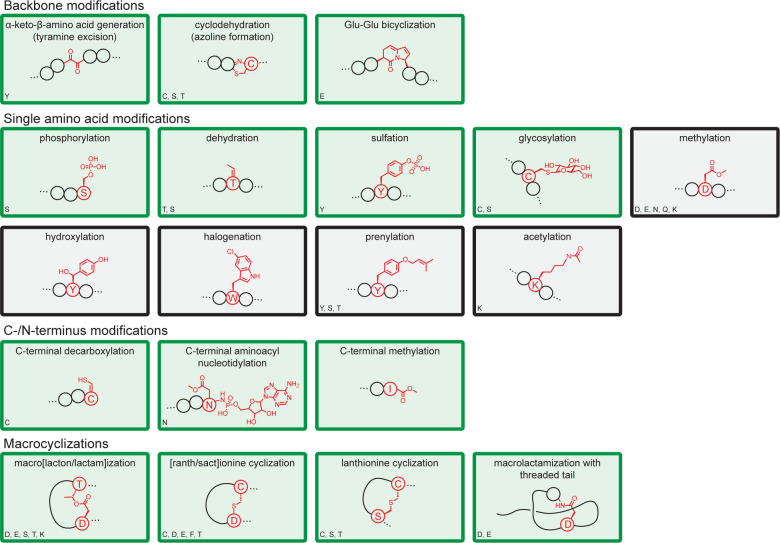
Summary of RiPP chemical modifications. All enzyme modification chemistries used in this publication. Example structures are shown in each box, with the modified residues/structures highlighted in red. Amino acids involved are listed in the lower left of each box (if amino acids are chemically restrictive). Green boxes are those for which we observed functional enzymes in our expression system. Black boxes were tested, but no tested enzymes were functional.

Of the enzymes tested, 20 of the 46 did not modify a peptide at all and 3 of the 46 modified the peptide incorrectly or generated a complex mixture of modification products. This includes 14 clusters for which there are examples of successful expression in *E*. *coli* using a variety of expression methods (S2 Table). We looked for patterns based on the phylogeny from which the pathway was sourced, noting that they span cyanobacteria, actinobacteria, proteobacteria, and firmicutes (S6 Fig). There are successful examples from each of these phyla. The least successful phylum, Actinobacteria, yielded 2/7 functional pathways. We do not observe a relationship between similarity to *E*. *coli* and the likelihood of success. Most classes of chemical transformation had at least one example that was functional (Fig 5). However, both prenyl transferases (ComQ and KgpF) and all three P450 oxidases (MibO, CinX, and PbtO) were not functional. Other possible explanations for an enzyme to not modify include disruption of the modification by the SUMO tag, the necessity of other enzymes or genes from the native pathway to support modification, non-functional RBSs, co-factor availability, toxicity of the modified peptide and incompatible expression conditions.

## Discussion

While the number of characterized RiPP enzymes is growing rapidly in the literature, the conditions under which each enzyme is characterized vary across studies. This poses a challenge for high-throughput screening efforts if the conditions have to be re-optimized for each pathway. This manuscript presents a side-by-side survey of recombinant RiPP enzymes in *E*. *coli*, using the same growth and induction methods. Further, protocols are developed for every step to be performed in 96-well plate format under conditions that are consistent with high-throughput screening platforms [2, 76–78]. The RSTs address the problem of precursor peptide stability, for which degradation and solubility are likely the dominant causes of unobservable substrate. Their use increases the probability that a pathway will be successfully expressed in a new host; in other words, they increase the “hit rate” of screening efforts. The SUMO-based RSTs are compatible with many modifying enzymes, facilitate high-throughput purification and do not need to be removed prior to LC-MS analysis of modifications. Software was developed to rapidly analyze LC-MS data (Methods). Collectively, this presents a suite of tools that enable the high-throughput screening of RiPP pathways mined from sequence databases [13, 79, 80]. In this manuscript, we only look at the action of a single enzyme. To mine complete RiPP-encoding gene clusters, additional enzyme genes can either be assembled as operons or placed under the control of different inducible promoters (*e*.*g*., *E*. *coli* Marionette) [45].

The fraction of enzymes found to be functional in *E*. *coli* under common conditions is surprisingly high, especially considering the diversity in the source genera and chemistries. In our experience, it is much higher than the successful transfer of other natural products genes, such as non-ribosomal peptide synthases, which also produce peptidic products. These results imply that RiPP enzymes can be combined from different sources to create synthetic pathways from which all the enzymes are functionally expressed. Indeed, several examples have been published demonstrating the artificial combination of RiPP enzymes from different source species and pathways to make products not observed in nature [30, 81, 82]. Knowing that roughly half of RiPP enzymes are functionally compatible with *E*. *coli* expands the potential peptide chemical space that can be explored through the artificial mixing-and-matching of these enzymes. Fully enabling this requires a better understanding of the rules for designing precursor peptides that can be acted on by multiple modifying enzymes. Collectively, these tools for the mining and *de novo* design of RiPPs will allow the exploration of the vast universe of modified peptides for novel antibiotics, intercellular communication channels, and signaling molecules that influence animal and plant physiology.

## Materials and methods

### Strains, plasmids, media, and chemicals

*E*. *coli* NEB 10-beta (C3019I, New England BioLabs, Ipswich, MA, USA) was used for all routine cloning. *E*. *coli* BL21 (C2530H, New England BioLabs, Ipswich, MA, USA) was used to characterize RSTs and linker variants in low-throughput (flask) cultures. *E*. *coli* NEB Express (C2523I, New England BioLabs, Ipswich, MA, USA) was used to express all other experiments. All plasmids containing RST-fused precursor peptide genes use a pSC101 origin variant (var 2) with ampicillin resistance (S1 Fig and S7 Table) [83]. All plasmids carrying modifying enzyme genes contain p15A origins of replication and kanamycin resistance. LB-Miller media (B244620, BD, Franklin Lakes, NJ, USA) or TB media (T0311, Teknova, Hollister, CA, USA) supplemented with 0.4% glycerol (BDH1172-4LP, VWR, OH, USA) were used for peptide expression and modification. 2xYT liquid media (B244020, BD, Franklin Lakes, NJ, USA) and 2xYT + 2% agar (B214010, BD, Franklin Lakes, NJ, USA) plates were used for routine cloning and strain maintenance. SOB liquid media (S0210, Teknova, Hollister, CA, USA) was used for making competent cells. SOC liquid media (B9020S, New England BioLabs, Iwsich, MA, USA) was used for outgrowth. Cells were induced with the following chemicals: cumate (cuminic acid) ≥98% purity from Millipore Sigma (268402, Millipore Sigma, Saint Louis, MO, USA) added as 1000X stock (200 mM) in EtOH or DMSO; isopropyl β-D-1-thiogalactopyranoside (IPTG) ≥99% purity (I2481C, Gold Biotechnology, Saint Louis, MO, USA) added as 1000X stock (1 M) in water or DMSO; 3OC6-AHL from Millipore Sigma (K3007, Millipore Sigma, Saint Louis, MO, USA) added as a 1000X stock (10 mM) in DMF. Cells were selected with the following antibiotics: 50 μg/ml kanamycin (K-120-10, Gold Biotechnology, Saint Louis, MO, USA); 100 μg/ml carbenicillin (C-103-5, Gold Biotechnology, Saint Louis, MO, USA); 30 μg/ml chloramphenicol (B20841, Alfa Aesar, Ward Hill, MA, USA). Liquid chromatography was performed with Optima Acetonitrile (A996-4, Thermo Fisher Scientific, MA, USA) and water (Milli-Q Advantage A10, Millipore Sigma, Saint Louis, MO, USA) supplemented with LC-MS Grade Formic Acid (85178, Thermo Fisher Scientific). The following solvents/chemicals were used: Ethanol (V1001, Decon Labs, King of Prussia, PA, USA), Methanol (3016–16, Avantor, Center Valley, PA, USA), dimethyl sulfoxide (DMSO) (32434, Alfa Aesar, Ward Hill, MA, USA), Imidazole (IX0005, Millipore Sigma, Saint Louis, MO, USA), sodium chloride (X190, VWR, OH, USA), sodium phosphate monobasic monohydrate (20233, USB Corporation, Cleveland, OH, USA), sodium phosphate dibasic anhydrous (204855000, Acros, NJ, USA), guanidine hydrochloride (50950, Millipore Sigma, Saint Louis, MO, USA), tris (75825, Affymetrix, Cleveland, OH, USA), TCEP (51805-45-9, Gold Biotechnology, Saint Louis, MO, USA), and EDTA (0.5M stock, 15694, USB Corporation, Cleveland, OH, USA). DNA oligos and gblocks were ordered from Integrated DNA Technologies (San Francisco, CA, USA).

### Gene design

A list of plasmids and corresponding plasmid maps are provided in S5 Table and S1 Fig. Amino acid sequences of all modifying enzymes and peptides are provided in S6 Table. Sequences of genetic parts and full plasmids are in S7–S8 Tables. Unless stated otherwise, modifying enzymes and peptides were codon optimized for *E*. *coli*, filtered for Type IIs restriction sites (SapI, BsaI, BbsI, BsmBI), and synthesized and cloned by Twist Biosciences (San Francisco, CA, USA) into custom backbones. The following genes were obtained from other sources: *bmbC*, *truE*, *pgm2*, *strA*, and *paaP* were codon optimized for *E*. *coli* and synthesized/cloned using DNA oligos; *sboA* and *albA* were amplified from the genome of *Bacillus subtilus* subsp. spizizenii (ATCC 6633); *mdnA* and *mdnC* were amplified from pARW071 [29] and mdnA* is a leader truncation of *mdnA*; *pqqA* was amplified from the genome of *Klebsiella variicola* str. 342; *tfxA* was amplified from the genome of *Rhizobium leguminosarum* Jordan (ATCC 53912); *mcbCD* and *mcbA* were synthesized and cloned by Twist without codon optimization (sequences were sourced from *E*. *coli*); *truD* was amplified from plasmid Topo-E1 [17]; *paaA* was codon optimized for *E*. *coli* and synthesized as a gblock by IDT. Ribosome binding sequences for each modifying enzyme were designed using the RBS calculator [49, 50] with a target translation initiation rate of 20,000–30,000. The RBS for peptide expression was optimized with the RBS Calculator to maximize expression rate. The HIS_6_ tag for peptide expression (ATag-1, S1 Fig and S7 Table) is taken from the pTEV5 construct reported [46] for expression without SUMO (plasmids pEG3044 –pEG3055). With SUMO the first 10 amino acids of the pTEV5 construct were used as a HIS_6_ tag to limit the effect of the gene sequence on RBS translation rate. The SUMO sequence and the 4 amino acids N-terminal to SUMO were synthesized as a gblock with sequence from pE-SUMO vector (1001K, LifeSensors, Malvern, PA, USA). For initial testing of the RST_N_ construct in Figs 1C and 2, the linker between SUMO and the precursor peptide was adapted from the pTEV5 tag to generate Link-1 (plasmids pEG3058 –pEG3067). For future experiments with RST_N_, the repeated HIS_6_ sequence was removed, a TEV cleavage site was added, a cysteine after the SUMO cut site and after the TEV cut site was added, and several other amino acids were added to increase the length of the linker to generate Link-2 (plasmids pEG3121 –pEG3132 and pEG3248). A later iteration also added BsaI sites around the operon for subcloning (plasmids pEG2192 –pEG2575, pEG3157 –pEG3197, pEG3283 –pEG3286, and pEG3563 –pEG3905). When using RST_C_, the N-terminal affinity tag is maintained, but the four amino acids between HIS_6_ and SUMO were removed (ATag-3). For the linker (Link-3), the TEV cleavage site was removed from the C-terminus of the linker and a thrombin site was added to the N-terminus. An affinity tag was also added to the C-terminus of SUMO (ATag-4) in case the leader (and tag) was removed during modification (plasmids pEG3212 –pEG3215 and pEG3553 –pEG3562). Modifying enzymes and their individual RBSs are expressed using pLux in pEG1128 or pCym in pEG7034–7171 (flanking SapI sites around RBS+CDS), except pEG7172 and pEG7173 which lacked flanking SapI sites.

### Peptide expression/modification from flasks and purification

Plasmids were transformed into *E*. *coli* BL21, struck out on 2xYT agar with carbenicillin (or chloramphenicol for pEG3017) and kanamycin (if co-transforming modifying enzyme) and incubated (30°C, overnight). Individual colonies were used to inoculate 3 mL of LB media in a culture tube (352059, Corning, NY, USA) and incubated overnight (30°C, 250 r.p.m.) in an Innova44 (Eppendorf, NY, USA). Aliquots (500 μl) were taken from the overnight cultures and subcultured into 50 mL of LB media in a 250 mL Erlenmeyer flask. After 3 hours incubation (Innova44, 30°C, 250 r.p.m.), IPTG and 3OC6-AHL (if inducing modifying enzyme) was added to final concentrations of 1 mM and 10 μM and cultures were incubated for 20 hours (Innova44, 18°C, 250 r.p.m.) (note: IPTG was not added for pEG3017, where the MBP-tagged peptide is constitutively expressed). The 50 mL cultures were transferred to a falcon tube (352070, Corning, NY, USA), centrifuged (4,500g, 4°C, 20 min) in a Sorvall Legend XFR Centrifuge (Thermo Fisher Scientific, MA, USA), pellets were resuspended in 600 μl lysis buffer (5 M guanidinium hydrochloride, 300 mM NaCl, 50 mM sodium phosphate, pH 7.5), and freeze-thawed twice (frozen in -80°C freezer; thawed in innova44 incubator at 30°C, 250 r.p.m). Cell lysates were centrifuged (Eppendorf 5424, 21,130g, room temperature, 15 min) in an Eppendorf 5424 Centrifuge (Eppendorf, NY, USA) and the peptides affinity purified using His SpinTrap TALON columns (29-0005-93, GE Life Sciences (now Cytiva), Marlborough, MA, USA), following manufacturer instructions, using 600 μL lysis buffer twice for column equilibration, loading 600 μL clarified lysate, two washes with 600 μL wash buffer (300 mM NaCl, 50mM sodium phosphate, 5 mM imidazole, pH 7.5), and 200 μL elution buffer (300mM NaCl, 50mM sodium phosphate, 200 mM imidazole, pH 7.5) for elution. Purifications used an Eppendorf 5424 centrifuge.

### Liquid chromatography/mass spectrometry

All chromatography was performed using mobile phases ACN (acetonitrile supplemented with 0.1% formic acid and 0.1% water) and water (supplemented with 0.1% formic acid). LC-MS was performed on one of two mass spectrometers: “QQQ” is an Agilent 1260 Infinity liquid chromatograph with binary pump configured in low-dwell volume mode, high-performance autosampler chilled to 18°C, and column oven, coupled to an Agilent 6420 QQQ mass spectrometer equipped with an Agilent electrospray ionization (ESI) source; nitrogen gas is supplied by a Parker Nitroflowlab and ESI source parameters are 350°C gas temp at 12 L/min flow rate, 15 psi nebulizer voltage, 4000 V capillary voltage, 135 V fragmentor voltage, and 7 V cell accelerator voltage. “QTOF” is an Agilent 1260 Infinity II liquid chromatograph with binary pump configured in low-dwell volume mode and column oven set to 40°C, coupled to an Agilent 6545 QTOF mass spectrometer equipped with an Agilent electrospray ionization (ESI) source; nitrogen gas is building supplied and ESI source parameters are 350°C gas temperature, 12 L/min gas flow, 30 psig nebulizer pressure, 350°C sheath gas temperature, 8 L/min sheath gas flow, 3000 V capillary voltage, 1000 V nozzle voltage, 135 V fragmentor voltage, 15 V skimmer voltage, 600 V Oct 1 RF Vpp; the mass spectrometer was run in MS mode with reference mass enabled and tuned in positive mode with standard mass range (3200 *m/z*) and 2 GHz extended dynamic range. A list of peptide extracts analyzed is provided in S4 Table, which details the machine used for each extract. For extracts 192–202: analysis was done with a Phenomenex Aeris Widepore XB-C18 3.6 μm 150 mm x 2.1 mm column with column oven set to 30°C. Flow rate was 0.5 ml/min. Gradient was 3% ACN for 3 min, 3% to 54% ACN over 34 min, 54% to 97% ACN over 3 min, 97% ACN for 5 min, with 5 min re-equilibration. The mass spectrometer was run in positive mode 100–2250 *m/z* range with a 200 ms scan time. Injections were 20 μL. For extracts 216–225: analysis was done with a Phenomenex Aeris Widepore XB-C18 3.6 μm 150 mm x 2.1 mm column with column oven set to 30°C. Flow rate was 0.5 mL/min. The gradient was 10% ACN for 1 min, 10% to 15% ACN over 1.5 min, 15% to 50% ACN over 52.5 min, 50% to 97% ACN over 3 min, 97% ACN for 2 min, with 5 min re-equilibration. The mass spectrometer was run in positive mode 100–2250 *m/z* range with a 200 ms scan time. Injections were 20 μl. For all other extracts analyzed with “QQQ”: analysis was done with a Phenomenex Aeris PEPTIDE XB-C18 2.6 μm 50 mm x 2.1 mm column with column oven set to 40°C. Flow rate was 0.6 mL/min. Gradient was 20% ACN for 0.5 min, 20% to 47% ACN over 4.5 min, 47% to 90% ACN over 0.5 min, 90% ACN for 1 min, with 0.8 min re-equilibration. The mass spectrometer was run in positive mode, 500–2000 *m/z* range with a 300 ms scan time. Injections were 10 μL for extracts from LB media and 5 μL for extracts from TB media (as a starting point, injection volumes were occasionally adjusted depending on the yield of the 96-well prep). For all extracts analyzed with “QTOF”: analysis was done with a Phenomenex Aeris PEPTIDE XB-C18 2.6 μm 50 mm x 2.1 mm column. Flow rate was set at 0.5 mL/min. The gradient used was 20% ACN for 0.5 min, 20% to 55% ACN over 5.5 min, 55% to 90% ACN over 0.5 minutes, 90% ACN for 1.5 min, with 0.8 min re-equilibration. Injections were 5 μL for extracts from LB media and 2 μL for extracts from TB media (as a starting point, injection volumes were occasionally adjusted depending on the yield of the 96-well prep).

### Calculation of peptide molar masses

For large peptides/proteins, mass was calculated as described for ESIprot [84]: five consecutively charged m/z’s (*m*_1_, *m*_2_, *m*_3_, *m*_4_, *m*_5_) were taken from the spectra and used to calculate the charge states (*z*_1_, *z*_2_, *z*_3_, *z*_4_, *z*_5_) for each of the peaks. For peaks *m*_1_ and *m*_2_, which have charge states, *z*_1_ and *z*_2_, where *z*_2_ = *z*_1_−1 (peak 1 has one proton more than peak 2): *z*_1_ = (*m*_2_-1)/(*m*_2_-*m*_1_). Charges *z*_1_, *z*_2_, *z*_3_, and *z*_4_ were calculated using each of the four pairs of consecutively charged masses (*m*_1_ and *m*_2_, *m*_2_ and *m*_3_, *m*_3_ and *m*_4_, *m*_4_ and *m*_5_), subtracted by the number of protons the peak has compared to *m*_5_, and averaged together and rounded to the nearest integer to calculate the lowest charge (*z*_5_). Charges *z*_1-4_ are recalculated based on charge *z*_5_ (*z*_1_ = *z*_5_ + 4, *z*_2_ = *z*_5_ + 3, etc.), uncharged masses are calculated from each of the five *m/z*’s: uncharged mass = (z_x_ • *observed m/z*)–z_x_. The mean and standard deviation was calculated from each observed *m/z* to give the average observed mass and mass standard deviation. The final reported standard deviation is the greater of either the mass standard deviation or the standard deviation calculated from the manufacturer-specified resolution of the mass spectrometer. For the QQQ mass spectrometer: peak full width half max (FWHM) = 0.7, σ = 0.3 is scaled by the largest charge state observed to give the standard deviation < 0.3 *z*_1_. For the QTOF, FWHM = 0.06, σ = 0.026, standard deviation < 0.026 *z*_1_.

### Peptide expression in 96-well plates

Plasmids were transformed into *E*. *coli* NEB Express using 15–20 μL of competent cells and 1 μL of each plasmid being transformed in a 96-well PCR plate (1402–9596, USA Scientific, FL, USA or 951020401, Eppendorf, NY, USA). Transformations were incubated on ice (20–30 min), heat shocked (42°C, 30 sec), and incubated on ice again (5 min). Cells were then transferred to a deep well 96-well plate (1896–2000, USA Scientific, FL, USA) with 100 μL of SOC media. After outgrowth (Multitron Pro, 1 hr, 37°C) in an Infors HT Multitron Pro (Infors USA, MD, USA), 400 μL LB media was added with appropriate antibiotics (100 μg/ml carbenicillin and 50 μg/ml kanamycin) and incubated (Multitron Pro, 30°C, 900 r.p.m.) until all wells reached stationary phase (cultures were visibly saturated, 12–30 hours). Overnight cultures were diluted 1:100 into 1 mL LB or TB media with antibiotics in deep well plates. After a 3 hour incubation (Multitron Pro, 30°C, 900 r.p.m.), appropriate inducer was added (1 mM IPTG and 200 μM cumate) and cultures were incubated for 20 hours (Multitron Pro, 30°C, 900 r.p.m.). The 96-well plates were centrifuged (Legend XFR, 4,500 g, 4°C, 20 min) and media discarded. Pellets were stored at -20°C until purification.

### Haloduracin production and purification

Haloduracin was produced following the 96-well expression protocol described above, with each sample being produced in two wells of 1 mL TB media to double the amount of product produced. Culture pellets were resuspended in 800 μL lysis buffer, freeze-thawed (frozen at -80°C; thawed in Multitron Pro at 37°C, 900 r.p.m), and centrifuged (Legend XFR, 4,500 g, 4°C, 30 min). Peptides were affinity purified using HIS MultiTrap TALON plates, using 500 μL water and two 500 μL lysis buffer washes for column equilibration (Legend XFR, 500 g, 4°C, 2 min), loading 600 μL of both matching sample’s clarified lysates iteratively (load one, then centrifuge, then load the second, then centrifuge) (Legend XFR, 100 g, 4°C, 5 min), washing twice with 500 μL wash buffer, and eluting three times with 200 μL elution buffer to maximize titer. Purification was followed by solid-phase extraction (SPE) using Strata-XL microtiter plates (8E-S043-TGB, Phenomenex, CA, USA). Plates were conditioned with 1 mL methanol wash followed by 1 mL water wash. All 600 μL of TALON eluent was loaded, washed twice with 1 mL water, and then eluted twice with 500 μl 1:1 acetonitrile:water (supplemented with 0.1% formic acid). Plates with eluent were dried down at room temperature in a Savant Speedvac SPD2010 (Thermo Fisher Scientific, MA, USA), samples resuspended in 40 μL TE buffer (10 mM tris, 1 mM EDTA) with 20 μL 2 mg/mL TEV protease, and then incubated (stationary, 30°C, 8 hr). Cut fractions were desalted using a Strata-X SPE plate (8E-S100-TGB, Phenomenex, CA, USA) with same condition/wash/elution/drying steps as above. Dried down samples were resuspended in 50 μL 1:1 methanol:water.

### LC-MS/MS data acquisition

Chromatography was performed using the same ACN/water mobile phases as for LC-MS. MS/MS data were acquired on an Agilent 1260 Infinity II liquid chromatograph with binary pump configured in low-dwell volume mode and column oven set to 40°C, coupled to an Agilent 6545 QTOF mass spectrometer equipped with an Agilent electrospray ionization (ESI) source. Nitrogen gas is building-supplied and ESI source parameters are 350°C gas temperature, 12 L/min gas flow, 30 psig nebulizer pressure, 350°C sheath gas temperature, 8 L/min sheath gas flow, 3000 V capillary voltage, 1000 V nozzle voltage, 135 V fragmentor voltage, 15 V skimmer voltage, 600 V Oct 1 RF Vpp; the mass spectrometer was run in MS mode with reference mass enabled and tuned in positive mode with standard mass range (3200 *m/z*) and 2 GHz extended dynamic range. For the targeted MS/MS, the parent ion was selected, and 4 spectra/s were sampled with fixed collision energies of 30, 45, 60, and 75 V. A narrow isolation width (1.3 m/z) and observed monoisotopic mass (exact masses found in S1–S5 Notes) was used for fragmentation of each peptide. Sample analysis was performed with a Phenomenex Aeris PEPTIDE XB-C18 2.6 μm 50 mm x 2.1 mm column. The flow rate was set at 0.5 mL/min and 5 μl sample was injected. The gradient used was 10% ACN for 1.0 min, 10% to 70% ACN over 5.0 min, 70% to 90% ACN over 0.5 minutes, 90% ACN for 1.0 min, with 1.0 min re-equilibration. Accurate mass predictions of peptides were generated using the online resource, ChemCalc [85]

### MS/MS data analysis and spectra annotation

A published algorithm [59] was adapted for Python from R-package. For each spectrum, all ion abundances were capped at an upper limit/ceiling calculated as the user-specified upper limit multiplied by the average peak abundance in the spectrum. Background peaks below a threshold were then removed in mass window slices (number of slices is user-specified), where the threshold was calculated as the average peak abundance in each slice and its flanking slices, multiplied by a user-specified signal-to-noise threshold. Hypothetical ions were then matched to the filtered MS/MS signals within a user-specified *m/z* tolerance. Hypothetical ion *m/z*’s were determined by first calculating the parent ion mass (H^+^ adduct of full-length peptide with modification). The peptide was iteratively truncated along its backbone from its N-terminus (for y ions) or its C-terminus (for b ions). The masses of these truncated peptides were calculated and made up the hypothetical ion masses. Only monoisotopic masses were considered. To avoid spectrum deconvolution, we enabled the algorithm to work with multiply charged masses. To do this, we calculated the expected *m/z* values for [M+H]^1+^, [M+2H]^2+^, and [M+3H]^3+^ charge states of all hypothetical ions for matching to observed *m/z* values.

### Haloduracin antimicrobial assay

*Bacillus subtilis* PY79 was used as indicator strain for purified haloduracins. Overnight cultures were resuspended in LB (OD_600_ = 0.1) in a Cary 60 UV-Vis spectrometer (Agilent Technologies, CA, USA) and then a cotton-tipped applicator (24-106-2S, McKesson, TX, USA) was used to spread on an LB-agar surface. After drying, 5 μL of purified haloduracin or solvent control was added to plate. For combined haloduracin A1 + A2, 2.5 μL of each purified peptide was added to the same spot. Strains were incubated (30°C, overnight) and zones of inhibition visualized.

### Proteolytic cleavage and removal of SUMO

For purification of haloduracin for antimicrobial assays, TEV protease was purified as described previously [86] [Addgene #8827, concentrated to 2 mg/mL in TEV buffer (25 mM Tris-HCl, pH 8.0, 50 mM NaCl, 1 mM TCEP, 50% glycerol)]. For MS/MS analysis, TEV protease was prepared as a 50 mg/mL solution of 10% (w/w) TEV lyophilizate (Gene and Cell Technologies, CA, USA) in TEV Buffer.

### Peptide purification in 96-well plates

Culture pellets were resuspended in 800 μl lysis buffer, and freeze-thawed (frozen in liquid nitrogen at -196°C; thawed in Multitron Pro at 37°C, 900 r.p.m). Cell lysates were centrifuged (Legend XFR, 4,500 g, 4°C, 30 min) and peptides affinity-purified using His MultiTrap TALON plates (29-0005-96, GE Life Sciences (now Cytiva), Marlborough, MA, USA), following manufacturer instructions with a centrifuge used for each wash/flow-through step, using 500 μL water and two 500 μL lysis buffer washes for column equilibration (Legend XFR, 500 g, 4°C, 2 min), loading 600 μL clarified lysate (Legend XFR, 100 g, 4°C, 5 min), washing twice with 500 μL wash buffer (Legend XFR, 500 g, 4°C, 2 min), and eluting with 200 μL elution buffer (Legend XFR, 500 g, 4°C, 2 min).

### LCMS data analysis and peak integration

LC-MS datafiles were converted to mzXML format using MassHunter (Agilent Technologies, Santa Clara, CA, USA). Data were exported in centroid format with no deconvolution and a minimum signal intensity of 1,000. mzXML files were parsed and imported into python to a long-form pandas dataframe and filtered for signals between 1–6 min and 500–2,500 Da. For each extract, the expected molecular weight of unmodified, modified, and partially modified (if applicable) peptides were calculated based on the peptide sequence and the molar weights of each amino acid. Partial modification is only considered if the modification mass shift of individual modifications are ≥15 Da (S3 Table), since smaller shifts are not clearly resolved. For each modification state molecular weight, all charge state [M+xH]^x+^ (x is number of protons/charges) masses were calculated and extracted as an EIC with a mass window of +/- 2/x Da for extracts analyzed with “QQQ” and 1/x Da for extracts analyzed with “QTOF”. Charge state EIC intensities were summed together at each timepoint to generate an extracted compound chromatogram (ECC). If present, an ECC peak is fit with a skewed gaussian with parameters peak area, retention time, peak width, peak skew, and peak baseline. Peaks are considered real/trustworthy based on the following criteria: greater than eight charge states present/observed at the same retention time (+/- 0.2 min) with at least four being consecutive charge states, only one “large” peak in the ECC (*i*.*e*., there are no peaks greater than 80% of the largest peak height in the chromatogram), and not more than two “small” peaks (*i*.*e*., <3 peaks are greater than 40% of the largest peak height), peak skew between 0 and 1.5, peak width less than or equal to 0.25. Within an extract, “total peptide” is defined as the sum of the peak areas of unmodified, modified, and partially modified (if applicable). Fraction modified is defined as the modified peptide peak area divided by the “total peptide”. Peak integrations and masses for each extract are listed in the peak list file available on GitHub. Chromatograms, peak fits, and spectra for a representative replicate are shown in S5 Fig. All analyses were performed in Python 3.5 using pandas, scipy, numpy, and matplotlib libraries.

### Peptide purification for MS/MS analysis

Peptide/enzyme pairs presented with LC-MS/MS data in S2–S5 Notes were produced and purified following the protocol described for 96-well affinity expression and purification, except samples were eluted 3x with 200 μL elution buffer. After elution, 6 μL of TEV protease (Gene and Cell Technologies, USA) (50 mg of TEV lyophilizate/mL in TE buffer, lyophilizate is 10% TEV by weight) and 20 μL of TCEP (20 mM in water) were added to the eluent and incubated overnight at room temperature. A 20 μL aliquot of the sample was taken from this reaction and diluted 1:2 in TE buffer, centrifuged to remove precipitate, and analyzed with LC-MS/MS.

## Supporting information

S1 Fig Plasmid maps used in this study. **a.** Architectures of precursor peptide plasmid with MBP. **b.** Architectures of precursor peptide plasmids without SUMO (with ATag-1). **c.** Architectures of precursor peptide plasmids with the initially used RST_N_ (with Link-1). **d.** Architectures of plasmids with RST_N_ (with ATag-2 and Link-2). **e.** Architectures of plasmids with RST_N_, with flanking BsaI sites added around the operon for optional subcloning. **f.** Architectures of plasmids with RST_C_. **g.** Architecture of modifying enzyme plasmid with pLux promoter. **h.** Architectures of modifying enzyme plasmids with pCym promoter. **i.** Architecture of modifying enzyme plasmids with pCym promoter and SapI sites around RBS+gene for optional subcloning.
(PDF)

S2 Fig Haloduracin zones of inhibition. Each plate represents an individual replicate of Haloduracin A1 and A2 expression, purification, cleavage, and assaying for zone of inhibition. Quadrants are Haloduracin A1 (top right), Haloduracin A2 (top left), Haloduracin A1 and A2 (bottom right), and 50% methanol in water %vol/vol solvent control (bottom left).
(PDF)

S3 Fig Chromatograms of high-throughput peptide expression/modification samples. Spectra of the modified peaks are shown on the right. Chromatograms are grouped by peptide plasmid number and modifying enzyme plasmid number (numbers are shown without the preceeding “pEG”). Both expression medias are shown, with one replicate chosen for each and the extract number listed for the chosen replicate. Y-axis for all plots is Intensity in arbitrary units (x10^7^ for extracts analyzed with QQQ and x10^5^ for extracts analyzed with QTOF). From left to right, plots represent: TIC (black trace), ECC of unmodified peptide (green trace), ECC of partially modified peptide(s) (blue if passes peak thresholds or yellow otherwise), ECC of properly modified peptide (red trace), and mass spectrum. In ECC plots, peak fit is drawn as a black line. If a peak passes all thresholds, the peak area (x10^5^ for extracts analyzed with QQQ and x10^3^ for extracts analyzed with QTOF) is provided as a colored number in the top right corner of the plot. Partially modified peaks can have multiple numbers listed, one for each modification state (these are ordered ascending by magnitude top-to-bottom, left-to-right). All extracts are analyzed with QQQ unless there is an asterisk (*) next to the extract number, which denotes analyzed with QTOF. Peak lists and raw data are available for more detailed analysis on Zenodo.
(PDF)

S4 Fig Bar chart of successful peptide/modifying enzyme combinations. Peptide plasmid number and gene name, modifying enzyme plasmid number and gene name, and replicate extract numbers are listed alongside fraction modified in TB (dark grey) and LB (light grey) medias. Dashed line demarcates 50% modification (half of peptide modified). Three replicates are shown for each peptide/enzyme/media combination and represent independent expressions that were purified and assayed. Bar is shown at the replicate mean.
(PDF)

S5 Fig SboA+AlbA post-cleavage mass spectrum. Spectra of small mass-shift modification catalyzed by AlbA. Ion distribution for [M+4H]^4+^ is shown, with unmodified, modified, and observed *m/z*’s listed for the monoisotopic mass, which is labeled with an arrow. Data for cleaved peptide was collected once.
(PDF)

S6 Fig Phylogenetic tree species from which we mine enzymes. Tree was generated using the producing organisms listed in S2 Table and the NCBI Common Tree application. Species from which functional enzymes were sourced are black, nonfunctional are red.
(PDF)

S1 Note LC-MS/MS data legend. (PDF)

S2 Note HalA1+HalM1 MS/MS spectra. (PDF)

S3 Note HalA2+HalM2 MS/MS spectra. (PDF)

S4 Note PsnA2+PsnB MS/MS spectra. (PDF)

S5 Note PapA+PapB MS/MS spectra. (PDF)

S1 Table Flask-expressed peptide mass spectra masses. (PDF)

S2 Table Pathways investigated in this study. (PDF)

S3 Table Chemical modification types studied. (PDF)

S4 Table Structural validation of modified peptides. (PDF)

S5 Table New plasmids used in this work. (PDF)

S6 Table Enzyme and peptide amino acid sequences. (PDF)

S7 Table Genetic parts. (PDF)

S8 Table Plasmid sequences. (PDF)

S1 File Supplementary references. References from Supporting information figures/tables/notes.
(PDF)
