# Supplementary material for: Functional expression of diverse post-translational peptide-modifying enzymes in Escherichia coli under uniform expression and purification conditions
Source: PLoS One. 2022 Sep 19;17(9):e0266488. doi: 10.1371/journal.pone.0266488 (PMC9484694; doi:10.1371/journal.pone.0266488)
Supplement: S1 Fig — a. Architectures of precursor peptide plasmid with MBP. b. Architectures of precursor peptide plasmids without SUMO (with ATag-1). c. Architectures of precursor peptide plasmids with the initially used RSTN (with Link-1). d. Architectures of plasmids with RSTN (with ATag-2 and Link-2). e. Architectures of plasmids with RSTN, with flanking BsaI sites added around the operon for optional subcloning. f. Architectures of plasmids with RSTC. g. Architecture of modifying enzyme plasmid with pLux promoter. h. Architectures of modifying enzyme plasmids with pCym promoter. i. Architecture of modifying enzyme plasmids with pCym promoter and SapI sites around RBS+gene for optional subcloning. (PDF) [file pone.0266488.s001.pdf]

a

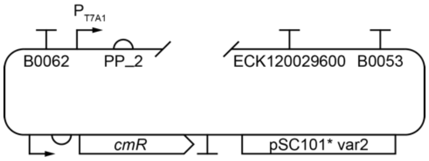

Name		
pEG3017	HIS_6-MBP	*truE**

b

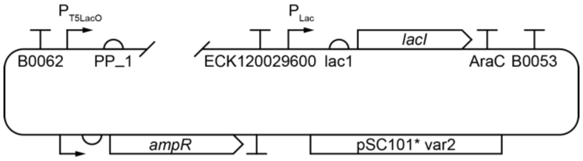

Name		
pEG3044	ATag-1	*truE**
pEG3045	ATag-1	*mdnA*
pEG3046	ATag-1	*bmbC*
pEG3047	ATag-1	*strA*
pEG3048	ATag-1	*pqqA*
pEG3049	ATag-1	*sboA*
pEG3051	ATag-1	*tfxA*
pEG3052	ATag-1	*procA1.7*
pEG3053	ATag-1	*tbtA*
pEG3055	ATag-1	*pgm2*

c

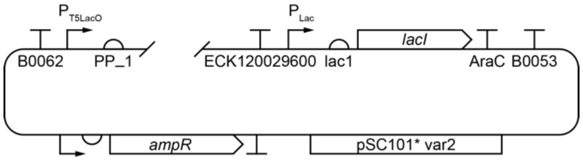

Name	RST_N			
pEG3058	ATag-2	SUMO	Link-1	*mdnA*
pEG3059	ATag-2	SUMO	Link-1	*sboA*
pEG3060	ATag-2	SUMO	Link-1	*pqqA*
pEG3061	ATag-2	SUMO	Link-1	*strA*
pEG3062	ATag-2	SUMO	Link-1	*bmbC*
pEG3063	ATag-2	SUMO	Link-1	*tfxA*
pEG3064	ATag-2	SUMO	Link-1	*procA1.7*
pEG3065	ATag-2	SUMO	Link-1	*tbtA*
pEG3067	ATag-2	SUMO	Link-1	*pgm2*

d

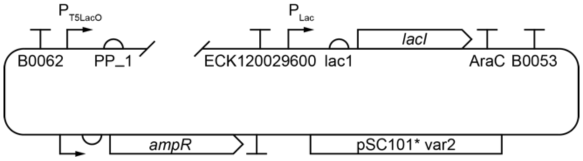

Name	RST_N			
pEG3121	ATag-2	SUMO	Link-2	*mdnA**
pEG3128	ATag-2	SUMO	Link-2	*procA**
pEG3132	ATag-2	SUMO	Link-2	*paaP*
pEG3248	ATag-2	SUMO	Link-2	*sboA*

e

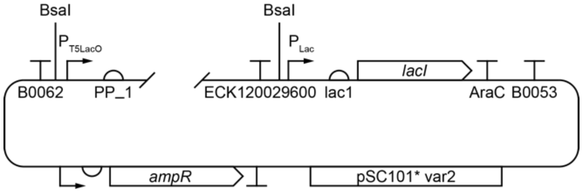

Name	RST_N			
pEG2192	ATag-2	SUMO	Link-2	*papoA*
pEG2194	ATag-2	SUMO	Link-2	*bamA*
pEG2195	ATag-2	SUMO	Link-2	*epiA*
pEG2199	ATag-2	SUMO	Link-2	*halA1*
pEG2200	ATag-2	SUMO	Link-2	*halA2*
pEG2312	ATag-2	SUMO	Link-2	*papA_tev*
pEG2571	ATag-2	SUMO	Link-2	*truE_tev*
pEG2575	ATag-2	SUMO	Link-2	*psnA2_tev*
pEG3157	ATag-2	SUMO	Link-2	*mibA*
pEG3161	ATag-2	SUMO	Link-2	*plpA1*
pEG3162	ATag-2	SUMO	Link-2	*plpA2*
pEG3165	ATag-2	SUMO	Link-2	*pbtA*
pEG3172	ATag-2	SUMO	Link-2	*ltnA1*
pEG3173	ATag-2	SUMO	Link-2	*ltnA2*
pEG3174	ATag-2	SUMO	Link-2	*crnA1*
pEG3175	ATag-2	SUMO	Link-2	*crnA2*
pEG3176	ATag-2	SUMO	Link-2	*bsjA2*
pEG3177	ATag-2	SUMO	Link-2	*bsjA3*
pEG3178	ATag-2	SUMO	Link-2	*cinA*
pEG3180	ATag-2	SUMO	Link-2	*lasA*
pEG3181	ATag-2	SUMO	Link-2	*albsA*
pEG3182	ATag-2	SUMO	Link-2	*mcbA*
pEG3194	ATag-2	SUMO	Link-2	*psnA2*
pEG3197	ATag-2	SUMO	Link-2	*amdnA*

pEG3283	ATag-2	SUMO	Link-2	*papA*
pEG3286	ATag-2	SUMO	Link-2	*pcpA*
pEG3563	ATag-2	SUMO	Link-2	*padeA*
pEG3564	ATag-2	SUMO	Link-2	*thcoA*
pEG3565	ATag-2	SUMO	Link-2	*stspA*
pEG3567	ATag-2	SUMO	Link-2	*lcnA*
pEG3568	ATag-2	SUMO	Link-2	*palA*
pEG3570	ATag-2	SUMO	Link-2	*raxX*
pEG3571	ATag-2	SUMO	Link-2	*comX*
pEG3572	ATag-2	SUMO	Link-2	*kgpE*
pEG3574	ATag-2	SUMO	Link-2	*tgnA**
pEG3871	ATag-2	SUMO	Link-2	*sgbA*
pEG3905	ATag-2	SUMO	Link-2	*truE*

f

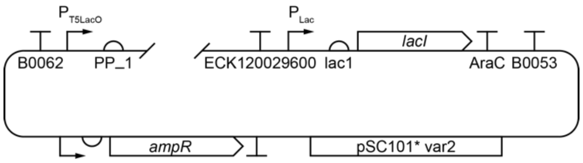

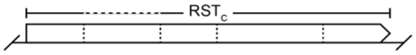

Name					
pEG3212	ATag-3	*capA*	Link-3	SUMO	ATag-4
pEG3213	ATag-3	*lasA*	Link-3	SUMO	ATag-4
pEG3214	ATag-3	*albsA*	Link-3	SUMO	ATag-4
pEG3215	ATag-3	*atxA1*	Link-3	SUMO	ATag-4
pEG3553	ATag-3	*cln1A1*	Link-3	SUMO	ATag-4
pEG3554	ATag-3	*cln1A2*	Link-3	SUMO	ATag-4
pEG3555	ATag-3	*cln2A1*	Link-3	SUMO	ATag-4
pEG3556	ATag-3	*cln2A2*	Link-3	SUMO	ATag-4
pEG3557	ATag-3	*cln3A1*	Link-3	SUMO	ATag-4
pEG3558	ATag-3	*cln3A2*	Link-3	SUMO	ATag-4
pEG3559	ATag-3	*cln3A3*	Link-3	SUMO	ATag-4
pEG3560	ATag-3	*csegA1*	Link-3	SUMO	ATag-4
pEG3561	ATag-3	*csegA2*	Link-3	SUMO	ATag-4
pEG3562	ATag-3	*csegA3*	Link-3	SUMO	ATag-4

g

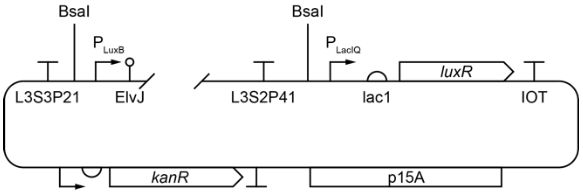

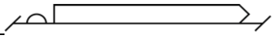

Name		
pEG1128	ME_61	*truD*

h

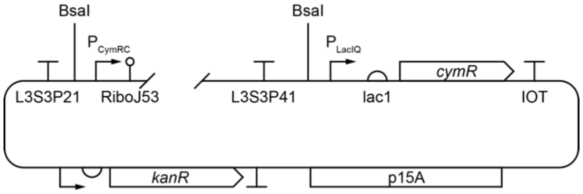

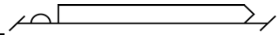

Name		
pEG7172	ME_24	*halM1*
pEG7173	ME_25	*halM2*

i

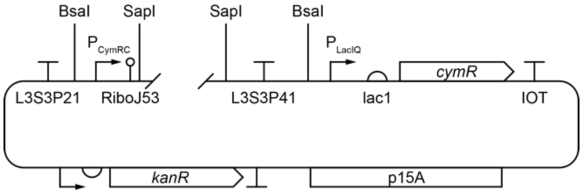

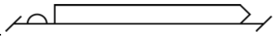

Name		
pEG7034	ME_60	*truD*
pEG7035	ME_1	*albA*
pEG7037	ME_36	*mdnC*
pEG7043	ME_53	*procM*
pEG7048	ME_37	*mibD*
pEG7058	ME_48	*pbtO*
pEG7059	ME_47	*pbtM1*
pEG7060	ME_41	*paaA*
pEG7066	ME_12	*cinX*
pEG7069	ME_30	*lasF*
pEG7071	ME_4	*albsT*
pEG7074	ME_39	*mibO*
pEG7076	ME_32	*ltnM1*
pEG7077	ME_33	*ltnM2*
pEG7078	ME_20	*crnM*
pEG7079	ME_9	*bsjM*
pEG7127	ME_54	*psnB*

pEG7130	ME_5	*amdnC*
pEG7137	ME_42	*padeK*
pEG7138	ME_59	*thcoK*
pEG7139	ME_57	*stspM*
pEG7141	ME_31	*lcnG*
pEG7142	ME_43	*palS*
pEG7143	ME_56	*sgbL*
pEG7144	ME_55	*raxST*
pEG7145	ME_19	*comQ*
pEG7146	ME_26	*kgpF*
pEG7147	ME_58	*tgnB*
pEG7148	ME_46	*patD*
pEG7149	ME_44	*papB*
pEG7160	ME_34	*lynD*
pEG7166	ME_45	*papoK*
pEG7169	ME_23	*epiD*
pEG7171	ME_8	*bamB*

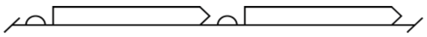

Name				
pEG7047	ME_38	*mibH*	ME_40	*mibS*
pEG7056	ME_51	*plpX*	ME_52	*plpY*
pEG7067	ME_10	*capB*	ME_11	*capC*
pEG7070	ME_2	*albsB*	ME_3	*albsC*
pEG7073	ME_35	*mcbC*	native	*mcbD*
pEG7132	ME_6	*atxB*	ME_7	*atxC*
pEG7133	ME_13	*cln1B*	ME_14	*cln1C*
pEG7134	ME_15	*cln2B*	ME_16	*cln2C*
pEG7135	ME_17	*cln3B*	ME_18	*cln3C*
pEG7136	ME_21	*csegB*	ME_22	*csegC*
pEG7152	ME_49	*pcpX*	ME_50	*pcpY*

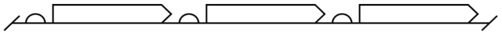

Name						
pEG7068	ME_27	*lasB*	ME_28	*lasC*	ME_29	*lasD*

S1 Figure. Plasmid maps used in this study. a. Architectures of precursor peptide plasmid with MBP. **b.** Architectures of precursor peptide plasmids without SUMO (with ATag-1). **c.** Architectures of precursor peptide plasmids with the initially used RST_N (with Link-1). **d.** Architectures of plasmids with RST_N (with ATag-2 and Link-2). **e.** Architectures of plasmids with RST_N, with flanking BsaI sites added around the operon for optional subcloning. **f.** Architectures of plasmids with RST_C. **g.** Architecture of modifying enzyme plasmid with pLux promoter. **h.** Architectures of modifying enzyme plasmids with pCym promoter. **i.** Architecture of modifying enzyme plasmids with pCym promoter and SapI sites around RBS+gene for optional subcloning.
