# Supplementary material for: Functional expression of diverse post-translational peptide-modifying enzymes in Escherichia coli under uniform expression and purification conditions
Source: PLoS One. 2022 Sep 19;17(9):e0266488. doi: 10.1371/journal.pone.0266488 (PMC9484694; doi:10.1371/journal.pone.0266488)
Supplement: S2 Fig — Each plate represents an individual replicate of Haloduracin A1 and A2 expression, purification, cleavage, and assaying for zone of inhibition. Quadrants are Haloduracin A1 (top right), Haloduracin A2 (top left), Haloduracin A1 and A2 (bottom right), and 50% methanol in water %vol/vol solvent control (bottom left). (PDF) [file pone.0266488.s002.pdf]

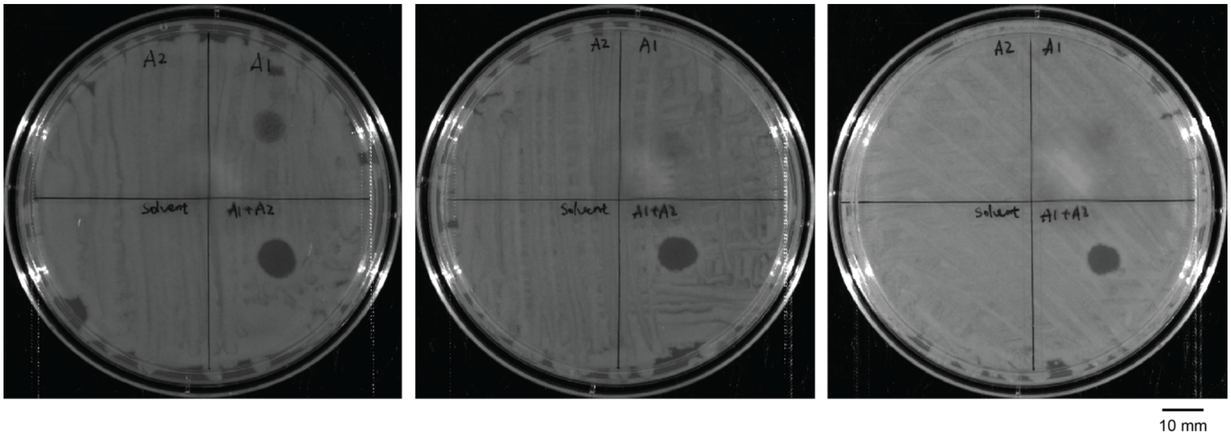

S2 Figure. Haloduracin zones of inhibition. Each plate represents an individual replicate of Haloduracin A1 and A2 expression, purification, cleavage, and assaying for zone of inhibition. Quadrants are Haloduracin A1 (top right), Haloduracin A2 (top left), Haloduracin A1 and A2 (bottom right), and 50% methanol in water %vol/vol solvent control (bottom left).
