# Supplementary material for: Functional expression of diverse post-translational peptide-modifying enzymes in Escherichia coli under uniform expression and purification conditions
Source: PLoS One. 2022 Sep 19;17(9):e0266488. doi: 10.1371/journal.pone.0266488 (PMC9484694; doi:10.1371/journal.pone.0266488)
Supplement: S3 Fig — Spectra of the modified peaks are shown on the right. Chromatograms are grouped by peptide plasmid number and modifying enzyme plasmid number (numbers are shown without the preceeding “pEG”). Both expression medias are shown, with one replicate chosen for each and the extract number listed for the chosen replicate. Y-axis for all plots is Intensity in arbitrary units (x107 for extracts analyzed with QQQ and x105 for extracts analyzed with QTOF). From left to right, plots represent: TIC (black trace), ECC of unmodified peptide (green trace), ECC of partially modified peptide(s) (blue if passes peak thresholds or yellow otherwise), ECC of properly modified peptide (red trace), and mass spectrum. In ECC plots, peak fit is drawn as a black line. If a peak passes all thresholds, the peak area (x105 for extracts analyzed with QQQ and x103 for extracts analyzed with QTOF) is provided as a colored number in the top right corner of the plot. Partially modified peaks can have multiple numbers listed, one for each modification state (these are ordered ascending by magnitude top-to-bottom, left-to-right). All extracts are analyzed with QQQ unless there is an asterisk (*) next to the extract number, which denotes analyzed with QTOF. Peak lists and raw data are available for more detailed analysis on Zenodo. (PDF) [file pone.0266488.s003.pdf]

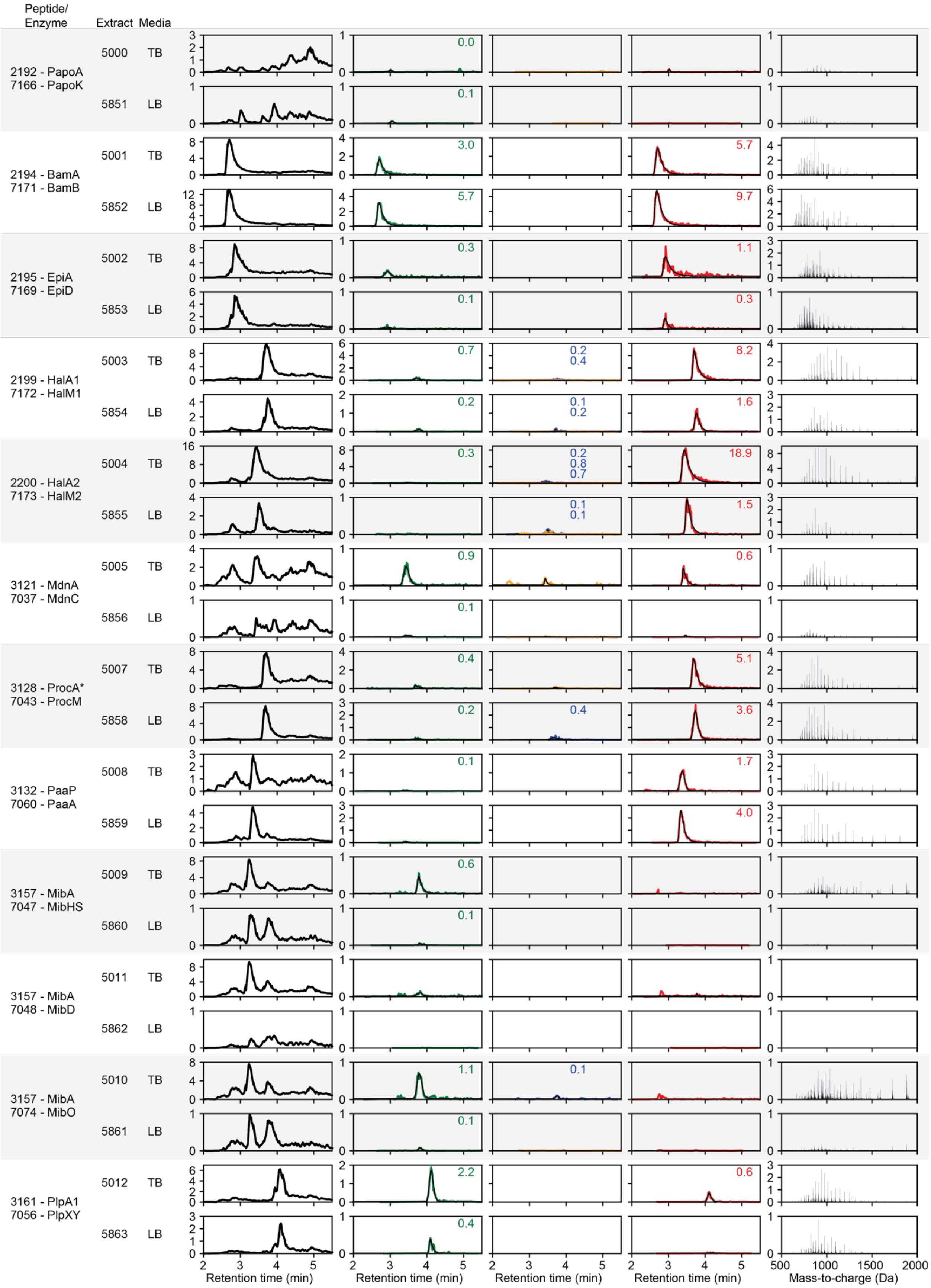

Peptide/ Enzyme
Extract
Media
2192 - PapoA
7166 - PapoK
5000 TB
5851 LB
2194 - BamA
7171 - BamB
5001 TB
5852 LB
2195 - EpiA
7169 - EpiD
5002 TB
5853 LB
2199 - HalA1
7172 - HalM1
5003 TB
5854 LB
2200 - HalA2
7173 - HalM2
5004 TB
5855 LB
3121 - MdnA
7037 - MdnC
5005 TB
5856 LB
3128 - ProcA*
7043 - ProcM
5007 TB
5858 LB
3132 - PaaP
7060 - PaaA
5008 TB
5859 LB
3157 - MibA
7047 - MibHS
5009 TB
5860 LB
3157 - MibA
7048 - MibD
5011 TB
5862 LB
3157 - MibA
7074 - MibO
5010 TB
5861 LB
3161 - PlpA1
7056 - PlpXY
5012 TB
5863 LB
Retention time (min)
Mass-to-charge (Da)

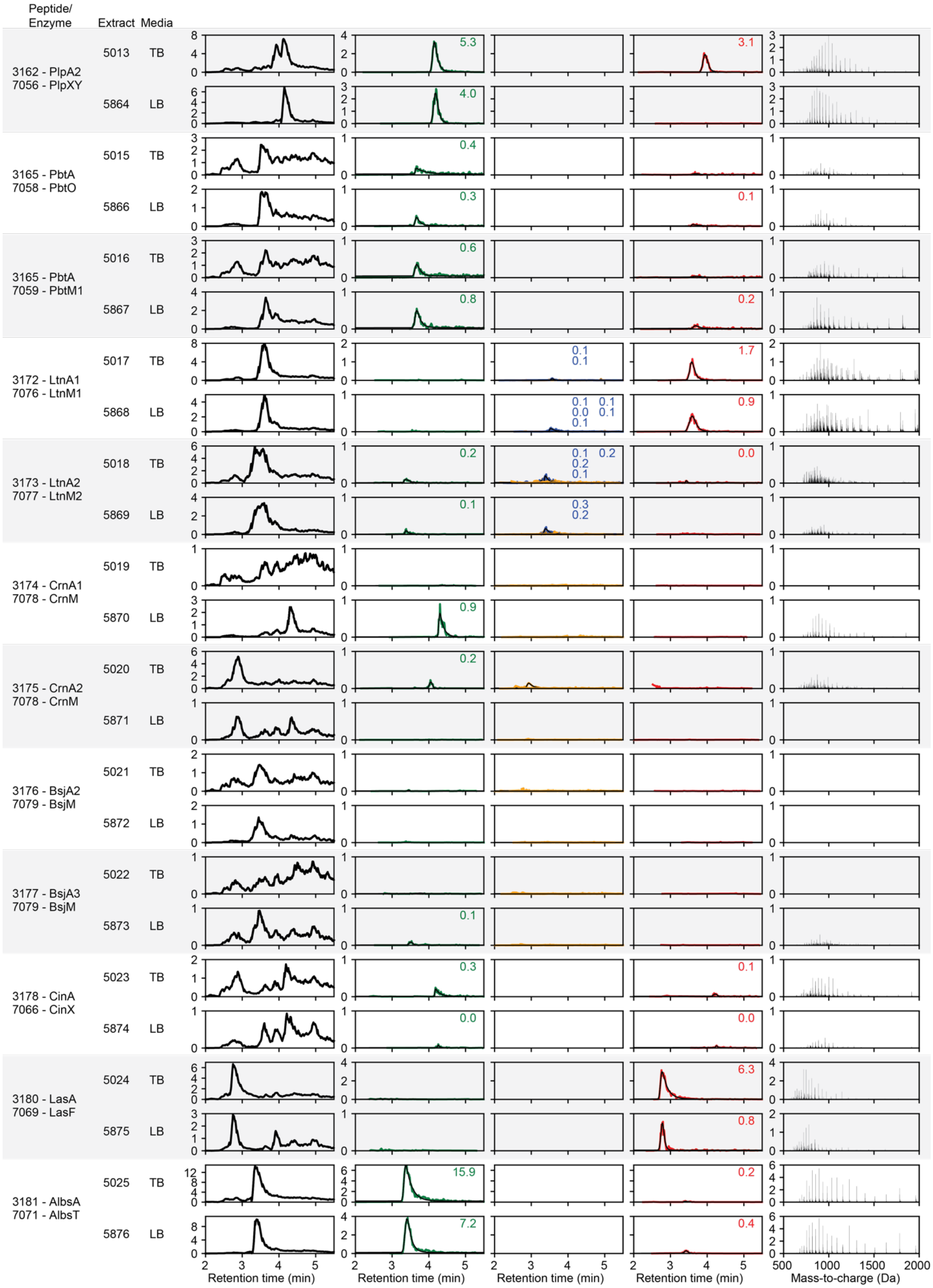
Peptide/ Enzyme
Extract
Media
3162 - PlpA2
7056 - PlpXY
5013
TB
5864
LB
5.3
3.1
4.0
3165 - PbtA
7058 - PbtO
5015
TB
5866
LB
0.4
0.3
0.1
3165 - PbtA
7059 - PbtM1
5016
TB
5867
LB
0.6
0.8
0.2
3172 - LtnA1
7076 - LtnM1
5017
TB
5868
LB
0.1
0.1
1.7
0.1 0.1
0.0 0.1
0.1
0.9
3173 - LtnA2
7077 - LtnM2
5018
TB
5869
LB
0.2
0.1 0.2
0.2
0.1
0.0
0.1
0.3
0.2
3174 - CrnA1
7078 - CrnM
5019
TB
5870
LB
0.9
3175 - CrnA2
7078 - CrnM
5020
TB
5871
LB
0.2
3176 - BsjA2
7079 - BsjM
5021
TB
5872
LB
3177 - BsjA3
7079 - BsjM
5022
TB
5873
LB
0.1
3178 - CinA
7066 - CinX
5023
TB
5874
LB
0.3
0.1
0.0
0.0
3180 - LasA
7069 - LasF
5024
TB
5875
LB
6.3
0.8
3181 - AlbsA
7071 - AlbsT
5025
TB
5876
LB
15.9
0.2
7.2
0.4
Retention time (min)
Retention time (min)
Retention time (min)
Retention time (min)
Mass-to-charge (Da)

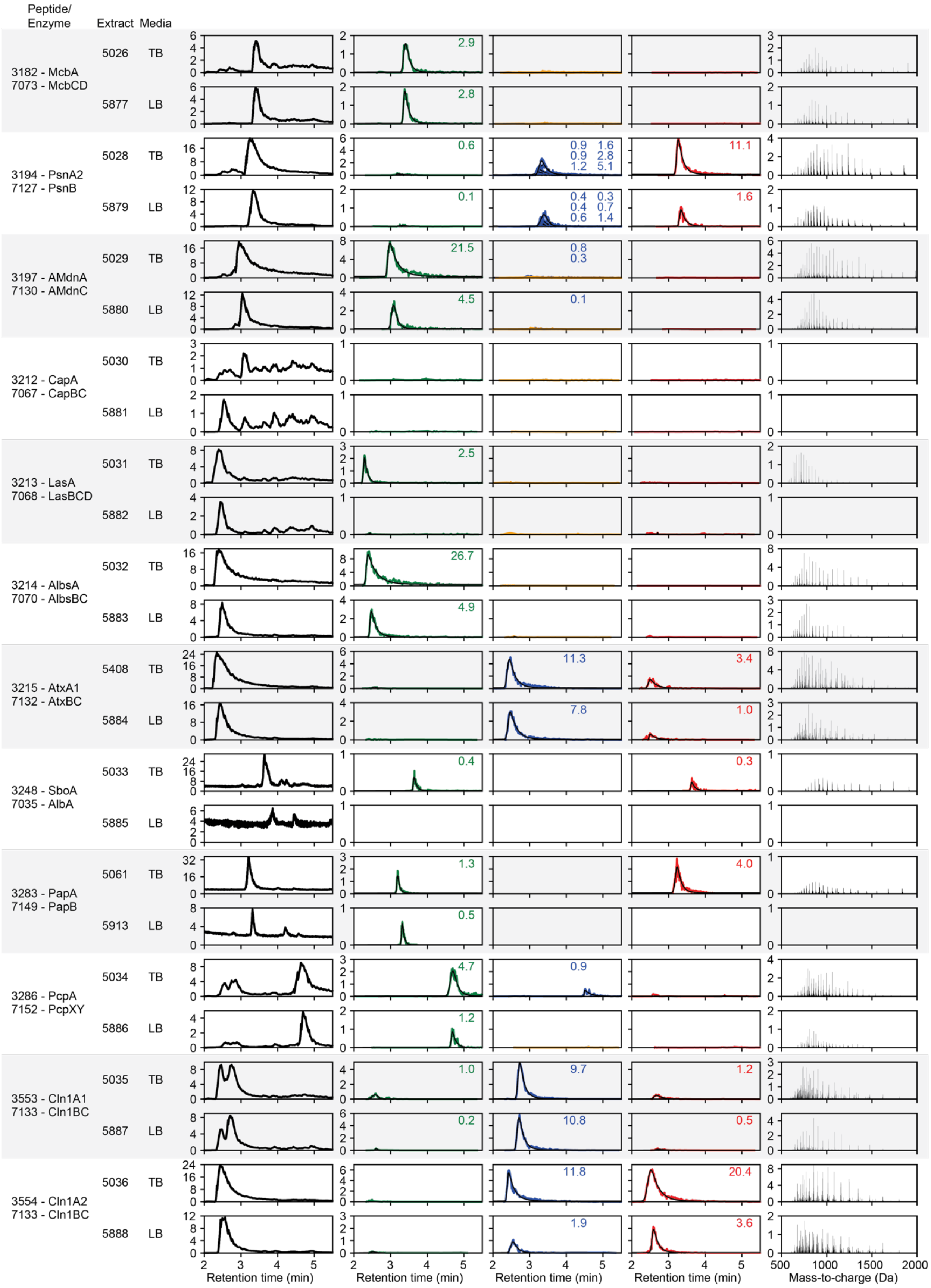
Peptide/ Enzyme
Extract
Media
3182 - McbA
7073 - McbCD
5026 TB 2.9
5877 LB 2.8
3194 - PsnA2
7127 - PsnB
5028 TB 0.6 0.9 1.6 0.9 2.8 1.2 5.1 11.1
5879 LB 0.1 0.4 0.3 0.4 0.7 0.6 1.4 1.6
3197 - AMdnA
7130 - AMdnC
5029 TB 21.5 0.8 0.3
5880 LB 4.5 0.1
3212 - CapA
7067 - CapBC
5030 TB
5881 LB
3213 - LasA
7068 - LasBCD
5031 TB 2.5
5882 LB
3214 - AlbsA
7070 - AlbsBC
5032 TB 26.7
5883 LB 4.9
3215 - AtxA1
7132 - AtxBC
5408 TB 11.3 3.4
5884 LB 7.8 1.0
3248 - SboA
7035 - AlbA
5033 TB 0.4 0.3
5885 LB
3283 - PapA
7149 - PapB
5061 TB 1.3 4.0
5913 LB 0.5
3286 - PcpA
7152 - PcpXY
5034 TB 4.7 0.9
5886 LB 1.2
3553 - Cln1A1
7133 - Cln1BC
5035 TB 1.0 9.7 1.2
5887 LB 0.2 10.8 0.5
3554 - Cln1A2
7133 - Cln1BC
5036 TB 11.8 20.4
5888 LB 1.9 3.6
Retention time (min)
Retention time (min)
Retention time (min)
Retention time (min)
Mass-to-charge (Da)

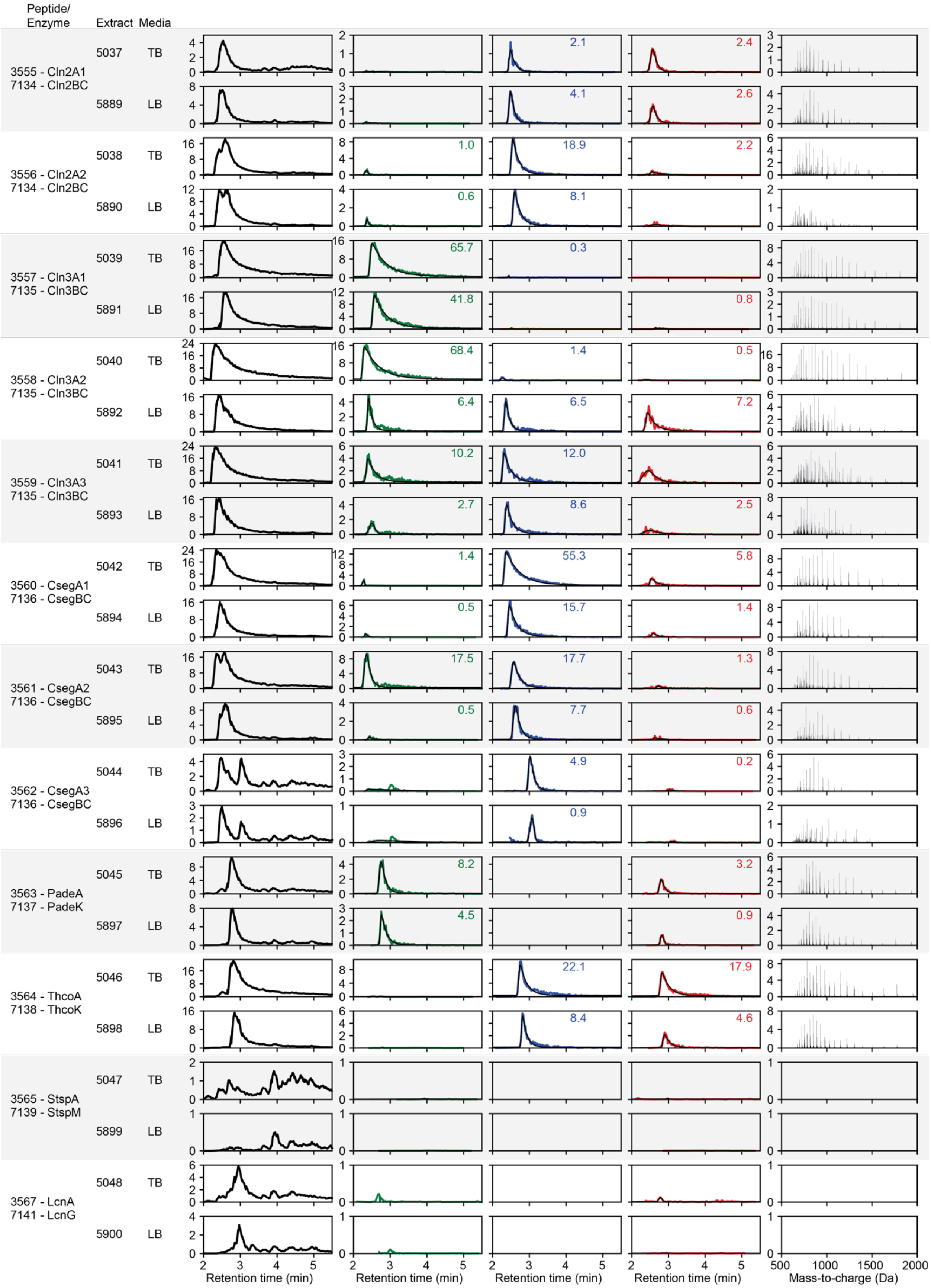
Peptide/ Enzyme
Extract
Media
3555 - Cln2A1
7134 - Cln2BC
5037 TB
2.1
2.4
5889 LB
4.1
2.6
3556 - Cln2A2
7134 - Cln2BC
5038 TB
1.0
18.9
2.2
5890 LB
0.6
8.1
3557 - Cln3A1
7135 - Cln3BC
5039 TB
65.7
0.3
5891 LB
41.8
0.8
3558 - Cln3A2
7135 - Cln3BC
5040 TB
68.4
1.4
0.5
5892 LB
6.4
6.5
7.2
3559 - Cln3A3
7135 - Cln3BC
5041 TB
10.2
12.0
5893 LB
2.7
8.6
2.5
3560 - CsegA1
7136 - CsegBC
5042 TB
1.4
55.3
5.8
5894 LB
0.5
15.7
1.4
3561 - CsegA2
7136 - CsegBC
5043 TB
17.5
17.7
1.3
5895 LB
0.5
7.7
0.6
3562 - CsegA3
7136 - CsegBC
5044 TB
4.9
0.2
5896 LB
0.9
3563 - PadeA
7137 - PadeK
5045 TB
8.2
3.2
5897 LB
4.5
0.9
3564 - ThcoA
7138 - ThcoK
5046 TB
22.1
17.9
5898 LB
8.4
4.6
3565 - StspA
7139 - StspM
5047 TB
5899 LB
3567 - LcnA
7141 - LcnG
5048 TB
5900 LB
Retention time (min)
Retention time (min)
Retention time (min)
Retention time (min)
Mass-to-charge (Da)

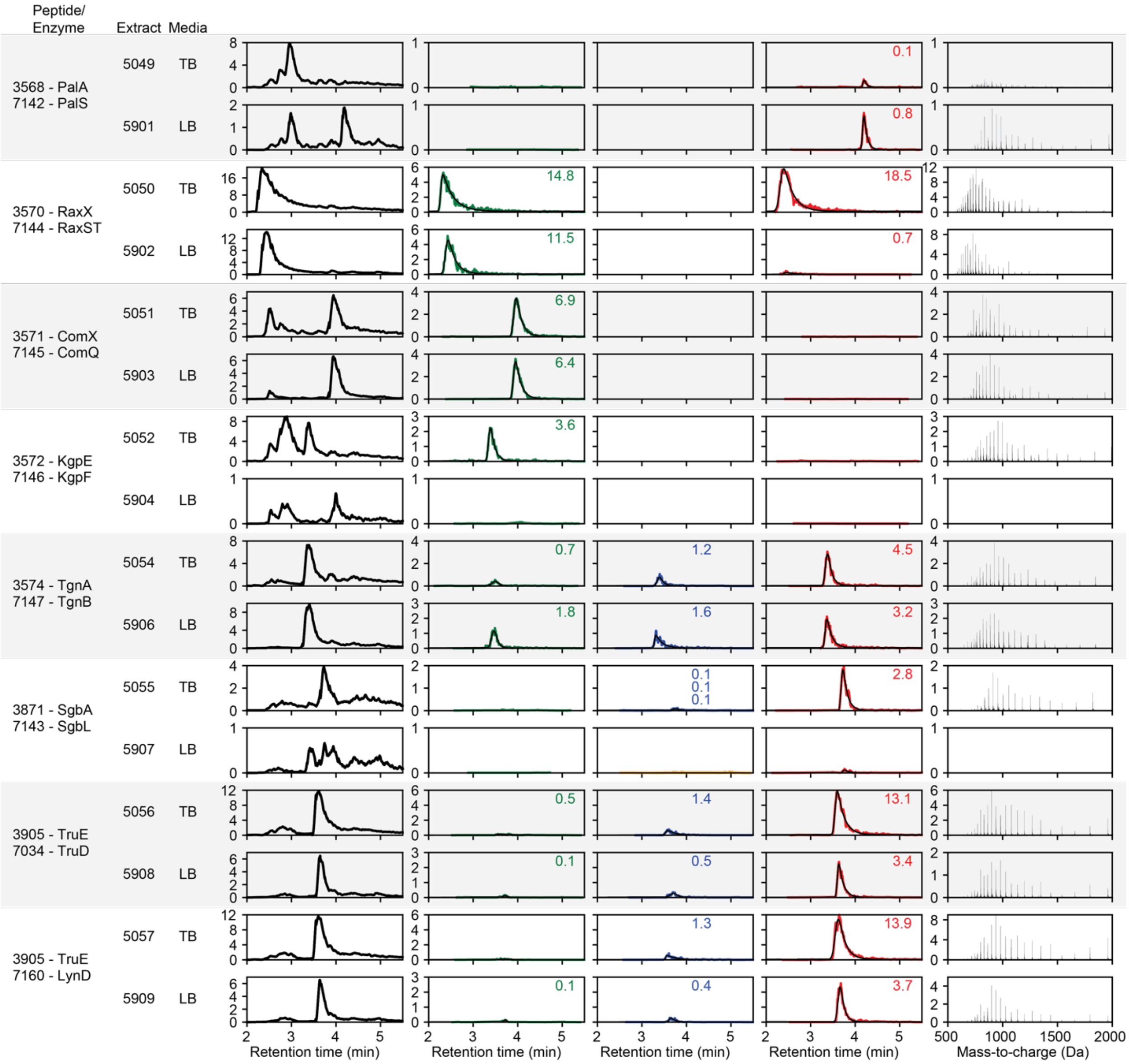

S3 Figure. Chromatograms of high-throughput peptide expression/modification samples. Spectra of the modified peaks are shown on the right. Chromatograms are grouped by peptide plasmid number and modifying enzyme plasmid number (numbers are shown without the preceeding "pEG"). Both expression medias are shown, with one replicate chosen for each and the extract number listed for the chosen replicate. Y-axis for all plots is Intensity in arbitrary units (x10^7 for extracts analyzed with QQQ and x10^5 for extracts analyzed with QTOF). From left to right, plots represent: TIC (black trace), ECC of unmodified peptide (green trace), ECC of partially modified peptide(s) (blue if passes peak thresholds or yellow otherwise), ECC of properly modified peptide (red trace), and mass spectrum. In ECC plots, peak fit is drawn as a black line. If a peak passes all thresholds, the peak area (x10^5 for extracts analyzed with QQQ and x10^3 for extracts analyzed with QTOF) is provided as a colored number in the top right corner of the plot. Partially modified peaks can have multiple numbers listed, one for each modification state (these are ordered ascending by magnitude top-to-bottom, left-to-right). All extracts are analyzed with QQQ unless there is an asterisk (*) next to the extract number, which denotes analyzed with QTOF. Peak lists and raw data are available for more detailed analysis on Zenodo.
