# Supplementary material for: Functional expression of diverse post-translational peptide-modifying enzymes in Escherichia coli under uniform expression and purification conditions
Source: PLoS One. 2022 Sep 19;17(9):e0266488. doi: 10.1371/journal.pone.0266488 (PMC9484694; doi:10.1371/journal.pone.0266488)
Supplement: S4 Fig — Peptide plasmid number and gene name, modifying enzyme plasmid number and gene name, and replicate extract numbers are listed alongside fraction modified in TB (dark grey) and LB (light grey) medias. Dashed line demarcates 50% modification (half of peptide modified). Three replicates are shown for each peptide/enzyme/media combination and represent independent expressions that were purified and assayed. Bar is shown at the replicate mean. (PDF) [file pone.0266488.s004.pdf]

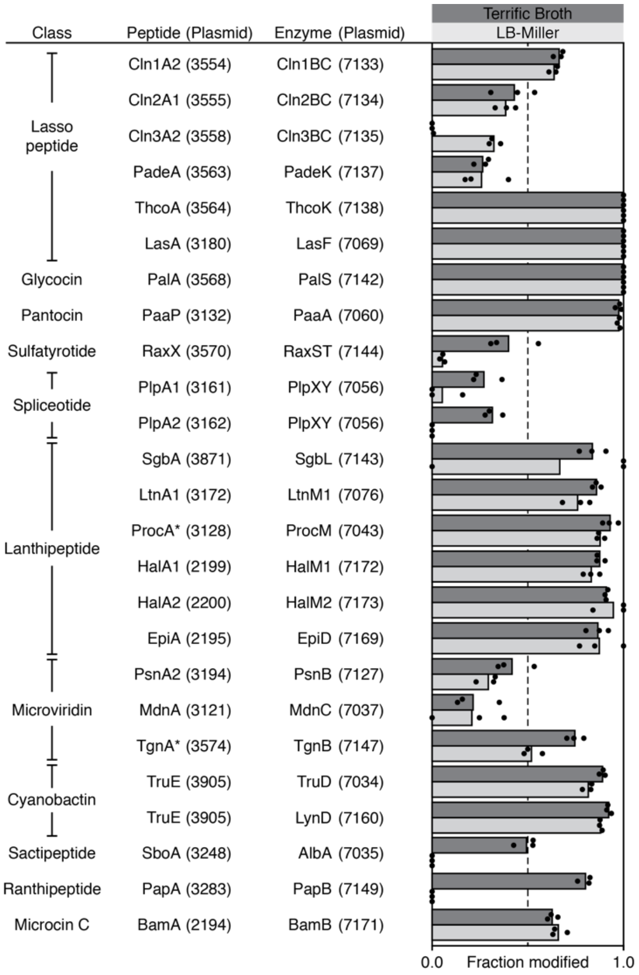

S4 Figure. Bar chart of successful peptide/modifying enzyme combinations. Peptide plasmid number and gene name, modifying enzyme plasmid number and gene name, and replicate extract numbers are listed alongside fraction modified in TB (dark grey) and LB (light grey) medias. Dashed line demarcates 50% modification (half of peptide modified). Three replicates are shown for each peptide/enzyme/media combination and represent independent expressions that were purified and assayed. Bar is shown at the replicate mean.
