# Supplementary material for: Functional expression of diverse post-translational peptide-modifying enzymes in Escherichia coli under uniform expression and purification conditions
Source: PLoS One. 2022 Sep 19;17(9):e0266488. doi: 10.1371/journal.pone.0266488 (PMC9484694; doi:10.1371/journal.pone.0266488)
Supplement: S5 Fig — Spectra of small mass-shift modification catalyzed by AlbA. Ion distribution for [M+4H]4+ is shown, with unmodified, modified, and observed m/z’s listed for the monoisotopic mass, which is labeled with an arrow. Data for cleaved peptide was collected once. (PDF) [file pone.0266488.s005.pdf]

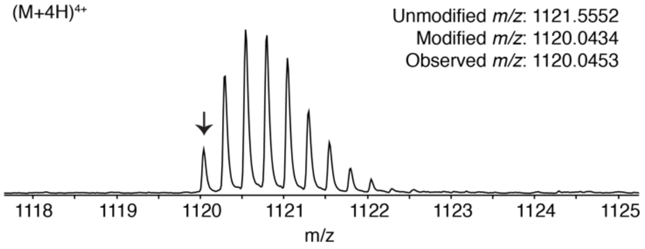

S5 Figure. SboA+AlbA post-cleavage mass spectrum. Spectra of small mass-shift modification catalyzed by AlbA. Ion distribution for $[M+4H]^{4+}$ is shown, with unmodified, modified, and observed *m/z*'s listed for the monoisotopic mass, which is labeled with an arrow. Data for cleaved peptide was collected once.
