# Supplementary material for: Functional expression of diverse post-translational peptide-modifying enzymes in Escherichia coli under uniform expression and purification conditions
Source: PLoS One. 2022 Sep 19;17(9):e0266488. doi: 10.1371/journal.pone.0266488 (PMC9484694; doi:10.1371/journal.pone.0266488)
Supplement: S6 Fig — Tree was generated using the producing organisms listed in S2 Table and the NCBI Common Tree application. Species from which functional enzymes were sourced are black, nonfunctional are red. (PDF) [file pone.0266488.s006.pdf]

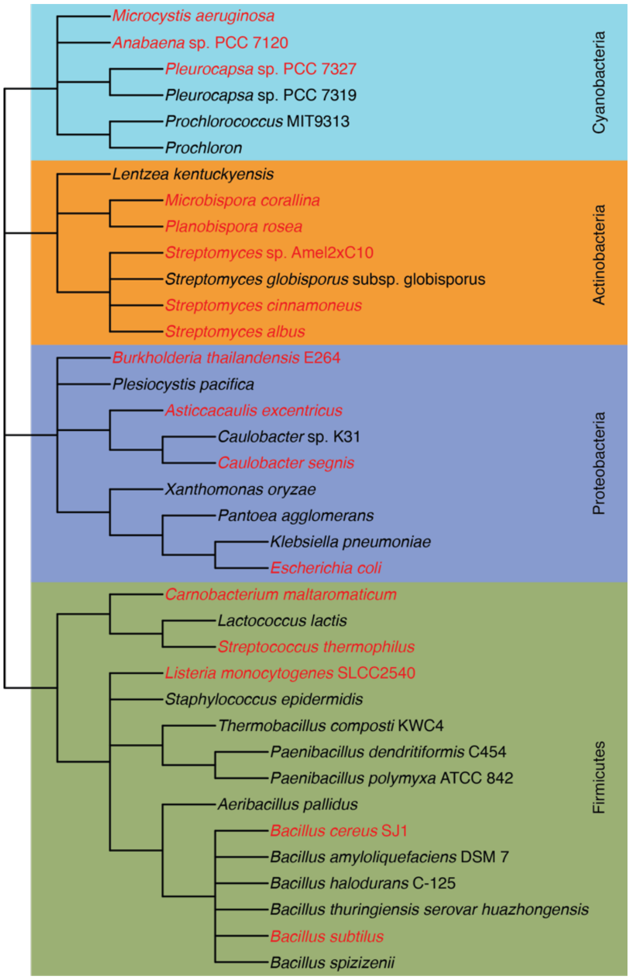

S6 Figure. **Phylogenetic tree species from which we mine enzymes.** Tree was generated using the producing organisms listed in Supplementary Table 2 and the NCBI Common Tree application. Species from which functional enzymes were sourced are black, nonfunctional are red.
