# Supplementary material for: Functional expression of diverse post-translational peptide-modifying enzymes in Escherichia coli under uniform expression and purification conditions
Source: PLoS One. 2022 Sep 19;17(9):e0266488. doi: 10.1371/journal.pone.0266488 (PMC9484694; doi:10.1371/journal.pone.0266488)
Supplement: S1 Note — (PDF) [file pone.0266488.s007.pdf]

S1 Note. LC-MS/MS Data Legend

[ion charge state] Expected versus observed masses<br>↓Arrow - parent ion used in fragmentation<br>Spectrum of unfragmented peptide	**Notes**<br>Notes about the structure or MS/MS data
Predicted structure, with y/b ions labeled and mapped modifications shown<br><br>MS/MS spectra were obtained using four different collision energies (30, 45, 60, or 75), with the most informative spectra selected for display. Within each spectra, peaks that match expected fragment masses are labeled with their y/b-ion number and charge state (only proton adducts are considered). Peak lists are provided in each spectrum that lists predicted and observed *m/z* and error (ppm).<br><br>MS/MS data was collected once for each peptide+enzyme combination.	
