# Supplementary figures and images for: Functional expression of diverse post-translational peptide-modifying enzymes in Escherichia coli under uniform expression and purification conditions
Source: PLoS One. 2022 Sep 19;17(9):e0266488. doi: 10.1371/journal.pone.0266488 (PMC9484694; doi:10.1371/journal.pone.0266488)

## S2 Note. HalA1+HalM1 MS/MS Spectra

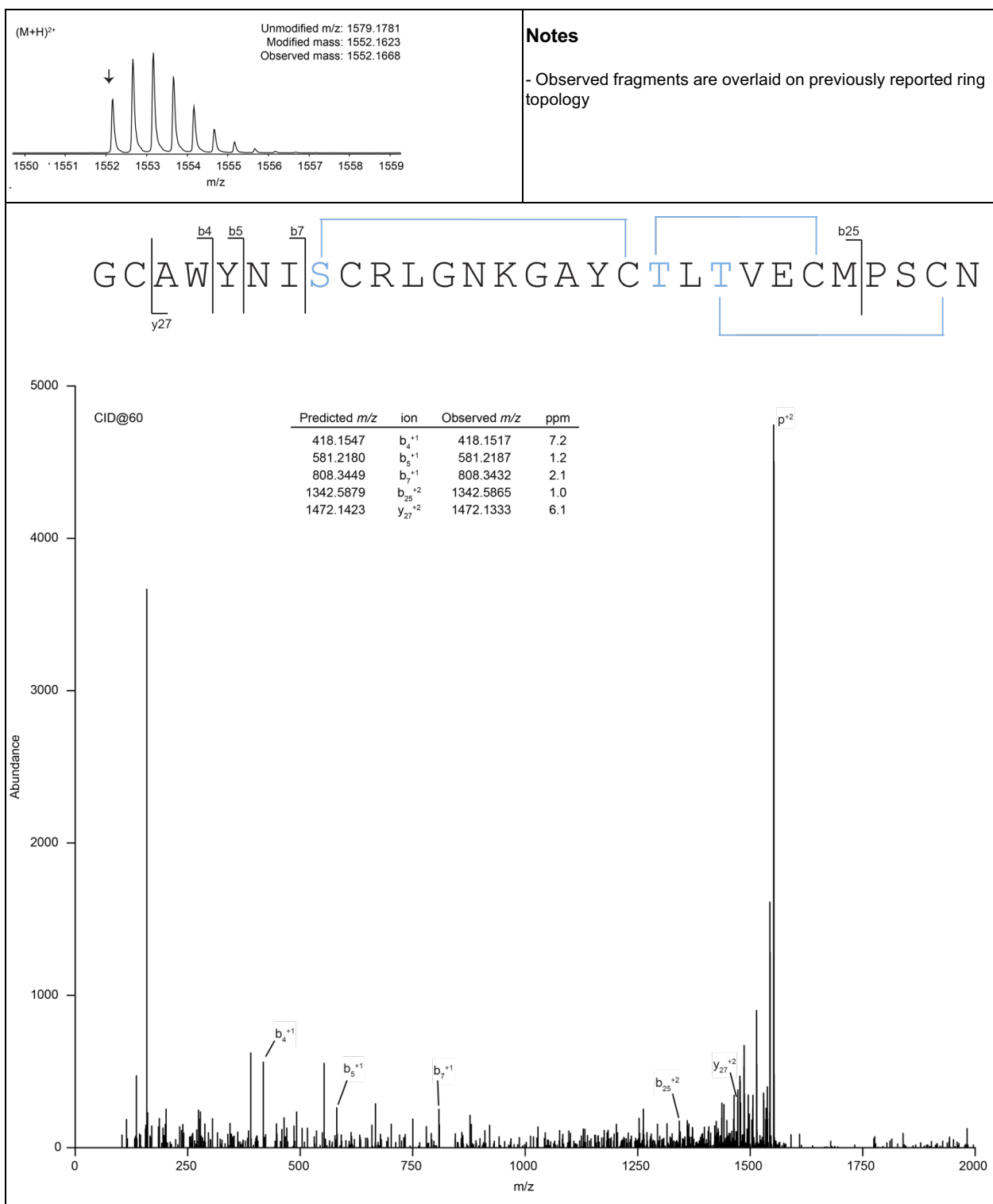

Supplement: S2 Note — (PDF) [file pone.0266488.s008.pdf]

### S3 Note: HalA2+HalM2 MS/MS Spectra

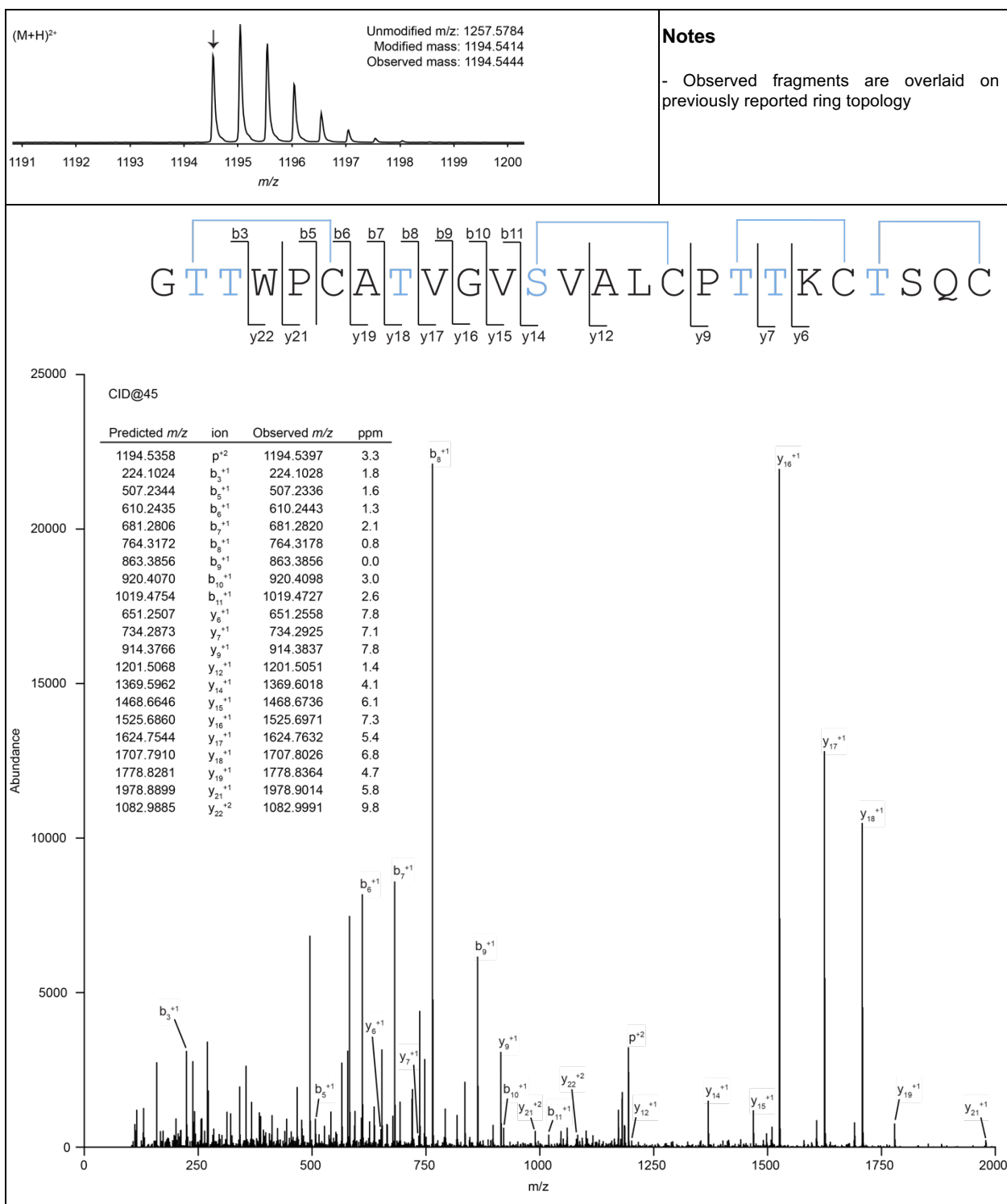

Supplement: S3 Note — (PDF) [file pone.0266488.s009.pdf]

# S4 Note: PsnA2+PsnB MS/MS Spectra

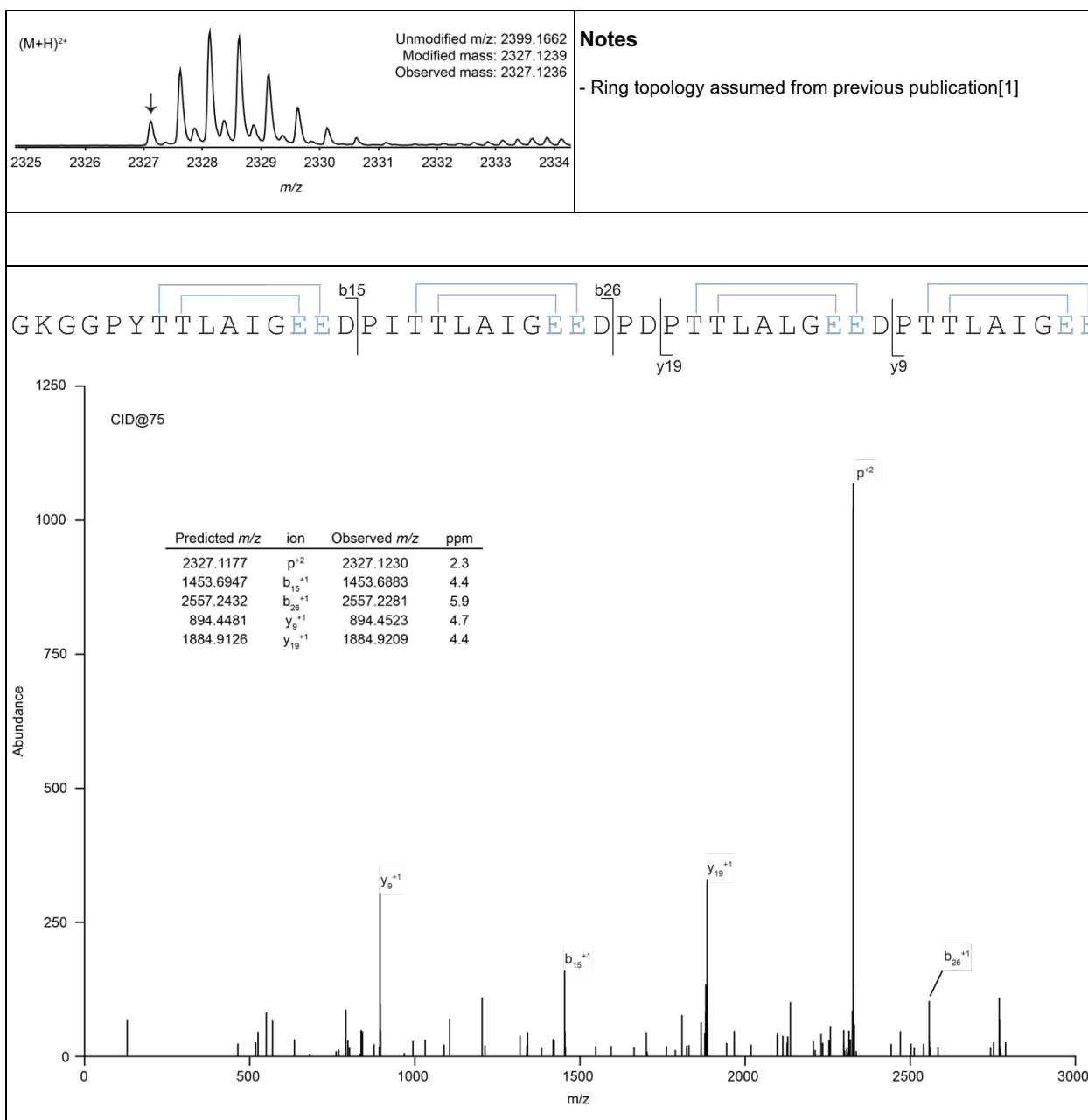

Supplement: S4 Note — (PDF) [file pone.0266488.s010.pdf]

# S5 Note: PapA+PapB MS/MS spectra

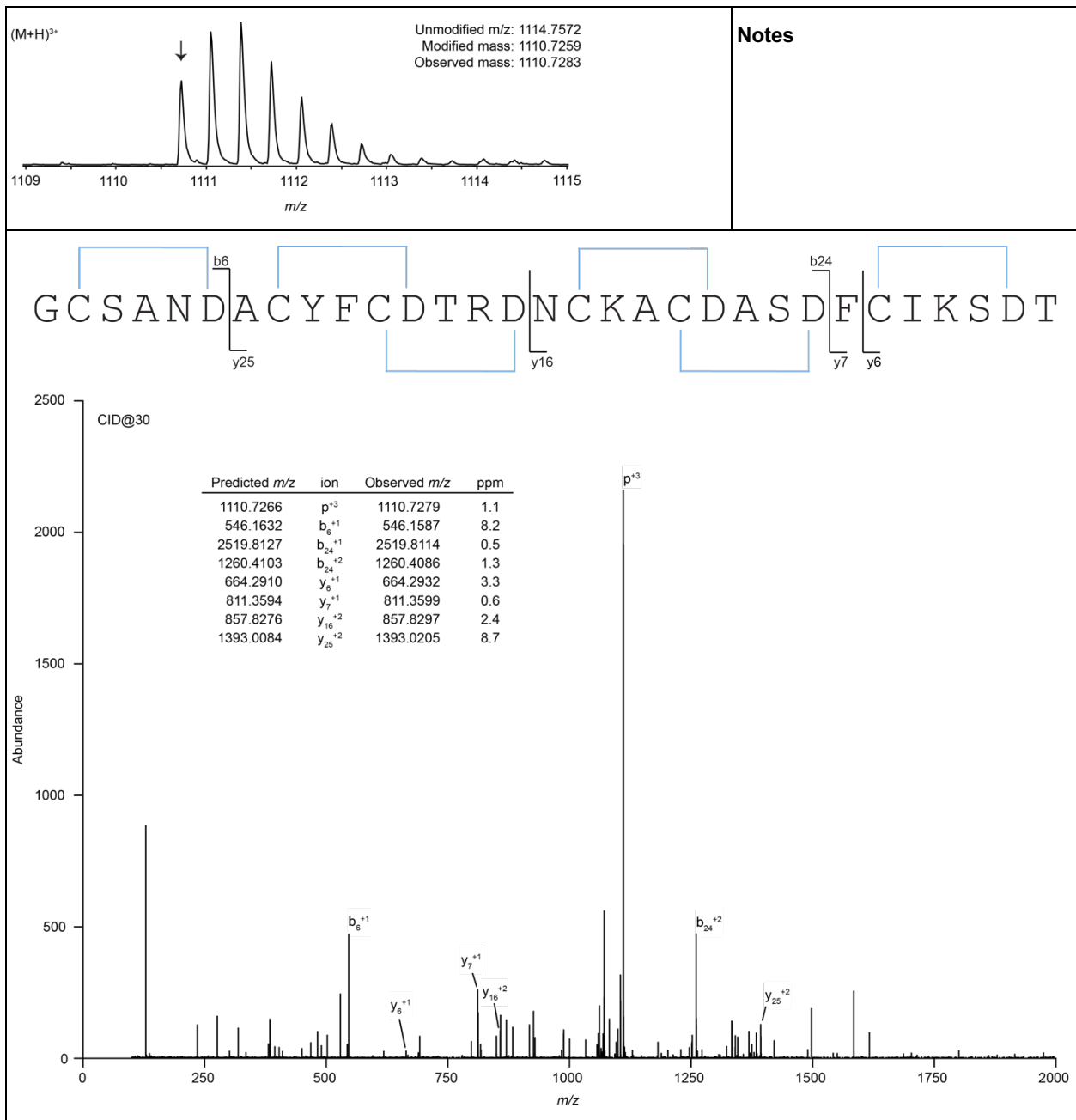

Supplement: S5 Note — (PDF) [file pone.0266488.s011.pdf]
