# Supplementary material for: Functional expression of diverse post-translational peptide-modifying enzymes in Escherichia coli under uniform expression and purification conditions
Source: PLoS One. 2022 Sep 19;17(9):e0266488. doi: 10.1371/journal.pone.0266488 (PMC9484694; doi:10.1371/journal.pone.0266488)
Supplement: S1 Table — (PDF) [file pone.0266488.s012.pdf]

S1 Table. Flask-expressed peptide mass spectra masses

Extract	Tag	Peptide[a]	Enzyme[a]	Spectra Masses					Observed Mass[b]	Stdev[c]	Predicted Mass	Figure
				1	2	3	4	5				
132	MBP	TruE (3017)	TruD (1128)	833.96	847.84	862.24	877.12	892.44	50,811.8	18.3	50,815.0	1c
192	HIS_6	MdnA (3045)	-	953.48	1,072.60	1,225.70	1,429.80	1,715.56	8,572.7	3	8,575.2	2
193	HIS_6	BmbC (3046)	-	-	-	-	-	-	-	-	7,688.2	2
194	HIS_6	StrA (3047)	-	-	-	-	-	-	-	-	6,194.8	2
195	HIS_6	PqqA (3048)	-	-	-	-	-	-	-	-	5,644.3	2
196	HIS_6	SboA (3049)	-	-	-	-	-	-	-	-	7,203.0	2
198	HIS_6	TfxA (3051)	-	690.90	759.90	844.20	949.70	1,085.10	7,588.9[d]	3	7,286.2	2
199	HIS_6	ProcA1.7 (3052)	-	948.15	1,027.16	1,120.36	1,232.35	1,369.17	12,313.3	4	12,318.7	2
200	HIS_6	TbtA (3053)	-	-	-	-	-	-	-	-	7,942.7	2
202	HIS_6	Pgm2 (3055)	-	781.80	879.42	1,004.94	1,172.17	1,406.59	7,027.4	3	7,029.5	2
216	RST_N[e]	MdnA (3058)	-	902.34	945.26	992.49	1,044.64	1,102.63	19,829.3	7	19,828.7	2
217	RST_N[e]	SboA (3059)	-	721.50	759.47	801.51	849.42	901.71	14,412.8[d]	6	18,456.6	2
218	RST_N[e]	PqqA (3060)	-	805.64	845.86	890.32	939.65	994.96	16,896.8	6	16,897.8	2
219	RST_N[e]	StrA (3061)	-	1,247.30	1,343.23	1,455.11	1,587.32	1,745.95	17,449.0	4	17,448.3	2
220	RST_N[e]	BmbC (3062)	-	1,115.21	1,184.77	1,263.68	1,353.89	1,458.04	18,940.7	5	18,941.7	2
221	RST_N[e]	TfxA (3063)	-	675.70	705.10	737.10	772.20	810.70	16,193.9[f]	7	18,539.7	2
222	RST_N[e]	ProcA1.7 (3064)	-	842.76	873.97	907.71	943.94	983.13	23,571.5	8	23,572.2	2
223	RST_N[e]	TbtA (3065)	-	1,372.23	1,477.59	1,600.70	1,746.16	1,920.77	19,196.6	4	19,196.2	2
225	RST_N[e]	Pgm2 (3067)	-	1,306.91	1,407.43	1,524.57	1,663.13	1,829.52	18,283.5	4	18,283.0	2
230	RST_N[e]	TruE (3057)	TruD (1128)	941.8	988.8	1040.8	1098.6	1163.1	19,756.2	6	19,753.9	1c

a. Plasmid number in parenthesis is shown without “pEG” prefix
b. Observed mass was calculated using technique described in the methods.
c. Sandard deviation was calculated as the maximum of: 1. the standard deviation of the parent masses calculated from each of the multiply charged ions, or 2. the maximal charge state (lowest *m/z*) observed multiplied by the standard deviation of the mass spectrometer used (Methods).
d. Observed mass for SUMO-SboA is a product of cleavage at MK/KAVIVENK in the leader peptide.
e. RST_N used was the initial version using Link-1
f. Observed masses for SUMO-TfxA are the result of three different leader peptide cleavages: KGSI/KATFKA, KGSIKAT/FKA, or KGSIKATF/KA.
