# Supplementary material for: Functional expression of diverse post-translational peptide-modifying enzymes in Escherichia coli under uniform expression and purification conditions
Source: PLoS One. 2022 Sep 19;17(9):e0266488. doi: 10.1371/journal.pone.0266488 (PMC9484694; doi:10.1371/journal.pone.0266488)
Supplement: S2 Table — (PDF) [file pone.0266488.s013.pdf]

S2 Table. Pathways investigated in this study

RiPP Class	Cluster Name	Molecule Name(s)	Producing organism	Biological Activity	Previous Expression Conditions	Ref
Lasso-peptide	Las	lassomycin	*Lentzea kentuckyensis*	Antibiotic	Native host	[2]
	Cap	capistruin	*Burkholderia thailandensis* E264	Antibiotic	Full Cluster in *E. coli*	[3]
	Albs[a]	albusnodin	*Streptomyces albus*	Unknown	Full Cluster in *S. lividans*	[4]
	Atx	astexin 1-3	*Asticcacaulis excentricus*	Unknown	Selected genes in *E. coli*	[5]
	Cln	caulonodin I-VII	*Caulobacter* sp. K31	Unknown	Selected genes in *E. coli*	[6]
	Cseg	caulosegnins I-III	*Caulobacter segnis*	Unknown	Selected genes in *E. coli*	[7]
	Pade	Paeninodin	*Paenibacillus dendritiformis* C454	Unknown	Selected genes in *E. coli*	[8]
	Thco	unnamed	*Thermobacillus composti* KWC4	Unknown	Selected genes in *E. coli*	[9]
	Papo	unnamed	*Paenibacillus polymyxa* CR1	Unknown	Selected genes in *E. coli*	[8]
	Stsp	unnamed	*Streptomyces* sp. Amel2xC10	Unknown	Full cluster in *E. coli*, and *in vitro*	[10]
Glycocin	Lcn	listeriocytocin	*Listeria monocytogenes* SLCC2540	Unknown	Selected genes in E. coli	[11]
	Pal	pallidocin	*Aeribacillus pallidus* 8	Antibiotic	Selected genes in *E. coli*	[12]
Microcin C	Bam	unnamed	*Bacillus amyloliquefaciens* DSM7	Antibiotic	Selected genes in E. coli	[13]
ComX	Com	ComX	*Bacillus subtilus*	quorum sensing	Selected genes in *E. coli*	[14]
Pantocin	Paa	pantocin	*Pantoea agglomerans*	Antibiotic	Selected genes in *E. coli*	[15, 16]
Sulfa-tyrotide	Rax	RaxX	*Xanthomonas oryzae*	Plant signalling	Selected genes in *E. coli*	[17, 18]
Splice-otide	Plp	unnamed	*Pleurocapsa* sp. PCC7319	Unknown	Selected genes in *E. coli*	[19]
	Pcp	unnamed	*Pleurocapsa* sp. PCC7327	Unknown	Selected genes in *E. coli*	[19]
Lanthi-peptide	Crn	carnolysin A1' carnolysin A2'	*Carnobacterium maltaromaticum* C2	Antibiotic	Selected genes in *E. coli*	[20]
	Sgb	unnamed	*S. globisporus* subsp. globisporus NRRL B2293	Unknown	Selected genes in *E. coli*	[21]
	Bsj	bicereucins	*Bacillus cereus* SJ1	Antibiotic	Selected genes in *E. coli*	[22]
	Ltn	lacticin S<br>lacticin 3147	*Lactococcus lactis*	Antibiotic	Selected genes in *E. coli*	[23]
	Proc	prochlorosins	*Prochlorococcus* MIT9313	Unknown	Selected genes in *E. coli*	[24]
	Mcb	microcin B17	*Escherichia coli*	Antibiotic	Full cluster in *E. coli*	[25]
	Mib	micro-bisporicin	*Microbispora corallina*	Antibiotic	Full cluster in *Nonomuraea sp.* ATCC 39727, Selected genes in *E. coli*	[26]

	Cin	cinnamycin	*Streptomyces cinnamoneus cinnamoneus* DSM 40005	Antibiotic	Selected genes in *E. coli*	[27]
	Hal	haloduracin A1<br>haloduracin A2	*Bacillus halodurans* C-125	Antibiotic	Selected genes in *E. coli*	[28, 29]
	Epi	epidermin	*Staphylococcus epidermidis*	Antibiotic	Selected genes in *E. coli*	[30, 31]
Micro-viridin	AMdn	unnamed	*Anabaena* sp. PCC7120	Unknown	*In vitro*	[32]
	Psn	plesiocin	*Plesiocystis pacifica*	protease inhibitor	*In vitro*	[1]
	Mdn	microviridin L	*Microcystis aeruginosa* NIES843	protease inhibitor	Full cluster in *E. coli*	[33]
	Tgn	unnamed	*Bacillus thuringiensis serovar huazhongensis* BGSC 4BD	Unknown	Selected genes in *E. coli*	[34]
Cyano-bactin	Tru	trunkamide<br>patellins	*Prochloron* spp.	Unknown	Full cluster in *E. coli*	[35]
	Lyn	unnamed	*Prochloron* spp.	Unknown	Selected genes in *E. coli*	[36]
	Kgp	kawaguchi-peptin	*Microcystis aeruginosa* NIES-88	Unknown	Full cluster in *E. coli*	[37]
Thio-peptide	Pbt	GE2270	*Planobispora rosea*	Antibiotic	Full cluster in *Nonomuraea sp.* ATCC 39727	[38]
Sacti-peptide	Alb/Sbo	subtilosin A	*Bacillus subtilis* subsp. spizizenii	Antibiotic	Selected genes in *E. coli*	[39]
	Pap	freyrasin	*Paenibacillus polymyxa* ATCC 842	Antibiotic	Selected genes in *E. coli*	[40]
