# Supplementary material for: Functional expression of diverse post-translational peptide-modifying enzymes in Escherichia coli under uniform expression and purification conditions
Source: PLoS One. 2022 Sep 19;17(9):e0266488. doi: 10.1371/journal.pone.0266488 (PMC9484694; doi:10.1371/journal.pone.0266488)
Supplement: S3 Table — (PDF) [file pone.0266488.s014.pdf]

S3 Table. Chemical modification types studied

Type	Enzyme Type	Mass Shift[a]	Enzyme Name
Lassopeptide	Amino-peptidase+cyclase	-Leader (leader cleavage)<br>-18 Da (cyclization)	LasBCD, CapBC, AlbsBC, AtxBC, Cln1BC, Cln2BC, Cln3BC, CsegBC
	Acetyl-transferase	+42 Da (acetylation)	AlbsT
	Kinase	+80 Da (phosphorylation)	PadeK, ThcoK, PapoK
	O-methyl-transferase	+14 Da (methylation)	LasF, StspM
Glycocin	Glycosyl-transferase	+162.14 Da (glycosylation)	LcnG, PalS
Microcin	cytidylyl-transferase	+305.18 Da (cytidylation)	BamB
ComX	Prenyl transferase	+204.4 Da (prenylation)	ComQ
Pantocin	Claisen	-80 Da (Claisen condensation and decarboxylation)	PaaA
Sulfatyrotide	Sulfo-transferase	+80 Da (sulfation)	RaxST
Spliceotide	rSAM tyrosinase	-135 Da (tyramine excision)	PlpXY, PcpXY
Lanthipeptide	LanM: Dehydratase + thioether cyclase	-18 Da (dehydration)	CrnM, SgbL, BsjM, LtnM1, LtnM2, ProcM, HalM1, HalM2
	TOMM	-18 Da (dehydration)	McbCD
	halogenase	+34.5 Da (chlorination)	MibHS
	P450	+16 Da (hydroxylation)	MibO, CinX
	De-carboxylase	-44 Da (decarboxylation)	MibD, EpiD
Microviridin	Lactone cyclase	-18 Da (dehydration)	AMdnC, PsnB, MdnC, TgnB
Cyanobactin	TOMM	-18 Da (dehydration)	TruD, LynD
	Prenyl transferase	+136.2 Da (prenylation)	KgpF
Thiopeptide	P450	+16 Da (hydroxylation)	PbtO
	N-methyl-transferase	+14 Da (methylation)	PbtM1
Sactipeptide	rSAM cyclase	-2 Da (dehydrogenation)	AlbA
SCIFF/ Ranthipeptide	rSAM cyclase	-2 Da (dehydrogenation)	PapB

a. Mass shift listed is for a single modification. Enzymes can multiply-modify their peptide substrate, resulting in a total mass shift that is multiplied by the integer number of modifications performed.
