# Supplementary material for: Functional expression of diverse post-translational peptide-modifying enzymes in Escherichia coli under uniform expression and purification conditions
Source: PLoS One. 2022 Sep 19;17(9):e0266488. doi: 10.1371/journal.pone.0266488 (PMC9484694; doi:10.1371/journal.pone.0266488)
Supplement: S4 Table — (PDF) [file pone.0266488.s015.pdf]

S4 Table. Structural validation of modified peptides

Peptide	Enzyme	Mass Shift	Number Mods Expected	Number Mods Observed	Multiple Structures Possible?	LC-MS/MS Done?	Modified Structure Certainty
Cln1A2	Cln1BC	-18	1	1	NO	-	Only one structure possible
Cln2A1	Cln2BC	-18	1	1	NO	-	Only one structure possible
Cln3A2	Cln3BC	-18	1	1	NO	-	Only one structure possible
PadeA	PadeK	+80	1	1	NO	-	Only one structure possible
ThcoA	ThcoK	+80	1-2	1-2	NO	-	Only one structure possible, both modification states are considered properly modified
LasA	LasF	+14	1	1	NO	-	Only one structure possible
PalA	PalS	+162.1	1	1	YES	NO	Structure not validated
PaaP	PaaA	-80	1	1	NO	-	Only one structure possible
RaxX	RaxST	+80	1	1	NO	-	Only one structure possible
PlpA1	PlpXY	-135	1	1	NO	-	Only one structure possible
PlpA2	PlpXY	-135	1	1	NO	-	Only one structure possible
SgbA	SgbL	-18	4	4	YES	NO	Structure not validated
LtnA1	LtnM1	-18	7	7	YES	NO	Structure not validated
ProcA*	ProcM	-18	2	2	YES	NO	Structure not validated
HalA1	HalM1	-18	3	3	YES	YES	MS/MS matches literature
HalA2	HalM2	-18	7	7	YES	YES	MS/MS matches literature, but location of one cycle is not resolved
EpiA	EpiD	-44	1	1	NO	-	Only one structure possible
PsnA2	PsnB	-18	8	1	YES	YES	MS/MS supports four bicyclic cores, but cannot determine connectivity within each core
MdnA	MdnC	-18	2	2	YES	NO	Structure not validated
TruE	LynD	-18	2	2	NO	-	Only one structure possible
TruE	TruD	-18	2	2	NO	-	Only one structure possible
SboA	AlbA	-2	3	3	YES	NO	Structure not validated
PapA	PapB	-2	6	6	YES	YES	Existing knowledge of PapB is that only CxxxD/E cycles are formed, which would make only one structure possible. MS/MS supports but does not unequivocally prove this cyclization pattern.
BamA	BamB	+305.2	1	1	NO	-	Only one structure possible
