# Supplementary material for: Functional expression of diverse post-translational peptide-modifying enzymes in Escherichia coli under uniform expression and purification conditions
Source: PLoS One. 2022 Sep 19;17(9):e0266488. doi: 10.1371/journal.pone.0266488 (PMC9484694; doi:10.1371/journal.pone.0266488)
Supplement: S5 Table — (PDF) [file pone.0266488.s016.pdf]

S5 Table. New plasmids used in this work

Name	Origin	Marker	Gene	Description
pEG1128	p15A	Kan	*truD*	pLux modifying enzyme expression plasmid
pEG2192	pSC101 var2	Amp	*papoA*	RST_N peptide expression plasmid
pEG2194	pSC101 var2	Amp	*bamA*	RST_N peptide expression plasmid
pEG2195	pSC101 var2	Amp	*epiA*	RST_N peptide expression plasmid
pEG2199	pSC101 var2	Amp	*halA1*	RST_N peptide expression plasmid
pEG2200	pSC101 var2	Amp	*halA2*	RST_N peptide expression plasmid
pEG2312	pSC101 var2	Amp	*papA_tev*	RST_N peptide expression plasmid
pEG2575	pSC101 var2	Amp	*psnA2_tev*	RST_N peptide expression plasmid
pEG3017	pSC101 var2	Cm	*truE**	MBP-tag peptide expression plasmid
pEG3045	pSC101 var2	Amp	*mdnA*	HIS-tag peptide expression plasmid
pEG3046	pSC101 var2	Amp	*bmbC*	HIS-tag peptide expression plasmid
pEG3047	pSC101 var2	Amp	*strA*	HIS-tag peptide expression plasmid
pEG3048	pSC101 var2	Amp	*pqqA*	HIS-tag peptide expression plasmid
pEG3049	pSC101 var2	Amp	*sboA*	HIS-tag peptide expression plasmid
pEG3051	pSC101 var2	Amp	*tfxA*	HIS-tag peptide expression plasmid
pEG3052	pSC101 var2	Amp	*procA1.7*	HIS-tag peptide expression plasmid
pEG3053	pSC101 var2	Amp	*tbtA*	HIS-tag peptide expression plasmid
pEG3055	pSC101 var2	Amp	*pgm2*	HIS-tag peptide expression plasmid
pEG3057	pSC101 var2	Amp	*truE**	RST_N (Link-1) peptide expression plasmid
pEG3058	pSC101 var2	Amp	*mdnA*	RST_N (Link-1) peptide expression plasmid
pEG3059	pSC101 var2	Amp	*sboA*	RST_N (Link-1) peptide expression plasmid
pEG3060	pSC101 var2	Amp	*pqqA*	RST_N (Link-1) peptide expression plasmid
pEG3061	pSC101 var2	Amp	*strA*	RST_N (Link-1) peptide expression plasmid
pEG3062	pSC101 var2	Amp	*bmbC*	RST_N (Link-1) peptide expression plasmid
pEG3063	pSC101 var2	Amp	*tfxA*	RST_N (Link-1) peptide expression plasmid
pEG3064	pSC101 var2	Amp	*procA1.7*	RST_N (Link-1) peptide expression plasmid
pEG3065	pSC101 var2	Amp	*tbtA*	RST_N (Link-1) peptide expression plasmid
pEG3067	pSC101 var2	Amp	*pgm2*	RST_N (Link-1) peptide expression plasmid
pEG3121	pSC101 var2	Amp	*mdnA**	RST_N peptide expression plasmid
pEG3128	pSC101 var2	Amp	*procA**	RST_N peptide expression plasmid
pEG3132	pSC101 var2	Amp	*paaP*	RST_N peptide expression plasmid
pEG3157	pSC101 var2	Amp	*mibA*	RST_N peptide expression plasmid
pEG3161	pSC101 var2	Amp	*plpA1*	RST_N peptide expression plasmid
pEG3162	pSC101 var2	Amp	*plpA2*	RST_N peptide expression plasmid
pEG3165	pSC101 var2	Amp	*pbtA*	RST_N peptide expression plasmid
pEG3172	pSC101 var2	Amp	*ltnA1*	RST_N peptide expression plasmid
pEG3173	pSC101 var2	Amp	*ltnA2*	RST_N peptide expression plasmid
pEG3174	pSC101 var2	Amp	*crnA1*	RST_N peptide expression plasmid
pEG3175	pSC101 var2	Amp	*crnA2*	RST_N peptide expression plasmid
pEG3176	pSC101 var2	Amp	*bsjA2*	RST_N peptide expression plasmid
pEG3177	pSC101 var2	Amp	*bsjA3*	RST_N peptide expression plasmid
pEG3178	pSC101 var2	Amp	*cinA*	RST_N peptide expression plasmid
pEG3180	pSC101 var2	Amp	*lasA*	RST_N peptide expression plasmid
pEG3181	pSC101 var2	Amp	*albsA*	RST_N peptide expression plasmid
pEG3182	pSC101 var2	Amp	*mcbA*	RST_N peptide expression plasmid
pEG3194	pSC101 var2	Amp	*psnA2*	RST_N peptide expression plasmid
pEG3197	pSC101 var2	Amp	*aMdnA*	RST_N peptide expression plasmid

pEG3212	pSC101 var2	Amp	*capA*	RST_C peptide expression plasmid
pEG3213	pSC101 var2	Amp	*lasA*	RST_C peptide expression plasmid
pEG3214	pSC101 var2	Amp	*albsA*	RST_C peptide expression plasmid
pEG3215	pSC101 var2	Amp	*atxA1*	RST_C peptide expression plasmid
pEG3248	pSC101 var2	Amp	*sboA*	RST_N peptide expression plasmid
pEG3283	pSC101 var2	Amp	*papA*	RST_N peptide expression plasmid
pEG3286	pSC101 var2	Amp	*pcpA*	RST_N peptide expression plasmid
pEG3553	pSC101 var2	Amp	*cln1A1*	RST_C peptide expression plasmid
pEG3554	pSC101 var2	Amp	*cln1A2*	RST_C peptide expression plasmid
pEG3555	pSC101 var2	Amp	*cln2A1*	RST_C peptide expression plasmid
pEG3556	pSC101 var2	Amp	*cln2A2*	RST_C peptide expression plasmid
pEG3557	pSC101 var2	Amp	*cln3A1*	RST_C peptide expression plasmid
pEG3558	pSC101 var2	Amp	*cln3A2*	RST_C peptide expression plasmid
pEG3559	pSC101 var2	Amp	*cln3A3*	RST_C peptide expression plasmid
pEG3560	pSC101 var2	Amp	*csegA1*	RST_C peptide expression plasmid
pEG3561	pSC101 var2	Amp	*csegA2*	RST_C peptide expression plasmid
pEG3562	pSC101 var2	Amp	*csegA3*	RST_C peptide expression plasmid
pEG3563	pSC101 var2	Amp	*padeA*	RST_N peptide expression plasmid
pEG3564	pSC101 var2	Amp	*thcoA*	RST_N peptide expression plasmid
pEG3565	pSC101 var2	Amp	*stspA*	RST_N peptide expression plasmid
pEG3567	pSC101 var2	Amp	*lcnA*	RST_N peptide expression plasmid
pEG3568	pSC101 var2	Amp	*palA*	RST_N peptide expression plasmid
pEG3570	pSC101 var2	Amp	*raxX*	RST_N peptide expression plasmid
pEG3571	pSC101 var2	Amp	*comX*	RST_N peptide expression plasmid
pEG3572	pSC101 var2	Amp	*kgpE*	RST_N peptide expression plasmid
pEG3574	pSC101 var2	Amp	*tgnA**	RST_N peptide expression plasmid
pEG3871	pSC101 var2	Amp	*sgbA*	RST_N peptide expression plasmid
pEG3905	pSC101 var2	Amp	*truE*	RST_N peptide expression plasmid
pEG7034	p15A	Kan	*truD*	pCym modifying enzyme expression plasmid
pEG7035	p15A	Kan	*alba*	pCym modifying enzyme expression plasmid
pEG7037	p15A	Kan	*mdnC*	pCym modifying enzyme expression plasmid
pEG7043	p15A	Kan	*procM*	pCym modifying enzyme expression plasmid
pEG7047	p15A	Kan	*mibHS*	pCym modifying enzyme expression plasmid
pEG7048	p15A	Kan	*mibD*	pCym modifying enzyme expression plasmid
pEG7056	p15A	Kan	*plpXY*	pCym modifying enzyme expression plasmid
pEG7058	p15A	Kan	*pbtO*	pCym modifying enzyme expression plasmid
pEG7059	p15A	Kan	*pbtM1*	pCym modifying enzyme expression plasmid
pEG7060	p15A	Kan	*paaA*	pCym modifying enzyme expression plasmid
pEG7066	p15A	Kan	*cinX*	pCym modifying enzyme expression plasmid
pEG7067	p15A	Kan	*capBC*	pCym modifying enzyme expression plasmid
pEG7068	p15A	Kan	*lasBCD*	pCym modifying enzyme expression plasmid
pEG7069	p15A	Kan	*lasF*	pCym modifying enzyme expression plasmid
pEG7070	p15A	Kan	*albsBC*	pCym modifying enzyme expression plasmid
pEG7071	p15A	Kan	*albsT*	pCym modifying enzyme expression plasmid
pEG7073	p15A	Kan	*mcbCD*	pCym modifying enzyme expression plasmid
pEG7074	p15A	Kan	*mibO*	pCym modifying enzyme expression plasmid
pEG7076	p15A	Kan	*ltnM1*	pCym modifying enzyme expression plasmid
pEG7077	p15A	Kan	*ltnM2*	pCym modifying enzyme expression plasmid
pEG7078	p15A	Kan	*crnM*	pCym modifying enzyme expression plasmid
pEG7079	p15A	Kan	*bsjM*	pCym modifying enzyme expression plasmid

pEG7127	p15A	Kan	*psnB*	pCym modifying enzyme expression plasmid
pEG7130	p15A	Kan	*amdnC*	pCym modifying enzyme expression plasmid
pEG7132	p15A	Kan	*atxBC*	pCym modifying enzyme expression plasmid
pEG7133	p15A	Kan	*cln1BC*	pCym modifying enzyme expression plasmid
pEG7134	p15A	Kan	*cln2BC*	pCym modifying enzyme expression plasmid
pEG7135	p15A	Kan	*cln3BC*	pCym modifying enzyme expression plasmid
pEG7136	p15A	Kan	*csegBC*	pCym modifying enzyme expression plasmid
pEG7137	p15A	Kan	*padeK*	pCym modifying enzyme expression plasmid
pEG7138	p15A	Kan	*thcoK*	pCym modifying enzyme expression plasmid
pEG7139	p15A	Kan	*stspM*	pCym modifying enzyme expression plasmid
pEG7141	p15A	Kan	*lcnG*	pCym modifying enzyme expression plasmid
pEG7142	p15A	Kan	*palS*	pCym modifying enzyme expression plasmid
pEG7143	p15A	Kan	*sgbL*	pCym modifying enzyme expression plasmid
pEG7144	p15A	Kan	*raxST*	pCym modifying enzyme expression plasmid
pEG7145	p15A	Kan	*comQ*	pCym modifying enzyme expression plasmid
pEG7146	p15A	Kan	*kgpF*	pCym modifying enzyme expression plasmid
pEG7147	p15A	Kan	*tgnB*	pCym modifying enzyme expression plasmid
pEG7149	p15A	Kan	*papB*	pCym modifying enzyme expression plasmid
pEG7152	p15A	Kan	*pcpXY*	pCym modifying enzyme expression plasmid
pEG7160	p15A	Kan	*lynD*	pCym modifying enzyme expression plasmid
pEG7166	p15A	Kan	*papoK*	pCym modifying enzyme expression plasmid
pEG7169	p15A	Kan	*epiD*	pCym modifying enzyme expression plasmid
pEG7171	p15A	Kan	*bamB*	pCym modifying enzyme expression plasmid
pEG7172	p15A	Kan	*halM1*	pCym modifying enzyme expression plasmid
pEG7173	p15A	Kan	*halM2*	pCym modifying enzyme expression plasmid
