# Supplementary material for: Functional expression of diverse post-translational peptide-modifying enzymes in Escherichia coli under uniform expression and purification conditions
Source: PLoS One. 2022 Sep 19;17(9):e0266488. doi: 10.1371/journal.pone.0266488 (PMC9484694; doi:10.1371/journal.pone.0266488)
Supplement: S6 Table — (PDF) [file pone.0266488.s017.pdf]

S6 Table. Enzyme and peptide amino acid sequences

Name	Sequence
AlbA	MFIEQMFPFINESVRVHQLPEGGVLEIDYLRDNVSISDFEYLDLNKTAYELCMRMDGQKTAEQILAEQCAVYDESPEDHKDWYYDMLNMLQNKQVIQLGNRASRHTITTSGSNE<br>FPMPLHATFELTHRCNLKCAHCYLESSPEALGTVSIEQFKKTADMLFDNGVLTCEITGGEIFVHPNANEILDYVCKKFKKVAVLTNGTLMRKESLELLKTYKQKIIVGISLDSV<br>NSEVHDSFRGRKGSFAQTCKTIKLLSDHGIFVRVAMSVFEKNMWEIHDMAQKVRDLGAKAFSYNWVDDFGRGRDIVHPTKDAEQHRKFMEYEQHVIDEFKDLIPIIPYERKRAA<br>NCGAGWKSIVISPFGEVRPCALFPKEFSLGNIFHDSYESIFNSPLVHKLWQAQAPRFSEHCMKDKCPFSGYCGGCYLKGLNSNKYHRKNICSWAKNEQLEDVVQLI
AlbsA	MDSLLSTETVISDDELLPIEVGGTAELTEGQGGGQSEDKRRAYNC
AlbsB	MPELPRFATAPRHVRALDFGHVLVLIDYRSNHVQCLLPAAAAHWTATARTGRLDTMPAALATQLLTSALLVPRPTATPWTAPVAAPPAPPSWGGSEHPAGTSRPRARHRHSTTA<br>AAALACVLAIKAAGPTRYAMQRLTTVVKAAASTCRRPATPAQATAAALAVRQACWYSPARTACLEESAATVILLATRRLSSTWCHGVAPDPIRLHAWVETEDGTPVAEPASTLA<br>YTPALTIGGHHQHQP
AlbsC	MIFGGFSTTREVRQRPGNAEFIATDSPIWRLGRSPARCVAADHGQRRLVVLGECGATDGELSRLATAGLPTDITWRWPGVYVVVEEQPERTVLHTDPAAALPVYATPWQGGWAW<br>STSARILARLTEAPIDGQRLACSVLAPSVPALSGTRTFFAGIEQLALGSRIELPVDGSRLRVTVRWRPDPVPGEPYHRLRTALTEAVALRVNRAPDLSCDLSGGLDSTSLAVLA<br>AVCLPESHHLNAITIHPEGDESGADLRYARLAAAHHGRIRHHLLPLAAEHLPYTEITAVPPTTEPAPSTLTRARLAWQLDWMRQHLGSRTHMTGDGGDSVLFQPPAHLADLLRH<br>RQWRRTLSESLGWARLRHTSVLPLLRGAATLARTSRRSGLQDLARALAGAGQQGDGRGNVSWFAPLPLPGWATPTARRLLLDAADEAISTADPLPGLDTSLRVLIDEIREVART<br>AAADAELADAHGTTLHNPFLDPRTIDAVLRTPIAHRPAVHSYKPALGHAMQDLLPGAVARRSTKGSFNADHYAGMRANLPALTALADGHLADLGLLEPTRFRSHLRQAAAGIPM<br>PLAAIEQALSAEAWCHAHHATPSPAWTTQPPEHPHA
AlbsT	MSTSPEQTLWISTDTCGLGPYRADLVDTYWQWEQDPTLLVGYGRQSPQSLEARTEGMAHQLRGDNIRFTIYDLCSSTPTPAGVATLLPDHSVRTAEYVIMLAPEARGRGLGTTA<br>TQLTLDYAFHITNLRMVWLKVLAPNTAGIRAYEKAGFRTVGALREAGYWLGKVCDEVLMDALAKDFTGPSAVHAALTGASGRQLRRAP
AMdnA	MPENRQEDLNAQAVPFFARFLEGQNCEDLTDEESEAVSGGKRGQTRKYPSDCEDGNGVTGKLRDEDIAVTLKYPSDNEDNGGGEIVTLKFPSDDDDQPVG
AMdnC	MNVLIITHSHDNESISLVTQAIESQGGKAFRFDTDRFPTEVQLDIYYSNTEKCVLVADDQKLDLNEVTAVWYRRIAIGGKIPPTMDKQLRQASIQESRATIQGMIASIRGFHLD<br>PVPNIRRAENKQLQLQVARKIGLDTPRTLTTNNPQAVKEFAAECQQDVITKMLSSFAIYDEKGGEQVVFTNPVKSEDLENLEGLRFCPMTFQEKIAKVLELRITIVGKSILTAA<br>VNSQALDKSRYDWRKQGVALLDAWQTHTLPQDVADKLLQLMAHFGLNYGAIDVILTPDNRYVFLEVNPVGEFFWLERCPGLPISQAIAKVLLSHI
AtxA1	MHTPIISETVQPKTAGLIVLGKASAETRGLSQGVEPDIGQTYFEESRINQD
AtxB	MYELNDGVGLALVDQHPIFLDLKTDRYLSLSPDGAAVLLGAAPATKESPLFLGLESIGLVKNGPSGLKPCQIAVATGSAPPRKVQFESLSLLLLRLIRARLDQRALLKRVTDLK<br>KAGTIAQTKNRDCALSLLGSVETEAKACRTLLSSTDKCLPDAFAIATHLRRRGVDAKLVFGVRLPFAAHAWVQVDDIVVGDRPDRILAFTPILVV
AtxC	MRYVASFFVRGHVSTPALRHPEPKGFAYAKVSGGLSVWSDAPIRHRAPLITVGAVFDRASFKGLDCDLSGLRQDGLNTLKAETFGPYLALEVADNGTLRVYRDPSGGAPCYYLQ<br>TEDGFWLASDADLLFTHSGVHPSVSLPGLIEHLRRPEFQNEGTCLNVKQVRPGEQVDLSLSGEVRACLFPPASSLRPPELHRAYDDIKAELRALILRSIKAYASDFPHVVVSFS<br>GGLDSSVVAAGLAQTSTKVLLHTFKGPDAKGDETAFAAECAAYLGLSLEIDTLSIDDVDLSATISPHLPRPSTSFFLPSLLRGFSTSSQTRTGGAIFSGNGGDSVFCFMHSATP<br>LADLMCRPSGLTPFMQTWADVQKLTRASATEVLRRALKTAMARGYIWPESNLLLSRDTSSSRLTPDSVLSSLEGILPGRLRHLALIRRAHNTFEPFAPWRTPPVVHPLMAKPIQ<br>AFCLSLPSWMWVSGGKDRSLVRDAFEGLLPDSVRLRKSKGSPAGFLHALYRAKGRQMIERIRHGYLRREGIIDISTGPDALFSEGFRNPRVMHRFFELAATEVWIDHWRNWRRP<br>RT
BamA	LKIRKVKIVRAQNGHYTN
BamB	MEGLYQLKVHSRIHKLQNNIAIGSMPPHALIIEDAPEYLSNVLRFFSSKKTIKEAEVYLSDNTNLSSNEINLLLGDLIENEIIVKQNYDSNNRYSRHSLYYEMIDANAENAQKI<br>LAEKTVGLVGMGGIGSNVAMNLAAAGVGKLIFSDGDTIELSNLTRQYLYKEDQVGLSKVESAKEQLQLLNSEVELIPVCESISGEELFDNHFSECDFVVLSADSPFFVHEWINN<br>AALKYGFSYSNAGYIETYGAIGPLVIPGETACYECYKDKGDLYLYSDNKEEFSVNLNESFQAPSYGPLNAMVSSIQANEVIRHLLGLKTKTSGKRLLINSEIYKIHEENFEKKN<br>NCLCSDIKGEKLSKNTLNSDKELHEVYIEERESDSFNSILLDKTMSKLVKINKEETKILDIGCATGEQALYFANKGAKVTAVDISDDMLKVLDKKASNINAGSIKTMRGNIESI<br>EVNDTFNYIVCNNILDYLPEIDRTLRKLNMFLKNDGTLIVTIPHPVKDGGGWRKDYYNGKWNYEEFILKDYFNEGLIEKSREDKNGETVIKSIKTYHRTTETYFNSFTDAGFKV<br>VSLLEPQPLSTVSETHPILFEKCSRIPYFQVFVLKKEDRHAI
BmbC	MGPVVVFDCMTADFLNDDPNNAELSALEMEELESWGAWDGEATS
BsjA2	MTNEEIIVAWKNPKVRGKNMPSHPSGVGFQELSINEMAQVTGGAVEQRATPTLATPLTPHTPYATYVVSGGVVSAISGIFSNNKTCLG
BsjA3	MTNEEIIVAWKNPKVRGKNMPSHPSGVGFQELSINEMAQVTGGAVEQRATPATPATPWLIKASYVVSGAGVSFVASYITVN
BsjM	MIKNVNLKEAIKGLTVSERYDTLKNSGVNLNLNISALEEWRNRKNLLADEDFTEMLTVLEYDPVYFSHAINENIEEHIDIYKSKILGENWFIVLNDILDELDNPIEYKKEMNHS<br>YLLRPFLLYAEKEMNKYIVNRKELLPVEPQVIQQIMENLASKLFAVSVKSFVLELNISKLKDELAGETPDERFHSFIRLMGEKTRLVDFYNEYIVLSRILVNITILFVNNIIEL<br>FERLQESKLDIVKKLGVQEEFKISNISIGEGDTHQQGRSVIVLTFVSGKKVVYKPKNLKVVSAYNSLIDWINNKNNILKMPSYNTLIYDDFVIEEFVEKRDCKSIEEVKKYYIR<br>YGQILGIMYILNGNDFHMENLIASGEYPIIVDLETLLQNIINFKNKPSADLITTKKMLNLVNSTLLLPEKLLKGDITDEGIDMSALAGKEQHLERREYQLKNLFTDNMVFDLEK<br>VKIEGANNIPKLNGENVDYSTYIDEIVVGFENICNLFIQYRDELLHSGILEEFKDVKVRHVLRNTVVYAKMLANTYHPDYLRDSLNREQVLENIWVHPFERKEFIKSEMEDILN<br>NDIPIFFSYASSKDIIDSNGKLHKNVMEISGYERFTTKLKELNPFLIEQQVSVINIKTGRYGDKKFEKNYSVRDVATEKKDNPIDFLQEAMNIGDKILEHAIICDETKTISWLT<br>INNHHDKNWEIGPISGEFYDGLAGISLFYHYLYKKSHNVEYKKIRDYAFNMAKVKALSLKYDSGLTGYASLLYTAHKIVQDEPRKQYKDVINEVFKYIDESKVVTAKYNWLHGT<br>ASIIHVLLNLYEDSRDMAYLTKCIQYGKYLVKQIKEHKDMLAPGFSQGISSVIMVLVRLSKKCEVEEFLELALELMEMERNKLGNLSESNWLNGLVGIGLSRIKLKGLDSNLQV<br>DNDIELVLDGVMNSLYSKDDTLSCGNSGTVELFLSLFEQTKKKEYLDMAKAICGKMIEESRISFEYQTKSLPGLELVGLYSGLAGIGYQFLRISDVEDIASIATLD
CapA	MVRFLAKLLRSTIHGSNGVSLDAVSSTHGTPGFQTPDARVISRFGFN
CapB	MQPDLEVVDVRRGESFKAWSHGYPYRTVRWHFHPEFEVHLIVETTGQMFVGDYVGGFGPGNLVLMGPNLPHNWVSDVPEGKTVAERNLVVQFGQAFVSRCEDSLTEWRHVETLL<br>ADARRGVQFGPRTSEAIKPLFAELIHARGLRRIVLFLSMLQILVDATDRELLASPAYQADPSTFASTRINHALAYIGKNLANELRETDLARLAGQSVSAFSHYFRRHTGLPFVQ<br>YVNRMRINLACQLLMDGDASVTDICFRSGFNNLSNFNRQFLAVKGMSPSRFRRYQALNDASRDASEAAAKRGAGIAGAPAIVPAAQARGEARPIPEVLLSG
CapC	MMLTASSTPASGNPAARALRAAAFALALGGACVAHAAPLRIGMTFQELNNPYFVTMQKALNEAAASIGAQVIVTDAHHDVSKQVSDVEDMLQKKIDILLVNPTDSTGIQSAIVS<br>AKKAGAVVVAVDANANGPVDSFVGSKNFDAGAMSCEYLAKAINGGGEVAILDGIPVVPILERVRGCRAALAKFPNVKIVDVQNGKQERATALTVTENMIQAHPKLKGVFSVNDG<br>GSMGALSAIEASGKDIRLTSVDGAPEAVAAIQKPNSKFIETSAQFPRDQIRLAIGIGLAKKWGANVPKAIPVDVKLIDKGNAKTFSW
CinA	MTASILQSVVDADFRAALIENPAAFGASTAVLPTPVEQQDQASLDFWTKDIAATEAFACKQSCSFGPFTFVCDGNTK
CinX	MALKTCEEFLRDALDPDRFGREMKAVTEIPEIVKLGHRHGYGFTAEEFLTKAMSFGAPPAGAAAPGESASVPGQNGSSPGHAARAAMAGPEAGATSFAHYEYRLDELPEFAPVV<br>AELPKLKVMPPSVGPDRFAARYRDEDMRTISMSPADPAYQAWHQELAGRGWRDAEDTAAAPDAPRRDFHLLNLDEHVDYPGYEEYFAAKTRVVAALENLFGGDVRCSGSMWYPP<br>SSYRLWHTNADQPGWRMYLVDVDRPFADPDRTSFFRYLHPRTREIVTLRESPRIVRFFKVEQDPEKLFWHCIANPTDRHRWSFGYVVPENWMDALRHHG
Cln1A1	MTPIQSKFCLLRVGSAKRLTQSFDVGTIKEGLVSQYYFA

Cln1A2	MTQVSPSPLRLIRVGRALDLTRSIGDSGLRESMSSQTYWP
Cln1B	MPLWLAQDVHAVALDEDIVVLDAVSDAYLCLVGASALISLGSERSVSADPVAAETLREAGLVGPHPSGATRPIPPKPTIDLPDAARQAQGRELRAAAWAGAATAIDFRRRSFRQ<br>LLARAGQRPPGQAAAPADEVLAAAAVFMRLRPWSPVGGACLMRSYYLLRHLRILGFDADWIIGVRTWPFMAHCWLQVGAVALDDDVERLTAYTPILAV
Cln1C	MGDYLALYWPRGMPGVAADAMRAAIEAEGAWTLAFEAYQLVVYVKGPRAPKVRALPDQGGVVIGELFDTAATREGRVQDFPIALIKDVAAQDAARILATHAWGRYVAVLKAGDR<br>PPWIFRDPSGAVECLAWVRDEVTIISSDVAAQRAWSPDRLAIDWSGLGRVLARGNLWGEICPLAGVTAIAPGTARCDLGDAALSLWRPGDHARRSRHDVSPRDLARVVDASVAA<br>LARDRSAILVEISGGLDSAIVATSLARCGAPVVAGINHYWPEPEGDERRWAQDIADRCGFRLIAGQRQRLLLDEAKLLRHAQGPRPGLNAQDPDLDHDLAEQAKALGADALFSG<br>QGGDGVFYQMANAALAADILMGKPAPMGRAASLAAVARRARATVWSLCGQAMFPSRAFAAGMPPPSFLSAGLAPPPVHPWIADQRGVSPAKRIQIRGLTNIQCAFGDSLRGRAA<br>DLLYPLMAQPVMELCLSIPAPLLAVGALDRPFARAAFADRLPPRSLVRRSKGDVTVFFSKSLAASLPALRPFLLDGRLAEQGLIDRAKLEPLLHPEPMIWRDSVGEVMLAAYLE<br>AWVRAWEAKLRVS
Cln2A1	MNTLKTRLIRFGSAKRLTRAGTGVLLPETNQIKRYDPA
Cln2A2	MTTPKFRLIRLGSAKRLTRSGIGDVFPEPNMVRRWD
Cln2B	MTLTWRPGVHAVMVEDDLVLLDEAADAYVCLLDGAKVVSVRADGALSFNPPHAAEDMIAGGLVEPSSSAAASANPPAKLPCTPLARLSRPRHVKVRPAEAALFLIQAWGVARAV<br>RRWPMARLLEALRGDRAAEPAKGRRSMAEACAVFDALLAWSPFDGECLFRSVLRRRFLMALGHSPDLVIGVRTWPFRAHCWLQSGVDALDDWPERLCAYRPILAASASQGR
Cln2C	MSYLLMTWPPGQPSVEADALHAAFNGQGGWSLVLERFCLRVYVRGAAAPAVTLTPKGGVLIGEMFDRAATETGAVAAYDLSRLGDDDGMAVARRVVDEAWGRYVLVLPVKERRP<br>VVLREPLGALDALIWRKGDVWCVGADVPPGLEPKDLGVEETRLTHLIAEPDLASASLPLTGVAAVMPGTAVDETGQVHRLWTPARFARSPRTDAWTAAERIPLVTRACIAALSA<br>NRSGILCEISGGLDSAIVATSLKAEGAKISSGINFHWPQAEADERPYARAVAKSVRTRLQVVASRVAPVDPETFDEIVVARPSFNAIDPVYDTVLAQRLIQGGEGALFTGQGGD<br>AVFYQMPAPQLSLDLLARGPRRRGLMGLSRRTNRSVWSLLRMGLRAPVRATFPYGARGADRPPMHPWLEDARGVGAAKRIQIEALVANQAVFEASRRGAAAHLVHPLLSQPLVE<br>LCLSTPAAVLAGAEQDRAFVRSAFRAQLPRLVLDRQSKGDLSVFFAKGVARSLPGLRPRLLEGRLAARGLIDVEALSQAMQPEAMIWRDGSAEILCLAVLESWLRSWEARGA
Cln3A1	MQRIIDETTDGLIELGAASVQTQGDVLFAPEPGVGRPPMGLSED
Cln3A2	MERIEDHIDDELIDLGAASVETQGDVLNAPEPGIGREPTGLSRD
Cln3A3	MEFEGIPSPDARIDLGLASEETCGQIYDHPEVGIGAYGCEGLQR
Cln3B	MRVAVPDHLAYCVKQGGVTFLDVRGDRYFGLPPVLEHAFVAIAEADFLLKEPNSLLEPLEALGVLVRGQARRADLTIPSANLSWVDEVSPTPPRLDPASLVATVTSVIRTRLSQ<br>KSKSLQALLEEVRTRRPGSPAHNWQLMRRLTAGFRASRAWAPIEPICLLDSLALLDFLHRRGLYPHIVFGVIRQPFAAHCWVQADDVVLNDRLDHVGEYTPILVV
Cln3C	MEDYVVLIWPALAEAPARDLIRRLPKLKTVIETSGLVVLRPENGAGLRVGGNGVVLGSVFRTGGDRETVAEFSESEASAIATSRGQQLVTEFWGGYLAVLGDASRSEVMVLRDP<br>SGAMPAYCLVHGEVQIICSRLEVLEDAGLGQQALNWDVVAQLLAFPNLRGRSTGLKGVEELLPGCRLTFTGGLKTETLTWNPWLFARPSAQAPERGVAATAVRQAVEVSVRKWA<br>DQSSPVLLELSGGLDSSIIACCLDEPRTAATFVNFVTPTAEGDERGYARLVAKAADKQLIEQDIRADEVDVTRPRPGRHPRPASQALLQPLEQACAELAPQLGARSFFSGLGGD<br>NVFCSIATASPAADALLTSGLGRQFWAAIGDLCARHNCTVWAALSATLKKLLRSDRRLVIKPNLDFLSFREDAIDRPDHPWLEVAADRLPGKREHVASILLAQGFLDRYEHAQV<br>AAVRFPLLTQPVMEACLRVPTWMANHQGRNRAVARDAFFDRLPPRVRDRQTKGGLNAFMGVAFERNRQALARHLLDGRLVQRGLIDAVAIKSALASPVLEGGAMNRLLYLADVE<br>SWVRSWEDV
ComQ	MKEIVKQNISNKDLSQLLCSFIDSKETFSFAESAILHYVVFGGENLDVATWLGAGIEILILSSDIMDDLEDEDNHHALWMKINRSESLNAALSLYTVGLTSIYSLNTNPLIFKY<br>VLRYVNEAMQGQHDDITNKSKTEDESLEVIRLKCGSLIALANVAGVLLATGEYNETVERYSYYKGIVAQISGDYHVLLSGNRSDIEKNKQTLIYLYLKRLFNNASEELLYLFSH<br>KDLYYKALLDREKFEEKLIQAGVTQYISVLLEIYKQKCFSTIEQLNLDKEKKELIKESLLSYKKGDTRCKT
ComX	MQDLINYFLNYPEALKKLKNKEACLIGFDVQETETIIKAYNDYYRADPITRQWGD
CrnA1	MSELSMEKVVGETFEDLSIAEMTMVQGSGDINGEFTTSPACVYSVMVVSKASSAKCAAGASAVSGAILSAIRC
CrnA2	MSESNMKKVVGETFEDLSIAEMTKVQGSGDVMPESTPICAGFATLMSSIGLVKTIKGNVKSFSVLI
CrnM	MNDINKNKTKTINEKIKIFTKEEVIDISYFEEWRSVRTLLNENYFKIMLEEMNISKNQFSYALQPLNDEFKLHTNVKNEEWIKCFNRVINNFNYKNINYKVGLYLPIQPFSVYL<br>QEKLKEILKKLNNIKINDKIIDAFIEAHLIEMFDLVGKVIALKFEDYKQINFLKNTNNGTRLEEFLRSTFYSRKSFLKLFNEFPVLARVCTVRTIYLINNFSAIIQNINSDYLE<br>IQEFLNVDFLNLTNITLSTGDSHEQGKSVSILYFDEKKLIYKPKNLKISEIFESFIDWYTNVSNHKLLDLKIPKGIFKDDYTYNEFIEPNYCENKREIENYYNRYGYLIAICYL<br>FNLNDLHVENVIAHGEYPVIVDIETSFQVPVQMEDDTLYVKLLRELELESVSSSFLLPTNLSFGMDDKVDLSALSGTMVELNQQILAPVNINMDNFHYEKSPSYFPGGNNIPKN<br>NKSVTVDYKKYLLNIVTGFDEFMKYTQENQLEFIEFLKKFSDKKIRVLVKGTEKYASMIRYSNHPNYNKEMKYRERLMMNLWAYPYKDKRIVNSEVQDLLFNDIPIFYSFPNSR<br>DLIDSRGLVYKDYLPVTGLQKAIDRVKDTSVKSLFDQKLILQSSLGLWDEILNKPVQKKELLFEKQNFNYVKEAINIAELLIGYLIETDDQSTMLSIDCSEDKHWKIVPLDESL<br>YGGLSGIALFFLDIYKITKDEKYFNYYDKIISTAIKQCKATIFSSSFTGWLSPIYPLILEKKYFGTMKDKKFFDYTMEKLSNMTEEQINNMDGMDYISGKAGIVKLLISAYRES<br>KNNENIGLALSKFSNDLIQNIGTGKVSELQNVGLAHGISGIMVVVASLDTFKSEYIREQLAIEYEMFCLREDSYKWCWGISGMIQARLEILKLSPECVDKKELNLLIKRFKNIL<br>NQMINEDSLCHGNGSIITTMKMIYMYTQDTEWNSLINLWLSNVSIYSTLQGYSIPKLGDVTIKGLFDGICGIGWLYLYSNFSIENVLLLEV
CsegA1	MTKKNATQAPRLVRVGDAHRLTQGAFVGQPEAVNPLGREIQG
CsegA2	MTKTHRLIRLGDAQRLTQGTLTPGLPEDFLPGHYMPG
CsegA3	MTSRFQLLRLGKADRLTRGALVGLLIEDITVARYDPM
CsegB	MDLWLSAGVYAVMIDDDVVFLDVATNAYFCLPAVGSVLALEGRSLRVAARELAEDLIQAGLASAAAAIEPPPSTPAPVRTARAVLEALPARERPRPRLAHWRQAIMAGLASRAA<br>ERRPFAQRLPPPSTGVSPPASEGLLADLDAFRRLQPWLPFDGACLFRSQMLRDYLLALGHRVDWIFGVRTWPFGAHCWLQAGDLVLDDEAERLIAYHPIMVR
CsegC	MGYAALTYPGGLAAAAFDEMVEALIDAGWTLALRAFRLAVLTDGQAPAVSPLMGRGGVAGVLIGEAFDRRATLGGAVARAALDGLADIDPLEAGRHLIETAWGGYVGMWIGRAE<br>AGPTLLRDPSGALEALAWRRDGVTVMSARPLTGRAGPADLAIDWPRIVQILADPISAALGPPPLTGLATIDPGAAVHGADGQERSVLWTPAAVVRGARHRPWPSRQDLRRTIDA<br>TVAALASDAGPIVCEISGGLDSAIVATSLAASGLGPQLTVNFYGDQPEADERGYAQAVAERIGAPLRTLRREPFAFDETVLAAAGQAARPNFNALDPGYDAGLVGALEAIDARA<br>LFTGHGGDTVFYQVAASALAADLLGGAPCEGSRRARLEEVARRTRRSIWSLAWEAFSGRPSTVSIEGQLLRQEAERIRRVGLTHPWVGGLSSVTPAKRQQIRALVSNLNAHGAT<br>GRAERARIVHPLLAQPVVEACLAIPAPILSAGEGERSFAREAFADRLPPSIVGRRSKGEISVFLNRSLAASAPFLRGFLLEGRLAARGLIDRDELAAALEPEAIVWKDASRDLL<br>TAAALEAWVRHWEARIGEGEAAEGERAAGRGTAATGPRTSARKANTR
EpiA	EAVKEKNDLFNLDVKVNAKESNDSGAEPRIASKFICTPGCAKTGSFNSYCC
EpiD	MHGKLLICATASINVININHYIVELKQHFDEVNILFSPSSKNFINTDVLKLFCDNLYDEIKDPLLNHINIVENHEYILVLPASANTINKIANGICDNLLTTVCLTGYQKLFIFP<br>NMNIRMWGNPFLQKNIDLLKSNDVKVYSPDMNKSFEISSGRYKNNITMPNIENVLNFVLNNEKRPLD
HalA1	MTNLLKEWKMPLERTHNNSNPAGDIFQELEDQDILAGVNGAENLYFQGCAWYNISCRLGNKGAYCTLTVECMPSCN
HalA2	MVNSKDLRNPEFRKAQGLQFVDEVNEKELSSLAGSENLYFQGTTWPCATVGVSVALCPTTKCTSQC

HalM1
MRELQNALYFSEVVFGPNLEKIVGEKRLNFWLKLIGEDPENLKEFLSRKGNSFEEQTLPEKEAIVPNRLGEEALEKVREELEFLNTYSTKHVRRVKELGVQIPFEGILLPFISM
YIEKFQQQQLRKKIGPIHEEIWTQIVQDITSKLNAILHRTLILELNVARVTSQLKGDTPEERFAYYSKTYLGKREVTHRLYSEYPVVLRLLFTTISHHISFITEILERVANDRE
AIETEFSPCSPIGTLASLHLNSGDAHHKQRTVTILEFSSSLKLVYKPRSLKVDGVFNGLLAFLNDRTGEVIKDQYCPKVLQRDGYGYVEFVTHQSCQSLEEVSDFYERLGSLMS
LSYVLNSSDFHFENIIAHGPYPVLIDLETIIHNTADSSEETSTAMDRAFRMLNDSVLSTGMLPSSIYYRDQPNMKGLNVGGVSKSEGQKTPFKVNQIANRNTDEMRIEKDHVTL
SSQKNLPIFQSAAMESVHFLDQIQKGFTSMYQWIEKNKQEFKEQVRKFEGVPVRAVLRSTTRYTELLKSSYHPDLLRSALDREVLLNRLTVDSVMTPYLKEIIPLEVEDLLNGD
VPYFYTLPEERALYQEASAINSTFFTTSIFHKIDQKIDKLGIEDHTQQMKILHMSMLASNANHYADVADLDIQKGHTIKNEQYVEMAKDIGDYLMELSVEGENQGEPDLCWIST
VLEGSSEIIWDISPVGEDLYNGSAGVALFYAYLFKITGEKRYQEIAYKALVPVRRSVAQFQHHPNWSIGAFNGASGYLYAMGTIAALFNDERLKHEVTRSIPHIEPMIHEDKIY
DFIGGSAGALKVFLSLSGLFDEPKFLELAIACSEHLMKNAIKTDQGIGWKPPWEVTPLTGFSHGVSGVMASFIELYQQTGDERLLSYIDQSLAYERSFFSEQEENWLTPNKETP
VVAWCHGAPGILVSRLLLKKCGYLDEKVEKEIEVALSTTIRKGLGNNRSLCHGDFGQLEILRFAAEVLGDSYLQEVVNNLSGELYNLFKTEGYQSGTSRGTESVGLMVGLSGFG
YGLLSAAYPSAVPSILTLDGEIQKYREPHEA

HalM2
MKTPLTSEHPSVPTTLPHTNDTDWLEQLHDILSIPVTEEIQKYFHAENDLFSFFYTPFLQFTYQSMSDYFMTFKTDMALIERQSLLQSTLTAVHHRLFHLTHRTLISEMHIDKL
TVGLNGSTPHERYMDFNHKFNKTSKSKNLFNIYPILGKLVVNETLRTINFVKKIIQHYMKDYLLLSDFFKEKDLRLTNLQLGVGDTHVNGQCVTILTFASGQKVVYKPRSLSID
KQFGEFIEWVNSKGFQPSLRIPIAIDRQTYGWYEFIPHQEATSEDEIERYYSRIGGYLAIAYLFGATDLHLDNLIACGEHPMLIDLETLFTNDLDCYDSAFPFPALARELTQSV
FGTLMLPITIASGKLLDIDLSAVGGGKGVQSEKIKTWVIVNQKTDEMKLVEQPYVTESSQNKPTVNGKEANIGNYIPHVTDGFRKMYRLFLNEIDELMDHNGPIFAFESCQIRH
VFRATHVYAKFLEASTHPDYLQEPTRRNKLFESFWNITSLMAPFKKIVPHEIAELENHDIPYFVLTCGGTIVKDGYGRDIADLFQSSCIERVTHRLQQLGSEDEARQIRYIKSS
LATLTNGDWTPSHEKTPMSPASADREDGYFLREAQAIGDDILAQLIWEDDRHAAYLIGVSVGMNEAVTVSPLTPGIYDGTLGIVLFFDQLAQQTGETHYRHAADALLEGMFKQL
KPELMPSSAYFGLGSLFYGLMVLGLQRSDSHIIQKAYEYLKHLEECVQHEETPDFVSGLSGVLYMLTKIYQLTNEPRVFEVAKTTASRLSVLLDSKQPDTVLTGLSHGAAGFAL
ALLTYGTAANDEQLLKQGHSYLVYERNRFNKQENNWVDLRKGNAYQTFWCHGAPGIGISRLLLAQFYDDELLHEELNAALNKTISDGFGHNHSLCHGDFGNLDLLLLYAQYTNN
PEPKELARKLAISSIDQAHTYGWKLGLNHSDQLQGMMLGVTGIGYQLLRHINPTVPSILALELPSSTLTEKELRIHDR

KgpE
MKNPTLLPKLTAPVERPAVTSSDLKQASSVDAAWLNGDNNWSTPFAGVNAAWLNGDNNWSTPFAGVNAAWLNGDNNWSTPFAADGAE

KgpF
MINYANAQLHKSKNLMYMKAHENIFEIEALYPLELFERFMQSQTDCSIDCACKIDGDELYPARFSLALYNNQYAEKQIRETIDFFHQVEGRTEVKLNYQQLQHFLGADFDFSKV
IRNLVGVDARRELADSRVKLYIWMNDYPEKMATAMAWCDDKKELSTLIVNQEFLVGFDFYFDGRTAIELYISLSSEEFQQTQVWERLAKVVCAPALRLVNDCQAIQIGVSRAND
SKIMYYHTLNPNSFIDNLGNEMASRVHAYYRHQPVRSLVVCIPEQELTARSIQRLNMYYCMN

LasA
MDKRVRYEKPSLVKEGTFRKTTAGLRRLFADQLVGRRNI

LasB
MKGEEMLGHPQTGFVVLPDNDATGDVTGRLLPWGDVVTVYPSGRPWIIGNCWDRPVLVHDGVIVLGHTSVTRDQIARHGNDPHRLLDEADGAFHAAVLIGHEVHVRGSAYGVCR
LYTCVVDGVTLVSDRTDVLQRLAGTDVDVDVLAGHLLEPIPHWLGEQPLLTSVEPVPPTHHVILTPDARSRLRPSRRRRPEPSLGLRDGAELVRERLAAAVATRVDSPALITSE
LSGGYDSTSVSYLAARGKAEVVLVTAAGRDSTSEDLWWAERAAAGLPELDHVVLPADELPFTYAGLTEPGALLDEPCTAVAGRERVLALVRKAAARGSTLHLTGHGGDHLFTSL
PTPFHDLFRTRPVAALRQLRAFGALAAWPTRKLMRELADRRDHSTWWRAHARPQNGQPDPHSPMLGWAIPPTVPAWVTADGVRAIELGILEMAERAEPLGHARGEHAELDSIFE
GARMARGLNRMATHAGVPLAAPFHDDRVVEACLSIRPEERISAWQYKPLLNAAMQGVVPSTVLDRSAKDDGSIDVAYGLQEHRDELVALWESSRLAETGLIDAGMLRRLCAQPS
SHELEHGSLYATIACELWLRGLDQDRTQRY

LasC
MPVQLRRHVSFTATEYGGVLLDETKGAYWRLNTTGAEVVRAMGEAERDEIVRHVVATFDVDAQTAAQDVDVLLAELRDAGLVAS

LasD
MSVNMALRGHGMSGRRRRLDATRARLAVVVARVLNLLPPRLIRRCLRVLSRGARPASIEAAEAARRTVVAVSPAAAGAYGCLIRSIATTLVLRSRGQWPTWCVGVRAEPPFGAH
AWIEAEERLVDEPGTMHTYRRLITVGPLSRKVR

LasF
MSIELTPSLADLVDPLPGHALRAAATLRLADLIAAGADTAPALAAAARIDADAIARLMRYLCSRGIFQAHEGRYALTEFSELLLDEDPSGLRKTLDQDSYGDRFDRAVAELVDV
VRSGEPSYPRLYGSTVYDDLAADPALGEVFADVRGLHSAGYGEDVAAVAGWSSCLRVVDLGGGTGSVLLAVLERHPSLSGAVLDLPYVAPQAKKALQASAFAQRCEFIKGSFFD
PLPPADRYLLCNVLFNWDDAQAGAILARCAQAGPVAGVVVAERLIDPDAEVELVAAQDLRLLAVCGGRQRGTAEFEALGAAHGLALTSVTLTASGMSLLRFDVCRAGSAGGEVV
EKS

LcnA
MTKGLDKMLLTKKKKDSMGLLNEIDVTTLDEQLGGKMSKAWCRSMVVSCVYNLVDFSSSSDGKKTCALYRKYC

LcnG
MDGTNKRLEDKWFDINFLEMYTRSCLKTFGYFDEILIVKKRIEVLKNVLEKQYLSTNDYAEEFFELNTTLESIKEYIKLNLVIEKEPISICIMVKNEERCIKRCIDSVEILAEE
IIIIDTGSTDNTINIIEECANDKIKVFSKEWRNDFSEIRNYAIEKASSEWLVFIDADEYLDEASVLNLLSTLNIFNNHKLKDSIVLCPMINEANNTIHFRTGKFFRKDSGIKFF
GTCHEEPRIKGMPNSTLLIPIKVDYLHDGYLAKVQSNKDKKTRNIELLEGMVELEPDNPRWAYMFVRDGFAILDNEYIEKTCLRFLLLDKNVRICVNNLQDHKFTLSLLTILGR
LYLRECEFEKSNLIIRILDELIPNSLDGKFLAFMERFSKLKIEINTLLTEVIEYRRNHEVDETSLINTQGYHIDYVLSILLFETGNYAQSKKYFDFLQENHFLEELFQDSSYSI
ILKMLESVED

LtnA1
MNKNEIETQPVTWLEEVSDQNFDEDVFGACSTNTFSLSDYWGNNGAWCTLTHECMAWCK

LtnA2
MKEKNMKKNDTIELQLGKYLEDDMIELAEGDESHGGTTPATPAISILSAYISTNTCPTTKCTRAC

LtnM1
MKFNKNVFPEINETDFDNNIKPLLDELESRITIPQEELSFSSINDDLFRELTRNEEYPYQSICTIVANIVMDDGSEIWRKDIFVDSNSVREAVCDILSQTLFLYFIRCFSEQIK
DIRKTDEDKESTYNRYINLLFSSNFKIFSDEYPVLWYRTIRIIKNRWYSIKKSLLLTQKHRVEIDKQLDIPHKMKIKGLKIGGDTHNGGATVTTIFFEKGYKLIYKPRSTSGEF
SYKKFIEKINPYLKKDMGAIKAIDFGEYGFSEYIECNTDEEDMKQVGQLAFFMYLLNASDMHYSNVIWTKQGPVPIDLETLFQPDRIRKGLKQSETNAYHKMEKSVYGTGIIPI
SLSVKGKKGEVDVGFSGIRDERSSSPFRVLEILDGFSSDIKIVWKKQQKSSSSKNNLIVDHKKEREILQRAQSVVEGFQETSKIFMKHREEFISIILDSFENIKIRYIHNMTFR
YEQLLRTLTDAEPAQKIELDRLLLSRTGILSISSSPYISLSECQQMWQGDVPYFYSKFSSKSIFDTNGFVDEIELTPRQAFIIKAESITNDEVDFQSKIIKLAFMARLSDPHTT
NDNKLNKKVIIESNQQSNSSESGNKAILFLSDLLKNNVLEDRYSHLPKTWIGPVARDGGLGWAPGVLGYDLYSGRTGPALALAAAGRVLKDKDSIELSADIFNKSSQILQEKTY
DFRNLFASGIGGFSGITGLFWALNAAGNILNNDDWIKTSNQSMLLLNENMLKVDKNFFDLISGNSGAIGMMYLTNPNFYLSRSKINDILLTTDCLITEMEKDETSGLAHGVSQI
LWFLSIMMQRQPSSEIKIRATIVDNIIKKKYTNSYGEIECYYPTDGHSKSTSWCNGTSGILVAYIEGYKANIVDKSSVYHIINQINVEQLQHDNIPIMCHGSLGVYESLKYASK
YFEIETKYLLDVMRNGGCSSQEVLKYYGKGNGRYPLSPGLMAGQSGALLHCCKLEDNDISVSPISLMT

LtnM2
MDPSIKKLVDSIIEFYKKDIYLAYKELEREIKNIDKTIYNTSNDEILRIFKESLISIITDDIYRLSIKTFIYEFHKFRIDNGFPAVKDSESAFNYYISTFDVKTIARWFEKFPM
LESIISSSIKNDCTFMVDVCVNFILDLSECEKINLISEDSRLITISSSNSDPHNGGTRVLFFRFHNGDTILYKPRSLTVDKLISNIFEEVFEFDATNSKNPIPKVLDRGTYGWQ
EFIEKKSISSSEIKQAYYNLGIFSSIFTVLGSTDIHDENLIFKGTTPYFIDLETALSPRIRYEGNEENLFYRMSSSLFTSIVGTTIIPAKLAVHSQEIMIGAINTPAKQKTKKD
GFNIINFGTDAVDIAKQNIEVERIANPMRIKNNIVNDPLPYQNIFTRGFKEGIKSIILKKGSIISILNNFNSPIRYIMRPTAKYYLILDAAVFPENLYSEQTLNKTLNYLKPPK
IVENSLISKQLFLAEKRILSEGDIPSFYVLGKEKNIRAQNFISEQIFEETAVDNAIQILESISQDWVNFNERLIAEGFSYIREQSRGYLSSDFENSDIFKSSLTETKKSGYTAM
LKTIISMSVKTSENKKIGWLPGIYDDYPISYMSAAFCSFHDSGGIITLLEHHFGHCSPEYNEMKRGLLELGKMLKINNSNLSIISGSESLEFLYTHREVECLELEYILNNSAEI
MGDVFLGKLGLYLILASYLKTDLKIFQDFSIICQKNLEFKKFGIAHGELGYLWTIFRIQNKLKNKNACLSIYHEVLNIYKGKRIESVGWCNGLSGILMILSEMSTVLEKNQDYL
FKLANLSTKLNEESVDLSVCHGASGVLQTLLFVYSNTNDKRYLSLANKYWKKVLDNSIKYGFYNGERDKDYLLGYFQGWSGFTDSALLLDKYNNNEQVWIPINLSSDIYQHNLN
NCKEKNYEGDGCHKS

LynD
MQSTPLLQIQPHFHVEVIEPKQVYLLGEQANHALTGQLYCQILPLLNGQYTLEQIVEKLDGEVPPEYIDYVLERLAEKGYLTEAAPELSSEVAAFWSELGIAPPVAAEALRQPV
TLTPVGNISEVTVAALTTALRDIGISVQTPTEAGSPTALNVVLTDDYLQPELAKINKQALESQQTWLLVKPVGSVLWLGPVFVPGKTGCWDCLAHRLRGNREVEASVLRQKQAQ
QQRNGQSGSVIGCLPTARATLPSTLQTGLQFAATEIAKWIVKYHVNATAPGTVFFPTLDGKIITLNHSILDLKSHILIKRSQCPTCGDPKILQHRGFEPLKLESRPKQFTSDGG
HRGTTPEQTVQKYQHLISPVTGVVTELVRITDPANPLVHTYRAGHSFGSATSLRGLRNTLKHKSSGKGKTDSQSKASGLCEAVERYSGIFQGDEPRKRATLAELGDLAIHPEQC
LCFSDGQYANRETLNEQATVAHDWIPQRFDASQAIEWTPVWSLTEQTHKYLPTALCYYHYPLPPEHRFARGDSNGNAAGNTLEEAILQGFMELVERDGVALWWYNRLRRPAVDL
GSFNEPYFVQLQQFYRENDRDLWVLDLTADLGIPAFAGVSNRKTGSSERLILGFGAHLDPTIAILRAVTEVNQIGLELDKVPDENLKSDATDWLITEKLADHPYLLPDTTQPLK
TAQDYPKRWSDDIYTDVMTCVNIAQQAGLETLVIDQTRPDIGLNVVKVTVPGMRHFWSRFGEGRLYDVPVKLGWLDEPLTEAQMNPTPMPF

McbA
MELKASEFGVVLSVDALKLSRQSPLGVGIGGGGGGGGGGGSCGGQGGGCGGCSNGCSGGNGGSGGSGSHI

McbC

MSKHELSLVEVTHYTDPEVLAIVKDFHVRGNFASLPEFAERTFVSAVPLAHLEKFENKEVLFRPGFSSVINISSSHNFSRERLPSGINFCDKNKLSIRTIEKLLVNAFSSPDPG
SVRRPYPSGGALYPIEVFLCRLSENTENWQAGTNVYHYLPLSQALEPVATCNTQSLYRSLSGGDSERLGKPHFALVYCIIFEKALFKYRYRGYRMALMETGSMYQNAVLVADQI
GLKNRVWAGYTDSYVAKTMNLDQRTVAPLIVQFFGDVNDDKCLQ

McbD

MINVYSNLMSAWPATMAMSPKLNRNMPTFSQIWDYERITPASAAGETLKSIQGAIGEYFERRHFFNEIVTGGQKTLYEMMPPSAAKAFTEAFFQISSLTRDEIITHKFKTVRAF
NLFSLEQQEIPAVIIALDNITAADDLKFYPDRDTCGCSFHGSLNDAIEGSLCEFMERQSLLLYWLQGKANTEISSEIVTGINHIDEILLALRSEGDIRIFDITLPGAPGHAVLT
LYGTKNKISRIKYSTGLSYANSLKKALCKSVVELWQSYICLHNFLIGGYTDDDIIDSYQRHFMSCNKYESFTDLCENTVLLSDDVKLTFEENITSDTNLLNYLQQISDNIFVYY
ARERVSNSLVWYTKIVSPDFFLHMNNSGAININNKIYHTGDGIKVRESKMVPFP

MdnA

MAYPNDQQGKALPFFARFLSVSKEESSIKSPSPEPTYGGTFKYPSDWEDY

MdnA*

MALPFFARFLSVSKEESSIKSPSPEPTYGGTFKYPSDWEDY

MdnC

MTVLIVTFSHDNESIPLVIKAIEAMGKKAFRFDTDRFPTEVKVDLYSGGQKGGIITDGEQKLELKEVSSVWYRRMRYGLKLPDGMDSQFREASLKECRLSIRGMIASLSGFHLD
PIAKVDHANHKQLQLQVAQQLGLLIPGTLTSNNPEAVKQFAREFEATGIVTKMLSQFAIYGDKQEEMVVFTSPVTKEDLDNLEGLQFCPMTFQENIPKALELRITIVGEQIFTA
AINSQQLDGAIYDWRKEGRALHQQWQPYDLPKTIEKQLLELVKYFGLNYGAIDMIVTPDERYIFLEINPVGEFFWLELYPPYFPISQAIAEILVNS

MibA

MPADILETRTSETEDLLDLDLSIGVEEITAGPA

MibD

MTAHSDAGGDPRPPERLLLGVSGSVAALNLPAYIYAFRAAGVARLAVVLTPAAEGFLPAGALRPIVDAVHTEHDQGKGHVALSRWAQHLLVLPATANLLGCAASGLAPNFLATV
LLAADCPITFVPAMNPVMWRKPAVRRNVATLRADGHHVVDPLPGAVYEAASRSIVEGLAMPRPEALVRLLGGGDDGSPAGPAGPVGRAEHVGAVEAVEAVEAVEAVEAAEALA

MibH

MARSEESNTLARLFDVLGDDAAAAREWVTEPHRLIASNERLGTAPEAPADDDPEAIRTVGVIGGGTAGYLTALALKAKRPWLDVALVESADIPIIGVGEATVSYMVMFLHHYLG
IDPAEFYQHVRPTWKLGIRFEWGSRPEGFVAPFDWGTGSVGLVGSLRETGNVNEATLQAMLMTEDRVPVYRGEGGHVSLMKYLPFAYHMDNARLVRYLTELATRRGVHHVDATV
AEVRLDGPDHVGDLITTDGRRLHYDFYVDCTGFRSLLLEKALGIPFESYASSLFTDAAITGTLAHGGHLKPYTTATTMNAGWCWTIPTPESDHLGYVFSSAAIDPDDAAAEMAR
RFPGVTREALVRFRSGRHREAWRGNVIAVGNSYAFVEPLESSGLLMIATAVQILVSLLPSSRRDPLPSNVANQALAHRWDAIRWFLSIHYRFNGRLDTPFWKEARAETDISGIE
PLLRLFSAGAPLTGRDSFARYLADGAAPLFYGLEGVDTLLLGQEVPARLLPPRESPEQWRARAAAARSLASRGLRQSEALDAYAADPCLNAELLSDSDSWAGERVAVRAGLR

MibO

MIFGPDFHRDPYPVYRRLRDEAPCHHEPALGLYALSRYEDVLAALRQPTVFSSAARAVASSAAGAGPYRGADTVSPERETAAEGPARSLLFLDPPEHQVLRQAVSRGFTPQAVL
RLEPAVRDIAAGLADRIPDRGGGEFVTEFAAPLAIAVILRLLGVPEADRARVSELLSASALSGAEAELRSYWLGLSALLRDREDAGEGDGEDRGVVAALVRPDAGLRDADVAAG
PAVRAPLTDEQVAAFCALVGQAGTESVAMALSNALVLFGRHHDQWRTLCARPDAIPAAFEEVLRYWAPTQHQGRTLTAAVRLHGRLLPAGAHVLLLTGSAGRDERAYPDPDVFD
IGRFHPDRRPSTALGFGLGAHFCLGAALARLQARVALRELTRRFPRYRTDEERTVRSEVMNGFGHSRVPFST

MibS

MTTGTTVAHAVEPDGFRAVMATLPAAVAIVTAAAADGRPWGMTCSSVCSVTLTPPTLLVCLRTASPTLAAVVSGRAFSVNLLCARAYPVAELFASAAADRFDRVRWRRPPGTGG
PHLADDARAVLDCRLSESAEVGDHVVVFGQVRAIRRLSDEPPLMYGYRRYAPWPADRGPGAAGG

PaaA

MSLTNVKPLIKESHHIILADDGDICIGEIPGVSQVINDPPSWVRPALAKMDGKRTVPRIFKELVSEGVQIESEHLEGLVAGLAERKLLQDNSFFSKVLSGEEVERYNRQILQFS
LIDADNQHPFVYQERLKQSKVAIFGMGGWGTWCALQLAMSGIGTLRLIDGDDVELSNINRQVLYRTDDVGKNKVDAAKDTILAYNENVHVETFFEFASPDRARLEELVGDSTFI
ILAWAALGYYRKDTAEEIIHSIAKDKAIPVIELGGDPLEISVGPIYLNDGVHSGFDEVKNSVKDKYYDSNSDIRKFQEARLKHSFIDGDRKVNAWQSAPSLSIMAGIVTDQVVK
TITGYDKPHLVGKKFILSLQDFRSREEEIFK

PaaP

MIKFSTLSQRISAITEENAMYTKGQVIVLS

PadeA

MKKQYSKPSLEVLDVHQTMAGPGTSTPDAFQPDPDEDVHYDS

PadeK

MTERAAVRTDHYKAFGFRIESDFVLPELPPAGEREPLDNITVRRTDLQPLWNSSIHFYGNFAILDHGRTVMFRVPGAAIYAVQDASSILVSPFDQAEENWVRLFILGTCIGIIL
LQRKIMPLHGSAVAIDGKAYAIIGESGAGKSTLALHLVSKGYPLLSDDVIPVVMTQGSPWVVPSYPQQKLWVDTLKHMGMDNANYTPLYERKTKFAVPVGSNFHEEPLPLASIF
ELVPWDAATHIAPIQGMERFRVLFHHTYRNFLVQPLGLMEWHFKTLSSFVHQIGMYRLHRPMVGFSTLDLTSHILNITRQGENDQ

PalA

MKDLLKELMYEVDLEEMENLQGSGYSAAQCAWMALSCVNYIPGVGFGCGGYSACELYKRYC

PalS

MGNLRDFYQLMKDNYADSNLFKDLNLIHNISNDIQIGINCDFSEMLGELVGNYDSLNYPSITCGILTYNEERCIKRCLESVVNEFDEIIVLDSVSEDNTVKIIKENFNDVKVYV
EPWKNDFSFHRNKIINLATCDWIYFIDADNYYDSKNKGKAMRIAKVMDFLKIEGVVSPTVIEHDNSMSRDTRKMFRLKDNILFSGKVHEEPVYANGEIPRNIIVDINVFHDGYN
PKIINMMEKNERNITLTKEMMKIEPNNPKWLYFYSRELYQTQRDIALVQSVLFKALELYENSSYTRYYVDTIALLCRVLFESKNYQKLTECLNILENNTLNCSDIDYYNSALLF
YNLLLRIKKISSTLKENIDMYERDYHSFINPSHDHIKILILNMLLLLGDYQDAFKVYKEIKSIEIKDEFLVNVNKFKDNLLSFIDSINKI

PapA

MLKQINVIAGVKEPIRAYGCSANDACYFCDTRDNCKACDASDFCIKSDT

PapA_tev

LKQINVIAGVKEPIRAYENLYFQGCSANDACYFCDTRDNCKACDASDFCIKSDT

PapB

MANLIQDREDELIHFHPYKLFEVDSKTFFYNVVTNAIFEIDSLIIDILHSKGKNEEHVVKDLAERYELSQVREAIQNMKEAYIIATDANISDVEKMGILDNSQRVFKLSSLTLF
MVQECNLRCTYCYGEEGEYNQKGKMTSEIARSAVDFLIQQSGEIEQLNITFFGGEPLLNFPLIQETVQYVHEQSEIHNKKFSFSITTNGTLITPKIKNFFYKHHFAVQTSIDGD
EKTHNFNRFFKGGQGSYDLLLKRTEEMRNDRKIGARGTVTPAELDLSKSFDHLVKLGFRKIYLSPALYSLSDDHYDTLSKEMVKLVEQFRELLEREDYVTAKKMSNVLGMLSKI
HSGGPRIHFCGAGTNAAAVDVRGNLFPCHRFVGEDECSIGNLFDEDPLSKQYNFIENSTVRNRTTCSKCWAKNLCGGGCHQENFAENGNVNQPVGKLCKVTKNFINATINLYLQ
LTQEQRSILFG

PapoA

SKKEWQEPTIEVLDINQTMAGKGWKQIDWVSDHDADLHNPS

PapoK

MHDRSANVSWTKYIAFGLRIASELNLPELILAAPEAVEDVVIRQADLTAWSGQLEQANFVMLDERFMFQIPGTAIYAVREGKEIEVSIFSGADPDTVRLFVLGTCMGVLLMQRR
ILPIHGSAVVIGGRAYAFVGESGTGKSTLAAAFRQAGYQMVSDDVIAVKATASSAIVYPAYPQQKLGLDSLLQLEALRENKHARKRNNIRSLTDGNSVMPQYSDLRMLAGELNK
YAVPAVDEFFNDPLPLGGVFELVADSPIRALMREGELVAVTEQPLNVLECLHTLLQHTYRRVIIPRMGLSEWSFDTAARMARKVEGWRLLRDSSVFTASEVVQRVLDIIRKEEK
SYGSH

PbtA

MNLNDLPMDVFEMADSGMEVESLTAGHGMPEVGASCNCVCGFCCSCSPSA

PbtM1

MLSSALEVDIDEAAVAADLRELAAALDRSGYGEILTCFLPQKAQAHIWAQTAAKIDGPLRTLMELFLLGRAVPQDDLPPRIAAVIPGLVSAGLVKTGQGAVWLPNLILLRPMGQ
WLWCQRPHPSPTMYFGDDSLALVHRMVTYRGGRALDLCAGPGVQALTAALRSEHVTAVEINPVAAALCRTNIAMNGLSDRMEVRLGSLYDVVRGEVFDDIVSNPPLLPVPEDVQ
FAFVGDGGRDGFDISWTILDGLPEHLSDRGACRIVGCVLSDGYVPVVMEGLGEWAAKHDFDVLLTVTAHVEAHKDSSFLRSMSLMSSAISGRPAEELQERYAADYAELGGSHVA
FYELCARRGGGSARLADVSATKRSAEVWFV

PbtO

MTQYPLSRPEPLGVHPDYRRLRETCPVARVGSPYGPAWLVTRYADVAAVLTDARFSRAAAPEDDGGILLNTDPPEHDRLRKLIVAHTGTARVERLRPRAEEIAVALARRIPGEG
EFISAFAEPFSHRVLSLFVGHLVGLPAQDLGPLATVVTLAPVPDRERGAAFAELCRRLGRQVDRETLAVVLNVVFGGHAAVVAALGYCLLAALDAPLPRLAGDPEGIAELVEET
LRLAPPGDRTLLRRTTEPVELGGRTLPAGALVIPSIAAANRDPDRPVGRRMPRHLAFGRGAHACLGMALARMELQAALKALAEHAPDVRLPAGTGALVRTHEELSVSPLAGIPI
QR

PcpA

MSSNILEKVKEFFVRLVKDDAFQSQLQNNSIDEVRNILQEAGYIFSKEEFETATIELLDLKERDEFHELTEEELVTAVGGVTGGSGIYGPIQAMYGAVVGDPKPGKDWGWRFPS
PLPKPSPIPSPWKPPVDVQPMYGVVVSNDS

PcpX	MTYRRTSYAVWEITLKCNLACSHCGSRAGHTRAKELSTQEALDLVRQMADVGIIEVTLIGGEAFLRPDWLQIAEAITKAGMLCSMTTGGYGISLETARKMKAAGIASVSVSIDG<br>LEETHDRLRGRKGSWQAAFKTMSHLREVGIFFGCNTQINRLSAPEFPLIYERIRDAGARAWQIQLTVPMGRAADNANILLQPYELLDLYPMIARVARRARQEGVQIQPGNNIGY<br>YGPYERLLRGRGSDSEWAFWQGCAAGLSTLGIEADGAIKGCPSLPTSAYTGGNIREHSLREIVEESEQLRFNLGAGTSQGTAHLWGFCQTCEFSELCRGGCTWTAHVFFNRRGN<br>NPYCHHRALFQAEQGIRERVVPKVEAQGLPFDNGEFELIEEPIDAPLPENDPLHFTSDLVQWSASWQEESESIGAVVD
PcpY	MVENIDNEREKSANEIEPESLLLPRQAWQSQIAYLKAILKAKQALDRIEKRYLR
Pgm2	MEREIVWTEIEESDLAAVVSASNVKDGPTVSSSNVKDR
PlpA1	MSIENAKSFYERVSTDKQFRTQLENTASAEERQKIIQAAGFEFTNQEWEIAKEQILATSESNNGELSEAELTAVSGGVDLSIFELLDEEPLFPIRPLYGLPI
PlpA2	MSIESAKAFYQRMTDDASFRTPFEAELSKEERQQLIKDSGYDFTAEEWQQAMTEIQAARSNEELNEEELEAIAGGAVAAMYGVVFPWDNEFPWPRWGG
PlpX	MTKKYRRVSYAVWEITLKCNLACSHCGSRAGQARTKELSTEEAFNLVRQLADVGIKEVTLIGGEAFMRSDWLEIAKAVTEAGMICGMTTGGFGVSLETARKMKEAGIKTVSVSI<br>DGGIPETHDRQRGKKGAWHSAFRTMSHLKEVGIYFGCNTQINRLSASEFPIIYERIRDAGARAWQIQLTVPMGNAADNADMLLQPYELLDIYPMLARVAKRAKQEGVRIQAGNN<br>IGYYGPYERLLRGSDEWTFWQGCGAGLNTLGIEADGKIKGCPSLPTAAYTGGNIRDRPLREIVEQTEELKFNLKAGTEQGTDHMWGFCKTCEFAELCRGGCSWTAHVFFDRRGN<br>NPYCHHRALKQAQKDIRERFYLKVKAKGNPFDNGEFVIIEEPFNAPLPENDLLHFNSDHIQWPENWQNSESAYALAK
PlpY	MNSNQIPNKVATAAQKSDDSSSVLPRQGWQDKQAFIKALIKAKQSLEIAEISNFLT
ProcA*	MSEEQLKAFIAKVQADTSLQEQLKVEGADVVAIAKASGFAITTEDLNSHRQNLSDDELEGVAGGFFCVQGTANRFTINVC
ProcA1.7	MSEEQLKAFIAKVQADTSLQEQLKVEGADVVAIAKASGFAITTEDLKAHQANSQKNLSDAELEGVAGGTIGGTIGGTIVSITCETCDLLVGKMC
ProcM	MESPSSWKTSWLAAIAPDEPHKFDRRLEWDELSEENFFAALNSEPASLEEDDPCFEEALQDALEALKAAWDLPLLPVDNNLNRPFVDVWWPIRCHSAESLRQSFVSDSAGLADE<br>IFDQLADSLLDRLCALGDQVLWEAFNKERTPGTMLLAHLGAAGDGSGPPVREHYERFIQSHRRNGLAPLLKEFPVLGRLIGTVLSLWFQGSVEMLQRICADRTVLQQCFAIPCG<br>HHLKTVKQGLSDPHRGGRAVAVLEFADPNSTANSSMHVVYKPKDMAVDAAYQATLADLNTHSDLSPLRTLAIHNGNGYGYMEHVVHHLCANDKELTNFYFNAGRLTALLHLLGC<br>TDCHHENLIACGDQLLLIDTETLLEADLPDHISDASSTTAQPKPSSLQKQFQRSVLRSGLLPQWMFLGESKLAIDISALGMSPPNKPERIALGWLGFNSDGMMPGRVSQPVEIP<br>TSLPVGIGEVNPFDRFLEDFCDGFSMQSEALIKLRNRWLDVNGVLAHFAGLPRRIVLRATRVYFTIQRQQLEPTALRSPLAQALKLEQLTRSFLLAESKPLHWPIFAAEVKQMQ<br>HLDIPFFTHLIDADALQLGGLEQELPGFIQTSGLAAAYERLRNLDTDEIAFQLRLIRGAVEARELHTTPESSPTLPPPATPEALMSSSAETSLEAAKRIAHRLLELAIRDSQGQ<br>VEWLGMDLGADGESFSFGPVGLSLYGGSIGIAHLLQRLQAQQVSLMDADAIQTAILQPLVGLVDQPSDDGRRRWWRDQPLGLSGCGGTLLALTLQGEQAMANSLLAAALPRFIE<br>ADQQLDLIGGCAGLIGSLVQLGTESALQLALRAGDHLIAQQNEEGAWSSSSSQPGLLGFSHGTAGYAAALAHLHAFSADERYRTAAAAALAYERARFNKDAGNWPDYRSIGRDS<br>DSDEPSFMASWCHGAPGIALGRACLWGTALWDEECTKEIGIGLQTTAAVSSVSTDHLCCGSLGLMVLLEMLSAGPWPIDNQLRSHCQDVAFQYRLQALQRCSAEPIKLRCFGTK<br>EGLLVLPGFFTGLSGMGLALLEDDPSRAVVSQLISAGLWPTE
PsnA2	MSKNENNKKQLRDLFIEDLGKVTGGKGGPYTTLAIGEEDPITTLAIGEEDPDPTTLALGEEDPTTLAIGEE
PsnA2_tev	MSKNENNKKQLRDLFIEDLGKVTGENLYFQGKGGPYTTLAIGEEDPITTLAIGEEDPDPTTLALGEEDPTTLAIGEE
PsnB	MTNLDTSIVVVGSPDDLHVQSVTEGLRARGHEPYVFDTQRFPEEMTVSLGEQGASIFVDGQQIARPAAVYLRSLYQSPGAYGVDADKAMQDNWRRTLLAFRERSTLMSAVLLRW<br>EEAGTAVYNSPRASANITKPFQLALLRDAGLPVPRSLWTNDPEAVRRFHAEVGDCIYKPVAGGARTRKLEAKDLEADRIERLSAAPVCFQELLTGDDVRVYVIDDQVICALRIV<br>TDEIDFRQAEERIEAIEISDEVKDQCVRAAKLVGLRYTGMDIKAGADGNYRVLELNASAMFRGFEGRANVDICGPLCDALIAQTKR
RaxST	MDYHFISGLPRAGSSLLAALLRQNPQLHADVTSPVARLYAAMLMGMSEEHPSNVQIDDAQRVRLLRAVFDAYYQNRQELGTVFDTNRAWCSRLTGLARLFPRSRMICCVRDVGW<br>IVDSFERLAQSQPLRLSALFGYDPEDSVSMHADLLTAPRGVVGYALDGLRQAFYGDHADRLLLLRYDTLAQRPAQAMEQVYAFLQLPAFAHDYAGVQAEAERFDAALQMPGLHR<br>VRRGVHYVPRRSVLPPALFDQLQELAFWESAPSHGALLV
RaxX	MNHSKKSPAKGAASLQRPAGAKGRPEPLDQRLWKHVGGGDYPPPGANPKHDPPPRNPGHH
SboA	MKKAVIVENKGCATCSIGAACLVDGPIPDFEIAGATGLFGLWG
SgbA	MENQDLELLARLHALPETEPVGVDGLPYGETCECVGLLTLLNTVCIGISCA
SgbL	MTSHATEVEWEDLLRQALHATGTGARWAVEADEMWCRVAPVPGTRREQGWKLHVSATTASAPEVLTRALGVLLREKSGFKFARSLEQVSALNSRATPRGSSGKFITVYPRSDAE<br>AVALARDLHAATAGLAGPRILSDQPYAAHSLVHYRYGAFVGRRRLSDDGLLVWFIEDPDGNPVEDKRTGRYAPPPWAVCPFPASVPVAPHDGEATSRPVVLGGRFAVREAIRQT<br>NKGGVYRGSDTRTGTGVVIKEARPHVEGDASGGDVRDWLRAEARTLEKLKGTGLAPEAVALFEHAGHLFLAQDEVPGVTLRTWVAEHFRDVGGERYRADALAQVARLVDLVAAA<br>HARGLVLRDFTPGNVMVRPDGELRLIDLELAVLEDEAALPTHVGTPGFSAPERLADAPVRPTADYYSLGATACFVLAGKVPNLLPEEPVGRPSEERLAAWLTACTRPLRLPDGV<br>VDMILGLMRDDPAERWDPSRAREALRKADPTARPGDADRTAVRRTGSSAVAGPVPDSRTADGRTADGRSADEVVAGLVDHLVDSMTPADDRLWPVSTLTGESDPCTVQQGAAGV<br>LAVLTRYFELTGDPRLPGLLSTAGRWIADRTDVRSPRPGLHFGGRGTAWALYDAGRAVDDRRLVEHALDLALAPPQATPHHDVTHGTAGSGLAALHLWQRTGDTRFADLAVEAA<br>DRLTAAARREPSGVGWAVPAEADSPEGGKRYLGFAHGAAGIGCFLLAAAELSRQPDHRATALEVGEGLVADAVRIGEAAQWPAQSGDLPTAPYWCHGAAGIGTFLVRLWQATGD<br>DRFGDLARGSAHAVAERASRAPLAQCHGLAGNGDFLLDLADATGDPVHRDTAEELAGLILAEGTRRQGHVVFPNEYGEVSSSWSDGSAGILAFLLRTRHTGPRHWMVEQRG
StspA	MKKFYEAPALIERGAFAAATAGFGRLLADQLVGRLIP
StspM	MADHIAAGHDTVLSLAERTGTDPDLLGRVLRFLACRGVFAEPRPGTYALTPLSLTLLEGHPSGLREWLDASGAGARMDAAVGDLLGALRSGEPSYPRLHGRPFYEDLALHSRGP<br>AFDGLRHTHAESYVADLLAAYPWERVRRVVDVGGGTGVLVEALMRTHATLRTVLVDLPGAVATATARIAAAGFGNRYTPVTGSFFDPLPAGADVYTLVNVVHNWNDERASALLR<br>RCADAGRRDSTFVIVERLADDADPRAITAMDLRMFLFLGGKERTAAQIREVASAAGMAHQSTIKTPSGLHLLVFRKKRFAARGHGRRMVT
TbtA	MDLNDLPMDVFELADSGVAVESLTAGHGMTEVGASCNCFCYICCSCSSA
TfxA	MDNKVAKNVEVKKGSIKATFKAAVLKSKTKVDIGGSRQGCVA
TgnA*	MYRPYIAKYVEEQTLQNSTNLVYDDITQISFINKEKNVKKINLGPDTTIVTETIENADPDEYFL
TgnB	MKTILIITNTLDLTVDYIINRYNHTAKFFRLNTDRFFDYDINITNSGTSIRNRKSNLIINIQEIHSLYYRKITLPNLDGYESKYWTLMQREMMSIVEGIAETAGNFALTRPSVL<br>RKADNKIVQMKLAEEIGFILPQSLITNSNQAAASFCNKNNTSIVKPLSTGRILGKNKIGIIQTNLVETHENIQGLELSPAYFQDYIPKDTEIRLTIVGNKLFGANIKSTNQVDW<br>RKNDALLEYKPANIPDKIAKMCLEMMEKLEINFAAFDFIIRNGDYIFLELNANGQWLWLEDILKFDISNTIINYLLGEPI
ThcoA	MRKKEWQTPELEVLDVRLTAAGPGKAKPDAVQPDEDEIVHYS
ThcoK	MTRTNTGYRYRAFGLRIDSDIPLPELGDGTRPDGDADLTVVRCGEAEPEWAEGGGGGRLYAAEGIVSFRVPQTAAFRITNGNRIEVHAYSGADEDRIRLYVLGTCMGALLLQRR<br>ILPLHGSVVARDGRAYAIVGESGAGKSTMSAALLERGFRLVTDDVAAIVFDERGTPLVMPAYPQQKLWQDSLDRLQIAGSGLRPLFERETKYAVPADGAFWPEPVPLVHIYELV<br>HSDGQTPELQPIAKLERCYTLYRHTFRRSLIVPSGLSAWHFETAVKLAEKTGMYRLMRPAKVFAARESARLIETHADGEVSR

TruD	MQPTALQIKPHFHVEIIEPKQVYLLGEQGNHALTGQLYCQILPFLNGEYTREQIVEKLDGQVPEEYIDFVLSRLVEKGYLTEVAPELSLEVAAFWSELGIAPSVVAEGLKQPVT<br>VTTAGKGIREGIVANLAAALEEAGIQVSDPRDPKAPKAGDSTAQLQVVLTDDYLQPELAAINKEALERQQPWLLVKPVGSILWLGPLFVPGETGCWHCLAQRLQGNREVEASVL<br>QQKRALQERNGQNKNGAVSCLPTARATLPSTLQTGLQWAATEIAKWMVKRHLNAIAPGTARFPTLAGKIFTFNQTTLELKAHPLSRRPQCPTCGDRETLQRRGFEPLKLESRPK<br>HFTSDGGHRAMTPEQTVQKYQHLIGPITGVVTELVRISDPANPLVHTYRAGHSFGSATSLRGLRNVLRHKSSGKGKTDSQSRASGLCEAIERYSGIFQGDEPRKRATLAELGDL<br>AIHPEQCLHFSDRQYDNRESSNERATVTHDWIPQRFDASKAHDWTPVWSLTEQTHKYLPTALCYYRYPFPPEHRFCRSDSNGNAAGNTLEEAILQGFMELVERDSVCLWWYNRV<br>SRPAVDLSSFDEPYFLQLQQFYQTQNRDLWVLDLTADLGIPAFVGVSNRKAGSSERIILGFGAHLDPTVAILRALTEVNQIGLELDKVSDESLKNDATDWLVNATLAASPYLVA<br>DASQPLKTAKDYPRRWSDDIYTDVMTCVEIAKQAGLETLVLDQTRPDIGLNVVKVIVPGMRFWSRFGSGRLYDVPVKLGWREQPLAEAQMNPTPMPF
TruE*	MNKKNILPQLGQPVIRLTAGQLSSQLAELSEEALGGVDASYAVFWPICSYDD
TruE	MNKKNILPQLGQPVIRLTAGQLSSQLAELSEEALGGVDASTLPVPTLCSYDGVDASTVPTLCSYDD
