# Supplementary material for: Functional expression of diverse post-translational peptide-modifying enzymes in Escherichia coli under uniform expression and purification conditions
Source: PLoS One. 2022 Sep 19;17(9):e0266488. doi: 10.1371/journal.pone.0266488 (PMC9484694; doi:10.1371/journal.pone.0266488)
Supplement: S7 Table — (PDF) [file pone.0266488.s018.pdf]

S7 Table: Genetic Parts

Promoters

Name	Sequence	Source
P_{CymRC}	AACAAACAGACAATCTGGTCTGTTTGTATTATGGAAAATTTTTCTGTATAATAGATTCAACAAACAGACAATCTGGTCTGTTTGTATTAT	[41]
P_{LacI}	GCGGCGCGCCATCGAATGGCGCAAAACCTTTCGCGGTATGGCATGATAGCGCCC	[41]
P_{LacIQ}	GCGGCGCGCCATCGAATGGTGCAAAACCTTTCGCGGTATGGCATGATAGCGCCC	[41]
P_{LuxB}	ACCTGTAGGATCGTACAGGTTTACGCAAGAAAATGGTTTGTTACAGTCGAATAAA	[41]
P_{T5LacO}	AATCATAAAAAATTTATTTGCTTTGTGAGCGGATAACAATTATAATAGATTCAATTGTGAGCGGATAACAATT	[41]
P_{T7A1}	ATCCCGAAAATTTATCAAAAAGAGTATTGACTTAAAGTCTAACCTATAGGATACTTACAGCCATCGAGAGCTGCG	[41]

Ribosom binding sites (RBSs)

Name	Gene	Sequence	Source
lac1	LacI	GGAAGAGAGTCAATTCAGGGTGGTGAAT	[41]
lux1	LuxR	GGAAGAGAGTCAATTCAGGGTGGTGAAT	[41]
PP_1	peptide	ACCCAACACCACCAGCAAGCCTAAGGAGGAGAAAT	RBS Calc
PP_2	MBP-TruE*	TTCCACCATCAAAACACGGAGAGTAGCCCAC	RBS Calc
ME_1	AlbA	AGAATCAAGCAAGTCAAAGGAGTTAACCCGA	RBS Calc
ME_2	AlbsB[b]	AGAGTTTAGGAGAAAGACATAAGGAAATATTAA	RBS Calc
ME_3	AlbsC[b]	GGAAGCAGCCGTAAAAGGTAGGTTTTTTTT	RBS Calc
ME_4	AlbsT	AGACGCTTGAACCAGCAATAAGGAGAGTAATT	RBS Calc
ME_5	AMdnC	AGAGGCTATATAGGATAGGGGGGTCCCC	RBS Calc
ME_6	AtxB[b]	AGAGCTGTTAGTCGCTGCCAGGAGGTCCCGT	RBS Calc
ME_7	AtxC[b]	CTTTTAACATCCCTTCTCATAAGGAGGTTTTA	RBS Calc
ME_8	BamB	GCCCCGTCAGACACCTTCTAAGGAGGACATAT	RBS Calc
ME_9	BsjM	AGAGACGGGCGGCCACCAGGAGGAACGAGA	RBS Calc
ME_10	CapB[b]	AGAGGCCTACAGATATTCCAGACTAACACTAAGGAGGAAAACG	RBS Calc
ME_11	CapC[b]	TGGCTTCCGTTTTTCACCACTTGTTAAGGAGTACTTT	RBS Calc
ME_12	CinX	AGAAATTTTTCATACCGAGGGAGGAAAAT	RBS Calc
ME_13	Cln1B[b]	AGACAGTAGTATAAAGGAGGGTTCAAGT	RBS Calc
ME_14	Cln1C[b]	TTCAATAAATTAAGGAATTTTG	RBS Calc
ME_15	Cln2B[b]	AGAACCACTATAAGGAACGATTT	RBS Calc
ME_16	Cln2C[b]	CAGTATAACTAGAACAACAAGGAGTCAGATA	RBS Calc
ME_17	Cln3B[b]	AGATCCCGATAAAGGAGGTCCTA	RBS Calc
ME_18	Cln3C[b]	TAACATAAGGAGGGTTTCTAA	RBS Calc
ME_19	ComQ	AGAGGAACGAGAAATAAGGACACAGATAT	RBS Calc
ME_20	CrnM	AGATCACCCATACCAAGTATAACGAGAACCTCC	RBS Calc
ME_21	CsegB[b]	AGATCACTGCAATAGTAAGGAGGTATATA	RBS Calc
ME_22	CsegC[b]	AGCACCGAGGGGTCAATAATAAGGAGGTAAAC	RBS Calc
ME_23	EpiD	ACTGAACTATAAGGTAGGTATATT	RBS Calc
ME_24	HalM1	CCAATCAAGGAGGTAGAAAACATA	RBS Calc
ME_25	HalM2	TAAAACCGCTCGTAAGGAGGTCTT	RBS Calc
ME_26	KgpF	AGAACGCAGACAATTTCATAGGAGGTCCCG	RBS Calc
ME_27	LasB[b]	AGACAATTCATAAGGAGGTTAAGGT	RBS Calc

ME_28	LasC[b]	CCTACTACTCTGATCCCCATAAGGAGGTTTTTT	RBS Calc
ME_29	LasD[b]	CAACCTAATCTTAGGCGAGGTCATTTTTT	RBS Calc
ME_30	LasF	AGAGCCATCAGATTTAAGGAACATAAAAA	RBS Calc
ME_31	LcnG	AGACTATCGATAATAGGAGGTAGACC	RBS Calc
ME_32	LtnM1	AGACAATTGAAGCAGGCTAGCCAGGAGTTCCAT	RBS Calc
ME_33	LtnM2	AGAATTCCACCCCCCACTAAGGAGGTTTTTT	RBS Calc
ME_34	LynD	CTAAATTCCCCCGAGGTCAATA	RBS Calc
ME_35	McbC	AGAGCTTCACCCTACAAGGAGGATATAGA	RBS Calc
ME_36	MdnC	AGACGCCCGCAACATTTTATTTTAAGGACGACCCA	RBS Calc
ME_37	MibD	AGATAACCCAATCCGTAAGGACACACGTCAAGGAGGCGATTT	RBS Calc
ME_38	MibH[b]	AGAGCACATCAGACCTAAGGAAAATATAA	RBS Calc
ME_39	MibO	AGAGTTCATCAGTTTATTAGGAAAAT	RBS Calc
ME_40	MibS[b]	ACCCTGCCATTTTTTTAGCCCAAAGAACACGGAGCATCTTT	RBS Calc
ME_41	PaaA	AGATCATTTCCAATAAGGGGGACACT	RBS Calc
ME_42	PadeK	AGACACCGAAACCTAAGGAGGGATAT	RBS Calc
ME_43	PalS	AGACCAAACAATTAGGAGGACAAAT	RBS Calc
ME_44	PapB	AGAACTAAGGAGGTTAGAGG	RBS Calc
ME_45	PapoK	TTCAATCGTTAAGGAGGTACATAA	RBS Calc
ME_47	PbtM1	AGAGGAACGGATAAGGAGGTCAATAT	RBS Calc
ME_48	PbtO	AGACGTCACTATCAAACACACTAATACCACATAAGGAGCGAACA	RBS Calc
ME_49	PcpX[b]	AGACACAGGGAGGTCTTTAT	RBS Calc
ME_50	PcpY[b]	CACAAGGGGGTAGTAGT	RBS Calc
ME_51	PlpX[b]	AGAGCCACCATTTATAAGGAGAACCTACCG	RBS Calc
ME_52	PlpY[b]	ATATAAAGTTAAGGAGTTGCAC	RBS Calc
ME_53	ProcM	AGAAATCACATTACGCATAGGGGGAGGTAGACAC	RBS Calc
ME_54	PsnB	AGACGAATATAAGGAATAAAATA	RBS Calc
ME_55	RaxST	AGAGCCTTCCACAAACTAAGGAGCACAATT	RBS Calc
ME_56	SgbL	AGAAAAACGAGGAGGTAATAG	RBS Calc
ME_57	StspM	AGAGGCGGTATTAAGGGGGCCAGAG	RBS Calc
ME_58	TgnB	AGAAATATTACAACGAGGTAAAGGC	RBS Calc
ME_59	ThcoK	AGAGCATTCCATAAGGAGAAATTTT	RBS Calc
ME_60	TruD	AGACACACTCGAATTACTCAAAGGACCTCTAGCA	RBS Calc
ME_61	TruD	AGCCACACTCGAATTACTCAAAGGACCTCTAGCA	RBS Calc

Terminators

Name	Details	Sequence	Source
B0062		CAGATAAAAAAAATCCTTAGCTTTCGCTAAGGATGATTTCT	iGEM
ECK120029600		TTCAGCCAAAAAACTTAAGACCGCCGGTCTTGTCCACTACCTTGCAGTAATGCGGTGGACAGGATCGGCGGTTTTCTTTTCTCTTCTCAA	[42]
AraC	w/ 2 SNPs	TTGGTAACGAATCAGACAATTGACGGCTCGAGGGAGTAGCATAGGGTTTGCAGAATCCCTGCTTCGTCCATTTGACAGGCACATTATGCATCGATGATAAGCTGTCAAACATGAGCA	[42]
B0053	aka His Operon Terminator	TCCGGCAAAAAAGGGCAAGGTGTCACCACCCTGCCCTTTTTCTTTAAAACCGAAAAGATTACTTCGCGTT	iGEM
L3S3P21		CCAATTATTGAAGGCCTCCCTAACGGGGGGCCTTTTTTTGTTTCTGGTCTCCC	[42]
L3S2P41		CTCGGTACCAAAAAAAAAAAAAAAGACGCTGAAAAGCGTCTTTTTTTTTTTTGGTCC	[42]

L3S3P41	g->c SNP to remove BsaI site. Predicted to not affect the hairpin by Vienna [43]	AAAAAAAAAAAAACACCCTAACGGGTGTTTTTTTTTTTTTTGGTGTCCC	[42]
IOT		TTGGTAACGAATCAGACAATTGACGGCTCGAGGGAGTAGCATAGGGTTTGCAGAATCCCTGCTTCGTCCATTTGACAGGCACATTATGCATCGATGATAAGCTGTCAAACATGAGCAGATCCTCTACGCCGGACGCATCGTGGCCGGCATCACCGGCGCCACAGGTGCGGTTGCTGGCGCCTATATCGCCGACATCACCGATGGGGAAGATCGGGCTCGCCACTTCGGGCTCATGAGCAAATATTTTATCTG	[44]

Ribozymes

Name	Details	Sequence	Source
RiboJ53		AGCGGTCAACGCATGTGCTTTGCGTTCTGATGAGACAGTGATGTCGAAACCGCCTCTACAAATAATTTTGTTTAA	[45]
ElvJ		AGCCCCATAGGGTGGTGTGTACCACCCCTGATGAGTCCAAAAGGACGAAATGGGGCCTCTACAAATAATTTTGTTTAA	[45]

Linkers/Tags

Name	Details	Sequence	Source
ATag-1	Affinity tag, previously published as pTEV5	ATGTCATATTACCACCATCACCATCATCACGACTATGATATTCCCACAAGCGAGAACTTGTACTTTCAAGGG	[46]
ATag-2	N-terminal SUMO affinity tag	ATGTCATATTACCACCATCACCATCATCACGGGTCCCTGCAG	derived from ATag-1
ATag-3	C-terminal sumo affinity tag (N-terminal to the peptide)	ATGTCATATTACCACCATCACCATCATCAC	derived from ATag-1
ATag-4	C-terminal sumo affinity tag (C-terminal to SUMO)	TCCATTACAAGCCACCATCACCATCATCACGGT	derived from ATag-1
Link-1	N-terminal SUMO linker v1	CATCACCATCACCACCATGGATATGATATTAGCACAGGT	derived from ATag-1
Link-2	N-terminal SUMO linker v2	TGCATGTCATATTACGACTCCATTCCCACAAGCGAGAACTTGTACTTTCAAGGGTGC	derived from ATag-1
Link-3	C-terminal sumo linker	CGACTGGTTCCGCGTGGTAGCTATTACGACTCCATTCCCACAAGCGAGAAC	derived from ATag-1
RST_N (with Link-1)	Concatenation of: ATag-2, SUMO, and Link-1	ATGTCATATTACCACCATCACCATCATCACGGGTCCCTGCAGGACTCAGAAGTCAATCAAGAAGCTAAGCCAGAGGTCAAGCCAGAAGTCAAGCCTGAGACTCACATCAATTTAAAGGTGTCCGATGGATCTTCAGAGATCTTCTTCAAGATCAAAAAGACCACTCCTTTAAGAAGGCTGATGGAAGCGTTCGCTAAAAGACAGGGTAAGGAAATGGACTCCTTAAGATTCTTGTACGACGGTATTAGAATTCAAGCTGATCAGGCCCCTGAAGATTTGGACATGGAGGATAACGATATTATTGAGGCTCACCGCGAACAGATTGGAGGTCATCACCATCACCACCATGGATATGATATTAGCACAGGT	
RST_N	Concatenation of: ATag-2, SUMO, and Link-2	ATGTCATATTACCACCATCACCATCATCACGGGTCCCTGCAGGACTCAGAAGTCAATCAAGAAGCTAAGCCAGAGGTCAAGCCAGAAGTCAAGCCTGAGACTCACATCAATTTAAAGGTGTCCGATGGATCTTCAGAGATCTTCTTCAAGATCAAAAAGACCACTCCTTTAAGAAGGCTGATGGAAGCGTTCGCTAAAAGACAGGGTAAGGAAATGGACTCCTTAAGATTCTTGTACGACGGTATTAGAATTCAAGCTGATCAGGCCCCTGAAGATTTGGACATGGAGGATAACGATATTATTGAGGCTCACCGCGAACAGATTGGAGGTTGCATGTCATATTACGACTCCATTCCCACAAGCGAGAACTTGTACTTTCAAGGGTGC	
RST_C	Concatenation of: ATag-3, peptide insert, Link-3, SUMO, ATag-4	ATGTCATATTACCACCATCACCATCATCAC……peptide_insert……CGACTGGTTCCGCGTGGTAGCTATTACGACTCCATTCCCACAAGCGAGAACGACTCAGAAGTCAATCAAGAAGCTAAGCCAGAGGTCAAGCCAGAAGTCAAGCCTGAGACTCACATCAATTTAAAGGTGTCCGATGGATCTTCAGAGATCTT4CTTCAAGATCAAAAAGACCACTCCTTTAAGAAGGCTGATGGAAGCGTTCGCTAAAAGACAGGGTAAGGAAATGGACTCCTTAAGATTCTTGTACGACGGTATTAGAATTCAAGCTGATCAGGCCCCTGAAGATTTGGACATGGAGGATAACGATATTATTGAGGCTCACCGCGAACAGATTGGAGGCTCCATTACAAGCCACCATCACCATCATCACGGT	

Genes

Name	Details	Sequence	Source

SUMO	sequence from pE-SUMO	GACTCAGAAGTCAATCAAGAAGCTAAGCCAGAGGTCAAGCCAGAAGTCAAGCCTGAGACTCACATCAATTTAAAG<br>GTGTCCGATGGATCTTCAGAGATCTTCTTCAAGATCAAAAAGACCACTCCTTTAAGAAGGCTGATGGAAGCGTTC<br>GCTAAAAGACAGGGTAAGGAAATGGACTCCTTAAGATTCTTGTACGACGGTATTAGAATTCAAGCTGATCAGGCC<br>CCTGAAGATTTGGACATGGAGGATAACGATATTATTGAGGCTCACCGCGAACAGATTGGAGGT	Life-Sensors
lacI		ATGAAACCAGTAACGTTATACGATGTCGCAGAGTATGCCGGTGTCTCTTATCAGACCGTTTCCCGCGTGGTGAAC<br>CAGGCCAGCCACGTTTCTGCGAAAACGCGGGAAAAAGTGGAAGCGGCGATGGCGGAGCTGAATTACATTCCCAAC<br>CGCGTGGCACAACAACTGGCGGGCAAACAGTCGTTGCTTATTGGCGTTGCCACCTCCAGTCTGGCCCTGCACGCG<br>CCGTCGCAAATTGTCGCGGCGATTAAATCTCGCGCCGATCAACTGGGTGCCAGCGTGGTGGTGTCGATGGTAGAA<br>CGAAGCGGCGTCGAAGCCTGTAAAGCGGCGGTGCACAATCTTCTCGCGCAACGCGTCAGTGGGCTGATCATTAAC<br>TATCCGCTGGATGACCAGGATGCCATTGCTGTGGAAGCTGCCTGCACTAATGTTCCGGCGTTATTTCTTGATGTC<br>TCTGACCAGACACCCATCAACAGTATTATTTTCTCCCATGAGGACGGTACGCGACTGGGCGTGGAGCATCTGGTC<br>GCATTGGGTCACCAGCAAATCGCGCTGTTAGCGGGCCCATTAAGTTCTGTCTCGGCGCGTCTGCGTCTGGCTGGC<br>TGGCATAAATATCTCACTCGCAATCAAATTCAGCCGATAGCGGAACGGGAAGGCGACTGGAGTGCCATGTCCGGT<br>TTTCAACAAACCATGCAAATGCTGAATGAGGGCATCGTTCCCACTGCGATGCTGGTTGCCAACGATCAGATGGCG<br>CTGGGCGCAATGCGCGCCATTACCGAGTCCGGGCTGCGCGTTGGTGCGGATATCTCGGTAGTGGGATACGACGAT<br>ACCGAAGATAGCTCATGTTATATCCCGCCGTTAACCACCATCAAACAGGATTTTCGCCTGCTGGGGCAAACCAGC<br>GTGGACCGCTTGCTGCAACTCTCTCAGGGCCAGGCGGTGAAGGGCAATCAGCTGTTGCCAGTCTCACTGGTGAAA<br>AGAAAAACCACCCTGGCGCCCAATACGCAAACCGCCTCTCCCCGCGCGTTGGCCGATTCATTAATGCAGCTGGCA<br>CGACAGGTTTCCCGACTGGAAAGCGGGCAG	[41]
HIS_6-MBP		ATGTCATATTACCACCATCACCATCATCACGACTATGATATTCCCACAAGCATGAAAATCGAAGAAGGTAAACTG<br>GTAATCTGGATTAACGGCGATAAAGGCTATAACGGATTGGCTGAAGTCGGTAAGAAATTCGAGAAAGATACCGGA<br>ATTAAAGTCACCGTTGAGCATCCGGATAAACTGGAAGAGAAATTCCCACAGGTTGCGGCAACTGGCGATGGCCCT<br>GACATTATCTTCTGGGCACACGACCGCTTTGGTGGCTACGCTCAATCTGGCCTGTTGGCTGAAATCACCCCGGAC<br>AAAGCGTTCCAGGACAAGCTGTATCCGTTTACCTGGGATGCCGTACGTTACAACGGCAAGCTGATTGCTTACCCG<br>ATCGCTGTTGAAGCGTTATCGCTGATTTATAACAAAGATCTGCTGCCGAACCCGCCAAAAACCTGGGAAGAGATC<br>CCGGCGCTGGATAAAGAACTGAAAGCGAAAGGTAAGAGCGCGCTGATGTTCAACCTGCAAGAACCGTACTTCACC<br>TGGCCGCTGATTGCTGCTGACGGGGGTTATGCGTTCAAGTATGAAAACGGCAAGTACGACATTAAAGACGTGGGC<br>GTGGATAACGCTGGCGCGAAAGCGGGTCTGACCTTCCTGGTTGACCTGATTAAAAACAAACACATGAATGCAGAC<br>ACCGATTACTCCATCGCAGAAGCTGCCTTTAATAAAGGCGAAACAGCGATGACCATCAACGGCCCGTGGGCATGG<br>TCCAACATCGACACCAGCAAAGTGAATTATGGTGTAACGGTACTGCCGACCTTCAAGGGTCAACCATCCAAACCG<br>TTCGTTGGCGTGCTGAGCGCAGGTATTAACGCCGCCAGTCCGAACAAAGAGCTGGCGAAAGAGTTCCTCGAAAAC<br>TATCTGCTGACTGATGAAGGTCTGGAAGCGGTTAATAAAGACAAACCGCTGGGTGCCGTAGCGCTGAAGTCTTAC<br>GAGGAAGAGTTGGCGAAAGATCCACGTATTGCCGCCACCATGGAAAACGCCCAGAAAGGTGAAATCATGCCGAAC<br>ATCCCGCAGATGTCCGCTTTCTGGTATGCCGTGCGTACTGCGGTGATCAACGCCGCCAGCGGTCGTCAGACTGTC<br>GATGAAGCCCTGAAAGACGCGCAGACTCGTATCACCAAGTCGTACTACCATCACCATCACCATCACGGCGGTAGT<br>GGCGAAAACCTGTATTTTCAGGGT	
luxR		ATGAAAAACATAAATGCCGACGACACATACAGAATAATTAATAAAATTAAAGCTTGTAGAAGCAATAATGATATT<br>AATCAATGCTTATCTGATATGACTAAAATGGTACATTGTGAATATTATTTACTCGCGATCATTTATCCTCATTCT<br>ATGGTTAAATCTGATATTTCAATCCTAGATAATTACCCTAAAAAATGGAGGCAATATTATGATGACGCTAATTTA<br>ATAAAATATGATCCTATAGTAGATTATTCTAACTCCAATCATTCACCAATTAATTGGAATATATTTGAAAACAAT<br>GCTGTAAATAAAAAATCTCCAAATGTAATTAAAGAAGCGAAAACATCAGGTCTTATCACTGGGTTTAGTTTCCCT<br>ATTCATACGGCTAACAATGGCTTCGGAATGCTTAGTTTTGCACATTCAGAAAAAGACAACTATATAGATAGTTTA<br>TTTTTACATGCGTGTATGAACATACCATTAATTGTTCCTTCTCTAGTTGATAATTATCGAAAAATAAATATAGCA<br>AATAATAAATCAAACAACGATTTAACCAAAAGAGAAAAAGAATGTTTAGCGTGGGCATGCGAAGGAAAAAGCTCT<br>TGGGATATTTCAAAAATATTAGGTTGCAGTGAGCGTACTGTCACTTTCCATTTAACCAATGCGCAAATGAAACTC<br>AATACAACAAACCGCTGCCAAAGTATTTCTAAAGCAATTTTAACAGGAGCAATTGATTGCCCATACTTTAAAAAT<br>TGATAA	
cymR		ATGAGCCCGAAACGTCGTACCCAGGCAGAACGTGCAATGGAAACCCAGGGTAAACTGATTGCAGCAGCACTGGGT<br>GTTCTGCGTGAAAAAGGTTATGCAGGTTTTCGTATTGCAGATGTTCCGGGTGCAGCCGGTGTTAGCCGTGGTGCA<br>CAGAGCCATCATTTTCCGACCAAACTGGAACTGCTGCTGGCAACCTTTGAATGGCTGTATGAGCAGATTACCGAA<br>CGTAGCCGTGCACGTCTGGCAAAACTGAAACCGGAAGATGATGTTATTCAGCAGATGCTGGATGATGCAGCAGAT<br>TTTTTTCTGGATGATGATTTTAGCATCGGCCTGGATCTGATTGTTGCAGCAGATCGTGATCCGGCACTGCGTGAA<br>GGTATTCTGCGTACCGTTGAACGTAATCGTTTTGTTGTTGAAGATATGTGGCTGGGTGTGCTGGTGAGCCGTGGT<br>CTGAGCCGTGATGATGCCGAAGATATTCTGTGGCTGATTTTTAACAGCGTTCGTGGTCTGACAGTTCGTAGCCTG<br>TGGCAGAAAGATAAAGAACGTTTTGAACGTGTGCGTAATAGCACCCTGGAAATTGCACGTGAACGTTATGCAAAA<br>TTCAAACGT	[41]

Modifying Enzymes

Name	Details	Sequence	Source
albA	Amplified from genome	ATGTTTATAGAGCAGATGTTTCCATTTATTAATGAAAGTGTAAGAGTTCACCAGCTTCCTGAGGGCGGCGTGTTA<br>GAAATCGACTACTTGCGCGATAATGTCTCCATTTCTGACTTTGAGTATTTGGATCTCAACAAAACGGCTTACGAG<br>CTCTGCATGCGCATGGATGGCCAAAAAACAGCTGAGCAGATTTTAGCTGAGCAATGTGCAGTGTATGATGAATCA<br>CCGGAAGATCATAAAGATTGGTATTACGACATGCTCAACATGCTCCAGAACAAGCAGGTTATTCAGCTTGGAAAC<br>CGGGCCAGCCGCCATACAATCACCACGAGCGGAAGCAATGAATTTCCGATGCCCCTGCACGCCACCTTTGAACTG<br>ACGCACCGCTGTAATTTGAAATGCGCCCACTGTTATTTGGAAAGCTCACCTGAAGCGCTCGGCACCGTGTCGATT<br>GAGCAATTCAAAAAAACGGCTGATATGCTGTTTGATAACGGTGTATTGACATGCGAAATCACAGGTGGAGAAATT<br>TTTGTCCATCCAAACGCCAATGAGATTCTTGACTATGTGTGTAAAAAGTTCAAAAAAGTCGCTGTCTTAACAAAC<br>GGAACACTCATGCGAAAAGAGAGCCTGGAGCTTTTGAAAACTTACAAGCAAAAAATCATCGTCGGCATTTCTCTA<br>GATAGTGTCAATTCCGAGGTCCATGACTCCTTTAGAGGGAGAAAAGGCTCTTTTGCCCAAACTTGTAAAACGATA<br>AAATTGTTGAGTGACCACGGTATATTTGTCAGAGTCGCTATGTCTGTATTCGAAAAAAACATGTGGGAAATCCAC<br>GATATGGCCCAAAAGGTTCGGGATCTCGGGGCGAAGGCGTTTTCTTACAATTGGGTTGACGATTTCGGAAGAGGC<br>AGGGATATTGTCCATCCAACGAAAGACGCCGAGCAGCACCGCAAGTTTATGGAATACGAGCAACATGTGATTGAT<br>GAGTTTAAAGATCTGATTCCGATTATTCCCTATGAGAGAAAACGCGCGGCAAATTGCGGCGCTGGCTGGAAGTCC<br>ATTGTGATCAGTCCGTTCGGCGAAGTACGTCCTTGCGCCCTCTTTCCAAAGGAATTTTCATTGGGAAATATTTTT<br>CATGATTCCTATGAAAGCATCTTTAACTCCCCTCTCGTCCATAAACTGTGGCAAGCGCAAGCGCCGCGGTTCAGC<br>GAACATTGCATGAAAGACAAATGCCCGTTCAGCGGCTATTGCGGAGGCTGTTACTTAAAAGGGCTGAACTCTAAC<br>AAATATCACCGGAAAAACATTTGCTCTTGGGCGAAAAATGAACAATTAGAAGATGTGGTCCAGCTTATT	ATCC 6633<br><br>[39]
albsB	Codon optimized	ATGCCTGAGCTTCCCCGTTTCGCGACGGCCCCTCGTCACGTGCGTGCCCTGGATTTCGGTCATGTTCTGGTCCTG<br>ATCGATTACCGTTCCAATCACGTCCAGTGCCTGCTTCCGGCAGCCGCAGCCCATTGGACAGCCACAGCGCGTACC<br>GGCCGCTTGGACACCATGCCGGCAGCGCTGGCCACCCAGTTACTGACATCGGCGTTATTAGTACCGCGGCCGACC<br>GCAACACCGTGGACGGCACCTGTAGCGGCACCACCTGCTCCACCGTCATGGGGTGGATCCGAGCATCCTGCCGGG<br>ACATCACGCCCTCGGGCACGTCATCGGCACTCAACCACGGCTGCGGCGGCGCTGGCATGTGTGCTGGCGATTAAG<br>GCAGCAGGCCCAACCCGCTATGCTATGCAGCGCTTGACCACGGTCGTGAAGGCAGCCGCTTCTACGTGCCGTCGC<br>CCGGCAACGCCAGCACAAGCGACGGCTGCTGCGCTTGCGGTCCGTCAGGCATGCTGGTACTCGCCAGCGCGTACA<br>GCCTGTCTGGAAGAATCCGCCGCGACTGTCATTTTACTCGCTACCCGGCGTTTGAGTTCGACATGGTGCCATGGA<br>GTAGCTCCCGATCCGATTCGCCTCCATGCCTGGGTGGAAACTGAGGATGGGACACCTGTAGCAGAGCCAGCCTCG<br>ACCCTTGCGTACACCCCGGCCTTAACCATTGGAGGCCACCATCAACACCAGCCT	[4]

albsC	Codon optimized	ATGATCTTTGGTGGATTTTCGACGACCCGTGAAGTTCGTCAACGCCCTGGTAATGCCGAGTTTATTGCTACGGAC<br>TCGCCTATTTGGCGCCTCGGTCGTAGTCCAGCTCGTTGCGTGGCTGCGGACCATGGACAGCGTCGCCTGGTAGTG<br>TTGGGAGAATGCGGGGCAACGGATGGCGAATTATCTCGCCTGGCGACCGCGGGGCTGCCCACGGATATTACCTGG<br>CGCTGGCCAGGCGTGTACGTGGTGGTCGAAGAACAACCGGAACGTACGGTGCTGCACACTGATCCAGCAGCTGCA<br>CTCCCGGTATACGCAACCCCTTGGCAAGGCGGCTGGGCATGGTCAACCAGCGCGCGCATCCTGGCACGTTTAACA<br>GAAGCTCCAATTGATGGTCAACGCCTGGCATGTTCAGTGCTGGCCCCGTCTGTTCCGGCTCTGAGCGGTACCCGC<br>ACATTCTTTGCGGGTATCGAACAATTGGCCCTGGGTTCGCGTATTGAACTGCCGGTGGATGGGTCCCGTCTGCGT<br>GTTACGGTACGTTGGCGCCCGGATCCAGTCCCGGGAGAACCATATCATCGCTTGCGCACAGCGTTGACCGAGGCG<br>GTCGCCCTGCGTGTCAACCGCGCACCAGACCTGTCATGCGACCTCTCGGGCGGCCTCGATTCCACGTCACTGGCA<br>GTCCTGGCGGCTGTGTGCTTACCGGAGTCCCACCATCTGAATGCTATCACGATTCATCCGGAGGGCGATGAAAGT<br>GGCGCGGACTTACGGTATGCGCGCTTGGCAGCTGCGCACCACGGGCGTATTCGCCACCACCTTCTCCCCCTTGCG<br>GCAGAACACCTGCCGTATACTGAAATTACGGCGGTGCCCCCTACCACCGAACCGGCACCTTCAACATTAACGCGT<br>GCACGCCTCGCGTGGCAGTTAGATTGGATGCGCCAGCACTTAGGCAGCCGCACCCATATGACTGGCGATGGAGGC<br>GACAGCGTACTGTTCCAACCGCCGGCACATCTGGCGGATCTCCTGCGGCATCGGCAGTGGCGTCGGACTTTGTCG<br>GAAAGTTTGGGATGGGCACGCCTTCGCCATACGTCTGTTTTACCCTTACTGCGTGGAGCAGCAACTCTTGCACGT<br>ACATCACGTCGGTCGGGCCTCCAGGATCTCGCACGCGCATTGGCGGGTGCAGGTCAGCAGGGCGATGGTCGTGGC<br>AATGTGAGCTGGTTCGCACCATTACCGCTGCCTGGCTGGGCGACCCCAACCGCTCGTCGCTTACTGCTTGATGCA<br>GCCGATGAAGCTATCTCGACCGCGGATCCGTTACCGGGACTGGATACGTCGCTGCGCGTACTGATCGATGAAATT<br>CGCGAAGTCGCCCGCACGGCAGCGGCAGATGCCGAACTGGCGGATGCTCACGGAACGACTCTGCATAACCCATTT<br>CTCGATCCGCGCACTATTGATGCAGTCCTGCGCACGCCAATCGCACATCGCCCGGCGGTCCACTCGTATAAGCCA<br>GCGCTGGGGCATGCAATGCAGGATTTGCTCCCGGGTGCAGTCGCTCGGCGCTCAACTAAAGGCTCTTTTAACGCC<br>GATCATTATGCGGGGATGCGTGCAAATCTGCCAGCATTGACAGCGCTGGCAGATGGCCACCTGGCCGACCTGGGT<br>TTGTTGGAGCCGACGCGCTTCCGCAGTCATCTTCGCCAAGCCGCCGCGGGCATTCCGATGCCGCTTGCGGCGATC<br>GAACAGGCGCTGTCTGCCGAAGCATGGTGTCATGCACATCACGCCACCCCAAGCCCTGCCTGGACAACGCAGCCA<br>CCGGAACACCCGCATGCC	[4]
albsT	Codon optimized	ATGAGCACGTCCCCCGAACAGACCCTCTGGATCTCAACTGATACCTGTGGTCTGGGGCCGTATCGCGCTGACTTG<br>GTGGATACCTATTGGCAGTGGGAACAAGACCCAACATTGCTTGTAGGCTACGGTCGTCAGTCACCGCAGTCACTG<br>GAGGCCCGCACGGAAGGTATGGCCCACCAATTGCGTGGCGATAACATCCGTTTCACTATCTATGATCTGTGCAGC<br>AGTACACCTACCCCGGCGGGCGTGGCAACGCTGCTGCCCGATCATAGCGTCCGTACTGCCGAGTATGTTATTATG<br>CTTGCGCCTGAAGCACGTGGGCGTGGCTTAGGAACCACCGCCACGCAGCTGACGTTAGATTATGCGTTTCACATC<br>ACCAATCTGCGGATGGTCTGGTTGAAAGTACTGGCGCCGAACACCGCGGGCATCCGTGCGTATGAGAAAGCTGGC<br>TTTCGTACAGTTGGAGCGCTTCGCGAAGCCGGCTATTGGCTGGGGAAGGTCTGCGATGAGGTACTGATGGATGCC<br>TTAGCGAAAGACTTCACGGGTCCAAGTGCAGTCCACGCAGCATTAACTGGCGCCAGCGGTCGCCAGCTGCGCCGT<br>GCACCT	[4]
amdnC	Codon optimized	ATGAACGTTCTGATTATAACGCATTCCCACGATAACGAGAGCATTTCATTGGTAACCCAAGCCATTGAATCCCAG<br>GGTGGTAAAGCATTTCGCTTCGATACCGATCGTTTTCCGACGGAAGTCCAGCTGGACATCTATTACTCAAATACA<br>GAGAAATGCGTGCTGGTGGCTGACGATCAAAAACTGGATTTAAATGAAGTAACCGCGGTCTGGTATCGCCGCATT<br>GCGATCGGTGGCAAAATCCCGCCCACGATGGATAAGCAACTTCGTCAGGCCTCGATTCAGGAGAGTCGTGCTACA<br>ATTCAAGGCATGATAGCGAGCATTCGCGGCTTTCACCTTGACCCAGTGCCGAACATTCGTCGCGCTGAAAATAAG<br>CAACTGCAGCTGCAGGTTGCCCGCAAAATCGGACTGGATACCCCACGCACTCTCACCACTAATAATCCGCAGGCC<br>GTGAAGGAATTTGCGGCAGAATGCCAGCAGGACGTAATCACCAAAATGCTGAGTAGTTTTGCGATTTATGATGAG<br>AAAGGCGGAGAACAGGTGGTTTTCACCAATCCCGTGAAATCTGAGGATCTGGAAAATTTAGAAGGTCTGCGCTTT<br>TGCCCTATGACGTTTCAAGAGAAAATCGCAAAGGTTCTGGAGCTCCGGATCACCATCGTGGGTAAGTCAATTTTA<br>ACGGCTGCGGTGAATTCACAGGCCCTGGACAAATCCCGTTATGATTGGCGCAAGCAGGGCGTAGCATTACTGGAT<br>GCATGGCAGACCCATACGTTACCCCAGGACGTGGCTGATAAATTGCTTCAACTGATGGCCCATTTCGGGTTAAAC<br>TATGGAGCCATTGACGTGATTCTGACCCCGGATAATCGCTATGTGTTCTTGGAGGTCAATCCGGTGGGCGAATTC<br>TTTTGGCTTGAGCGTTGCCCAGGTCTGCCGATTAGTCAAGCTATTGCTAAAGTGCTGCTTTCTCATATA	[32]
atxB	Codon optimized	ATGTACGAGCTGAATGATGGCGTAGGTTTGGCCCTCGTGGATCAGCATCCGATTTTTCTGGACCTGAAAACAGAC<br>CGTTACCTGTCGTTGAGTCCAGATGGGGCAGCAGTCCTGCTGGGAGCAGCGCCAGCCACCAAAGAGAGTCCACTG<br>TTTCTCGGATTAGAATCCATTGGCTTGGTCAAAAACGGTCCGTCAGGCCTTAAGCCTTGCCAAATTGCCGTAGCC<br>ACTGGGTCTGCACCGCCCCGTAAGGTGCAATTCGAGTCGTTGTCACTCCTGCTTTTGCGCTTAATTCGTGCACGT<br>CTGGATCAACGTGCTCTTTTGAAGCGTGTGACCGACTTAAAGAAGGCCGGCACCATTGCCCAGACGAAGAACCGT<br>GACTGCGCCTTGTCATTATTAGGTAGCGTGGAGACTGAGGCAAAGGCTTGTCGTACCCTTTTAAGTAGTACAGAC<br>AAATGCCTGCCCGACGCATTCGCAATTGCAACGCACCTGCGCCGTCGCGGAGTAGACGCCAAGTTAGTTTTCGGT<br>GTGCGCCTGCCATTCGCGGCACATGCCTGGGTCCAGGTAGATGATATTGTAGTGGGTGATCGTCCCGACCGTATC<br>CTTGCGTTCACCCCCATCTTAGTCGTT	[5]
atxC	Codon optimized	ATGCGCTATGTCGCGTCTTTCTTTGTTCGCGGACATGTCAGCACACCAGCACTGCGTCACCCAGAGCCAAAGGGT<br>TTCGCTTATGCAAAAGTCAGTGGCGGACTGAGCGTATGGAGCGATGCGCCGATTCGTCACCGTGCGCCCCTTATT<br>ACAGTGGGCGCGGTGTTCGATCGCGCGTCTTTTAAAGGGCTGGATTGCGACTTATCAGGTCTGCGTCAGGATGGT<br>CTTAATACATTGAAAGCGGAAACGTTCGGACCCTACCTGGCGTTAGAGGTTGCCGATAACGGCACCCTTCGCGTT<br>TATCGCGATCCGTCAGGCGGCGCGCCTTGCTATTACCTGCAGACCGAGGACGGCTTCTGGCTTGCAAGCGATGCT<br>GATTTGTTATTCACTCATTCGGGCGTACATCCATCAGTAAGCTTACCGGGACTGATTGAACACTTGCGTCGTCCA<br>GAGTTCCAAAATGAGGGCACATGCTTAAACGTCAAGCAAGTACGCCCTGGGGAGCAGGTTGATTTATCGCTCTCG<br>GGCGAGGTCCGTGCCTGTTTGTTCCCGCCTGCATCATCCCTGCGCCCGCCTGAGTTGCACCGCGCATACGATGAC<br>ATTAAGGCTGAGCTGCGCGCTCTGATTTTACGCAGCATTAAGGCCTATGCCAGTGATTTCCCTCACGTTGTTGTT<br>AGCTTCAGCGGTGGTCTGGATAGCAGTGTTGTTGCGGCCGGCTTAGCGCAAACTTCCACTAAGGTCCTGCTTCAC<br>ACCTTTAAGGGCCCAGATGCCAAAGGGGACGAGACTGCCTTCGCCGCAGAATGCGCGGCATATCTGGGTTTAAGC<br>TTAGAGATTGATACTCTCAGTATCGATGACGTTGATCTGTCGGCAACTATTTCCCCGCACCTGCCGCGCCCCAGC<br>ACATCATTCTTCTTGCCATCACTGCTGCGCGGTTTCTCTACCTCGAGCCAAACGCGCACAGGCGGGGCAATCTTT<br>TCGGGAAACGGCGGTGACTCGGTCTTTTGTTTCATGCATAGCGCGACCCCGCTGGCCGATTTGATGTGTCGTCCG<br>TCAGGTCTTACGCCGTTCATGCAAACATGGGCCGACGTGCAAAAGCTTACCCGTGCCTCAGCGACCGAAGTGCTG<br>CGTCGCGCGTTAAAGACAGCCATGGCGCGTGGCTACATCTGGCCTGAATCCAATCTCCTCTTGTCCCGCGACACA<br>AGCTCGAGCCGTTTAACACCTGACTCCGTTCTGTCGAGCCTTGAGGGGATTCTGCCCGGTCGCTTGCGTCACCTC<br>GCCCTGATTCGTCGTGCTCACAACACCTTCGAGCCATTCGCCCCTTGGCGTACGCCGCCAGTCGTTCACCCTCTC<br>ATGGCCAAGCCGATTCAAGCCTTCTGCCTTTCTCTTCCTTCATGGATGTGGGTCAGCGGTGGTAAAGACCGCTCG<br>CTCGTGCGTGACGCGTTCGAAGGATTACTTCCAGATTCAGTGCGCCTTCGTAAATCAAAGGGAAGTCCTGCAGGC<br>TTTCTGCATGCGCTGTACCGCGCCAAGGGTCGTCAAATGATTGAGCGTATCCGTCACGGTTACCTGCGTCGTGAG<br>GGGATCATCGATATCTCTACTGGCCCGGACGCATTGTTCTCGGAAGGGTTCCGCAATCCGCGTGTAATGCACCGT<br>TTCTTTGAGCTCGCCGCAACTGAGGTGTGGATCGATCACTGGCGCAACTGGCGCCGCCCCCGCACA	[5]
bamB	Codon optimized	ATGGAAGGGTTGTATCAGCTGAAAGTGCATAGTCGTATACACAAACTGCAAAATAATATCGCAATAGGTAGCATG<br>CCGCCTCACGCGCTGATCATCGAGGATGCCCCCGAATATTTGTCAAACGTTCTGCGCTTCTTTAGTAGCAAAAAG<br>ACTATAAAAGAAGCTGAAGTGTACCTGTCGGATAATACGAATCTGAGCTCCAATGAGATCAACCTGTTGTTAGGT<br>GATCTGATTGAGAACGAGATTATCGTAAAGCAAAACTACGACTCGAATAATCGGTACAGTCGACACAGTCTGTAT<br>TACGAGATGATTGATGCCAACGCTGAAAACGCGCAGAAAATTCTGGCAGAGAAAACAGTGGGCCTCGTTGGGATG<br>GGCGGGATTGGTTCCAATGTAGCCATGAATCTCGCAGCCGCCGGTGTTGGCAAACTGATCTTTAGTGATGGCGAT<br>ACCATAGAACTGTCTAATTTAACGCGACAGTATCTTTACAAAGAGGATCAGGTGGGCTTGAGCAAAGTAGAGAGC<br>GCCAAAGAACAACTGCAATTACTGAATAGCGAAGTCGAGCTTATCCCGGTTTGCGAAAGTATCTCTGGTGAGGAA<br>CTGTTCGACAACCATTTCTCCGAATGCGATTTCGTCGTACTGTCCGCCGACTCTCCGTTCTTTGTTCACGAATGG<br>ATTAACAATGCCGCGTTGAAATATGGCTTCTCCTACTCTAACGCAGGATATATCGAAACCTATGGCGCGATCGGT<br>CCACTGGTGATACCTGGGGAAACTGCCTGCTACGAATGCTATAAAGACAAGGGCGATCTTTACTTGTACTCCGAC<br>AACAAGGAAGAATTTTCTGTGAACCTGAATGAATCATTCCAAGCACCGAGCTATGGACCGCTTAATGCGATGGTT<br>AGTTCCATTCAGGCGAATGAAGTGATACGCCACCTCCTCGGACTTAAAACCAAAACGTCCGGCAAACGGCTGCTG	[13]

		ATCAACAGTGAAATCTACAAAATCCACGAAGAGAACTTCGAGAAGAAGAACAACTGCCTGTGCTCGGATATTAAG<br>GGCGAGAAGCTGTCGAAGAACACCCTTAACTCCGATAAAGAGCTGCACGAAGTGTATATCGAAGAACGCGAATCG<br>GATTCTTTCAACTCCATTCTCTTGGATAAAACCATGAGCAAGCTGGTAAAAATTAACAAAGAGGAGACAAAAATC<br>CTCGACATTGGTTGCGCTACCGGCGAACAGGCTCTGTATTTCGCGAATAAAGGTGCTAAGGTGACCGCTGTCGAC<br>ATTTCAGACGATATGTTGAAGGTGCTGGACAAGAAAGCAAGCAACATTAACGCGGGGAGTATCAAAACCATGCGT<br>GGTAATATCGAATCCATCGAGGTGAATGACACTTTTAATTACATCGTCTGTAACAACATCCTTGATTACCTGCCG<br>GAGATCGACCGCACGCTGAGAAAACTTAACATGTTTTTGAAAAATGACGGGACGCTGATTGTGACGATTCCCCAC<br>CCCGTGAAGGATGGTGGAGGGTGGCGGAAAGATTATTATAACGGCAAATGGAACTACGAAGAGTTTATCCTGAAG<br>GATTACTTCAACGAGGGTCTGATCGAAAAGAGCCGCGAGGACAAAAATGGGGAAACGGTGATCAAAAGCATTAAA<br>ACGTACCACAGAACCACCGAAACCTATTTCAATAGCTTTACTGACGCTGGCTTCAAGGTAGTATCTCTGCTGGAA<br>CCGCAACCGCTTTCAACTGTTTCAGAGACTCATCCAATTCTGTTCGAAAAGTGTTCGCGCATTCCGTACTTTCAA<br>GTTTTTGTGCTCAAGAAAGAGGATCGCCACGCCATT	
bsjM	Codon optimized	ATGATCAAAAATGTAAACCTCAAAGAGGCCATTAAAGGTTTGACCGTATCAGAACGTTATGACACTCTGAAAAAT<br>TCGGGAGTCAACCTGAATCTGAACATTTCGGCTTTGGAAGAGTGGCGCAACCGTAAGAATCTTTTAGCCGATGAG<br>GACTTTACGGAGATGCTGACGGTGCTGGAATATGACCCGGTGTATTTTAGCCACGCGATTAACGAGAACATCGAA<br>GAACATATCGATATCTACAAGAGCAAAATTCTGGGGGAAAACTGGTTTATCGTGCTGAACGATATTCTGGACGAG<br>CTCGATAATCCCATCGAATACAAGAAAGAGATGAATCACAGCTACCTCCTGCGTCCGTTCTTGCTCTACGCCGAA<br>AAGGAGATGAACAAATACATTGTCAATCGTAAGGAGTTACTTCCGGTGGAACCCCAGGTCATCCAACAGATCATG<br>GAAAATTTGGCCTCCAAACTGTTCGCCGTTTCTGTGAAAAGCTTTGTCCTGGAGCTGAATATTTCGAAATTGAAG<br>GACGAACTGGCCGGCGAAACACCGGACGAACGCTTTCACTCATTTATTCGTTTGATGGGTGAGAAAACGCGCCTG<br>GTGGACTTTTACAACGAATATATCGTTCTGAGTCGTATTCTGGTGAACATCACGATCTTATTCGTCAACAACATT<br>ATTGAGCTGTTTGAGCGCCTGCAGGAATCCAAGCTGGATATTGTTAAGAAACTTGGCGTGCAGGAGGAGTTCAAA<br>ATCAGTAATATTAGCATTGGCGAAGGTGATACACATCAGCAAGGACGCTCGGTTATCGTTCTTACGTTCGTGAGT<br>GGAAAGAAAGTGGTGTATAAACCAAAAAATCTGAAAGTTGTTTCTGCTTATAATTCTTTAATTGACTGGATCAAC<br>AATAAAAATAATATTCTGAAAATGCCTTCGTATAACACATTGATTTATGATGATTTCGTGATCGAGGAGTTTGTC<br>GAGAAACGTGACTGCAAAAGTATCGAGGAGGTCAAAAAATATTATATTCGTTATGGGCAAATTTTGGGGATTATG<br>TATATCTTAAATGGGAACGATTTTCATATGGAAAACCTGATTGCCTCGGGTGAATATCCGATCATTGTTGACTTG<br>GAAACGCTGCTTCAGAACATTATCAATTTTAAAAACAAACCATCAGCGGACTTGATCACCACCAAAAAGATGCTT<br>AACCTGGTAAACAGTACTCTGCTGCTCCCTGAAAAACTTCTGAAGGGCGACATCACGGACGAAGGAATCGACATG<br>TCAGCCTTGGCAGGGAAAGAACAACACTTGGAACGCCGCGAATACCAGTTGAAAAACCTGTTCACCGACAACATG<br>GTTTTTGATCTCGAAAAAGTGAAAATCGAAGGTGCGAACAACATCCCGAAATTAAACGGTGAAAACGTTGACTAC<br>AGCACCTATATTGATGAGATTGTGGTTGGGTTCGAAAATATCTGTAACCTGTTCATTCAATATCGCGACGAGTTA<br>CTGCATTCCGGCATCCTGGAGGAGTTTAAAGATGTGAAGGTTCGTCATGTGCTTCGCAATACGGTTGTTTATGCT<br>AAGATGCTGGCGAATACATATCATCCAGATTACCTGCGTGATTCGTTGAATCGCGAACAGGTTCTTGAAAACATT<br>TGGGTGCATCCGTTTGAGCGCAAAGAATTCATTAAGAGCGAGATGGAAGATATCCTCAACAACGACATCCCGATC<br>TTTTTCTCATACGCGTCGTCTAAGGATATTATCGATTCGAATGGCAAACTGCACAAAAACGTTATGGAAATTTCG<br>GGTTACGAACGTTTTACCACCAAACTGAAGGAACTGAATCCCTTTCTGATTGAACAGCAGGTGAGCGTTATTAAT<br>ATTAAAACCGGCCGCTATGGGGATAAGAAATTCGAAAAAAATTATAGCGTGCGCGACGTTGCAACGGAGAAAAAA<br>GATAATCCGATTGATTTCCTGCAGGAGGCAATGAATATCGGCGATAAAATTTTGGAACATGCTATCATCTGTGAT<br>GAGACCAAAACGATTTCGTGGCTTACCATTAACAACCATCATGATAAAAATTGGGAAATTGGGCCTATTTCCGGT<br>GAATTTTATGATGGTCTGGCGGGAATTTCACTCTTCTACCACTACCTCTATAAAAAATCCCACAATGTCGAGTAT<br>AAAAAAATTCGTGATTACGCGTTCAACATGGCGAAAGTCAAAGCCCTGTCACTGAAATACGATAGTGGCTTGACC<br>GGTTACGCTTCCTTGCTGTATACGGCACACAAGATTGTTCAGGATGAACCGCGGAAGCAATACAAAGACGTGATC<br>AACGAAGTGTTCAAGTACATTGATGAGAGCAAAGTCGTGACCGCTAAGTATAACTGGTTGCATGGCACTGCCTCT<br>ATTATTCATGTGTTATTGAACCTCTACGAGGACTCTCGTGATATGGCGTACCTGACTAAATGTATTCAGTACGGC<br>AAATATTTGGTCAAGCAAATCAAAGAACACAAGGATATGCTTGCGCCTGGCTTTAGCCAGGGCATCTCTTCGGTC<br>ATTATGGTTCTGGTGCGCTTAAGTAAAAAGTGTGAAGTCGAAGAATTTCTCGAATTAGCTCTGGAATTAATGGAA<br>ATGGAACGCAACAAACTGGGAAACCTTTCTGAATCAAACTGGCTGAACGGCTTGGTGGGCATTGGCTTATCACGT<br>ATCAAACTGAAAGGACTGGATTCCAACTTACAGGTCGACAACGACATCGAACTCGTCCTGGATGGCGTCATGAAC<br>AGCTTGTACTCAAAAGATGATACTTTGAGCTGTGGTAACTCTGGCACAGTGGAATTGTTCCTGAGTCTGTTTGAA<br>CAGACGAAAAAGAAAGAGTATCTGGATATGGCGAAAGCAATCTGCGGGAAAATGATCGAAGAGAGTCGCATCTCC<br>TTTGAGTATCAGACAAAGAGTCTGCCGGGTTTAGAACTGGTGGGCCTCTACTCTGGCTTAGCCGGAATTGGTTAT<br>CAATTCTTACGTATCTCGGACGTTGAGGATATTGCGAGCATTGCTACCTTAGAT	[22]
capB	Codon optimized	ATGCAGCCAGACCTGGAGGTTGTTGATGTTCGTCGCGGCGAGTCGTTCAAGGCATGGTCGCATGGGTACCCATAT<br>CGCACTGTTCGCTGGCACTTCCATCCTGAGTTTGAAGTACATCTGATCGTGGAAACCACCGGCCAGATGTTTGTG<br>GGTGATTATGTCGGAGGCTTTGGTCCGGGTAATCTGGTCCTGATGGGTCCCAATCTGCCTCATAATTGGGTGTCT<br>GACGTTCCTGAGGGTAAAACCGTTGCAGAGCGTAACCTTGTTGTTCAATTTGGGCAAGCGTTCGTTTCCCGTTGC<br>GAGGATTCCTTAACGGAGTGGCGTCACGTGGAAACGTTACTGGCGGATGCGCGGCGTGGCGTGCAATTTGGGCCG<br>CGCACCTCTGAGGCCATTAAACCTCTGTTCGCGGAACTGATTCACGCGCGCGGCCTGCGTCGCATTGTGCTGTTT<br>CTGTCTATGCTGCAAATCCTCGTCGATGCAACGGATCGCGAACTGCTGGCATCTCCAGCTTATCAGGCGGATCCT<br>TCGACATTTGCAAGCACGCGCATTAATCATGCGCTGGCCTACATTGGAAAGAATCTGGCGAACGAGCTTCGTGAA<br>ACAGATTTAGCACGGCTGGCCGGACAGTCTGTTTCCGCCTTCTCTCATTATTTTCGTCGTCATACCGGCCTGCCT<br>TTCGTGCAGTACGTTAATCGCATGCGTATCAACCTGGCCTGTCAGCTTCTGATGGACGGGGACGCATCGGTGACA<br>GATATTTGTTTCCGTAGCGGTTTTAACAACCTGTCCAATTTTAACCGTCAGTTTCTGGCAGTGAAAGGTATGTCA<br>CCCAGTCGGTTCCGTCGCTACCAGGCTCTCAACGACGCGTCACGTGATGCGAGTGAAGCGGCTGCAAAACGCGGC<br>GCAGGTATTGCAGGTGCACCGGCAATCGTTCCAGCGGCTCAAGCACGTGGCGAGGCACGCCCAATTCCTGAAGTG<br>CTGCTTAGCGGC	[3]
capC	Codon optimized	ATGATGCTGACGGCGAGCTCCACACCGGCATCCGGTAATCCAGCTGCCCGTGCATTGCGCGCCGCTGCCTTTGCA<br>CTGGCCTTAGGCGGAGCATGCGTTGCGCATGCCGCACCTCTGCGGATTGGCATGACATTCCAAGAATTGAATAAC<br>CCGTATTTTGTGACCATGCAGAAAGCACTGAACGAAGCCGCGGCGAGCATTGGCGCGCAAGTGATTGTAACAGAC<br>GCACATCACGACGTGTCAAAACAGGTATCAGACGTTGAGGATATGCTGCAGAAGAAAATTGATATTTTACTGGTG<br>AATCCAACCGACTCCACGGGCATCCAGAGTGCGATTGTTTCCGCAAAGAAGGCTGGCGCCGTGGTCGTGGCGGTC<br>GATGCCAATGCCAATGGCCCGGTGGATTCCTTCGTAGGGTCCAAGAATTTTGATGCCGGCGCTATGTCATGCGAG<br>TACCTTGCGAAAGCGATCAACGGCGGCGGCGAAGTGGCCATTCTGGATGGCATCCCGGTCGTCCCAATCCTGGAA<br>CGTGTCCGCGGCTGCCGCGCGGCACTGGCCAAATTCCCGAATGTGAAAATTGTCGACGTTCAGAATGGAAAACAG<br>GAACGTGCGACAGCGTTAACGGTAACCGAGAATATGATCCAGGCGCACCCGAAACTGAAAGGTGTGTTTAGTGTA<br>AACGACGGCGGGTCAATGGGCGCTTTGAGCGCCATTGAAGCGAGCGGCAAAGATATCCGCCTCACGTCCGTAGAT<br>GGTGCCCCAGAGGCGGTGGCGGCGATTCAAAAGCCGAACTCCAAATTTATTGAAACAAGCGCTCAATTTCCGCGC<br>GACCAGATCCGTTTAGCGATTGGTATTGGCCTGGCCAAGAAATGGGGCGCGAACGTGCCAAAAGCGATTCCAGTC<br>GACGTGAAACTGATTGACAAAGGGAACGCGAAAACCTTTAGTTGG	[3]
cinX	Codon optimized	ATGGCTCTCAAAACCTGCGAAGAATTTCTGCGCGATGCGTTAGATCCGGATCGCTTCGGCCGCGAGATGAAGGCA<br>GTAACAGAAATTCCCGAGATCGTTAAACTCGGCCATCGTCATGGTTATGGATTTACTGCCGAAGAATTTCTGACC<br>AAAGCTATGAGTTTTGGTGCTCCGCCGGCAGGAGCAGCAGCACCTGGCGAATCAGCCAGCGTTCCTGGCCAGAAC<br>GGTTCCTCCCCCGGACACGCTGCGCGTGCAGCTATGGCTGGTCCAGAAGCAGGGGCCACCAGCTTTGCCCACTAT<br>GAATACCGTCTGGATGAGCTGCCGGAATTCGCCCCCGTTGTGGCCGAGCTTCCGAAACTGAAAGTCATGCCGCCT<br>TCCGTGGGACCTGATCGGTTTGCAGCACGCTACCGTGATGAAGATATGCGCACAATTTCAATGAGTCCGGCGGAT<br>CCGGCTTACCAGGCTTGGCACCAGGAACTGGCGGGTCGTGGTTGGCGCGATGCAGAAGATACGGCTGCTGCTCCA<br>GATGCCCCACGGCGCGATTTTCATCTGCTGAACCTCGATGAGCATGTAGATTACCCAGGTTATGAAGAATATTTT<br>GCGGCCAAGACCCGTGTCGTCGCGGCACTCGAAAACCTGTTTGGTGGTGACGTGCGTTGCTCAGGCTCTATGTGG<br>TATCCGCCGTCGAGCTATCGCTTATGGCATACAAATGCCGATCAACCGGGGTGGCGTATGTACCTGGTAGATGTA<br>GATCGCCCATTCGCGGACCCCGACCGTACCTCCTTCTTTCGCTACCTGCATCCACGTACCCGTGAAATCGTCACG<br>CTGCGCGAAAGCCCTCGTATTGTCCGTTTCTTTAAAGTCGAACAGGATCCCGAGAAGCTGTTCTGGCACTGTATC<br>GCGAACCCCACCGATCGCCATCGCTGGTCGTTTGGTTACGTTGTTCCGGAAAACTGGATGGACGCCCTCCGTCAC<br>CATGGC	[27]

cln1B	Codon optimized	ATGCCTTTATGGTTAGCGCAGGACGTCCACGCGGTCGCTCTGGACGAAGATATCGTGGTGCTGGATGCGGTGAGC<br>GACGCATACCTGTGTTTAGTTGGTGCCAGCGCTCTGATCAGCTTGGGCAGCGAGCGTTCCGTCAGTGCAGATCCG<br>GTGGCCGCTGAGACACTTCGTGAGGCTGGTCTGGTGGGTCCACATCCTAGCGGCGCCACCCGACCAATACCTCCG<br>AAGCCGACGATTGACTTACCTGATGCAGCCCGTCAGGCGCAAGGTCGTGAATTACGTGCCGCCGCGTGGGCTGGC<br>GCGGCAACCGCAATCGATTTCCGCCGGCGTTCATTTAGACAACTCCTCGCGAGAGCAGGGCAACGCCCGCCGGGT<br>CAAGCAGCTGCTCCGGCTGATGAGGTATTGGCAGCAGCCGCAGTGTTCATGCGGTTACGTCCATGGTCACCCGTT<br>GGAGGCGCGTGCCTTATGCGTTCGTATTACTTATTACGGCATTTGCGCATCCTCGGTTTCGATGCCGATTGGATC<br>ATTGGTGTGCGTACGTGGCCATTTATGGCCCATTGCTGGCTGCAGGTCGGTGCCGTCGCACTCGACGATGACGTC<br>GAGAGATTAACAGCATACACACCGATTCTGGCGGTG	[6]
cln1C	Codon optimized	ATGGGCGACTACCTGGCTCTGTACTGGCCGCGCGGCATGCCCGGTGTAGCTGCAGACGCAATGCGGGCCGCCATC<br>GAAGCTGAGGGCGCCTGGACCCTGGCGTTCGAGGCCTACCAGCTGGTAGTGTATGTCAAAGGGCCCCGAGCACCT<br>AAAGTGCGTGCCCTGCCGGATCAGGGCGGGGTGGTCATTGGGGAACTGTTTGATACTGCAGCAACCCGCGAAGGA<br>CGCGTGCAGGACTTTCCTATAGCGCTGATCAAAGACGTCGCAGCTCAGGATGCCGCACGTATTCTTGCTACCCAT<br>GCGTGGGGTCGTTATGTGGCTGTATTAAAAGCCGGTGATCGTCCGCCATGGATCTTTCGCGATCCAAGCGGGGCG<br>GTGGAATGTCTGGCGTGGGTCCGCGATGAAGTGACCATCATTAGCAGCGATGTTGCAGCGCAACGAGCTTGGTCC<br>CCTGATCGGCTGGCGATTGACTGGTCGGGACTGGGACGTGTACTGGCACGCGGAAACTTATGGGGAGAAATTTGC<br>CCGCTGGCTGGCGTCACGGCGATTGCGCCAGGTACCGCACGGTGTGATCTCGGTGATGCAGCTCTGAGCCTGTGG<br>CGCCCAGGAGATCATGCACGTCGTAGTCGTCATGATGTTTCCCCACGTGATTTGGCAAGAGTGGTGGATGCTAGC<br>GTTGCAGCCCTGGCTAGAGATCGCAGCGCTATTCTGGTCGAAATCAGCGGGGGACTGGATTCCGCTATCGTTGCC<br>ACGTCGCTGGCTCGTTGTGGAGCCCCAGTTGTTGCTGGAATTAACCATTACTGGCCCGAACCGGAGGGTGATGAA<br>CGTCGCTGGGCCCAGGACATCGCAGATCGGTGCGGTTTTCGCCTGATCGCGGGCCAACGTCAGCGGCTGTTGCTG<br>GACGAGGCAAAGCTGCTGAGACATGCACAGGGCCCGCGACCTGGTCTGAATGCGCAGGACCCGGACCTCGATCAC<br>GATCTGGCGGAACAGGCTAAAGCGTTGGGTGCCGATGCACTGTTCTCAGGGCAAGGTGGCGATGGTGTGTTCTAT<br>CAAATGGCAAATGCTGCACTGGCAGCCGATATCCTCATGGGGAAACCTGCTCCTATGGGTAGAGCCGCGTCTTTA<br>GCCGCTGTGGCTCGTCGGGCACGAGCCACGGTCTGGAGTTTGTGCGGCCAGGCTATGTTTCCGTCGCGCGCATTT<br>GCCGCTGGTATGCCGCCGCCAAGTTTCTTGAGCGCCGGTTTGGCGCCGCCACCCGTGCACCCGTGGATTGCAGAC<br>CAGCGCGGTGTTTCACCGGCGAAACGTATTCAAATTCGGGGGCTGACCAATATTCAATGTGCTTTCGGCGATAGC<br>TTACGGGGCCGAGCAGCAGATCTTTTATATCCGCTTATGGCCCAACCGGTCATGGAACTGTGTCTGTCTATCCCT<br>GCACCGCTGTTGGCAGTAGGCGCATTGGATCGCCCTTTCGCACGTGCGGCGTTCGCAGATCGATTACCTCCTCGT<br>TCACTCGTTCGACGCTCAAAAGGTGATGTTACCGTGTTTTTCAGCAAAAGCCTTGCAGCAAGCCTGCCGGCCCTT<br>CGTCCTTTCCTGCTGGACGGGCGCCTTGCAGAACAGGGTCTGATCGATCGAGCAAAACTGGAACCTCTGCTGCAC<br>CCCGAACCGATGATTTGGCGCGACTCAGTCGGCGAGGTAATGCTGGCAGCGTATCTTGAAGCCTGGGTGCGCGCA<br>TGGGAAGCCAAGTTGCGTGTTAGC	[6]
cln2B	Codon optimized	ATGACTCTGACCTGGCGCCCGGGTGTTCACGCGGTAATGGTCGAAGATGATCTGGTTCTGCTGGATGAAGCAGCG<br>GACGCTTATGTCTGTTTGTTGGATGGCGCCAAAGTGGTTAGCGTCCGGGCTGACGGTGCTCTGAGCTTCAATCCC<br>CCACATGCAGCAGAAGATATGATCGCGGGTGGCCTCGTCGAACCTTCATCAAGTGCCGCGGCGTCAGCAAACCCG<br>CCGGCAAAACTCCCATGTACTCCGCTGGCGCGCTTATCGCGCCCGCGGCATGTAAAAGTGCGTCCGGCTGAAGCG<br>GCCTTGTTCCTGATCCAAGCCTGGGGTGTTGCGCGTGCGGTACGTCGTTGGCCAATGGCTAGATTATTAGAAGCA<br>TTACGTGGAGATCGTGCCGCAGAACCGGCGAAAGGCCGCCGATCGATGGCGGAGGCGTGCGCTGTTTTTGATGCG<br>CTTCTGGCCTGGAGCCCTTTTGACGGTGAATGTTTGTTTCGCTCAGTATTACGACGTAGATTTTTAATGGCACTG<br>GGCCATTCGCCGGACTTGGTGATAGGCGTGCGTACCTGGCCGTTCCGCGCACATTGCTGGCTGCAGAGCGGAGTG<br>GATGCCCTGGATGATTGGCCGGAACGGCTCTGCGCATATCGCCCGATTCTGGCAGCTTCTGCAAGCCAGGGTAGA	[6]
cln2C	Codon optimized	ATGAGTTACCTGCTGATGACCTGGCCGCCGGGGCAGCCGAGCGTAGAAGCTGATGCACTTCACGCAGCCTTTAAC<br>GGGCAGGGTGGATGGAGCCTGGTTTTGGAACGATTCTGCCTGCGCGTATACGTGCGTGGCGCGGCAGCCCCTGCA<br>GTTACCCTTACCCCGAAAGGAGGCGTGCTCATTGGTGAGATGTTTGATCGGGCTGCCACAGAAACGGGCGCCGTT<br>GCCGCTTATGATCTGAGCCGCCTGGGAGATGACGACGGTATGGCCGTAGCCCGGCGTGTGGTGGACGAAGCGTGG<br>GGGAGATATGTGTTGGTGCTGCCAGTTAAAGAACGCCGTCCAGTGGTTTTGCGAGAACCACTGGGCGCGCTGGAT<br>GCGCTGATCTGGCGCAAAGGCGATGTCTGGTGCGTGGGGGCAGACGTACCCCCGGGTCTTGAACCAAAAGATCTG<br>GGTGTGGAAGAGACTAGACTGACGCACCTGATCGCGGAACCGGATCTGGCATCTGCGAGCCTGCCCTTAACCGGC<br>GTCGCGGCAGTGATGCCAGGTACTGCGGTCGATGAAACCGGCCAGGTGCACCGTCTGTGGACCCCCGCGCGTTTT<br>GCTCGCTCCCCTCGCACTGACGCGTGGACTGCAGCCGAACGTATTCCGCTGGTTACCCGTGCGTGCATCGCGGCG<br>CTGTCTGCGAATCGAAGTGGTATTCTGTGCGAGATTTCGGGCGGCCTGGATAGCGCTATTGTTGCGACCTCTCTG<br>AAAGCGGAAGGTGCGAAGATTAGTAGCGGGATCAACTTCCATTGGCCCCAGGCTGAAGCAGATGAGCGCCCGTAC<br>GCACGCGCTGTTGCGAAAAGCGTGCGAACCCGGTTACAGGTGGTAGCGAGTCGTGTAGCGCCCGTTGACCCGGAA<br>ACGTTTGATGAGATCGTGGTCGCGCGACCAAGTTTTAATGCCATTGATCCAGTCTATGATACCGTACTGGCCCAA<br>CGTCTGATTCAGGGCGGTGAAGGAGCCCTGTTTACCGGACAAGGTGGTGACGCAGTTTTCTATCAGATGCCAGCA<br>CCACAACTTTCGTTGGATTTGTTGGCTCGTGGCCCCCGCCGCCGCGGTCTTATGGGATTATCACGCCGCACCAAC<br>CGCAGTGTCTGGTCGTTGCTGCGCATGGGCTTACGTGCACCCGTACGAGCAACCTTTCCCTACGGTGCGAGAGGT<br>GCCGATCGTCCTCCGATGCACCCGTGGCTGGAGGACGCGCGTGGTGTTGGGGCCGCGAAACGGATTCAGATCGAA<br>GCGCTGGTTGCTAACCAGGCCGTGTTTGAAGCATCTCGTCGCGGTGCGGCGGCTCATTTGGTGCACCCACTGCTG<br>TCGCAACCGCTTGTGGAGCTGTGCCTTTCAACCCCAGCGGCCGTGCTGGCGGGTGCCGAACAAGATAGAGCATTC<br>GTGCGTAGCGCTTTTCGTGCGCAACTGCCACGCCTGGTCTTAGATCGTCAAAGCAAAGGAGATCTGAGCGTTTTC<br>TTTGCTAAAGGTGTGGCGCGGAGCTTGCCGGGCTTGCGTCCGCGTCTGCTCGAAGGACGCTTAGCGGCACGTGGC<br>CTGATCGACGTGGAAGCGTTATCACAAGCGATGCAGCCAGAAGCGATGATTTGGCGTGACGGTTCGGCCGAAATC<br>CTGTGCCTTGCTGTTCTGGAATCATGGCTCCGCTCTTGGGAGGCTCGTGGTGCA	[6]
cln3B	Codon optimized	ATGCGCGTTGCAGTGCCGGATCATTTAGCGTATTGCGTAAAACAAGGTGGAGTTACGTTTCTGGACGTCCGCGGG<br>GATCGTTACTTCGGCCTGCCGCCGGTGCTGGAACACGCGTTCGTTGCCATTGCCGAGGCGGATTTTCTGCTGAAA<br>GAACCAAATTCACTTCTGGAGCCACTCGAAGCACTGGGTGTCTTAGTGCGAGGCCAAGCCCGCCGTGCCGATCTG<br>ACAATTCCGTCTGCAAATCTGTCATGGGTGGATGAGGTCAGCCCGACCCCACCACGTCTTGACCCTGCGTCACTC<br>GTCGCAACCGTCACGTCTGTTATTCGAACGCGTCTGAGCCAAAAGAGTAAGTCCTTGCAGGCTCTCTTGGAAGAG<br>GTCCGTACCCGCCGTCCGGGATCGCCGGCCCATAATTGGCAGCTGATGCGTCGTCTGACGGCTGGATTCCGTGCA<br>TCGCGTGCTTGGGCGCCGATAGAACCCATCTGCCTCCTGGACAGCTTGGCGTTACTGGATTTTCTGCATCGCCGT<br>GGCCTGTATCCGCATATTGTTTTCGGTGTGATCCGCCAACCGTTTGCCGCTCATTGTTGGGTGCAAGCTGATGAT<br>GTAGTCCTGAATGACCGGCTGGATCATGTCGGTGAATATACACCGATCCTGGTGGTC	[6]
cln3C	Codon optimized	ATGGAAGATTACGTGGTCCTCATTTGGCCGGCACTCGCTGAAGCTCCTGCACGCGACTTGATTCGTCGCCTGCCG<br>AAACTCAAAACCGTCATTGAAACTAGCGGATTGGTGGTACTGCGCCCCGAAAATGGTGCGGGTCTGCGGGTAGGC<br>GGGAACGGTGTGGTCCTGGGTAGCGTCTTTCGCACCGGCGGTGATCGCGAAACTGTTGCGGAATTTTCGGAATCG<br>GAAGCATCCGCGATCGCCACGAGTCGTGGTCAGCAGTTAGTGACAGAGTTCTGGGGTGGCTACCTGGCTGTTCTT<br>GGAGATGCTTCGCGTTCCGAAGTGATGGTCCTGCGAGATCCTTCAGGTGCAATGCCGGCTTATTGTTTAGTTCAT<br>GGCGAAGTCCAGATCATCTGCTCTCGCTTGGAGGTCCTGGAGGACGCAGGACTGGGGCAGCAGGCGCTGAACTGG<br>GACGTGGTGGCGCAATTACTGGCCTTCCCAAACCTTCGAGGTCGCTCAACGGGTCTTAAAGGCGTGGAAGAATTA<br>CTTCCCGGTTGCCGTCTGACATTTACGGGAGGACTGAAAACCGAAACGCTGACCTGGAACCCGTGGCTTTTTGCC<br>CGCCCATCTGCGCAAGCGCCTGAACGTGGAGTTGCGGCGACCGCCGTGCGTCAGGCGGTGGAAGTAAGCGTTCGA<br>AAATGGGCTGATCAGAGTTCACCGGTACTTTTGGAATTGTCAGGCGGGCTGGATAGTAGTATCATCGCCTGCTGT<br>CTGGACGAACCGCGCACCGCGGCCACCTTCGTGAACTTTGTCACACCGACGGCCGAAGGCGATGAACGAGGATAT<br>GCACGTCTGGTTGCCAAGGCAGCAGATAAACAACTGATCGAGCAGGACATCCGGGCTGACGAAGTAGATGTTACC<br>CGTCCAAGACCTGGCCGCCATCCTCGTCCGGCCAGTCAGGCGCTGTTACAGCCGCTGGAACAGGCTTGCGCTGAA<br>CTGGCACCTCAGTTGGGTGCGAGAAGTTTCTTCTCCGGTCTGGGAGGAGACAACGTGTTTTGTAGCATTGCAACC<br>GCAAGCCCGGCTGCGGATGCACTTTTGACTAGCGGTCTGGGCCGACAGTTCTGGGCCGCAATCGGGGACCTGTGT<br>GCACGTCATAACTGCACCGTATGGGCAGCCTTAAGCGCCACGCTGAAGAAACTGCTCCGCTCAGATCGTCGTCTG<br>GTGATCAAACCAAACCTGGATTTTCTGTCCTTTCGGGAGGACGCCATAGACCGTCCGGATCACCCATGGCTTGAA<br>GTGGCCGCCGATCGTCTGCCGGGGAAACGCGAACATGTCGCAAGCATTCTGTTGGCGCAAGGCTTCCTGGATCGT	[6]

		TATGAGCACGCTCAGGTTGCTGCCGTCCGCTTTCCCTTGTTAACGCAACCGGTTATGGAGGCTTGTCTGCGCGTG<br>CCGACCTGGATGGCAAACCACCAGGGTCGCAATCGGGCGGTCGCACGCGATGCCTTCTTTGATCGCTTGCCCCCG<br>AGAGTACGTGATCGGCAGACAAAAGGAGGTTTGAACGCGTTTATGGGTGTTGCGTTCGAACGCAACCGTCAGGCC<br>TTAGCTCGTCATCTGTTAGACGGGCGCCTGGTACAGCGTGGCCTGATAGATGCAGTGGCAATAAAATCGGCGCTG<br>GCCTCACCAGTCCTGGAAGGAGGAGCCATGAACCGCTTACTGTACCTGGCCGATGTCGAATCCTGGGTACGCTCA<br>TGGGAAGATGTG	
comQ	Codon optimized	ATGAAGGAAATCGTGAAACAGAATATCAGTAACAAAGACCTGTCGCAACTCCTGTGTTCCTTCATTGATTCAAAG<br>GAAACTTTCAGTTTTGCCGAGAGCGCTATACTGCATTATGTAGTATTCGGCGGTGAGAACCTGGACGTAGCTACC<br>TGGCTGGGCGCCGGAATTGAAATTCTGATCCTGAGCAGCGATATCATGGACGACCTGGAGGACGAGGATAACCAT<br>CATGCGTTGTGGATGAAAATTAACCGCAGCGAGAGCTTGAATGCGGCCCTGTCCTTATACACCGTCGGCTTAACG<br>AGCATCTATTCCCTGAACACAAATCCGTTGATATTTAAGTATGTGCTGCGCTACGTCAATGAGGCCATGCAGGGT<br>CAGCATGATGATATAACCAATAAAAGCAAAACCGAAGATGAATCGCTTGAAGTGATTCGCCTTAAATGCGGCAGC<br>CTGATCGCCCTGGCAAATGTCGCGGGCGTGCTGTTAGCCACGGGCGAGTACAATGAAACAGTTGAACGTTACTCT<br>TATTACAAAGGCATCGTGGCGCAAATTTCCGGCGACTATCACGTGCTGCTGTCAGGAAACCGGAGCGATATCGAG<br>AAAAACAAACAGACACTGATTTACCTGTATCTGAAACGCCTGTTTAACAACGCGAGCGAGGAATTGCTGTATCTG<br>TTCTCCCATAAAGATTTGTACTATAAAGCCCTGCTCGACCGTGAAAAGTTTGAAGAAAAACTGATCCAGGCCGGG<br>GTGACGCAGTACATCAGCGTTCTGCTCGAAATATATAAGCAGAAGTGCTTCTCCACCATAGAACAGCTGAACTTA<br>GATAAAGAAAAGAAAGAGCTGATCAAGGAGAGCCTGCTGTCATATAAGAAAGGCGACACCCGTTGCAAGACC	[14]
crnM	Codon optimized	ATGAATGATATCAACAAAAACAAAACTAAAACCATTAACGAAAAGATTAAAATTTTCACCAAAGAAGAGGTGATT<br>GATATCAGTTACTTTGAAGAATGGCGCAGCGTTCGTACTCTGCTTAACGAAAACTACTTTAAAATTATGCTCGAG<br>GAAATGAATATTTCCAAAAACCAATTTTCGTATGCGCTGCAACCGTTAAACGACGAGTTCAAACTGCATACTAAC<br>GTTAAAAATGAAGAATGGATCAAATGCTTTAATCGCGTCATTAACAATTTTAACTATAAAAATATTAACTATAAA<br>GTTGGTTTGTACCTGCCTATTCAGCCTTTCTCCGTTTATTTACAGGAGAAACTGAAAGAGATCCTGAAGAAGCTG<br>AACAACATTAAGATTAATGATAAAATTATCGACGCCTTTATCGAAGCTCACCTGATCGAAATGTTCGACCTCGTC<br>GGTAAAGTAATCGCCCTTAAATTTGAAGATTATAAACAGATCAACTTCCTGAAAAACACAAATAATGGCACCCGC<br>TTGGAGGAATTCTTGCGTAGCACCTTTTATTCTCGGAAGTCATTTCTGAAACTGTTTAACGAGTTTCCGGTACTC<br>GCGCGGGTTTGCACCGTACGTACGATCTATTTGATCAATAACTTTAGTGCTATCATCCAGAACATCAATAGCGAC<br>TACCTGGAAATCCAGGAATTTCTGAACGTCGATTTCCTGAACTTGACAAACATCACTCTTTCGACGGGTGATTCC<br>CACGAACAGGGTAAAAGTGTGTCCATCCTCTATTTTGATGAAAAAAAGCTGATTTATAAACCGAAAAATCTGAAG<br>ATTTCAGAAATTTTCGAGAGCTTCATCGACTGGTACACCAACGTCTCTAACCATAAGCTGCTCGACCTGAAAATC<br>CCGAAAGGAATTTTTAAAGACGATTACACTTATAACGAATTTATTGAGCCAAACTACTGCGAGAATAAGCGCGAA<br>ATTGAAAATTACTATAACCGTTATGGGTACCTGATCGCAATCTGTTATCTGTTCAACCTGAATGACCTGCATGTA<br>GAAAATGTGATCGCCCATGGCGAGTACCCGGTTATTGTTGATATTGAAACGAGCTTTCAAGTCCCTGTGCAAATG<br>GAGGACGATACTTTATATGTGAAGCTGTTGCGCGAGCTGGAATTGGAAAGCGTTTCATCGTCGTTTCTGTTACCT<br>ACCAATCTGTCGTTTGGTATGGACGATAAAGTGGACCTGTCCGCGCTGAGCGGAACCATGGTCGAGCTGAATCAG<br>CAAATTCTGGCGCCTGTCAACATTAATATGGACAACTTTCATTACGAGAAATCACCGAGCTATTTTCCAGGCGGA<br>AACAATATCCCTAAAAACAACAAATCAGTGACTGTTGATTATAAAAAATACTTGCTCAATATTGTGACTGGTTTC<br>GACGAATTTATGAAGTATACCCAAGAAAATCAGCTGGAATTTATTGAGTTCCTGAAAAAATTCTCAGATAAAAAA<br>ATCCGGGTGCTGGTGAAGGGTACGGAAAAATATGCGTCCATGATTCGCTACAGCAACCATCCGAACTACAACAAA<br>GAAATGAAATATCGCGAGCGTCTCATGATGAACTTGTGGGCGTACCCTTACAAAGACAAGCGTATTGTTAATAGC<br>GAAGTACAGGACCTGTTATTTAACGATATCCCGATCTTTTACTCCTTTCCAAATAGCCGTGACCTCATTGATAGT<br>CGCGGCTTGGTGTATAAAGATTACCTTCCTGTGACAGGACTGCAGAAAGCAATTGATCGCGTGAAAGATACCTCG<br>GTAAAAAGCTTGTTCGACCAGAAGCTGATTCTTCAGAGTAGCTTAGGTCTGTGGGATGAGATTCTCAACAAGCCG<br>GTCCAGAAAAAGGAACTGCTCTTTGAAAAGCAGAACTTTAACTATGTGAAAGAGGCGATCAATATTGCGGAATTG<br>CTGATTGGCTATTTAATCGAAACGGACGACCAGAGCACCATGCTGAGCATTGATTGTTCTGAAGATAAACACTGG<br>AAGATTGTTCCTTTAGACGAATCCCTGTATGGTGGGCTGTCCGGCATTGCATTATTTTTTCTCGATATTTATAAA<br>ATTACCAAAGATGAAAAATATTTTAATTACTATGATAAAATCATTTCCACGGCCATTAAACAATGTAAAGCGACC<br>ATCTTCTCGTCAAGCTTCACGGGTTGGCTGAGTCCCATTTATCCGTTGATTCTGGAAAAGAAATACTTTGGTACC<br>ATGAAAGATAAGAAATTCTTTGACTACACGATGGAAAAGCTGTCGAATATGACTGAAGAACAAATTAACAACATG<br>GATGGTATGGACTATATCAGTGGCAAGGCGGGTATTGTCAAACTGCTGATTAGCGCGTACCGGGAATCGAAGAAC<br>AATGAAAACATCGGACTGGCCCTGAGTAAATTCAGCAACGATCTGATTCAAAATATTGGCACCGGCAAAGTCAGT<br>GAATTACAAAACGTGGGCCTGGCGCACGGCATTTCTGGTATTATGGTCGTAGTAGCCTCACTGGACACGTTTAAA<br>AGTGAATATATTCGCGAGCAGCTGGCAATTGAATATGAGATGTTCTGTTTGCGTGAAGATTCATACAAATGGTGT<br>TGGGGCATCTCTGGAATGATTCAAGCCCGTCTCGAAATTCTGAAACTGAGCCCGGAGTGTGTGGATAAAAAAGAG<br>CTGAACTTGCTTATTAAGCGTTTTAAAAACATCTTGAATCAGATGATTAACGAAGATTCCCTTTGTCACGGCAAC<br>GGTTCGATCATTACTACGATGAAGATGATCTATATGTACACCCAAGACACCGAGTGGAACTCTCTGATTAATCTG<br>TGGTTATCAAATGTAAGTATCTATTCGACCTTACAAGGCTATAGCATTCCAAAGCTGGGCGATGTAACAATTAAG<br>GGGTTGTTTGATGGCATTTGTGGTATTGGCTGGTTATACCTGTATTCGAACTTTAGCATTGAAAACGTGCTGCTC<br>CTCGAGGTC	[20]
csegB	Codon optimized	ATGGACCTGTGGTTGAGCGCCGGGGTCTATGCTGTCATGATCGATGATGATGTAGTTTTCCTGGACGTCGCCACC<br>AATGCATACTTCTGCCTCCCAGCCGTTGGGAGCGTGTTGGCACTCGAAGGTCGTTCGCTGCGTGTGGCGGCTCGC<br>GAACTGGCAGAAGATCTTATTCAGGCAGGCTTAGCATCCGCGGCTGCGGCAATCGAACCCCCACCGAGCACACCA<br>GCCCCAGTTCGCACTGCGCGTGCGGTATTGGAAGCTCTGCCGGCGCGTGAAAGACCACGTCCACGTCTTGCCCAC<br>TGGCGTCAGGCGATTATGGCTGGCTTGGCGTCCCGTGCCGCTGAACGTCGACCATTCGCGCAGAGACTGCCGCCG<br>CCTTCAACGGGGGTTTCACCTCCGGCATCAGAAGGCCTGCTTGCCGATCTGGATGCGTTCCGTCGACTTCAGCCA<br>TGGTTGCCGTTCGACGGTGCTTGTCTGTTCCGTAGCCAAATGCTGCGCGATTATCTCCTTGCGCTGGGTCACCGC<br>GTTGACTGGATTTTCGGTGTACGTACGTGGCCGTTTGGTGCCCACTGTTGGTTGCAGGCCGGCGACCTGGTGCTG<br>GATGATGAGGCCGAACGTCTGATTGCGTATCACCCCATTATGGTACGT	[7]
csegC	Codon optimized	ATGGGGTATGCCGCATTGACTTATCCGGGTGGTTTAGCGGCAGCAGCGTTTGATGAGATGGTAGAAGCACTGATC<br>GATGCTGGATGGACCTTGGCGTTGCGTGCGTTCAGACTCGCCGTTCTCACCGATGGTCAGGCTCCAGCCGTGTCG<br>CCGCTGATGGGCAGAGGCGGCGTAGCAGGCGTTCTCATCGGCGAAGCGTTTGATCGTCGCGCCACATTAGGTGGC<br>GCGGTCGCACGTGCCGCGCTGGATGGTTTGGCTGACATCGATCCGCTGGAAGCAGGTCGCCATCTGATTGAAACC<br>GCGTGGGGCGGCTACGTGGGTATGTGGATTGGTCGGGCCGAAGCTGGTCCGACACTGCTGCGCGATCCTAGTGGC<br>GCGCTCGAAGCCTTAGCGTGGCGCCGTGACGGTGTAACCGTTATGTCAGCGCGCCCGTTGACGGGGCGCGCAGGC<br>CCAGCTGATTTAGCAATCGATTGGCCACGTATCGTGCAGATTCTGGCCGATCCCATTTCCGCGGCTCTCGGCCCG<br>CCCCCTCTGACTGGCTTAGCGACCATAGACCCGGGCGCGGCGGTTCATGGCGCGGATGGCCAAGAACGCTCAGTG<br>CTGTGGACCCCAGCTGCAGTTGTCCGTGGTGCTCGTCACCGTCCTTGGCCAAGCCGTCAGGATCTGCGTCGCACC<br>ATCGATGCGACTGTCGCGGCACTGGCCTCGGATGCGGGCCCGATTGTCTGCGAAATTTCAGGAGGTCTGGACTCG<br>GCCATAGTTGCGACTAGCCTTGCGGCGTCCGGTCTGGGTCCGCAGCTGACAGTGAATTTTTACGGTGACCAGCCT<br>GAAGCTGATGAACGCGGATACGCTCAAGCCGTCGCCGAACGTATCGGTGCGCCTCTGCGGACCCTTCGTCGAGAG<br>CCGTTCGCGTTCGATGAAACCGTGCTGGCAGCCGCTGGACAGGCCGCACGTCCGAATTTTAACGCCCTCGATCCT<br>GGATACGATGCCGGGCTCGTGGGTGCCCTGGAAGCTATCGATGCTCGTGCATTATTTACGGGCCATGGCGGTGAT<br>ACCGTGTTTTATCAAGTGGCGGCCAGTGCCTTGGCCGCAGACTTACTGGGCGGCGCACCATGTGAAGGTAGCCGC<br>CGTGCACGTTTAGAGGAAGTAGCTCGGCGGACCCGACGCTCGATTTGGAGTCTTGCATGGGAAGCGTTTTCTGGT<br>CGACCCAGCACTGTAAGCATTGAAGGTCAGTTGCTTCGACAGGAAGCAGAGAGAATTCGGCGCGTCGGCCTGACC<br>CATCCGTGGGTTGGAGGCCTGTCGTCTGTGACCCCTGCGAAACGCCAGCAAATCCGCGCGCTGGTCAGTAACCTG<br>AACGCGCATGGCGCCACTGGTCGCGCCGAACGCGCTAGAATCGTGCACCCGCTTTTAGCTCAGCCGGTGGTTGAA<br>GCCTGCCTGGCGATTCCTGCCCCTATCCTCAGTGCGGGCGAAGGAGAACGCTCATTTGCGAGAGAAGCCTTTGCA<br>GACCGTTTGCCACCGAGCATTGTGGGCCGCCGAAGCAAAGGGGAAATTAGTGTGTTTCTTAACAGATCTTTAGCA<br>GCCAGCGCCCCCTTTCTGCGTGGCTTTTTACTTGAAGGACGGCTGGCGGCTCGCGGGCTGATTGATCGTGACGAA<br>CTTGCAGCCGCGCTGGAACCGGAAGCAATCGTCTGGAAGGATGCGTCACGCGACCTGCTTACTGCGGCGGCCCTG<br>GAGGCGTGGGTCAGACATTGGGAAGCACGTATTGGCGAGGGGGAAGCAGCGGAAGGTGAGCGTGCTGCCGGTCGT<br>GGTACCGCAGCGACGGGACCGCGTACAAGCGCGCGGAAGGCGAACACCCGT	[7]

epiD	Codon optimized	ATGCACGGTAAACTGCTGATCTGCGCAACTGCTTCGATCAACGTCATCAATATCAACCATTATATTGTGGAGCTG<br>AAACAGCACTTCGATGAGGTGAATATCCTGTTTTCACCTTCCTCGAAGAACTTTATCAACACCGATGTCCTGAAG<br>CTGTTTTGCGATAATCTGTATGACGAGATCAAAGATCCGCTGCTGAACCACATCAACATAGTGGAGAACCACGAG<br>TATATCTTGGTGCTGCCTGCCAGTGCCAATACGATCAACAAAATCGCGAACGGTATATGCGATAACCTCTTGACG<br>ACCGTATGCTTAACCGGGTACCAGAAACTGTTTATCTTTCCGAATATGAACATCCGCATGTGGGGAAATCCGTTC<br>TTACAGAAAAATATTGACCTGCTTAAAAGCAACGACGTGAAGGTGTATTCCCCCGACATGAACAAATCTTTTGAG<br>ATAAGCTCAGGCCGCTACAAAAATAACATCACGATGCCGAATATCGAAAACGTGCTGAATTTTGTCCTGAACAAT<br>GAGAAACGCCCGCTGGAT	[30, 31]
halM1	Codon optimized	ATGCGCGAACTCCAAAATGCGCTTTACTTTAGCGAAGTGGTTTTTGGACCGAATCTTGAGAAGATTGTAGGAGAA<br>AAGCGCCTCAATTTTTGGCTCAAACTTATAGGTGAGGACCCGGAAAACCTGAAGGAGTTTCTCTCGAGAAAGGGC<br>AATTCTTTCGAAGAACAAACCTTACCGGAAAAGGAAGCTATCGTTCCGAACCGCTTAGGTGAAGAGGCGCTGGAA<br>AAAGTCCGCGAAGAACTTGAGTTCCTCAATACTTACAGCACTAAACATGTGCGTCGCGTTAAAGAGTTGGGAGTG<br>CAGATCCCTTTCGAAGGGATTCTGCTGCCATTCATTAGCATGTATATCGAAAAATTTCAGCAGCAGCAACTTCGC<br>AAAAAGATAGGGCCGATTCACGAAGAGATCTGGACGCAGATTGTTCAAGATATCACCTCCAAATTAAATGCGATT<br>CTGCACCGTACCCTGATCCTGGAACTGAATGTAGCTCGTGTTACCTCCCAACTTAAAGGTGATACTCCGGAAGAA<br>AGATTCGCCTACTACTCGAAAACCTATTTAGGCAAACGTGAAGTAACTCACCGTCTGTATAGCGAATATCCGGTG<br>GTTCTGCGGTTGCTGTTCACCACCATTTCACACCACATTTCGTTCATTACGGAAATCCTTGAACGCGTTGCAAAT<br>GACCGTGAAGCCATTGAAACCGAATTTTCACCGTGTTCCCCGATTGGTACCCTCGCCTCTCTCCACTTAAACTCG<br>GGAGATGCTCACCATAAACAGCGTACTGTGACGATTTTGGAATTCTCCTCCTCGCTGAAACTTGTCTACAAACCT<br>CGCTCCCTCAAAGTTGATGGGGTGTTCAACGGTTTACTCGCTTTCCTGAACGATAGAACGGGGGAAGTCATTAAG<br>GACCAGTATTGCCCTAAGGTGTTACAGCGCGATGGCTACGGCTATGTGGAATTTGTCACTCACCAGTCTTGTCAA<br>TCCCTTGAGGAAGTGTCAGACTTCTACGAGAGACTCGGCTCTCTGATGAGTCTGTCCTACGTACTGAATAGTTCT<br>GACTTTCATTTCGAGAACATTATAGCTCATGGTCCCTATCCTGTCCTGATCGATCTTGAAACCATCATTCATAAT<br>ACAGCGGATAGCAGCGAGGAAACGTCTACCGCTATGGATCGCGCGTTCCGTATGTTGAACGATTCGGTGCTGTCC<br>ACTGGTATGCTTCCCTCCTCTATTTATTATCGCGATCAGCCGAATATGAAGGGTCTGAACGTCGGAGGTGTGAGC<br>AAATCAGAAGGTCAGAAAACACCGTTCAAAGTTAATCAAATCGCCAATCGCAACACCGATGAGATGCGTATCGAA<br>AAAGATCACGTTACCCTGAGCAGCCAGAAAAATCTGCCCATTTTTCAGTCTGCCGCAATGGAGAGCGTACATTTC<br>TTAGATCAGATCCAGAAAGGCTTTACCTCCATGTATCAGTGGATCGAGAAGAACAAACAAGAATTTAAAGAACAG<br>GTGCGTAAGTTTGAAGGTGTGCCGGTTCGTGCTGTTCTTCGGAGCACGACTCGCTATACCGAACTGCTGAAATCT<br>TCCTACCACCCTGACCTGCTCCGCAGCGCGTTGGACCGTGAAGTACTGCTGAACCGTTTGACTGTTGACTCGGTA<br>ATGACCCCGTATCTCAAAGAGATTATTCCACTCGAGGTGGAAGATCTGCTGAACGGTGACGTGCCATACTTCTAC<br>ACCCTGCCGGAAGAACGCGCCCTGTATCAGGAAGCGTCTGCGATCAATAGTACGTTCTTTACCACTTCGATTTTC<br>CATAAGATTGACCAGAAAATCGATAAGCTGGGTATCGAGGACCATACCCAGCAAATGAAGATCTTACACATGAGT<br>ATGCTTGCCTCTAACGCTAACCATTACGCCGATGTTGCCGACTTGGATATTCAGAAAGGACACACCATTAAAAAC<br>GAACAGTACGTTGAGATGGCCAAAGACATCGGTGATTACCTGATGGAGTTATCGGTCGAGGGTGAAAATCAAGGG<br>GAACCAGATCTGTGTTGGATTTCGACCGTCCTGGAAGGGAGCTCTGAAATCATTTGGGACATCAGCCCAGTGGGC<br>GAAGATTTATACAACGGCAGCGCTGGCGTCGCTCTCTTTTATGCGTACCTGTTCAAAATTACAGGTGAAAAGCGT<br>TACCAAGAGATCGCATACAAAGCCCTGGTTCCGGTTCGCCGCAGTGTGGCCCAATTCCAGCACCATCCGAATTGG<br>AGCATTGGTGCGTTTAACGGAGCGTCAGGCTATCTGTACGCGATGGGTACGATAGCGGCCCTGTTTAATGATGAA<br>CGTTTGAAGCATGAAGTAACCCGCAGCATTCCGCACATTGAACCGATGATCCACGAGGATAAGATCTATGATTTC<br>ATTGGCGGTTCCGCAGGGGCGCTGAAGGTGTTCCTGAGCCTGTCGGGGCTGTTTGACGAGCCGAAGTTTTTGGAA<br>CTTGCCATTGCATGCAGCGAACATCTGATGAAAAACGCCATTAAAACGGATCAAGGTATCGGCTGGAAACCACCG<br>TGGGAGGTCACCCCACTGACCGGTTTCAGCCATGGGGTTAGCGGCGTCATGGCATCCTTCATCGAACTGTACCAG<br>CAAACCGGTGATGAGCGCTTGCTCAGTTACATTGATCAGAGTTTAGCCTATGAACGTTCCTTCTTCAGCGAACAA<br>GAGGAGAACTGGCTGACTCCGAACAAAGAAACACCCGTGGTAGCTTGGTGCCACGGCGCGCCGGGAATTTTGGTA<br>TCACGACTGCTTCTGAAGAAATGCGGCTATTTGGATGAAAAAGTCGAAAAAGAAATTGAGGTGGCATTATCCACA<br>ACTATCCGTAAAGGCCTTGGTAACAATCGCAGTCTTTGCCATGGTGATTTCGGCCAGCTGGAAATTCTTCGCTTT<br>GCGGCGGAAGTGTTAGGCGATAGCTATCTCCAGGAAGTTGTCAACAATCTGTCCGGCGAGTTGTATAATCTTTTC<br>AAAACGGAGGGATATCAGAGCGGAACCAGCCGCGGTACTGAATCCGTGGGCCTGATGGTAGGTCTGTCCGGGTTT<br>GGGTATGGTTTACTTTCAGCGGCATATCCATCTGCTGTCCCCTCAATCTTAACATTGGATGGTGAGATCCAGAAG<br>TACCGGGAGCCTCATGAAGCC	[28, 29]
halM2	Codon optimized	ATGAAAACGCCGCTGACCTCGGAACATCCTTCAGTGCCGACGACGCTGCCGCATACTAACGACACCGATTGGCTC<br>GAGCAATTACATGACATTTTGTCCATTCCTGTTACGGAAGAAATCCAGAAATATTTCCACGCCGAAAATGATCTG<br>TTCTCGTTTTTCTATACACCGTTCCTGCAGTTTACGTACCAGAGCATGTCGGACTACTTTATGACCTTCAAGACC<br>GATATGGCCCTGATCGAAAGACAGAGCCTCCTGCAAAGCACGCTGACCGCGGTACATCACCGACTCTTCCACTTA<br>ACGCATCGCACCCTTATTAGTGAAATGCATATTGATAAACTTACCGTTGGCCTGAATGGCTCTACGCCGCACGAG<br>CGCTACATGGATTTCAACCACAAATTCAACAAAACCTCGAAGTCGAAGAACCTGTTTAACATCTACCCAATTTTG<br>GGAAAATTGGTCGTTAACGAAACTCTGCGCACTATTAACTTCGTCAAGAAAATCATTCAGCACTACATGAAGGAC<br>TACCTGCTCCTGTCGGACTTCTTCAAAGAGAAGGACTTGCGTCTTACCAACCTGCAATTAGGCGTGGGGGATACA<br>CACGTTAATGGGCAATGCGTCACCATTCTGACGTTTGCATCAGGCCAAAAAGTGGTATACAAACCTAGATCATTG<br>TCGATAGATAAACAGTTCGGAGAATTCATCGAGTGGGTAAACTCGAAAGGTTTTCAGCCTTCCTTGCGTATCCCT<br>ATTGCGATTGATCGTCAAACCTATGGTTGGTATGAATTCATCCCTCATCAAGAGGCCACCAGCGAAGATGAAATA<br>GAACGCTACTATTCTCGCATCGGTGGTTATCTGGCGATCGCCTACTTGTTCGGGGCAACCGACCTGCACCTGGAT<br>AACCTGATCGCCTGCGGCGAACATCCGATGCTTATTGATTTGGAAACACTCTTTACCAACGATCTCGACTGCTAT<br>GACAGTGCGTTTCCGTTCCCGGCGCTGGCCCGCGAATTAACCCAATCCGTTTTTGGCACCCTTATGCTTCCCATC<br>ACCATCGCGTCGGGGAAACTGCTGGATATAGACCTGTCAGCAGTAGGAGGCGGTAAAGGTGTGCAGTCCGAAAAG<br>ATCAAAACCTGGGTCATCGTGAATCAGAAAACTGATGAGATGAAGCTGGTCGAGCAGCCGTATGTTACCGAGAGT<br>TCCCAGAATAAACCAACAGTTAATGGGAAAGAGGCGAACATTGGCAATTATATTCCTCATGTCACAGATGGCTTT<br>CGTAAAATGTACCGCCTGTTTCTGAATGAAATTGATGAGTTAATGGATCATAACGGGCCAATCTTTGCGTTTGAG<br>AGTTGTCAGATTCGTCATGTTTTTCGAGCTACCCACGTGTATGCGAAATTTTTGGAGGCAAGTACCCACCCAGAT<br>TACTTGCAAGAACCTACCAGACGTAATAAACTGTTCGAGTCCTTTTGGAACATCACGTCGCTGATGGCACCGTTC<br>AAGAAAATTGTACCGCACGAAATCGCGGAGTTGGAGAACCATGATATTCCGTACTTCGTCCTGACTTGTGGCGGC<br>ACCATTGTTAAAGATGGATACGGCCGGGATATCGCAGACCTGTTTCAAAGTAGCTGCATCGAACGTGTAACTCAT<br>CGTCTGCAGCAGCTGGGAAGCGAGGATGAGGCGCGTCAAATTCGCTACATTAAAAGCAGCCTGGCGACGTTGACC<br>AACGGTGATTGGACCCCATCCCATGAGAAAACCCCGATGTCTCCGGCCTCGGCCGACCGTGAAGATGGTTACTTC<br>CTGCGCGAGGCTCAGGCCATCGGCGACGACATTTTGGCGCAGCTGATTTGGGAGGATGACCGTCACGCCGCTTAC<br>CTTATTGGCGTAAGCGTGGGCATGAACGAAGCCGTCACTGTGTCACCCCTGACGCCTGGCATCTACGACGGCACA<br>CTTGGCATAGTGCTGTTCTTCGATCAGCTGGCCCAGCAGACCGGCGAAACCCATTATCGCCACGCCGCCGACGCT<br>TTACTGGAAGGAATGTTCAAACAGCTGAAACCTGAACTGATGCCGTCTAGCGCTTACTTCGGACTGGGTAGCCTG<br>TTCTATGGCCTGATGGTGTTGGGCCTCCAGCGTTCCGACTCGCATATCATTCAGAAAGCGTATGAGTATCTGAAA<br>CATTTGGAAGAGTGTGTGCAGCATGAGGAAACGCCAGATTTTGTCTCGGGTTTGTCTGGTGTACTGTATATGCTC<br>ACGAAAATTTATCAGCTCACGAATGAACCGAGAGTTTTCGAAGTGGCCAAAACCACAGCTTCGCGTCTGTCTGTG<br>CTGCTTGACAGCAAGCAGCCCGACACTGTGCTCACCGGGTTATCCCATGGCGCCGCAGGATTCGCCCTTGCATTA<br>CTGACCTACGGAACCGCTGCAAATGATGAACAGTTGCTGAAACAGGGCCACTCCTATCTGGTGTACGAACGTAAT<br>CGGTTTAACAAACAGGAAAACAACTGGGTTGATTTACGTAAAGGCAACGCGTATCAAACATTTTGGTGCCATGGC<br>GCCCCGGGTATTGGCATCTCACGCCTCCTGTTAGCGCAATTTTACGATGACGAACTGCTGCATGAAGAGTTAAAC<br>GCAGCACTGAACAAGACTATTTCGGACGGCTTCGGCCACAATCACTCACTGTGTCATGGCGATTTCGGCAACCTC<br>GATCTGTTATTGCTTTATGCCCAATATACGAATAACCCAGAACCAAAGGAACTCGCTCGCAAACTGGCCATAAGC<br>AGTATCGATCAAGCGCACACGTATGGCTGGAAACTCGGGCTCAATCATAGCGATCAACTGCAGGGTATGATGTTA<br>GGGGTGACTGGTATCGGCTATCAGCTCCTTCGTCATATAAATCCGACAGTCCCCAGCATTTTGGCACTGGAACTG<br>CCCAGCTCCACGTTAACTGAAAAAGAGCTGAGAATCCATGATCGT	[28, 29]
kgpF	Codon optimized	ATGATCAATTATGCTAATGCGCAGCTCCATAAGAGTAAAAACTTGATGTATATGAAAGCCCACGAAAACATCTTC<br>GAAATCGAGGCGCTGTACCCGCTGGAATTGTTCGAGCGTTTTATGCAGTCCCAAACCGATTGCTCCATCGATTGT<br>GCCTGTAAAATTGATGGTGACGAATTGTATCCCGCCCGTTTTAGTCTGGCCCTGTATAACAACCAGTATGCCGAA	[37]

		AAGCAAATTCGCGAAACCATCGACTTCTTCCATCAGGTAGAGGGTCGGACCGAGGTGAAACTGAACTATCAGCAA<br>CTGCAGCACTTCCTGGGTGCTGACTTCGATTTTAGCAAAGTGATTCGAAACCTGGTGGGTGTGGATGCACGCCGC<br>GAACTGGCTGATTCCCGGGTTAAACTGTATATTTGGATGAACGATTACCCAGAGAAAATGGCGACCGCCATGGCA<br>TGGTGCGATGATAAGAAGGAATTGTCGACGTTGATAGTAAATCAGGAGTTTCTGGTCGGGTTCGATTTTTATTTC<br>GATGGTCGCACGGCAATAGAATTATACATTAGTCTGTCATCCGAAGAATTTCAGCAGACACAAGTTTGGGAACGC<br>CTCGCAAAGGTAGTGTGCGCCCCAGCGCTGCGCCTTGTTAATGATTGCCAGGCGATCCAGATTGGCGTGAGCCGT<br>GCCAATGATAGTAAGATCATGTATTACCATACCCTTAATCCGAACTCGTTTATCGACAATCTGGGCAATGAAATG<br>GCAAGCAGAGTTCACGCGTATTACCGACATCAACCGGTTCGCTCTCTGGTAGTATGCATACCAGAACAGGAGTTG<br>ACCGCCCGGTCCATACAGCGCTTAAACATGTATTACTGTATGAAC	
lasB	Codon optimized	ATGAAAGGCGAGGAAATGTTGGGACATCCACAGACCGGTTTTGTTGTACTGCCAGACAACGATGCCACCGGCGAC<br>GTGACGGGCCGCCTGTTACCTTGGGGTGATGTAGTTACAGTGTATCCGTCTGGCCGTCCATGGATCATCGGCAAC<br>TGCTGGGATCGCCCAGTCCTCGTCCATGATGGCGTGATCGTCTTGGGTCATACCAGCGTCACGCGTGATCAAATT<br>GCCCGTCATGGGAACGATCCGCATCGCTTACTGGACGAGGCCGACGGCGCATTTCATGCGGCGGTCCTGATCGGA<br>CACGAAGTTCATGTTCGCGGCTCCGCCTACGGTGTCTGTCGTCTGTATACATGCGTTGTTGACGGTGTGACCTTA<br>GTGAGTGATCGTACAGACGTCCTGCAGCGTCTGGCAGGTACTGATGTGGACGTCGACGTGCTGGCTGGCCACTTG<br>TTAGAGCCGATCCCGCACTGGTTAGGCGAACAACCGTTATTGACGTCCGTGGAGCCCGTGCCACCGACACATCAC<br>GTTATTTTAACTCCGGACGCACGTAGTCGTTTACGGCCATCACGTCGTCGTCGGCCTGAACCGTCGCTGGGTTTG<br>CGGGACGGTGCGGAACTTGTCCGGGAGCGTCTGGCCGCAGCTGTGGCTACCCGTGTGGACAGTCCAGCGTTAATT<br>ACCAGTGAACTGAGTGGCGGCTATGATTCCACTAGTGTGTCATACTTGGCAGCGCGCGGTAAAGCCGAGGTGGTG<br>CTGGTCACGGCCGCGGGACGTGACAGCACAAGCGAGGATCTGTGGTGGGCTGAACGCGCAGCCGCAGGGCTCCCG<br>GAACTCGATCACGTAGTGTTACCTGCGGATGAATTACCGTTTACGTACGCCGGCCTGACGGAGCCTGGTGCACTT<br>TTGGATGAACCGTGTACGGCTGTTGCCGGCCGTGAGCGTGTACTGGCGCTGGTACGTAAAGCCGCGGCCCGCGGC<br>TCTACACTTCATCTGACTGGCCATGGTGGCGATCACCTGTTTACTTCACTGCCGACACCGTTTCATGACCTGTTT<br>CGTACGCGTCCAGTCGCCGCGCTCCGCCAGTTGCGTGCATTTGGCGCGTTGGCTGCGTGGCCGACCCGTAAGCTG<br>ATGCGCGAACTCGCGGACCGCCGCGATCATAGCACCTGGTGGCGCGCGCACGCACGTCCTCAGAATGGCCAGCCG<br>GATCCGCACAGCCCCATGTTAGGCTGGGCAATTCCCCCGACTGTCCCGGCGTGGGTTACTGCTGACGGCGTGCGC<br>GCGATCGAACTTGGGATTTTAGAAATGGCAGAACGCGCGGAGCCCCTTGGTCATGCGCGCGGAGAACACGCTGAG<br>CTGGATTCAATCTTTGAAGGGGCGCGTATGGCCCGTGGCCTCAATCGTATGGCTACGCATGCCGGAGTCCCGCTT<br>GCAGCCCCGTTCCATGACGATCGGGTCGTGGAAGCGTGTCTGTCGATCCGGCCGGAGGAACGCATTTCTGCATGG<br>CAGTACAAACCCTTACTGAACGCCGCAATGCAGGGTGTGGTGCCGAGCACCGTTCTTGATCGTAGCGCTAAAGAT<br>GACGGGAGTATTGATGTGGCCTATGGGCTGCAGGAACACCGTGATGAACTGGTAGCGCTGTGGGAATCATCACGT<br>CTGGCGGAAACCGGTCTGATTGATGCGGGTATGCTGCGGCGTTTATGCGCGCAGCCGTCCTCCCACGAGCTCGAG<br>CATGGATCCTTGTACGCTACTATCGCTTGTGAGTTGTGGCTGCGTGGTTTAGATCAGGATCGTACCCAACGCTAC	[2]
lasC	Codon optimized	ATGCCGGTGCAGCTGCGTCGGCATGTGTCTTTTACGGCTACGGAATACGGCGGCGTGCTGCTGGATGAAACCAAA<br>GGCGCATACTGGCGTCTGAACACCACAGGCGCCGAAGTTGTTCGCGCCATGGGGGAAGCCGAGCGGGATGAGATT<br>GTACGGCATGTGGTGGCGACCTTCGATGTTGATGCGCAAACCGCAGCCCAGGATGTCGATGTCCTGCTGGCAGAA<br>CTTCGTGATGCCGGCCTTGTGGCCTCG	[2]
lasD	Codon optimized	ATGTCTGTGAATATGGCTCTCCGTGGCCATGGTATGTCCGGTCGCCGTCGTCGCTTAGATGCCACGCGTGCTCGC<br>CTGGCCGTTGTGGTTGCCCGTGTCCTGAATCTCTTACCGCCGCGCTTAATCCGTCGTTGTTTGCGTGTACTGAGT<br>CGCGGAGCCCGCCCTGCCTCGATTGAGGCAGCAGAAGCTGCTCGTCGTACTGTGGTTGCGGTGAGTCCAGCTGCC<br>GCCGGTGCGTACGGCTGTTTAATCCGCAGCATTGCCACCACCCTGGTTCTTCGTTCACGCGGGCAATGGCCAACC<br>TGGTGTGTTGGTGTACGTGCGGAGCCTCCTTTTGGTGCCCATGCCTGGATTGAAGCAGAGGAGCGGCTGGTGGAT<br>GAACCTGGTACTATGCATACTTACCGTCGTCTTATCACCGTTGGTCCACTGTCTCGCAAAGTTCGT	[2]
lasF	Codon optimized	ATGTCTATCGAACTGACGCCTAGTTTGGCCGATCTGGTCGATCCACTTCCAGGTCACGCACTGCGCGCTGCGGCG<br>ACATTACGTCTGGCAGATCTGATTGCGGCTGGTGCAGATACTGCACCGGCATTAGCAGCGGCGGCACGCATTGAT<br>GCTGACGCGATCGCGCGTCTTATGCGGTATCTGTGCAGTCGCGGATTTTTCAAGCACATGAAGGCCGGTACGCG<br>TTGACTGAATTTAGCGAATTGCTGCTGGATGAAGATCCATCTGGCCTGCGTAAAACCTTAGATCAGGATAGCTAT<br>GGGGATCGTTTCGACCGCGCGGTTGCGGAACTGGTGGACGTTGTACGGTCCGGTGAACCTTCTTATCCTCGCCTT<br>TACGGCTCGACGGTTTATGATGACCTGGCAGCCGATCCTGCCCTCGGCGAGGTGTTCGCGGATGTTCGTGGCTTG<br>CACTCCGCAGGGTATGGGGAAGATGTCGCGGCAGTGGCGGGTTGGTCCTCATGCCTGCGCGTTGTCGATCTGGGT<br>GGAGGGACTGGCTCCGTCCTGCTTGCTGTGTTAGAGCGTCACCCGTCCCTGTCAGGCGCAGTACTGGATCTGCCA<br>TACGTCGCCCCGCAGGCAAAGAAAGCTCTGCAGGCCTCAGCGTTTGCCCAACGTTGTGAATTTATCAAAGGGAGC<br>TTCTTCGATCCGTTACCTCCGGCAGACCGTTACCTGTTGTGTAACGTGCTGTTCAACTGGGATGACGCGCAAGCA<br>GGCGCTATTTTGGCACGCTGTGCGCAGGCGGGCCCTGTGGCCGGAGTAGTGGTAGCCGAACGTTTGATCGATCCG<br>GATGCGGAAGTGGAACTCGTAGCAGCTCAAGATCTGCGTCTGTTGGCTGTTTGCGGCGGTCGGCAGCGTGGCACC<br>GCTGAATTCGAAGCGCTTGGGGCAGCCCATGGCCTGGCGTTAACCAGCGTTACCCTCACGGCATCTGGTATGAGC<br>CTGCTCCGTTTCGATGTGTGTCGTGCCGGGAGTGCTGGCGGGGAAGTTGTGGAAAAATCT	[2]
lcnG	Codon optimized	ATGGACGGAACCAACAAGCGCCTGGAGGACAAGTGGTTTGATATTAACTTCCTGGAAATGTATACACGCAGCTGC<br>CTGAAAACTTTTGGCTACTTCGACGAAATTCTGATCGTGAAGAAACGCATCGAGGTCCTGAAGAACGTGCTTGAA<br>AAACAGTACTTGTCTACCAATGATTATGCTGAGGAGTTTTTCGAGCTGAATACCACCTTGGAGAGCATAAAAGAA<br>TACATCAAACTGAATCTGGTCATCGAGAAAGAACCGATCTCAATTTGCATTATGGTCAAAAACGAAGAACGTTGC<br>ATCAAGCGCTGCATTGATAGCGTTGAAATCCTCGCCGAGGAGATAATCATTATCGATACCGGCTCTACGGATAAT<br>ACCATTAACATTATTGAGGAATGCGCAAACGACAAAATTAAAGTGTTCTCAAAAGAATGGCGTAACGATTTTTCC<br>GAAATTCGGAACTATGCCATCGAGAAAGCGAGTAGCGAATGGCTGGTGTTTATAGATGCCGATGAATATCTGGAC<br>GAAGCCTCGGTGCTCAACCTGCTCAGTACGCTCAACATCTTTAACAATCATAAGCTCAAAGACTCTATTGTCCTG<br>TGCCCCATGATCAACGAAGCCAATAACACCATCCATTTCCGTACCGGGAAATTTTTCAGAAAAGACTCCGGGATT<br>AAATTCTTTGGTACCTGCCATGAGGAGCCCCGCATTAAAGGCATGCCGAATTCTACCCTGCTGATTCCGATCAAG<br>GTTGATTATCTGCATGACGGCTACCTGGCAAAAGTACAATCAAATAAAGACAAGAAAACCCGTAACATCGAACTG<br>TTAGAAGGTATGGTGGAACTGGAACCGGATAATCCTCGTTGGGCGTATATGTTTGTGCGCGACGGATTTGCAATC<br>CTCGATAACGAATACATTGAGAAAACTTGTTTGCGGTTTTTACTGCTGGACAAAAACGTACGCATCTGCGTCAAC<br>AACCTGCAAGACCATAAATTCACTTTGTCACTCCTGACGATCCTGGGCCGCCTCTATCTGCGCGAGTGCGAATTC<br>GAGAAAAGCAATCTGATAATTCGCATTCTTGACGAACTCATCCCTAATAGTCTGGATGGTAAATTTCTGGCATTC<br>ATGGAGCGATTCAGCAAACTGAAAATTGAGATTAATACGCTGTTAACGGAGGTCATCGAATATCGTCGTAACCAC<br>GAAGTAGATGAAACCAGTTTAATCAACACACAAGGCTACCATATCGACTATGTTCTGTCGATTTTGCTGTTCGAA<br>ACGGGTAATTACGCGCAAAGTAAGAAATACTTCGATTTCCTGCAGGAGAACCATTTTCTGGAAGAACTGTTTCAA<br>GACAGCTCTTATTCTATCATACTGAAAATGCTCGAGTCAGTAGAAGAT	[11]
ltnM1	Codon optimized	ATGAAGTTTAACAAGAACGTGTTCCCAGAGATCAATGAAACGGATTTCGATAACAATATCAAGCCCCTGCTGGAT<br>GAACTGGAATCTCGTATTACCATTCCGCAGGAGGAACTGAGCTTTTCAAGCATTAACGATGATTTATTTCGCGAG<br>TTAACCCGCAACGAGGAGTACCCTTACCAGAGCATTTGTACGATCGTTGCAAACATCGTGATGGATGACGGCAGT<br>GAGATTTGGCGCAAAGATATTTTTGTTGATTCCAATAGTGTGCGCGAAGCCGTATGCGACATTCTGAGCCAAACG<br>TTATTCCTCTATTTCATCCGCTGCTTCTCCGAACAAATTAAAGACATTCGCAAAACTGATGAGGATAAAGAGTCC<br>ACCTACAACCGCTACATTAACCTCCTGTTCAGCTCCAACTTCAAAATCTTCTCCGACGAATACCCTGTCCTGTGG<br>TATCGGACCATTCGCATCATCAAAAATCGCTGGTATTCTATCAAGAAATCGTTACTGCTGACTCAAAAACACCGT<br>GTGGAGATCGATAAGCAGTTGGACATCCCGCACAAGATGAAGATTAAAGGCCTGAAAATCGGGGGAGACACGCAT<br>AACGGCGGTGCCACAGTGACCACGATCTTCTTTGAGAAAGGGTATAAACTGATTTATAAGCCGCGGAGCACATCC<br>GGCGAATTCTCGTACAAGAAATTTATCGAAAAGATTAACCCGTACCTGAAGAAAGACATGGGAGCGATTAAAGCG<br>ATCGATTTCGGTGAATACGGCTTTTCTGAGTATATTGAGTGTAACACGGATGAAGAGGACATGAAACAGGTCGGT<br>CAGCTTGCATTTTTCATGTACCTGTTGAATGCATCAGATATGCATTATAGCAATGTCATTTGGACCAAACAGGGC<br>CCTGTGCCGATTGATTTAGAAACCTTGTTCCAGCCGGATCGTATTCGCAAAGGCCTGAAGCAGTCGGAAACTAAC<br>GCGTACCACAAAATGGAGAAAAGTGTATACGGAACGGGAATTATTCCAATTTCCCTGAGCGTTAAAGGCAAAAAG<br>GGTGAGGTCGACGTCGGCTTTAGTGGAATCCGTGATGAGCGCTCTAGTTCGCCGTTTCGCGTTCTGGAAATTTTG<br>GATGGGTTTTCGAGCGACATCAAAATCGTGTGGAAAAAGCAGCAGAAGTCTAGCTCCAGCAAAAACAATCTGATT	[23]

		GTCGATCACAAAAAGGAGCGCGAAATCCTTCAGCGTGCCCAGTCCGTCGTAGAAGGTTTCCAGGAAACCTCTAAA<br>ATCTTCATGAAACATCGTGAGGAATTCATCTCCATTATCTTAGACTCATTCGAGAACATCAAAATTCGCTACATC<br>CATAACATGACGTTTCGCTACGAACAGTTGCTGCGCACTCTGACGGATGCCGAGCCGGCCCAGAAGATTGAGTTA<br>GACCGTCTGCTGCTGAGTCGTACCGGAATTCTGTCCATCTCGTCTAGTCCCTACATCTCGCTCTCCGAATGTCAA<br>CAGATGTGGCAGGGTGACGTGCCGTACTTCTACTCGAAGTTTTCGAGCAAAAGTATCTTTGATACCAATGGCTTC<br>GTTGATGAAATCGAGCTGACGCCCCGCCAGGCATTTATCATCAAAGCCGAAAGTATCACCAACGATGAAGTCGAT<br>TTTCAGTCCAAGATCATTAAACTGGCGTTCATGGCACGCTTAAGTGACCCGCACACAACCAACGACAACAAACTG<br>AATAAAAAGGTGATTATCGAAAGCAACCAGCAGAGCAACAGCAGTGAATCAGGTAACAAAGCCATTTTGTTCCTG<br>AGCGATCTGCTGAAAAATAACGTACTGGAAGATCGTTATAGTCATCTGCCGAAAACTTGGATTGGCCCTGTAGCA<br>CGTGATGGCGGTTTGGGTTGGGCGCCGGGCGTGCTGGGATACGATCTGTACTCGGGCCGTACAGGACCTGCGTTA<br>GCATTGGCTGCGGCCGGGCGCGTTTTGAAAGATAAAGACAGTATCGAACTTAGCGCCGACATTTTTAATAAATCG<br>TCCCAGATTCTGCAGGAAAAGACTTACGACTTTCGTAACCTGTTCGCATCAGGTATCGGCGGTTTTAGCGGGATT<br>ACCGGTCTGTTTTGGGCGCTGAACGCGGCAGGGAATATTCTGAACAATGATGACTGGATTAAAACCTCGAATCAG<br>AGTATGCTGCTGCTGAATGAGAACATGCTGAAAGTGGACAAAAATTTCTTTGACCTGATTAGCGGCAACTCGGGA<br>GCGATCGGTATGATGTACCTGACCAATCCAAATTTCTATTTGTCTCGCTCGAAAATTAACGACATTCTGCTGACC<br>ACGGACTGCTTGATTACTGAAATGGAAAAAGACGAAACGAGCGGACTGGCCCATGGCGTGTCTCAGATCCTGTGG<br>TTCCTTAGCATTATGATGCAACGTCAGCCCTCAAGTGAAATCAAAATCCGCGCGACGATTGTCGACAACATCATC<br>AAGAAGAAGTATACGAATTCCTATGGCGAAATCGAATGCTACTATCCGACTGATGGGCACTCCAAATCCACCTCG<br>TGGTGCAACGGGACAAGTGGGATTCTGGTCGCCTATATTGAGGGGTATAAAGCTAATATCGTGGACAAATCCTCG<br>GTGTATCATATTATTAATCAGATCAACGTCGAACAACTTCAGCATGATAACATTCCGATCATGTGCCATGGTAGC<br>CTTGGTGTGTATGAATCGCTTAAATATGCGTCAAAGTACTTTGAAATCGAAACCAAGTACCTTCTGGATGTGATG<br>CGCAATGGCGGCTGCTCCTCCCAAGAAGTATTAAAGTACTATGGCAAGGGTAACGGCCGTTACCCGCTGTCACCA<br>GGTTTAATGGCGGGTCAGTCGGGCGCGTTGCTGCACTGTTGCAAACTGGAGGATAACGATATCAGCGTGAGCCCC<br>ATTTCACTGATGACG	
ltnM2	Codon optimized	ATGGATCCGAGTATCAAAAAGCTCGTGGATTCTATCATCGAATTCTACAAAAAGGACATCTACCTGGCATACAAA<br>GAGCTGGAACGCGAAATCAAAAACATCGATAAGACCATCTACAACACTTCAAATGACGAGATCTTGCGGATTTTT<br>AAAGAGAGCCTGATCAGCATCATCACCGATGATATTTACCGCCTCTCGATTAAAACCTTCATCTATGAGTTTCAC<br>AAGTTTCGTATCGATAACGGGTTTCCGGCTGTCAAAGATAGCGAAAGCGCCTTCAATTATTACATCAGTACCTTT<br>GACGTGAAAACGATCGCTCGCTGGTTTGAGAAATTCCCAATGCTGGAATCCATCATCTCCAGTAGCATCAAAAAC<br>GATTGCACATTTATGGTGGATGTATGTGTCAATTTCATCTTAGACCTGTCGGAATGCGAGAAGATTAATCTGATC<br>TCAGAGGATAGCCGGCTCATCACGATCTCATCCAGCAACTCTGACCCGCACAACGGTGGCACGCGTGTCTTGTTC<br>TTTCGTTTCCACAACGGTGATACCATTCTTTACAAACCCCGCAGCCTGACCGTGGACAAGCTGATCTCTAATATT<br>TTCGAAGAGGTATTCGAATTCGATGCGACGAACTCGAAAAATCCTATTCCCAAGGTGCTGGATCGGGGTACCTAT<br>GGCTGGCAGGAATTCATTGAGAAGAAATCGATCTCTTCCTCAGAGATTAAGCAGGCCTACTATAACCTGGGTATC<br>TTTAGCAGTATCTTTACAGTGTTAGGGTCTACTGATATCCACGATGAAAACTTGATTTTTAAAGGTACGACCCCG<br>TATTTCATCGATCTGGAAACAGCCCTCTCTCCGCGTATCCGGTATGAAGGTAATGAGGAAAACCTGTTCTATCGG<br>ATGAGCTCATCGTTGTTCACTTCTATCGTGGGGACGACTATTATTCCTGCAAAACTTGCTGTCCATTCCCAGGAA<br>ATTATGATCGGCGCAATTAACACCCCTGCGAAACAGAAAACCAAGAAGGATGGCTTTAACATCATCAACTTCGGC<br>ACGGATGCCGTCGATATCGCAAAACAGAATATTGAGGTGGAGCGTATTGCTAACCCTATGCGCATTAAAAATAAC<br>ATCGTGAACGATCCGCTGCCGTACCAGAACATCTTTACGCGCGGCTTCAAAGAGGGGATCAAATCCATCATCCTG<br>AAGAAAGGCTCGATCATTTCCATTCTGAACAACTTCAACAGCCCGATTCGTTACATCATGCGGCCGACGGCAAAA<br>TATTATTTGATTCTGGATGCCGCGGTATTTCCCGAAAACCTGTATTCGGAACAGACACTGAACAAAACCCTGAAT<br>TACTTAAAGCCGCCAAAAATCGTGGAAAATTCCCTGATTTCTAAACAGCTCTTTCTTGCCGAAAAACGCATTCTG<br>TCCGAAGGCGATATTCCGAGCTTCTATGTGCTGGGCAAAGAGAAAAATATCCGTGCGCAGAACTTCATTAGCGAA<br>CAGATCTTCGAGGAAACCGCGGTCGATAACGCGATTCAAATTCTGGAATCCATTTCGCAAGACTGGGTGAATTTT<br>AATGAGCGCCTGATTGCGGAGGGCTTCTCCTATATTCGTGAACAGAGTCGTGGCTATCTGTCCAGTGATTTTGAG<br>AACTCTGATATTTTCAAAAGCTCACTGACCGAAACAAAGAAGTCCGGTTATACCGCAATGCTGAAAACAATTATC<br>TCCATGTCGGTCAAGACCTCGGAAAACAAAAAGATCGGTTGGCTGCCAGGCATTTATGATGATTATCCGATCAGC<br>TATATGAGTGCCGCGTTTTGTTCGTTCCATGATTCCGGCGGTATCATCACTTTGCTTGAACACCACTTTGGGCAC<br>TGCTCCCCCGAATATAACGAGATGAAGCGCGGGCTGCTGGAACTGGGCAAAATGTTGAAAATTAACAATAGTAAC<br>CTGAGCATCATCTCCGGCTCAGAGTCTCTGGAATTTCTGTATACGCACCGCGAAGTCGAATGCCTGGAACTGGAA<br>TACATTTTAAACAATTCAGCGGAAATCATGGGCGACGTGTTCCTGGGGAAATTAGGCCTTTATCTTATCCTGGCG<br>AGCTACCTGAAAACAGACCTGAAAATTTTCCAAGATTTCAGTATCATCTGCCAGAAAAACCTCGAGTTTAAAAAG<br>TTCGGGATCGCGCACGGTGAATTAGGGTATCTGTGGACCATCTTCCGTATTCAAAACAAACTGAAGAACAAAAAT<br>GCGTGTCTGAGCATCTATCATGAAGTGTTGAACATTTATAAAGGTAAGCGCATTGAATCCGTGGGATGGTGCAAC<br>GGTTTATCGGGTATTCTGATGATTTTGTCAGAAATGAGCACCGTATTAGAGAAAAATCAAGACTATCTGTTCAAG<br>CTGGCAAATCTGAGCACTAAACTGAATGAGGAATCCGTTGACCTGAGTGTGTGCCACGGCGCCAGCGGGGTGCTT<br>CAAACACTGCTTTTCGTCTATAGCAACACGAACGATAAACGTTATCTCAGCCTGGCCAATAAGTATTGGAAGAAA<br>GTGCTGGATAACAGCATTAAGTACGGTTTCTACAATGGAGAACGCGATAAGGATTATCTGTTGGGATATTTCCAG<br>GGTTGGTCAGGCTTCACGGACAGCGCACTCCTGCTGGATAAATACAATAACAATGAGCAAGTGTGGATTCCGATC<br>AACCTGAGCTCCGATATCTATCAGCATAATCTGAACAACTGCAAAGAGAAGAATTATGAGGGCGATGGCTGCCAT<br>AAATCT	[23]
lynD	Codon optimized	ATGCAATCTACACCATTACTGCAAATACAACCACATTTCCATGTAGAGGTCATTGAACCAAAGCAAGTCTACTTG<br>TTGGGTGAACAAGCTAATCATGCATTGACAGGCCAATTATACTGCCAAATTTTGCCATTGTTAAACGGACAATAC<br>ACATTGGAACAAATCGTTGAAAAACTAGACGGAGAAGTACCACCTGAATACATTGATTATGTGCTGGAGAGACTA<br>GCTGAGAAGGGCTATCTGACTGAAGCAGCACCTGAATTATCTAGTGAAGTGGCCGCTTTCTGGTCTGAGCTGGGG<br>ATTGCACCTCCTGTCGCGGCCGAAGCATTACGTCAACCTGTGACTTTAACACCTGTTGGAAACATCAGCGAAGTA<br>ACAGTAGCAGCCTTAACCACAGCCCTACGTGATATCGGTATTTCCGTTCAAACACCTACAGAAGCTGGATCGCCA<br>ACTGCATTGAACGTTGTACTTACCGATGATTATCTCCAACCAGAACTCGCTAAGATCAATAAGCAAGCCTTAGAA<br>AGTCAACAAACTTGGCTACTTGTCAAACCAGTTGGCTCCGTGTTATGGTTGGGTCCGGTATTCGTGCCAGGAAAA<br>ACAGGTTGCTGGGATTGTTTGGCTCACAGATTAAGGGGGAATAGAGAGGTAGAGGCCTCTGTATTGAGACAAAAA<br>CAAGCTCAACAACAACGTAATGGACAAAGCGGGTCTGTAATAGGATGCCTTCCCACGGCTAGAGCGACACTGCCC<br>TCAACACTCCAAACTGGGCTGCAGTTCGCTGCTACCGAAATTGCTAAATGGATAGTTAAGTATCATGTTAATGCC<br>ACAGCGCCTGGCACCGTATTCTTCCCTACATTGGATGGTAAGATAATTACGCTAAATCACTCCATACTGGATTTG<br>AAGTCACATATTCTGATCAAGCGTTCTCAATGTCCCACCTGTGGTGACCCAAAAATCTTACAGCACCGTGGTTTC<br>GAACCTTTAAAACTTGAGTCAAGGCCTAAACAGTTCACCTCAGACGGCGGACATCGTGGTACTACCCCTGAACAA<br>ACTGTCCAGAAATATCAACATTTAATCTCGCCTGTTACCGGTGTAGTTACTGAATTGGTCAGGATAACTGATCCG<br>GCCAATCCACTAGTTCACACATATAGAGCTGGTCATAGCTTCGGGAGCGCTACATCGCTGAGAGGGCTGCGTAAT<br>ACCTTAAAGCATAAGAGTTCAGGTAAGGGTAAGACTGATTCTCAAAGTAAAGCCTCGGGCCTGTGTGAGGCGGTA<br>GAACGTTACTCAGGAATCTTTCAAGGTGACGAACCGAGAAAACGCGCCACATTGGCTGAATTGGGAGATTTGGCA<br>ATTCACCCTGAGCAATGCTTGTGTTTTTCCGACGGTCAGTACGCTAATAGAGAAACTTTAAACGAACAGGCAACG<br>GTGGCACATGATTGGATACCTCAACGTTTTGATGCATCACAAGCTATTGAATGGACTCCAGTCTGGTCCCTAACT<br>GAACAGACCCATAAATATTTGCCCACCGCATTGTGTTACTACCATTATCCTCTACCCCCAGAACACAGATTCGCA<br>CGTGGAGATTCGAATGGTAATGCTGCCGGAAATACGTTGGAAGAGGCTATACTCCAAGGCTTCATGGAATTAGTC<br>GAGAGAGATGGTGTGGCTTTATGGTGGTATAACAGGCTACGCAGACCCGCTGTAGACTTAGGCTCATTTAACGAG<br>CCATACTTCGTTCAGTTGCAACAATTCTACAGAGAAAACGATAGAGATTTGTGGGTTTTGGACTTGACAGCTGAT<br>TTAGGTATCCCGGCTTTCGCGGGCGTTTCTAATAGAAAAACTGGTAGTTCGGAGAGGTTGATATTAGGATTCGGT<br>GCACACCTCGATCCTACTATTGCAATTCTGAGAGCAGTTACAGAAGTTAACCAGATTGGCCTTGAATTAGATAAA<br>GTTCCAGACGAGAACCTTAAGAGCGACGCAACAGATTGGCTAATTACTGAAAAATTAGCTGACCACCCTTATTTG<br>TTACCAGATACAACTCAACCTCTAAAAACTGCTCAAGATTATCCTAAAAGGTGGTCTGACGATATATACACGGAC<br>GTAATGACTTGCGTTAATATTGCTCAACAAGCAGGACTTGAAACTCTAGTTATTGATCAAACACGTCCGGACATT<br>GGTTTGAATGTTGTTAAGGTGACAGTCCCGGGGATGAGGCACTTTTGGTCAAGATTTGGAGAGGGGAGGCTTTAT<br>GACGTGCCCGTCAAATTAGGTTGGCTTGACGAACCTTTGACCGAAGCGCAAATGAACCCCACGCCGATGCCTTTT	[36]

mcbCD	Synthesized without codon optimization as overlapping reading frames (same as native *E. coli* cluster)	ATGTCAAAACACGAACTCTCTTTAGTGGAAGTAACGCATTACACAGATCCTGAAGTTCTGGCCATTGTTAAAGAT<br>TTTCATGTCAGAGGTAACTTTGCTTCCCTCCCCGAATTTGCTGAACGAACTTTCGTGTCCGCGGTACCTCTTGCC<br>CATCTGGAGAAATTTGAAAATAAAGAAGTTCTCTTCAGGCCAGGTTTCAGCTCCGTAATAAACATATCCTCATCA<br>CATAATTTTAGTCGTGAAAGGCTCCCATCAGGAATAAACTTTTGCGACAAAAATAAACTTTCCATTCGTACTATT<br>GAAAAGTTATTAGTCAATGCATTCAGCTCACCTGATCCTGGCTCTGTAAGGCGGCCTTATCCTTCTGGGGGGCA<br>TTGTACCCGATTGAAGTTTTTTATGCAGATTATCTGAAAATACAGAAAACTGGCAGGCAGGAACTAATGTTTAT<br>CACTACCTGCCGCTAAGTCAGGCACTAGAACCTGTTGCTACATGTAATACTCAGTCACTCTACCGAAGCCTGTCC<br>GGTGGGGATTCGGAACGTCTTGGTAAACCCCATTTTGCTCTCGTCTATTGCATTATTTTTGAAAAAGCTTTGTTC<br>AAATATCGCTACAGAGGATACCGGATGGCCTTAATGGAAACAGGTTCGATGTATCAGAACGCAGTATTGGTTGCA<br>GATCAAATAGGACTGAAAAACCGGGTATGGGCGGATATACCGATTCATACGTAGCAAAAACAATGAATCTGGAT<br>CAGAGGACTGTAGCGCCACTGATCGTTCAGTTTTTTGGAGATGTAAACGATGATAAATGTCTACAGTAACCTTAT<br>GTCCGCATGGCCGGCCACAATGGCCATGAGTCCAAAACTGAACAGAAATATGCCAACGTTTTCTCAGATATGGGA<br>CTATGAGCGTATTACACCAGCCAGCGCGGCCGGTGAAACTCTGAAGTCAATTCAGGGGGCAATAGGTGAATATTT<br>TGAACGCCGTCATTTTTTTAATGAGATAGTCACCGGTGGTCAGAAAACATTATATGAGATGATGCCTCCATCTGC<br>TGCAAAGGCTTTTACCGAAGCATTTTTTCAGATCTCATCACTGACCCGCGATGAAATCATAACCCATAAATTTAA<br>AACGGTCAGAGCCTTTAATCTGTTTAGCCTTGAACAACAAGAAATACCTGCAGTCATAATTGCACTCGACAATAT<br>AACCGCTGCAGATGATCTGAAATTTTATCCTGACAGAGATACATGCGGATGTAGCTTTCATGGTAGTTTGAACGA<br>TGCCATAGAAGGTTCCTTGTGTGAATTTATGGAGAGACAGTCCCTCCTTCTTTACTGGTTACAGGGAAAAGCCAA<br>TACTGAAATATCCAGTGAAATAGTAACAGGCATAAATCATATAGATGAGATTTTACTGGCTCTCAGGTCAGAAGG<br>AGATATCAGGATTTTCGATATCACCCTGCCCGGAGCTCCTGGACACGCAGTACTAACCCTGTATGGCACAAAAAA<br>CAAAATCAGTCGAATAAAATACAGTACCGGATTATCCTATGCTAATAGTCTGAAAAAAGCACTTTGTAAATCCGT<br>AGTGGAATTGTGGCAATCGTATATATGCCTGCACAACTTTCTTATTGGCGGTTATACTGATGATGACATTATTGA<br>TAGTTACCAGCGTCACTTTATGTCATGCAACAAGTACGAGTCGTTTACGGATTTGTGTGAAAATACGGTACTACT<br>GTCTGATGATGTCAAGTTAACGTTTGAGGAAAATATTACGTCAGACACAAATTTATTAAACTATCTTCAACAAAT<br>TTCTGATAATATTTTTGTTTACTATGCCAGGGAAAGAGTAAGTAACAGCCTTGTCTGGTACACAAAAATAGTAAG<br>CCCTGATTTTTTCCTTCATATGAATAACTCAGGTGCAATAAACATTAATAATAAAATTTACCATACCGGGGACGG<br>TATTAAAGTCAGAGAATCAAAGATGGTACCATTCCCA	*E. coli*<br>[25]
mdnC	Amplified from pARW071	ATGACCGTTTTAATTGTTACTTTTAGCCACGATAATGAAAGTATTCCTCTGGTAATCAAAGCCATAGAAGCCATG<br>GGTAAAAAAGCCTTCCGTTTTGATACTGATCGCTTCCCTACAGAGGTGAAAGTTGATCTTTACTCAGGCGGTCAA<br>AAAGGCGGAATTATTACCGATGGAGAACAAAAATTAGAGCTAAAAGAAGTTTCTTCTGTCTGGTATCGACGCATG<br>AGATACGGACTAAAATTACCCGATGGGATGGATAGTCAATTTCGCGAAGCTTCTCTTAAGGAATGTCGGTTAAGT<br>ATTCGAGGAATGATTGCTAGTTTATCTGGCTTTCATCTTGATCCAATTGCTAAGGTAGATCATGCTAATCATAAA<br>CAATTGCAGTTACAAGTGGCGCAACAATTAGGTTTATTAATTCCGGGGACTTTAACTTCTAATAATCCTGAAGCT<br>GTCAAGCAATTTGCTCGGGAGTTTGAAGCGACGGGAATTGTGACTAAAATGCTTTCTCAATTTGCTATTTATGGA<br>GACAAGCAAGAGGAAATGGTTGTTTTTACCAGTCCTGTTACAAAGGAAGATCTAGATAATTTGGAAGGTTTGCAA<br>TTTTGTCCAATGACTTTTCAGGAAAACATTCCTAAAGCTTTGGAATTACGCATCACTATCGTCGGTGAACAAATA<br>TTTACGGCGGCGATTAATTCCCAACAATTAGACGGTGCTATCTACGATTGGCGAAAAGAGGGACGCGCGCTCCAT<br>CAACAATGGCAACCCTACGATTTACCGAAAACTATTGAAAAACAACTACTAGAATTAGTGAAATATTTCGGTCTT<br>AATTATGGTGCAATTGATATGATTGTCACACCAGATGAACGTTATATCTTTTTAGAAATTAATCCCGTTGGCGAG<br>TTTTTCTGGCTAGAACTTTATCCTCCTTATTTTCCTATCTCCCAGGCGATCGCTGAAATCCTAGTTAACTCA	[33]
mibD	Codon optimized	ATGACGGCACACAGCGACGCAGGAGGTGACCCACGCCCGCCTGAACGCTTACTGTTGGGGGTGTCAGGAAGTGTC<br>GCTGCACTGAACTTACCGGCGTACATTTATGCCTTTCGGGCAGCCGGTGTGGCACGTCTTGCGGTCGTGCTGACA<br>CCAGCGGCTGAAGGGTTCCTTCCAGCGGGTGCGTTACGCCCGATTGTGGATGCCGTTCATACGGAACATGACCAA<br>GGCAAAGGTCACGTAGCGCTGTCACGCTGGGCGCAACACTTACTCGTGCTGCCGGCAACAGCGAATTTGCTTGGC<br>TGTGCAGCGTCAGGACTTGCGCCGAACTTTTTAGCGACCGTTCTGCTCGCGGCAGATTGCCCAATCACATTCGTC<br>CCGGCGATGAATCCGGTCATGTGGCGTAAACCAGCCGTACGCCGGAACGTTGCAACCTTACGCGCAGATGGTCAT<br>CACGTGGTGGATCCTCTGCCGGGCGCTGTGTACGAAGCTGCCTCACGTTCTATCGTGGAAGGTCTTGCTATGCCG<br>CGCCCTGAAGCGTTAGTCCGTTTACTGGGTGGCGGTGATGACGGTTCTCCAGCAGGACCGGCAGGTCCGGTTGGA<br>CGCGCAGAGCATGTTGGGGCTGTTGAGGCTGTTGAAGCCGTGGAAGCAGTTGAGGCCGTTGAGGCTGCGGAAGCA<br>CTTGCG	[26]
mibH	Codon optimized	ATGGCACGTAGTGAGGAATCGAACACTCTGGCACGTCTGTTTGACGTGTTGGGTGACGATGCCGCTGCCGCACGT<br>GAATGGGTAACGGAACCCCATCGTCTGATCGCTAGCAATGAGCGCCTGGGCACAGCTCCGGAAGCCCCGGCGGAT<br>GACGATCCGGAGGCCATTCGGACGGTTGGAGTGATCGGAGGGGGCACAGCCGGGTATTTAACGGCGTTGGCTCTG<br>AAGGCTAAACGCCCTTGGTTGGATGTGGCGCTCGTCGAAAGTGCGGATATCCCGATCATTGGGGTAGGAGAGGCG<br>ACGGTGTCTTATATGGTGATGTTTCTGCACCATTATCTGGGCATTGATCCGGCGGAGTTTTACCAACATGTGCGC<br>CCTACTTGGAAACTGGGCATCCGTTTTGAATGGGGGTCACGTCCGGAGGGCTTTGTTGCGCCATTCGATTGGGGG<br>ACCGGATCTGTTGGCCTGGTTGGGAGCCTGCGTGAAACGGGCAATGTCAACGAAGCTACGTTACAGGCGATGCTC<br>ATGACGGAGGATCGCGTTCCGGTATATCGTGGCGAAGGTGGGCATGTTAGTCTGATGAAATATCTGCCATTCGCA<br>TATCATATGGATAACGCTCGCCTGGTTCGCTACCTGACGGAACTCGCCACTCGTCGTGGCGTGCATCATGTCGAT<br>GCGACTGTAGCTGAAGTTCGCCTGGATGGTCCTGACCACGTTGGGGACCTGATTACTACGGACGGTCGTCGCCTG<br>CACTATGACTTTTACGTCGATTGTACTGGATTTCGTTCCCTGCTGCTGGAAAAAGCCCTGGGTATCCCGTTCGAA<br>TCTTATGCGTCAAGCCTGTTTACCGACGCGGCAATTACCGGTACCCTTGCACATGGGGGTCATCTTAAACCTTAC<br>ACTACGGCAACTACCATGAATGCGGGCTGGTGTTGGACGATCCCTACTCCTGAGTCCGATCACCTGGGGTACGTT<br>TTCAGTAGTGCCGCGATCGATCCAGACGATGCAGCAGCAGAAATGGCCCGCCGTTTCCCGGGCGTTACCCGCGAA<br>GCATTAGTTCGCTTTCGCTCCGGCCGTCACCGTGAAGCTTGGCGCGGCAATGTCATCGCGGTAGGAAACAGCTAT<br>GCTTTCGTGGAACCTCTGGAGAGTTCGGGACTCCTGATGATTGCTACCGCAGTCCAGATCCTGGTGAGTTTGCTG<br>CCGAGTAGTCGTCGTGACCCGCTGCCTAGCAATGTGGCGAATCAGGCGTTAGCTCACCGGTGGGACGCGATTCGT<br>TGGTTTCTGAGTATTCATTACCGTTTCAACGGCCGCCTCGATACTCCGTTCTGGAAGGAAGCCCGTGCCGAAACA<br>GATATTAGCGGTATTGAACCGTTGCTTCGTCTGTTCAGTGCCGGTGCCCCTCTGACCGGTCGCGATAGCTTTGCG<br>CGCTATTTGGCCGACGGAGCAGCCCCGTTGTTCTATGGCCTGGAGGGTGTTGATACCTTACTGCTGGGACAGGAA<br>GTGCCTGCGCGTCTGTTACCACCGCGTGAATCTCCTGAGCAGTGGCGTGCCCGTGCTGCAGCAGCCCGCTCATTA<br>GCCTCGCGTGGCTTACGTCAGAGCGAAGCTCTGGATGCTTACGCTGCGGACCCCTGTCTCAATGCGGAACTGCTG<br>TCTGATAGCGACTCATGGGCGGGTGAACGCGTCGCGGTACGTGCAGGTCTGCGT	[26]
mibO	Codon optimized	ATGATTTTTGGCCCGGATTTTCATCGCGATCCGTATCCAGTGTATCGTCGTCTGCGTGATGAGGCTCCGTGCCAC<br>CATGAACCAGCGTTAGGTCTGTATGCGTTGAGCCGCTACGAGGACGTTCTGGCTGCCCTTCGTCAGCCCACCGTG<br>TTCAGCTCAGCAGCGCGTGCGGTAGCCTCCAGTGCAGCGGGAGCAGGTCCATACCGCGGTGCCGACACCGTTAGT<br>CCGGAGCGGGAAACTGCGGCTGAAGGGCCCGCCCGTAGCCTGTTGTTCCTGGATCCGCCAGAGCACCAGGTGCTG<br>CGTCAGGCGGTGTCCCGTGGCTTTACGCCGCAGGCAGTATTGCGCCTTGAGCCGGCCGTCCGCGACATTGCGGCG<br>GGTCTTGCTGATCGTATCCCCGATCGCGGTGGTGGCGAGTTCGTTACCGAATTTGCGGCTCCGCTGGCAATCGCA<br>GTGATTCTGCGGTTACTTGGTGTACCGGAAGCAGATCGTGCCCGCGTAAGCGAACTTTTATCGGCATCAGCCCTG<br>TCGGGGGCGGAAGCAGAACTGCGCTCCTATTGGCTGGGCCTTTCGGCACTCCTCCGCGATCGTGAAGATGCAGGC<br>GAAGGTGACGGAGAGGATCGTGGTGTGGTGGCGGCTCTGGTCCGTCCTGATGCTGGACTGCGCGACGCGGATGTT<br>GCCGCAGGACCTGCCGTGCGTGCACCGCTGACGGATGAGCAGGTTGCAGCATTCTGCGCCTTAGTGGGGCAAGCC<br>GGCACTGAAAGTGTGGCAATGGCGCTCTCCAACGCATTGGTCCTGTTCGGGCGTCACCATGACCAGTGGCGCACA<br>CTGTGTGCGCGTCCGGATGCGATTCCAGCAGCATTCGAAGAGGTCCTCCGCTATTGGGCACCTACGCAGCATCAA<br>GGTCGGACGTTAACCGCGGCGGTACGTTTACATGGCCGTCTGCTGCCGGCCGGTGCGCATGTACTGCTGCTGACC<br>GGTTCAGCCGGCCGGGATGAACGTGCGTACCCAGACCCCGATGTATTTGACATCGGTCGCTTCCACCCGGATCGT<br>CGTCCGTCGACCGCGCTGGGTTTTGGTCTGGGCGCACACTTTTGTTTAGGCGCTGCTCTCGCTCGTCTGCAGGCA<br>CGCGTAGCGCTGCGCGAACTGACACGCCGGTTCCCGCGTTATCGTACGGACGAGGAACGCACTGTGCGTTCGGAA<br>GTGATGAACGGGTTCGGCCACAGCCGTGTACCATTTTCCACG	[26]
mibS	Codon optimized	ATGACGACTGGCACCACGGTAGCGCATGCTGTAGAACCAGACGGTTTCCGCGCCGTGATGGCCACACTGCCGGCC<br>GCTGTGGCGATCGTTACGGCAGCTGCGGCAGATGGGCGCCCGTGGGGTATGACCTGCAGTTCGGTTTGCTCAGTG	[26]

		ACCTTGACCCCGCCGACCCTTCTGGTCTGCCTTCGGACGGCGTCCCCGACTCTGGCCGCAGTCGTGTCAGGTCGT<br>GCATTTAGCGTGAACCTTCTGTGTGCGCGGGCCTATCCTGTGGCGGAATTGTTTGCATCTGCGGCAGCAGACCGG<br>TTTGATCGCGTTCGTTGGCGTCGCCCGCCGGGTACAGGCGGTCCACATCTTGCCGATGATGCACGTGCAGTGTTA<br>GACTGTCGCCTGAGCGAAAGCGCAGAAGTAGGCGACCATGTGGTCGTATTTGGCCAAGTCCGGGCGATTCGTCGC<br>CTGAGTGATGAACCACCACTGATGTATGGTTATCGTCGTTACGCACCTTGGCCGGCAGATCGTGGTCCGGGTGCG<br>GCAGGCGGC	
paaA	Codon optimized	ATGAGCCTGACGAATGTCAAGCCGTTGATTAAAGAATCCCACCACATCATTTTAGCTGACGATGGTGACATTTGC<br>ATTGGGGAAATTCCGGGGGTGTCTCAGGTAATCAATGACCCGCCGTCGTGGGTTCGTCCTGCCCTGGCAAAGATG<br>GATGGCAAGCGTACTGTCCCCCGTATTTTCAAAGAACTGGTCAGTGAAGGCGTACAGATCGAATCCGAACATCTG<br>GAAGGCCTGGTAGCCGGGCTTGCCGAACGCAAACTTCTCCAGGATAACAGTTTCTTTTCCAAGGTGTTAAGCGGT<br>GAAGAAGTGGAGCGCTATAACCGCCAGATTCTGCAGTTCAGCCTTATCGATGCGGATAACCAGCACCCTTTCGTT<br>TACCAAGAGCGGCTGAAACAGTCTAAAGTCGCTATCTTCGGTATGGGTGGCTGGGGCACGTGGTGTGCATTGCAG<br>CTGGCCATGTCAGGCATTGGTACACTGCGGCTGATCGACGGCGATGATGTGGAACTGTCGAACATTAACCGCCAA<br>GTTCTGTATCGCACGGATGATGTAGGTAAAAACAAAGTTGATGCCGCCAAAGACACTATCCTGGCATACAACGAA<br>AACGTGCATGTTGAAACCTTCTTTGAATTCGCCAGCCCGGACCGTGCCCGGCTTGAAGAACTTGTGGGTGATTCT<br>ACCTTTATTATCCTGGCTTGGGCCGCGTTGGGTTACTACCGTAAAGATACGGCAGAGGAAATTATCCATTCGATT<br>GCGAAAGATAAAGCGATCCCTGTAATTGAACTCGGCGGTGATCCTTTGGAAATCTCTGTCGGTCCTATTTACCTG<br>AATGATGGCGTACACAGCGGCTTCGACGAGGTGAAAAATTCCGTTAAAGATAAATACTACGACAGCAACAGCGAT<br>ATCCGCAAATTTCAAGAGGCGCGGTTGAAACACAGCTTCATCGATGGCGATCGTAAAGTGAACGCGTGGCAATCA<br>GCGCCCAGCCTGAGTATTATGGCTGGTATCGTAACGGATCAGGTTGTGAAAACCATTACCGGGTACGACAAGCCA<br>CATCTCGTTGGCAAGAAATTTATCTTGAGTCTGCAAGATTTCCGCAGCCGCGAGGAGGAGATCTTTAAA	[15, 16]
padeK	Codon optimized	ATGACCGAACGTGCCGCAGTGCGTACCGACCATTATAAAGCCTTTGGGTTTAGAATTGAAAGCGATTTCGTGCTC<br>CCGGAACTTCCGCCCGCAGGCGAACGCGAACCGCTCGATAATATTACGGTTCGTCGTACCGACCTGCAGCCGCTC<br>TGGAATTCTAGTATCCATTTTTACGGAAACTTTGCCATTCTGGATCACGGACGCACGGTTATGTTTCGAGTTCCG<br>GGTGCTGCTATCTATGCGGTACAGGATGCTAGCAGCATATTAGTGTCCCCATTCGATCAGGCAGAAGAAAACTGG<br>GTACGTCTTTTTATTCTGGGTACCTGTATTGGGATCATCCTGCTGCAGCGTAAGATTATGCCGCTGCACGGTAGC<br>GCCGTTGCCATTGATGGCAAAGCCTACGCGATTATCGGCGAATCTGGTGCCGGCAAAAGCACTCTTGCACTGCAT<br>CTTGTCAGTAAGGGTTATCCATTGCTTTCGGATGATGTGATTCCGGTCGTTATGACCCAGGGCTCCCCCTGGGTG<br>GTGCCGTCGTACCCGCAACAAAAACTTTGGGTGGACACTCTGAAGCACATGGGAATGGATAATGCAAACTATACG<br>CCGCTGTACGAACGTAAAACGAAGTTCGCGGTGCCCGTGGGCAGTAATTTCCACGAAGAACCGCTGCCGTTAGCT<br>AGCATTTTCGAGCTTGTCCCGTGGGATGCGGCAACGCACATTGCCCCGATCCAAGGGATGGAACGCTTTCGTGTC<br>CTGTTCCACCACACTTATCGGAACTTTCTGGTTCAGCCGCTGGGTCTTATGGAATGGCATTTTAAAACTCTGAGC<br>TCGTTCGTTCACCAAATTGGAATGTATCGTCTGCATAGACCTATGGTCGGATTCAGTACCTTAGATTTAACGTCG<br>CACATTCTGAATATAACGCGTCAGGGAGAGAACGATCAA	[8]
palS	Codon optimized	ATGGGGAATTTGCGTGATTTCTACCAACTGATGAAAGATAACTATGCGGACTCTAATCTGTTCAAGGATTTGAAT<br>CTGATCCACAATATCTCCAACGACATCCAAATTGGAATTAATTGCGATTTCTCTGAAATGCTGGGAGAACTGGTA<br>GGTAATTACGATTCCCTGAACTATCCGTCAATCACCTGTGGTATTCTGACGTATAATGAAGAACGCTGCATTAAA<br>CGTTGTCTGGAAAGTGTGGTGAACGAATTCGATGAGATTATTGTCTTGGATAGTGTATCCGAGGACAATACCGTG<br>AAAATTATCAAGGAGAATTTCAACGATGTCAAAGTCTACGTCGAGCCATGGAAGAACGATTTTTCATTTCACCGC<br>AACAAGATCATTAATCTCGCAACGTGCGACTGGATCTACTTTATCGACGCGGATAATTATTATGATTCGAAGAAC<br>AAGGGTAAAGCCATGCGCATCGCTAAGGTTATGGATTTCTTGAAAATCGAAGGCGTTGTGAGCCCAACGGTCATT<br>GAGCATGACAATAGCATGAGCCGTGATACCCGTAAGATGTTTCGTCTGAAAGATAACATTCTGTTTAGCGGTAAA<br>GTTCATGAAGAACCGGTGTATGCCAATGGTGAGATCCCCCGGAACATCATAGTAGACATCAACGTGTTTCACGAC<br>GGCTATAACCCAAAGATTATCAACATGATGGAAAAGAACGAGCGCAATATCACCCTGACTAAAGAGATGATGAAG<br>ATCGAACCGAACAATCCGAAATGGCTGTACTTCTATAGCCGCGAACTCTATCAGACGCAACGTGACATTGCCCTT<br>GTGCAAAGTGTACTGTTCAAGGCACTGGAACTGTATGAAAACAGTTCATATACGCGTTATTATGTTGACACCATT<br>GCCTTACTGTGCCGAGTGCTGTTCGAATCTAAAAACTACCAGAAACTTACGGAATGTCTGAACATCCTGGAGAAC<br>AATACGCTTAACTGTTCCGATATCGATTACTATAATTCAGCGCTGCTGTTCTACAACCTGTTACTGCGCATCAAG<br>AAAATTAGCTCCACCCTGAAGGAGAACATTGATATGTACGAACGTGACTATCATAGCTTTATCAACCCCTCGCAT<br>GATCACATTAAGATTCTGATATTAAATATGCTCCTGCTGCTCGGCGATTACCAGGATGCCTTTAAGGTTTACAAG<br>GAGATCAAGTCCATTGAGATTAAAGATGAGTTTCTGGTGAACGTGAACAAATTCAAAGACAATCTTCTGAGCTTC<br>ATTGACTCCATTAACAAAATT	[12]
papB	Codon optimized	ATGGCAAACCTGATCCAGGACCGCGAGGACGAACTGATTCATTTCCATCCGTACAAACTGTTCGAGGTGGATTCA<br>AAAACCTTCTTCTATAACGTAGTCACCAACGCGATTTTTGAAATTGATAGCCTGATAATCGACATTCTTCACTCA<br>AAAGGTAAAAATGAGGAGCACGTTGTGAAAGATTTGGCTGAACGCTATGAGCTGTCTCAGGTTCGCGAAGCGATC<br>CAGAACATGAAAGAGGCATACATTATAGCAACCGATGCTAACATCTCCGACGTAGAGAAGATGGGTATCTTAGAT<br>AACTCGCAGCGCGTTTTTAAACTGTCTAGCCTGACGCTCTTTATGGTGCAGGAATGCAACCTGCGGTGTACGTAT<br>TGTTACGGCGAAGAAGGAGAATACAACCAGAAAGGTAAAATGACGTCCGAAATCGCCCGGAGCGCAGTGGATTTT<br>CTGATTCAACAGAGTGGTGAAATCGAACAGTTGAACATCACATTCTTTGGAGGCGAACCGCTGCTCAACTTTCCA<br>TTAATACAAGAAACCGTGCAGTATGTGCACGAACAGAGCGAGATCCATAACAAGAAATTTAGCTTTTCCATCACC<br>ACCAATGGCACGCTCATTACCCCCAAAATCAAAAACTTCTTCTATAAACACCACTTTGCAGTCCAGACTTCTATC<br>GATGGTGATGAAAAGACGCACAATTTCAATCGCTTCTTCAAAGGAGGCCAGGGCTCTTATGATCTGCTGTTAAAG<br>CGGACGGAAGAAATGCGCAATGACCGTAAAATTGGTGCACGTGGAACCGTGACCCCTGCCGAGCTGGACCTCTCA<br>AAATCATTTGACCACTTAGTTAAACTCGGCTTTCGCAAAATCTACTTATCACCCGCTTTATATAGTCTCTCTGAC<br>GATCACTACGACACCCTGAGCAAAGAGATGGTCAAACTTGTTGAACAATTCCGTGAGCTGCTGGAGCGTGAAGAT<br>TACGTCACCGCGAAGAAAATGTCTAATGTTCTGGGTATGTTATCGAAGATTCACTCCGGTGGCCCGCGCATTCAT<br>TTTTGCGGTGCCGGCACTAATGCTGCCGCTGTCGATGTCCGCGGCAACCTTTTCCCGTGTCATCGTTTCGTGGGT<br>GAAGATGAATGTTCAATCGGTAACCTGTTCGACGAGGACCCGCTGTCAAAACAGTACAACTTTATAGAGAATTCT<br>ACAGTACGCAACCGTACTACGTGTTCGAAATGCTGGGCGAAGAATCTGTGCGGCGGTGGTTGTCACCAAGAAAAT<br>TTCGCCGAGAATGGTAATGTGAACCAGCCAGTGGGCAAATTATGCAAAGTGACCAAAAACTTCATCAACGCGACC<br>ATCAATCTGTACTTGCAACTTACTCAAGAACAACGCAGCATTCTGTTCGGC	[40]
papoK	Codon optimized	ATGCACGATCGTAGCGCGAATGTTAGCTGGACCAAATACATCGCGTTTGGTCTGCGCATTGCCAGCGAACTCAAC<br>TTACCGGAACTGATATTGGCGGCTCCCGAAGCCGTTGAGGATGTTGTCATACGCCAGGCAGATCTCACGGCCTGG<br>TCTGGCCAACTTGAACAGGCAAATTTTGTCATGTTGGACGAACGTTTCATGTTTCAGATCCCGGGGACCGCCATT<br>TATGCGGTACGCGAAGGCAAAGAGATTGAAGTGAGCATCTTCTCTGGGGCCGACCCGGACACCGTGCGCCTTTTC<br>GTGCTGGGGACGTGCATGGGCGTGCTCTTGATGCAGCGCCGCATTCTGCCTATCCACGGCTCCGCCGTCGTTATC<br>GGTGGCCGCGCGTATGCCTTTGTTGGTGAATCAGGCACAGGTAAATCGACCTTAGCTGCAGCATTTCGGCAGGCC<br>GGTTACCAAATGGTTAGCGATGATGTCATTGCCGTCAAAGCGACCGCATCTAGCGCTATTGTTTACCCTGCGTAT<br>CCACAGCAAAAACTGGGTTTAGATTCGCTGTTGCAGCTTGAAGCGCTCCGTGAGAATAAGCACGCCCGCAAGCGT<br>AACAACATCCGTTCTCTGACGGATGGCAATAGTGTGATGCCGCAGTACAGCGATCTGCGCATGCTGGCGGGGGAA<br>CTGAATAAATATGCAGTTCCAGCCGTCGATGAATTCTTTAATGACCCGCTGCCGTTGGGCGGTGTTTTCGAACTG<br>GTAGCAGACAGTCCGATTCGAGCATTAATGCGCGAAGGCGAACTCGTCGCTGTGACCGAGCAACCGCTGAACGTT<br>CTGGAATGTTTACATACTCTTCTGCAACACACGTACCGTCGGGTAATCATCCCTCGAATGGGACTGAGCGAGTGG<br>AGCTTCGATACTGCGGCCCGAATGGCACGCAAGGTCGAGGGCTGGCGACTCCTCCGTGATAGCTCCGTGTTCACG<br>GCTAGTGAAGTCGTCCAGCGCGTCCTCGACATCATCCGTAAGGAGGAAAAGAGCTACGGATCACAC	[8]
pbtM1	Codon optimized	ATGCTGTCTAGCGCGCTGGAGGTGGATATCGATGAAGCTGCGGTGGCGGCGGACTTACGCGAATTGGCCGCAGCT<br>CTGGATCGCAGTGGTTATGGTGAAATCCTCACCTGTTTTCTGCCTCAGAAGGCACAGGCGCATATCTGGGCTCAG<br>ACCGCTGCAAAAATTGATGGGCCGTTGCGTACCCTGATGGAATTATTCCTTCTGGGTCGGGCGGTTCCCCAGGAT<br>GATCTCCCGCCTCGCATCGCGGCCGTGATTCCCGGTTTAGTTAGCGCAGGTCTGGTTAAGACTGGACAGGGCGCG<br>GTTTGGCTGCCGAACTTGATTCTGCTGCGTCCTATGGGCCAGTGGTTATGGTGTCAGCGGCCTCACCCCTCACCG<br>ACCATGTACTTTGGTGACGATAGCCTGGCGCTGGTTCACCGGATGGTAACATATCGTGGCGGCCGTGCCCTGGAT<br>TTATGTGCAGGTCCGGGTGTTCAGGCCCTTACCGCAGCCCTCCGCTCAGAGCACGTTACCGCGGTTGAGATCAAT	[38]

		CCGGTCGCGGCAGCCCTTTGCCGCACCAACATTGCCATGAACGGTCTGTCCGACCGCATGGAGGTTCGCCTGGGC<br>TCACTGTACGACGTCGTGCGCGGTGAGGTTTTTGATGATATTGTATCAAACCCGCCGCTGCTGCCTGTTCCGGAG<br>GATGTGCAATTCGCCTTTGTGGAGATGGCGGACGCGATGGTTTCGATATTTCTTGGACGATTCTGGATGGCCTG<br>CCTGAACATCTGTCCGACCGTGGTGCGTGTCGCATCGTTGGTTGTGTTCTGTCCGATGGCTATGTGCCTGTTGTG<br>ATGGAAGGCTTGGGAGAATGGGCCGCTAAACACGATTTCGACGTGCTTCTTACAGTGACCGCACATGTCGAGGCG<br>CATAAAGATAGTAGTTTTCTGCGTTCAATGAGCCTGATGAGTTCGGCGATCTCAGGCCGCCCAGCGGAGGAGCTG<br>CAAGAACGGTACGCAGCTGATTATGCCGAACTGGGCGGTTCCCACGTTGCGTTCTATGAACTGTGTGCCCGCCGT<br>GGTGGGGGTTCTGCACGTCTGGCCGACGTGAGCGCTACAAAACGCAGTGCGGAAGTGTGGTTTGTT	
pbtO	Codon optimized	ATGACCCAGTATCCCCTGTCGCGTCCAGAACCGCTGGGCGTGCACCCAGATTATCGTCGCCTGCGTGAGACTTGC<br>CCGGTTGCACGTGTGGGTAGCCCGTATGGCCCAGCGTGGCTTGTCACCCGTTACGCCGATGTGGCCGCAGTTCTG<br>ACCGATGCCCGTTTTAGTCGTGCAGCCGCTCCGGAAGATGATGGTGGCATCCTGCTGAACACCGATCCGCCGGAA<br>CATGATCGTCTGCGTAAACTGATTGTAGCACACACAGGCACCGCTCGCGTGGAACGGCTGCGTCCGCGTGCTGAA<br>GAGATCGCTGTTGCGTTAGCGCGCCGTATCCCGGGCGAAGGCGAATTCATTAGTGCATTTGCCGAGCCCTTCAGC<br>CATCGCGTTTTGTCTTTATTTGTTGGCCATCTTGTTGGGTTACCAGCGCAGGACCTGGGCCCCTTAGCGACCGTA<br>GTGACTCTGGCACCCGTTCCCGACCGCGAACGTGGCGCGGCATTTGCAGAGCTGTGTCGTCGGCTGGGTCGTCAG<br>GTGGATCGCGAAACGCTTGCAGTAGTTTTAAACGTGGTCTTTGGCGGACATGCGGCTGTAGTGGCCGCGCTGGGT<br>TATTGCCTGTTAGCTGCATTAGATGCGCCACTGCCACGTCTGGCCGGTGACCCAGAGGGCATTGCCGAACTGGTG<br>GAAGAAACCCTTCGTTTGGCTCCACCGGGAGATCGTACACTGTTGCGTCGTACTACAGAACCTGTGGAACTTGGC<br>GGTCGCACATTACCAGCGGGTGCGCTTGTAATCCCGTCCATTGCAGCCGCAAACCGTGATCCGGATCGCCCTGTG<br>GGCCGTCGTATGCCACGTCATCTTGCATTTGGACGTGGAGCGCATGCCTGTTTAGGCATGGCGCTGGCGCGCATG<br>GAACTCCAGGCAGCACTGAAAGCGTTAGCGGAACACGCGCCAGACGTACGGTTGCCGGCTGGTACAGGCGCGCTG<br>GTCCGCACACACGAAGAACTCTCGGTGAGCCCGCTCGCAGGAATCCCAATTCAACGC	[38]
pcpX	Codon optimized	ATGACATACCGTCGCACCTCCTATGCGGTATGGGAGATCACGCTGAAATGCAATCTGGCATGTTCGCACTGTGGA<br>AGTCGTGCCGGGCACACGCGAGCAAAAGAACTGTCCACACAGGAAGCGCTGGATCTGGTCCGTCAGATGGCTGAT<br>GTCGGCATTATCGAAGTTACTCTGATTGGGGGTGAAGCGTTCCTGCGTCCAGACTGGCTGCAGATTGCCGAGGCG<br>ATAACGAAAGCCGGGATGCTGTGCAGCATGACTACGGGCGGTTATGGCATATCGCTGGAAACCGCCCGCAAAATG<br>AAAGCGGCAGGAATCGCGAGCGTGAGCGTTAGCATCGATGGCTTGGAGGAAACCCATGATCGCTTACGCGGTCGC<br>AAAGGCTCTTGGCAGGCTGCGTTTAAAACAATGAGCCATTTGAGAGAAGTGGGCATCTTCTTTGGCTGTAACACC<br>CAGATTAACCGTCTGTCGGCCCCTGAATTTCCGCTGATATATGAACGCATCCGTGACGCCGGGGCACGTGCCTGG<br>CAGATCCAGCTTACGGTGCCGATGGGCCGCGCTGCCGATAACGCAAATATCCTTCTGCAACCGTACGAACTGCTT<br>GATCTGTATCCGATGATTGCTCGAGTGGCCCGCCGGGCCCGTCAAGAGGGCGTGCAAATCCAGCCAGGTAATAAT<br>ATTGGGTATTACGGCCCTTACGAACGTCTTTTACGTGGCCGGGGGAGCGATAGTGAGTGGGCATTTTGGCAGGGC<br>TGTGCCGCGGGCTTAAGTACCCTGGGTATTGAAGCGGATGGTGCTATAAAAGGTTGTCCCTCACTGCCAACGAGC<br>GCGTATACCGGCGGTAACATTCGCGAACATAGTCTGCGAGAAATAGTGGAAGAATCGGAACAGCTGCGTTTTAAC<br>CTCGGTGCAGGGACGAGCCAAGGGACCGCCCACTTGTGGGGCTTTTGCCAGACGTGTGAATTTAGTGAATTGTGC<br>AGAGGTGGTTGTACGTGGACAGCTCACGTGTTCTTTAACCGCCGTGGGAATAACCCGTATTGTCATCATCGGGCG<br>CTTTTCCAAGCGGAGCAGGGTATCAGAGAACGTGTCGTGCCAAAGGTCGAAGCTCAGGGCCTGCCGTTTGACAAC<br>GGTGAATTTGAACTTATCGAAGAACCTATTGACGCGCCTCTGCCCGAAAATGATCCACTGCACTTTACCAGCGAC<br>TTAGTGCAGTGGTCAGCGAGTTGGCAGGAAGAATCGGAATCTATAGGCGCAGTGGTAGAC	[19]
pcpY	Codon optimized	ATGGTGGAAAACATTGATAATGAACGTGAGAAAAGTGCGAACGAAATTGAACCGGAAAGCCTGCTTCTGCCGCGC<br>CAGGCTTGGCAGTCGCAGATCGCCTATCTTAAAGCGATTCTGAAAGCCAAACAGGCGCTTGACCGGATCGAAAAA<br>CGTTATCTGCGG	[19]
plpX	Codon optimized	ATGACCAAAAAGTATCGGCGTGTATCCTACGCAGTGTGGGAAATCACCCTGAAATGCAATCTGGCATGCTCTCAT<br>TGTGGCAGCCGCGCCGGCCAAGCCCGTACGAAAGAGCTGAGTACCGAAGAAGCGTTCAACCTGGTCCGCCAGCTG<br>GCCGACGTGGGCATTAAGGAAGTCACCCTGATCGGTGGTGAAGCCTTTATGCGTTCGGATTGGCTGGAAATCGCG<br>AAAGCCGTCACTGAAGCCGGCATGATCTGTGGCATGACCACAGGGGGCTTCGGGGTCAGTCTGGAAACGGCGCGT<br>AAAATGAAAGAAGCGGGCATTAAAACGGTGAGCGTTAGCATTGACGGTGGTATTCCTGAAACCCACGACCGCCAG<br>CGCGGTAAAAAGGGTGCGTGGCATAGTGCATTCCGGACTATGAGCCATCTGAAAGAAGTCGGGATCTACTTCGGT<br>TGCAACACTCAAATCAATCGTTTATCGGCGTCAGAATTCCCGATTATCTATGAACGTATTCGCGATGCTGGGGCA<br>CGTGCGTGGCAAATTCAGCTGACGGTTCCGATGGGCAACGCCGCGGATAACGCAGATATGCTGCTGCAACCGTAT<br>GAATTGCTCGACATCTATCCGATGTTAGCCCGCGTTGCCAAACGTGCGAAACAGGAAGGCGTGCGTATTCAGGCA<br>GGTAACAACATCGGGTACTATGGACCGTATGAGCGTCTGCTGCGTGGCAGCGACGAATGGACGTTTTGGCAAGGA<br>TGTGGTGCGGGCCTTAACACCCTCGGCATCGAAGCCGACGGCAAAATCAAAGGCTGTCCATCCCTGCCGACCGCC<br>GCGTACACCGGCGGTAACATTCGCGATCGCCCGCTGCGGGAAATCGTCGAACAGACCGAAGAACTGAAATTTAAC<br>TTAAAAGCTGGTACAGAACAAGGTACGGACCATATGTGGGGCTTTTGTAAAACCTGCGAATTCGCGGAACTCTGT<br>CGCGGCGGATGCAGCTGGACTGCGCATGTGTTCTTTGACCGGCGCGGCAATAATCCGTACTGCCACCATCGGGCT<br>CTGAAACAAGCCCAAAAAGACATTCGCGAACGCTTTTATTTAAAAGTGAAAGCAAAGGGCAACCCGTTCGACAAT<br>GGTGAATTTGTTATCATTGAAGAACCTTTTAACGCTCCGTTACCCGAGAATGACCTGCTGCACTTTAACAGTGAT<br>CACATTCAATGGCCAGAAAACTGGCAAAATAGTGAAAGCGCGTACGCATTGGCCAAG	[19]
plpY	Codon optimized	ATGAACAGTAATCAGATCCCTAACAAAGTTGCAACCGCGGCACAGAAATCTGACGACAGCAGCAGCGTATTACCG<br>CGCCAGGGGTGGCAAGACAAACAAGCCTTTATTAAGGCACTCATTAAAGCCAAACAGTCTCTCGAAATTGCCGAA<br>ATTAGCAACTTTTTAACC	[19]
procM	Codon optimized	ATGGAGAGTCCTAGCTCATGGAAAACATCGTGGCTGGCCGCCATCGCTCCGGATGAACCCCACAAATTCGACCGC<br>CGCTTAGAATGGGACGAGCTTTCAGAGGAGAACTTCTTCGCAGCACTGAACTCAGAACCTGCATCGTTGGAAGAG<br>GATGATCCATGTTTTGAAGAAGCACTGCAAGACGCCCTGGAGGCCTTGAAGGCAGCATGGGATTTACCCCTTCTT<br>CCCGTCGATAATAATCTTAATCGTCCCTTCGTAGATGTCTGGTGGCCCATTCGCTGTCACTCTGCGGAGAGCTTG<br>CGTCAAAGCTTCGTCAGTGATAGTGCTGGACTTGCGGACGAGATTTTTGATCAGCTGGCCGATTCGTTACTGGAC<br>CGTCTGTGCGCCCTGGGAGATCAGGTGTTGTGGGAGGCGTTTAACAAGGAGCGTACACCAGGAACGATGTTGTTA<br>GCCCACTTAGGAGCCGCAGGCGACGGCTCCGGACCCCCTGTACGTGAGCATTACGAACGTTTTATTCAGTCTCAC<br>CGCCGTAATGGATTAGCGCCTTTGCTTAAGGAATTCCCTGTACTGGGCCGCCTTATTGGAACAGTTTTGTCCCTT<br>TGGTTCCAAGGGAGCGTGGAAATGCTGCAACGTATCTGCGCTGACCGCACCGTTCTGCAACAGTGTTTCGCTATC<br>CCTTGCGGGCATCACCTGAAAACTGTAAAGCAGGGACTTTCTGATCCACACCGCGGCGGTCGCGCTGTGGCAGTT<br>TTGGAATTTGCGGACCCAAATTCCACCGCTAATTCAAGTATGCACGTAGTGTATAAACCGAAGGATATGGCTGTG<br>GATGCAGCTTACCAGGCCACCTTAGCAGATCTTAATACTCATAGCGACCTTTCCCCGTTGCGCACGCTTGCCATT<br>CATAACGGCAACGGATATGGTTACATGGAACATGTGGTTCACCATCTTTGCGCTAACGACAAAGAGCTGACAAAT<br>TTCTATTTCAACGCTGGGCGTTTAACCGCGCTTCTGCATCTTCTTGGATGTACTGACTGTCACCATGAAAATTTG<br>ATTGCATGTGGTGATCAATTACTGTTGATCGATACAGAAACATTATTGGAGGCGGATTTACCCGATCACATTTCG<br>GATGCTTCGAGCACCACGGCGCAACCAAAGCCTAGTAGCCTTCAAAAGCAATTTCAGCGTTCTGTTTTGCGTAGC<br>GGGTTACTTCCTCAATGGATGTTCCTGGGGGAGTCGAAGTTGGCCATCGACATCTCGGCTCTGGGAATGTCCCCA<br>CCCAATAAGCCTGAGCGTATTGCACTTGGCTGGTTAGGATTCAATTCTGACGGGATGATGCCTGGGCGTGTATCC<br>CAACCAGTTGAGATTCCTACATCCTTGCCCGTTGGGATTGGTGAGGTTAATCCCTTTGATCGTTTTTTAGAGGAT<br>TTTTGTGATGGCTTTTCCATGCAATCAGAGGCCCTTATTAAGCTTCGCAACCGTTGGCTGGACGTTAATGGGGTT<br>CTTGCTCATTTCGCGGGTCTGCCCCGCCGTATCGTTCTTCGCGCGACTCGCGTATACTTCACTATCCAGCGTCAG<br>CAGTTAGAGCCTACGGCACTGCGCTCTCCACTTGCACAGGCCTTGAAACTTGAGCAGCTTACTCGTTCTTTCTTG<br>TTGGCAGAGTCAAAGCCTCTTCACTGGCCCATTTTCGCAGCTGAAGTAAAGCAGATGCAGCACCTTGACATTCCT<br>TTCTTCACACACTTAATCGACGCTGACGCTCTGCAGCTGGGCGGCCTGGAACAAGAATTACCAGGCTTCATCCAG<br>ACTAGTGGCTTGGCAGCTGCTTACGAGCGTTTGCGTAATTTAGATACGGACGAGATTGCTTTCCAACTTCGTCTG<br>ATCCGCGGTGCAGTAGAGGCTCGCGAGTTGCATACTACGCCGGAGTCGAGCCCGACGTTGCCGCCGCCTGCCACC<br>CCCGAGGCTCTTATGTCCTCTTCAGCCGAGACTAGTTTAGAAGCTGCTAAGCGCATCGCTCACCGCTTACTGGAG<br>TTGGCAATTCGTGATTCTCAAGGGCAAGTAGAATGGCTGGGCATGGATCTGGGGGCAGATGGAGAGAGCTTCTCC<br>TTTGGCCCAGTTGGCTTGAGCCTTTATGGGGGCTCAATCGGTATCGCTCACCTTCTGCAACGTTTGCAGGCGCAG<br>CAAGTTTCCTTGATGGACGCAGACGCTATCCAAACGGCAATTTTACAGCCCCTTGTGGGACTGGTTGATCAACCT	[24]

		AGCGACGACGGACGTCGCCGTTGGTGGCGTGATCAGCCGCTGGGCTTAAGTGGATGTGGCGGTACCTTGCTTGCA<br>CTTACACTTCAAGGTGAACAAGCGATGGCTAATTCCCTGCTGGCCGCTGCTTTGCCCCGTTTTATCGAGGCTGAT<br>CAGCAACTTGACCTGATTGGTGGCTGCGCTGGACTGATCGGTTCGTTGGTACAATTAGGTACTGAAAGTGCCTTA<br>CAATTAGCTTTGCGTGCGGGCGACCATCTTATTGCGCAACAGAATGAAGAGGGGGCGTGGTCTAGCTCGTCATCA<br>CAGCCCGGTTTGTTGGGCTTTAGTCATGGTACTGCAGGTTACGCAGCAGCCTTAGCACACTTACATGCATTTTCC<br>GCTGATGAGCGTTACCGCACCGCAGCCGCTGCCGCTTTAGCATACGAACGCGCACGTTTTAATAAAGATGCCGGC<br>AACTGGCCAGACTACCGCTCGATCGGACGTGACTCTGATTCAGATGAACCGTCCTTTATGGCTTCCTGGTGTCAC<br>GGCGCACCCGGCATTGCCCTGGGCCGCGCCTGTTTGTGGGGTACGGCGCTTTGGGACGAAGAATGCACCAAGGAG<br>ATCGGAATTGGGTTACAGACCACAGCTGCTGTTTCGTCTGTTAGTACTGACCACCTGTGTTGTGGTTCACTTGGC<br>CTTATGGTATTATTAGAGATGCTGTCAGCAGGACCCTGGCCCATCGACAATCAATTACGTTCCCATTGCCAGGAC<br>GTAGCATTCCAGTACCGCCTGCAGGCTTTGCAGCGCTGTTCAGCCGAGCCGATTAAGCTTCGTTGCTTCGGTACA<br>AAAGAGGGCCTTTTAGTCCTGCCTGGATTTTTCACTGGCTTATCAGGAATGGGTTTAGCACTGCTTGAGGATGAT<br>CCATCTCGCGCCGTGGTTTCTCAACTGATCAGTGCGGGCTTATGGCCGACAGAG	
psnB	Codon optimized	ATGACGAATTTAGACACGAGCATTGTGGTCGTAGGAAGTCCGGATGATCTTCACGTCCAGTCAGTGACGGAGGGT<br>CTGCGTGCACGCGGTCACGAGCCTTACGTGTTTGACACCCAACGTTTTCCGGAAGAGATGACAGTGTCACTTGGT<br>GAACAGGGTGCCTCTATTTTTGTCGATGGCCAGCAAATTGCACGTCCGGCGGCGGTGTACCTCCGTTCACTGTAC<br>CAGAGCCCCGGCGCGTATGGGGTGGATGCCGACAAAGCGATGCAGGATAACTGGCGCCGCACATTGCTCGCTTTT<br>CGCGAGCGTAGTACCCTGATGAGCGCTGTGCTTCTGCGTTGGGAAGAAGCGGGGACTGCAGTGTATAATTCGCCA<br>CGCGCGTCGGCGAATATCACTAAACCGTTTCAGCTGGCGCTGCTGCGCGACGCTGGTCTGCCGGTACCACGTAGC<br>TTGTGGACAAACGACCCTGAAGCAGTGCGGCGGTTTCATGCGGAAGTGGGTGACTGTATTTACAAACCGGTCGCC<br>GGGGGAGCGCGTACACGCAAACTGGAAGCGAAAGATCTCGAAGCGGACCGCATCGAACGCCTGAGTGCAGCGCCG<br>GTGTGTTTTCAAGAACTGCTCACAGGAGATGATGTGCGTGTTTACGTGATAGATGACCAGGTAATATGCGCCCTG<br>CGCATCGTAACTGATGAGATCGATTTCCGCCAAGCAGAGGAACGTATCGAGGCCATCGAAATTTCAGATGAAGTA<br>AAAGACCAATGTGTACGTGCCGCCAAACTTGTTGGCCTGCGCTACACCGGTATGGATATCAAAGCCGGCGCCGAT<br>GGTAACTATCGTGTTCTCGAACTGAACGCGAGTGCGATGTTTCGCGGTTTCGAAGGCCGTGCGAATGTGGATATC<br>TGTGGACCGCTGTGTGATGCATTGATCGCTCAGACCAAACGT	[1]
raxST	Codon optimized. ST stands for SulfoTransferase and denotes a single gene, not two genes.	ATGGATTATCATTTCATCAGCGGACTGCCTCGTGCGGGGAGTTCATTACTGGCTGCGTTACTGCGTCAAAATCCG<br>CAGCTGCATGCCGATGTTACATCTCCGGTGGCGCGCCTTTACGCGGCCATGCTGATGGGTATGAGTGAAGAACAC<br>CCGAGCAACGTGCAGATTGACGATGCCCAACGTGTCCGTCTGTTACGTGCAGTATTTGATGCGTATTATCAGAAC<br>CGTCAGGAACTGGGGACAGTGTTCGATACTAACCGCGCATGGTGCTCTCGCCTCACGGGCCTGGCGCGTCTGTTT<br>CCGCGTAGTCGCATGATCTGCTGTGTACGCGATGTGGGCTGGATTGTTGATTCTTTTGAACGCCTGGCGCAGTCG<br>CAGCCGTTACGCCTTTCGGCCCTGTTCGGTTACGACCCCGAGGATTCGGTTAGCATGCACGCTGACTTACTCACT<br>GCGCCTCGCGGGGTAGTGGGCTACGCCCTGGATGGTTTACGTCAAGCGTTTTATGGAGATCACGCGGATCGTCTG<br>CTGTTGTTACGTTATGATACGCTGGCACAGCGTCCTGCACAAGCCATGGAACAGGTATATGCATTCCTGCAGCTC<br>CCTGCCTTTGCACATGATTATGCCGGTGTTCAGGCCGAAGCGGAACGCTTTGATGCCGCCCTGCAAATGCCTGGT<br>TTGCACCGCGTGCGTCGTGGTGTTCACTATGTTCCGCGACGTTCGGTTTTACCGCCTGCCCTGTTTGACCAGCTG<br>CAGGAACTTGCATTCTGGGAAAGTGCACCCAGCCATGGAGCGCTGCTCGTG	[17, 18]
sgbL	Codon optimized	ATGACAAGCCATGCAACCGAGGTTGAATGGGAGGACCTTCTGCGCCAAGCATTACACGCAACTGGTACAGGTGCT<br>CGTTGGGCTGTAGAGGCGGACGAGATGTGGTGCCGTGTCGCCCCGGTGCCTGGAACTCGCCGCGAGCAAGGATGG<br>AAGCTTCATGTAAGCGCGACGACCGCGAGTGCGCCCGAAGTCTTAACTCGTGCATTAGGCGTACTTCTGCGTGAA<br>AAGTCCGGGTTCAAATTTGCCCGCTCACTTGAACAAGTCTCGGCCTTGAATAGTCGTGCTACGCCCCGTGGTAGT<br>TCGGGTAAATTTATCACAGTATACCCCCGCTCAGACGCCGAAGCCGTCGCACTGGCTCGCGACCTGCATGCGGCA<br>ACGGCCGGCTTGGCTGGGCCCCGTATTCTTTCCGATCAACCATACGCCGCGCACAGCCTGGTGCATTATCGTTAT<br>GGGGCTTTCGTGGGACGTCGTCGCCTTTCAGATGACGGGCTTTTAGTTTGGTTTATTGAGGACCCAGATGGCAAT<br>CCCGTGGAGGATAAACGCACCGGACGTTATGCGCCGCCTCCCTGGGCTGTATGTCCGTTTCCTGCGAGCGTCCCC<br>GTTGCGCCCCATGACGGCGAAGCTACGAGTCGTCCTGTTGTCTTAGGTGGTCGCTTCGCGGTTCGTGAAGCCATC<br>CGTCAAACGAATAAAGGGGGCGTCTATCGCGGGTCGGACACACGCACTGGCACCGGCGTGGTTATCAAAGAGGCG<br>CGCCCACATGTTGAAGGAGACGCCAGTGGGGGCGATGTTCGTGACTGGCTTCGCGCAGAGGCGCGTACGCTTGAA<br>AAATTAAAAGGTACCGGCTTGGCACCAGAAGCGGTGGCGTTGTTTGAGCACGCTGGCCACTTGTTCTTAGCCCAA<br>GACGAGGTCCCGGGGGTTACGTTACGCACCTGGGTAGCGGAACACTTCCGTGACGTTGGAGGAGAGCGCTATCGT<br>GCCGACGCCCTGGCTCAGGTGGCTCGTTTAGTTGATTTAGTCGCGGCTGCTCATGCACGTGGCTTGGTCCTGCGC<br>GATTTTACACCAGGGAACGTGATGGTCCGTCCAGACGGCGAATTGCGCCTTATTGATTTAGAGCTGGCGGTTCTT<br>GAGGATGAGGCCGCATTGCCTACCCACGTCGGTACCCCGGGGTTTTCGGCACCCGAACGCCTTGCAGACGCTCCA<br>GTGCGTCCTACTGCTGACTACTATTCTCTGGGAGCCACAGCTTGTTTTGTCTTGGCCGGTAAAGTCCCTAATTTA<br>CTTCCTGAAGAACCCGTGGGTCGCCCATCGGAGGAGCGTCTTGCTGCCTGGTTGACTGCATGTACACGTCCGCTG<br>CGCCTGCCAGATGGAGTCGTTGACATGATCTTGGGGTTAATGCGCGATGATCCTGCAGAGCGCTGGGACCCATCC<br>CGCGCGCGTGAAGCACTGCGCAAAGCTGACCCGACAGCACGCCCCGGGGATGCTGATCGCACTGCAGTACGTCGT<br>ACGGGTTCGTCGGCAGTGGCCGGGCCAGTTCCTGACTCACGTACAGCAGATGGTCGTACAGCGGACGGCCGTTCC<br>GCGGATGAAGTTGTGGCAGGTCTTGTCGATCACTTAGTCGATAGTATGACCCCGGCAGATGATCGTCTGTGGCCG<br>GTAAGCACTCTTACGGGAGAATCGGATCCATGTACAGTCCAGCAAGGCGCTGCTGGGGTGCTTGCGGTGTTGACC<br>CGCTACTTCGAATTGACGGGCGATCCGCGCTTACCAGGCTTATTGTCGACAGCCGGACGTTGGATCGCAGACCGC<br>ACGGATGTTCGTTCACCTCGTCCGGGATTACATTTCGGGGGACGCGGAACAGCCTGGGCCTTATACGACGCGGGG<br>CGTGCAGTCGACGATCGTCGCTTGGTGGAACATGCTCTGGACTTAGCATTAGCCCCGCCCCAAGCGACTCCTCAT<br>CACGATGTCACGCATGGGACTGCGGGCTCAGGCTTAGCCGCCTTGCACCTGTGGCAGCGTACTGGAGATACTCGT<br>TTCGCGGATTTAGCAGTAGAGGCAGCTGATCGCTTAACAGCTGCAGCTCGTCGCGAGCCTTCGGGTGTTGGATGG<br>GCAGTACCTGCAGAGGCCGACTCCCCAGAAGGAGGCAAGCGTTACCTGGGCTTCGCTCATGGCGCAGCTGGGATT<br>GGGTGCTTCTTATTGGCTGCGGCGGAACTTAGTCGTCAACCCGATCATCGTGCAACTGCTTTGGAAGTTGGCGAA<br>GGCCTGGTTGCTGATGCTGTTCGCATCGGAGAGGCGGCACAGTGGCCTGCGCAATCCGGGGACTTGCCGACAGCG<br>CCTTACTGGTGCCATGGGGCGGCAGGTATCGGGACATTTCTTGTACGCTTATGGCAGGCGACCGGGGACGATCGC<br>TTCGGTGATCTGGCCCGCGGGAGTGCTCACGCTGTGGCCGAACGTGCTAGTCGCGCCCCATTGGCGCAATGTCAC<br>GGTTTGGCTGGAAACGGAGATTTCTTGTTGGATTTGGCAGACGCGACAGGCGATCCTGTGCATCGCGACACCGCG<br>GAAGAGTTAGCAGGGTTGATCTTGGCCGAAGGAACCCGTCGTCAGGGACATGTCGTTTTCCCTAATGAGTATGGG<br>GAAGTATCATCTTCATGGTCCGACGGTAGTGCGGGGATTCTTGCGTTCCTTCTGCGTACGCGTCATACGGGCCCT<br>CGCCATTGGATGGTAGAACAACGTGGG	[21]
stspM	Codon optimized	ATGGCGGATCATATTGCGGCCGGTCATGACACCGTCCTGAGCCTGGCCGAACGGACAGGTACCGATCCAGATCTG<br>CTGGGCCGTGTGTTGCGCTTCCTCGCTTGTCGTGGTGTTTTCGCCGAGCCTCGCCCAGGTACTTATGCCTTGACC<br>CCTCTGAGCTTAACTTTACTGGAAGGCCATCCGTCCGGTTTAAGAGAATGGTTGGATGCGTCGGGTGCGGGAGCG<br>CGCATGGACGCGGCAGTTGGAGATCTGCTTGGCGCCCTCCGCTCGGGTGAACCGAGCTATCCACGTCTGCATGGT<br>CGTCCGTTTTATGAAGATCTGGCGCTGCACAGCCGAGGCCCTGCTTTTGATGGACTGCGTCATACGCACGCCGAA<br>TCGTATGTTGCCGACCTGCTGGCAGCCTACCCGTGGGAACGCGTTCGTCGCGTGGTTGATGTAGGCGGTGGGACC<br>GGCGTATTGGTCGAGGCGCTTATGAGAACTCATGCGACCCTCCGTACAGTACTGGTCGATCTTCCAGGCGCGGTG<br>GCTACCGCTACCGCTCGAATTGCGGCTGCGGGTTTTGGCAATAGATATACACCGGTCACGGGTTCCTTCTTTGAT<br>CCGCTGCCTGCGGGGGCGGATGTTTACACCCTGGTTAACGTGGTTCACAACTGGAACGATGAGCGTGCCTCAGCT<br>CTGCTGCGTCGGTGTGCGGATGCGGGTCGCCGCGACAGTACGTTTGTTATCGTGGAACGCTTAGCGGACGATGCA<br>GACCCTCGTGCCATCACCGCCATGGACCTCCGTATGTTCCTTTTTCTGGGCGGTAAAGAGCGCACGGCCGCACAG<br>ATTCGCGAAGTAGCTAGTGCGGCTGGCATGGCCCACCAAAGCACCATTAAAACACCGTCTGGCCTCCACTTACTT<br>GTTTTCCGTAAGAAACGTTTCGCTGCTCGCGGTCACGGTCGTCGCATGGTGACC	[10]
tgnB	Codon optimized	ATGAAAACCATTCTGATTATTACCAATACCCTGGATCTGACCGTGGATTATATTATTAATCGCTATAATCATACC<br>GCTAAATTTTTTCGTCTGAATACCGATCGTTTTTTTGATTATGATATTAATATTACCAATAGCGGTACCAGCATT<br>CGTAATCGTAAATCTAATCTGATTATTAATATTCAGGAAATTCATAGCCTGTATTATCGCAAAATTACCCTGCCG<br>AATCTGGATGGCTATGAAAGTAAATATTGGACCCTGATGCAGCGCGAAATGATGAGTATTGTTGAAGGCATTGCA<br>GAAACCGCTGGCAATTTTGCACTGACCCGTCCGTCTGTGCTGCGCAAAGCTGATAATAAAATTGTGCAGATGAAA<br>CTGGCAGAAGAAATTGGTTTTATTCTGCCGCAGAGTCTGATTACCAATTCAAATCAGGCGGCAGCCTCATTTGC	[34]

		AATAAAAATAATACCAGCATTGTGAAACCGCTGAGTACCGGCCGCATTCTGGGTAAAAATAAAATTGGCATTATT<br>CAGACCAATCTGGTTGAAACCCATGAAAATATTCAGGGCCTGGAACTGTCTCCGGCTTATTTTCAGGATTATATT<br>CCGAAAGATACCGAAATTCGTCTGACCATTGTTGGTAATAAACTGTTTGGCGCCAATATTAAATCAACCAATCAG<br>GTTGATTGGCGCAAAAATGATGCACTGCTGGAATATAAACCGGCCAATATTCCGGATAAAATTGCCAAAATGTGT<br>CTGGAAATGATGGAAAAACTGGAAATTAATTTTGCGGCGTTTGATTTTATTATTCGTAATGGTGATTATATTTTT<br>CTGGAACTGAATGCCAATGGTCAGTGGCTGTGGCTGGAAGATATTCTGAAATTTGATATTTCAAATACCATTATT<br>AATTATCTGCTGGGTGAACCGATTTAA	
thcoK	Codon optimized	ATGACGAGAACCAACACCGGCTATCGTTATCGCGCGTTCGGCCTGCGCATAGACTCAGATATTCCGCTGCCAGAA<br>TTAGGGGACGGTACGCGCCCTGATGGTGACGCGGATCTGACGGTCGTCCGGTGTGGGGAAGCGGAGCCGGAATGG<br>GCTGAAGGTGGTGGCGGGGGTCGTCTGTATGCCGCTGAAGGCATTGTATCTTTTCGCGTGCCGCAGACGGCAGCG<br>TTCCGTATTACTAATGGAAATCGCATCGAGGTGCATGCCTACTCGGGGGCTGATGAGGATCGAATACGCCTGTAC<br>GTGTTAGGGACCTGTATGGGAGCGCTGTTACTGCAACGTAGAATCTTACCGCTTCATGGTTCGGTCGTCGCCCGT<br>GATGGTCGTGCGTATGCCATAGTTGGCGAAAGCGGAGCGGGCAAATCCACGATGAGTGCAGCACTTCTCGAACGT<br>GGATTCCGCCTCGTTACGGATGACGTGGCCGCCATCGTGTTCGATGAGCGTGGGACCCCACTGGTTATGCCGGCT<br>TATCCACAGCAAAAACTGTGGCAGGATTCCCTGGACCGTCTGCAAATTGCGGGCTCGGGCCTTCGTCCGCTGTTC<br>GAACGCGAAACGAAATACGCTGTACCCGCGGATGGGGCATTCTGGCCCGAACCGGTTCCATTGGTGCACATTTAC<br>GAACTGGTTCATAGCGATGGTCAAACGCCTGAACTGCAGCCGATTGCCAAATTAGAGCGTTGCTATACCTTGTAT<br>CGCCACACATTTCGTAGAAGCCTGATCGTCCCCAGCGGCTTAAGCGCCTGGCATTTTGAAACGGCAGTGAAACTT<br>GCGGAGAAAACGGGGATGTACCGTCTTATGCGCCCGGCCAAAGTTTTCGCGGCTCGCGAATCTGCTCGGCTGATT<br>GAAACTCACGCCGATGGTGAAGTGTCACGT	[9]
truD	Amplified from Topo-E1	ATGCAACCAACCGCCCTCCAAATTAAGCCCCACTTCCACGTTGAGATAATTGAGCCGAAGCAAGTGTATCTCCTG<br>GGCGAACAGGGCAACCACGCTCTCACCGGGCAGCTCTACTGCCAAATTCTGCCTTTCTTAAACGGCGAATACACC<br>CGAGAACAAATTGTGGAAAAGCTCGATGGGCAGGTCCCGGAGGAATATATCGACTTCGTACTCAGTCGTCTGGTG<br>GAGAAGGGCTATCTAACTGAGGTGGCTCCAGAACTATCCCTGGAAGTGGCAGCATTTTGGAGCGAATTGGGAATT<br>GCCCCTTCTGTAGTGGCAGAAGGGCTAAAGCAGCCAGTGACAGTGACAACGGCGGGCAAGGGCATTAGGGAAGGG<br>ATAGTGGCTAACCTGGCAGCAGCGCTGGAGGAAGCTGGCATTCAGGTGTCAGACCCAAGGGACCCAAAGGCCCCA<br>AAGGCAGGGGATTCTACTGCCCAGCTTCAGGTGGTGCTGACCGATGACTATTTACAGCCGGAACTTGCAGCGATC<br>AACAAGGAAGCCTTAGAGCGCCAACAACCCTGGTTGCTGGTTAAGCCTGTGGGCAGTATCCTCTGGTTGGGACCG<br>TTGTTCGTTCCTGGGGAAACCGGATGTTGGCACTGTCTTGCTCAACGATTGCAAGGCAACCGGGAAGTTGAAGCA<br>TCGGTATTGCAACAAAAGCGAGCGCTGCAGGAGCGCAACGGTCAAAATAAAAATGGTGCAGTGAGTTGCTTGCCC<br>ACAGCACGGGCAACCCTACCTTCTACTCTACAAACAGGTTTACAGTGGGCTGCCACTGAGATTGCTAAGTGGATG<br>GTCAAGCGGCACCTCAATGCCATAGCACCGGGAACGGCTCGTTTTCCCACTCTAGCTGGCAAGATATTTACATTC<br>AACCAGACGACTCTGGAGTTGAAAGCTCATCCTCTGAGCCGACGACCGCAATGTCCCACCTGTGGCGATCGGGAA<br>ACTCTCCAACGGCGCGGGTTTGAACCACTGAAGCTAGAGTCGCGCCCCAAACACTTCACCTCCGATGGCGGTCAT<br>CGCGCCATGACCCCAGAACAAACGGTGCAGAAGTACCAACACCTCATCGGGCCCATAACGGGGGTAGTGACGGAA<br>CTGGTGCGAATTTCTGACCCTGCCAATCCCTTGGTGCATACCTACCGGGCTGGGCATAGCTTTGGCAGTGCTACG<br>TCTCTGCGGGGGCTGCGCAATGTCCTACGCCACAAGAGTTCTGGTAAAGGCAAGACCGATAGCCAATCTCGGGCC<br>AGCGGACTTTGCGAGGCGATCGAGCGCTATTCGGGCATTTTTCAGGGAGACGAACCCCGCAAGCGGGCAACTTTG<br>GCTGAGTTGGGAGATTTGGCGATTCATCCAGAACAGTGTTTGCACTTTAGCGACAGGCAGTATGACAACCGGGAA<br>AGCTCGAACGAGCGAGCAACAGTGACTCACGACTGGATTCCCCAACGGTTCGATGCAAGTAAGGCTCACGACTGG<br>ACTCCCGTGTGGTCCCTAACGGAGCAAACCCATAAGTATCTGCCTACAGCCCTGTGCTATTACCGATACCCCTTC<br>CCCCCAGAACACCGTTTCTGCCGTAGTGACTCCAACGGAAACGCGGCGGGAAATACCCTGGAAGAGGCGATTTTG<br>CAAGGATTTATGGAACTGGTGGAACGGGATAGCGTGTGCCTGTGGTGGTACAATCGCGTTAGCCGTCCGGCTGTG<br>GATTTGAGTAGCTTTGACGAGCCTTATTTTTTGCAGTTGCAGCAGTTCTATCAAACTCAAAATCGCGATCTGTGG<br>GTACTGGATTTAACAGCAGATTTGGGCATTCCGGCTTTTGTAGGGGTATCGAATCGGAAAGCCGGCAGCTCGGAA<br>AGAATAATTCTCGGCTTTGGAGCGCACCTGGACCCGACAGTTGCCATCCTTCGCGCTCTTACGGAGGTCAACCAA<br>ATAGGCTTGGAATTGGATAAAGTTTCTGATGAGAGCCTCAAGAACGATGCCACGGATTGGTTAGTGAATGCTACA<br>TTGGCAGCTAGTCCCTATCTCGTTGCCGATGCTAGCCAACCCCTCAAGACTGCGAAGGATTATCCCCGGCGTTGG<br>AGTGACGATATTTACACCGATGTGATGACTTGTGTAGAAATAGCCAAGCAAGCAGGTCTAGAGACTTTGGTACTG<br>GATCAGACCAGACCCGACATAGGTTTAAATGTGGTTAAAGTCATTGTGCCAGGAATGCGTTTTTGGTCGCGATTT<br>GGCTCCGGTCGGCTCTATGACGTGCCAGTGAAGTTGGGATGGCGAGAGCAACCACTTGCTGAGGCACAAATGAAC<br>CCTACACCGATGCCATTT	[35]

Precursor peptides

Name	Details	Sequence	Source
albsA	Codon optimized	ATGGATTCACTGCTGTCAACAGAAACCGTCATTAGTGATGACGAACTGCTTCCGATTGAAGTTGGTGGTACCGCG<br>GAATTGACAGAGGGGCAGGGCGGCGGTCAGTCCGAGGATAAACGTCGCGCTTATAACTGC	[4]
amdnA	Codon optimized	ATGCCGGAAAATCGGCAGGAAGATCTCAACGCTCAGGCTGTACCATTCTTCGCGCGTTTCTTGGAGGGTCAAAAC<br>TGCGAGGACCTTACTGATGAGGAATCGGAGGCGGTTAGCGGTGGAAAACGCGGCCAAACCCGTAAATATCCAAGC<br>GACTGCGAAGATGGGAATGGCGTGACCGGTAAACTGCGCGATGAAGATATTGCAGTGACCTTGAAGTACCCATCC<br>GACAATGAAGATAATGGCGGCGGTGAAATTGTGACTCTGAAGTTTCCAAGTGATGATGATGATCAACCAGTAGGC	[32]
atxA1	Codon optimized	CCGATCATTAGCGAAACGGTCCAGCCTAAAACGGCTGGCCTGATTGTTCTGGGCAAGGCAAGCGCGGAAACGCGC<br>GGATTGAGCCAAGGCGTGGAACCGGACATTGGTCAGACGTACTTCGAAGAAAGCCGTATTAATCAGGAT	[5]
bamA	Codon optimized	CTGAAAATCCGCAAGGTGAAAATTGTCAGAGCGCAGAACGGCCACTACACGAAC	[13]
bmbC	Codon optimized	ATGGGTCCGGTTGTTGTGTTCGATTGCATGACGGCCGACTTTCTGAACGACGATCCAAATAACGCGGAGTTGTCT<br>GCCTTGGAAATGGAGGAGCTCGAGTCCTGGGGCGCCTGGGACGGAGAGGCTACCAGC	
bsjA2	Codon optimized	ATGACCAATGAAGAGATCATTGTCGCGTGGAAAAACCCTAAAGTCCGTGGCAAAAATATGCCAAGTCACCCGAGC<br>GGCGTGGGATTCCAAGAGCTTTCCATCAACGAGATGGCCCAAGTGACCGGCGGAGCAGTAGAACAGCGTGCAACA<br>CCAACCCTGGCAACCCCGCTGACCCCGCATACCCCGTACGCAACCTATGTGGTTAGCGGAGGCGTGGTTAGCGCG<br>ATTTCTGGTATCTTCAGCAACAATAAAACGTGTCTGGGC	[22]
bsjA3	Codon optimized	ATGACCAATGAGGAAATTATCGTTGCGTGGAAAAACCCGAAGGTGCGCGGCAAAAACATGCCTTCCCATCCGTCC<br>GGTGTGGGCTTCCAGGAATTATCTATTAATGAAATGGCACAGGTGACTGGTGGCGCGGTTGAACAGCGCGCGACG<br>CCGGCAACCCCAGCAACACCATGGCTGATTAAAGCGTCTTATGTGGTGAGTGGGGCGGGAGTTTCTTTTGTCGCA<br>AGCTATATCACTGTAAAC	[22]
capA	Codon optimized	ATGGTGCGTTTCCTGGCTAAGCTGCTGCGTTCAACGATCCATGGCTCTAATGGCGTGAGCCTCGACGCCGTCAGT<br>TCCACGCATGGTACTCCGGGGTTTCAGACACCTGATGCACGTGTTATTTCACGCTTTGGCTTTAAT	[3]
cinA	Codon optimized	ATGACGGCGAGTATTCTTCAGTCTGTCGTTGATGCGGACTTTCGTGCGGCCCTGATTGAAAACCCAGCCGCATTC<br>GGCGCGAGCACCGCAGTTTTGCCGACCCCAGTCGAACAGCAGGATCAGGCATCACTGGATTTTTGGACAAAAGAT<br>ATTGCTGCCACTGAGGCGTTTGCTTGCAAACAGTCTTGCTCATTTGGGCCGTTCACCTTTGTGTGCGACGGGAAT<br>ACCAAA	[27]
cln1A1	Codon optimized	ACTCCCATTCAATCCAAATTCTGCCTCCTGCGCGTGGGCAGTGCCAAACGGCTGACGCAGTCATTCGACGTGGGA<br>ACTATTAAGGAAGGTTTAGTCAGCCAGTATTATTTTGCG	[6]

cln1A2	Codon optimized	ACCCAGGTGAGCCCATCACCGCTGCGCCTGATTCGCGTCGGGAGAGCCTTGGACCTGACCCGCTCTATCGGGGAT<br>AGTGGGCTGCGTGAGTCCATGTCAAGCCAGACGTACTGGCCC	[6]
cln2A1	Codon optimized	AACACTTTAAAAACGCGTCTTATTCGCTTTGGGTCGGCTAAACGTCTGACGCGCGCAGGTACGGGCGTGCTGTTA<br>CCTGAAACCAACCAGATTAAGCGCTACGATCCAGCA	[6]
cln2A2	Codon optimized	ACCACACCCAAATTTCGACTGATTCGGTTAGGTTCAGCTAAGCGATTGACCCGGTCGGGAATCGGGGATGTGTTT<br>CCGGAGCCAAACATGGTTCGCCGCTGGGAT	[6]
cln3A1	Codon optimized	CAGCGTATAATAGATGAAACCACCGATGGTCTGATTGAACTGGGGGCGGCCAGCGTACAGACACAGGGCGATGTT<br>TTGTTTGCTCCGGAGCCTGGCGTGGGCCGACCTCCAATGGGCCTTTCCGAAGAT	[6]
cln3A2	Codon optimized	GAACGCATTGAAGATCATATTGATGATGAACTGATTGACCTGGGAGCTGCTTCGGTTGAAACCCAGGGAGATGTG<br>CTGAATGCACCGGAGCCTGGTATCGGTCGTGAACCGACAGGCTTGAGCCGCGAT	[6]
cln3A3	Codon optimized	GAATTTGAAGGTATCCCATCACCGGATGCGCGTATTGATTTGGGTCTGGCGTCGGAAGAAACCTGTGGTCAGATT<br>TATGATCACCCGGAAGTAGGCATCGGTGCGTACGGGTGCGAGGGCCTGCAGCGT	[6]
comX	Codon optimized	CAAGATCTGATTAATTACTTCCTGAATTATCCTGAGGCTCTGAAGAAACTCAAGAATAAGGAAGCCTGCTTAATT<br>GGGTTTGACGTCCAGGAAACCGAAACGATTATCAAAGCCTATAACGATTACTACCGCGCTGATCCGATCACGCGT<br>CAATGGGGTGAT	[14]
crnA1	Codon optimized	ATGTCCGAACTGAGTATGGAGAAAGTGGTCGGCGAAACATTTGAGGATCTGAGCATCGCGGAAATGACGATGGTG<br>CAGGGCAGCGGCGACATTAACGGCGAATTTACTACCTCGCCGGCATGTGTTTATTCCGTTATGGTTGTATCGAAA<br>GCAAGCAGCGCTAAATGTGCGGCCGGTGCATCGGCAGTCTCGGGAGCCATTCTGAGTGCGATTCGTTGC	[20]
crnA2	Codon optimized	ATGAGCGAATCCAACATGAAGAAGGTTGTTGGCGAAACCTTCGAAGATCTGAGCATCGCAGAAATGACGAAAGTT<br>CAGGGCTCAGGGGACGTGATGCCGGAATCTACCCCAATTTGTGCCGGCTTCGCAACCTTGATGAGTTCTATCGGT<br>CTTGTTAAAACCATCAAAGGCAATGTCAAAAGTTTCTCCGTCTTAATT	[20]
csegA1	Codon optimized	ACCAAGAAAAACGCAACACAGGCCCCACGTTTAGTACGTGTAGGCGATGCTCATCGTTTGACCCAAGGTGCTTTC<br>GTTGGACAGCCGGAAGCCGTAAATCCACTTGGACGTGAAATTCAAGGA	[7]
csegA2	Codon optimized	ACCAAAACACACAGACTGATCAGATTGGGCGACGCGCAACGCTTGACCCAGGGCACATTGACTCCGGGCTTACCG<br>GAGGACTTTCTGCCGGGCCATTACATGCCGGGG	[7]
csegA3	Codon optimized	ACTTCACGTTTCCAACTCCTGCGCCTGGGAAAAGCCGATCGTTTGACGCGTGGCGCGCTGGTCGGGCTCCTGATC<br>GAAGATATTACTGTCGCTCGCTACGACCCTATG	[7]
epiA	Codon optimized	GAAGCAGTTAAAGAGAAGAACGATCTGTTCAACCTGGATGTTAAAGTCAACGCAAAAGAAAGTAACGATAGTGGC<br>GCAGAACCACGCATAGCGTCGAAATTTATTTGCACACCAGGCTGCGCGAAAACGGGTTCGTTTAACAGCTATTGT<br>TGT	[30, 31]
halA1	Codon optimized	ACGAACTTGCTGAAAGAATGGAAAATGCCCCTGGAACGTACGCATAATAACTCCAACCCGGCGGGAGACATTTTT<br>CAGGAACTGGAAGATCAAGACATACTCGCCGGTGTGAATGGAGCAGAAAACTTATACTTTCAGGGTTGTGCGTGG<br>TATAACATTAGCTGCCGTCTGGGCAACAAAGGAGCCTACTGCACCCTTACAGTTGAGTGCATGCCCTCCTGTAAC	[28, 29]
halA2	Codon optimized	GTGAATTCCAAAGACCTGAGAAATCCAGAATTTCGCAAAGCTCAGGGTCTGCAGTTTGTAGATGAAGTTAATGAG<br>AAGGAACTCTCGAGTTTAGCCGGCAGCGAGAATCTTTACTTTCAAGGCACGACGTGGCCATGTGCGACCGTCGGC<br>GTTTCAGTTGCCTTGTGCCCGACGACCAAATGCACTTCACAGTGC	[28, 29]
kgpE	Codon optimized	AAGAACCCGACGCTGTTGCCCAAACTGACCGCGCCGGTCGAACGTCCGGCCGTAACTTCGTCGGATTTAAAGCAA<br>GCCTCAAGCGTCGATGCTGCATGGTTAAATGGCGATAATAACTGGTCAACCCCATTCGCCGGTGTGAACGCGGCA<br>TGGTTAAATGGGGACAACAACTGGTCCACGCCTTTTGCGGGCGTGAATGCTGCATGGCTTAATGGCGACAATAAC<br>TGGAGCACTCCATTTGCCGCCGATGGCGCTGAG	[37]
lasA	Codon optimized	ATGGACAAACGTGTGCGTTACGAAAAACCGAGCCTGGTGAAAGAGGGTACGTTTCGCAAAACTACCGCTGGCCTG<br>CGGCGTCTGTTCGCTGACCAGCTGGTTGGCCGCCGTAACATT	[2]
lcnA	Codon optimized	ACTAAAGGCCTGGACAAAATGCTTTTAACCAAAAAGAAGAAGGATAGTATGGGTCTGCTGAACGAAATCGACGTT<br>ACCACCCTGGATGAACAGTTAGGCGGTAAAATGAGCAAAGCATGGTGCCGATCCATGGTGGTGTCCTGCGTGTAT<br>AACCTGGTTGATTTTTCGTCGTCGAGTGACGGGAAAAAGACATGTGCTCTGTACCGCAAATATTGT	[11]
ltnA1	Codon optimized	ATGAATAAAAACGAAATCGAAACCCAGCCAGTTACGTGGCTGGAGGAAGTTTCTGATCAGAATTTTGATGAGGAT<br>GTCTTTGGTGCGTGTAGCACAAACACCTTCTCGCTGAGCGATTACTGGGGTAACAACGGTGCTTGGTGTACACTC<br>ACGCACGAATGTATGGCATGGTGCAAG	[23]
ltnA2	Codon optimized	ATGAAGGAAAAGAATATGAAGAAAAACGACACCATCGAACTTCAGCTTGGAAAATACCTGGAAGATGATATGATC<br>GAACTGGCTGAAGGGGATGAGTCCCATGGGGGTACTACCCCGGCTACCCCTGCGATTTCTATCCTCAGCGCGTAT<br>ATCAGCACCAATACCTGCCCGACAACTAAGTGTACACGCGCGTGC	[23]
mcbA	Synthesized, sequence from genome	ATGGAATTAAAAGCGAGTGAATTTGGTGTAGTTTTGTCCGTTGATGCTCTTAAATTATCACGCCAGTCTCCATTA<br>GGTGTTGGCATTGGTGGTGGTGGCGGCGGCGGCGGCGGCGGCGGTAGCTGCGGTGGTCAAGGTGGCGGTTGTGGT<br>GGTTGCAGCAACGGTTGTAGTGGTGGAAACGGTGGCAGCGGCGGAAGTGGTTCACATATC	*E. coli*<br>[25]
mdnA	Amplified from pARW071	ATGGCATATCCCAACGATCAACAAGGTAAAGCACTTCCTTTCTTTGCTCGTTTCTTGTCCGTAAGCAAAGAGGAA<br>TCTTCCATCAAGTCTCCTTCCCCTGAGCCTACCTACGGGGGCACCTTTAAATACCCTTCTGACTGGGAAGATTAT	[33]
*mdnA**	Amplified from mdnA	ATGGCACTTCCTTTCTTTGCTCGTTTCTTGTCCGTAAGCAAAGAGGAATCTTCCATCAAGTCTCCTTCCCCTGAG<br>CCTACCTACGGGGGCACCTTTAAATACCCTTCTGACTGGGAAGATTAT	[33]
mibA	Codon optimized	ATGCCAGCCGATATTCTGGAGACTCGTACCAGCGAAACGGAGGACTTACTGGATCTTGACCTGAGCATCGGTGTA<br>GAAGAAATCACCGCAGGCCCGGCAGTGACTTCTTGGTCACTGTGCACCCCTGGATGCACGAGTCCGGGCGGTGGC<br>TCCAATTGTTCGTTCTGTTGC	[26]
paaP	Codon optimized	ATGATTAAATTTTCTACATTGTCTCAGCGCATCAGCGCCATCACGGAAGAAAACGCCATGTACACTAAGGGTCAA<br>GTGATCGTATTGAGC	[15, 16]
padeA	Codon optimized	AAAAAGCAATATAGCAAACCTAGCCTGGAGGTTCTGGACGTCCACCAGACCATGGCTGGCCCGGGCACTAGTACG<br>CCAGACGCGTTTCAGCCAGATCCAGATGAAGATGTTCACTATGATTCG	[8]
palA	Codon optimized	AAAGATCTTCTGAAGGAACTGATGTATGAAGTAGACCTCGAAGAGATGGAGAATCTTCAGGGTAGCGGGTACTCA<br>GCCGCCCAGTGTGCCTGGATGGCGCTGAGCTGCGTCAATTACATCCCGGGAGTGGGATTCGGTTGTGGCGGCTAC<br>AGCGCATGTGAACTCTACAAGCGTTATTGT	[12]

papA	Codon optimized	ATGTTGAAACAGATCAATGTGATTGCTGGCGTAAAAGAGCCTATTCGCGCCTATGGTTGTTCGGCTAATGACGCA<br>TGCTATTTTTGCGACACGCGTGACAACTGCAAAGCCTGTGATGCCAGTGATTTTTGTATCAAAAGTGATACG	[40]
papA_tev	Codon optimized	TTGAAACAGATCAATGTGATTGCTGGCGTAAAAGAGCCTATTCGCGCCTATGAGAACTTGTATTTCCAGGGTTGT<br>TCGGCTAATGACGCATGCTATTTTTGCGACACGCGTGACAACTGCAAAGCCTGTGATGCCAGTGATTTTTGTATC<br>AAAAGTGATACG	
papoA	Codon optimized	AGCAAGAAAGAATGGCAAGAGCCCACGATCGAAGTGCTCGATATTAATCAGACTATGGCGGGTAAGGGCTGGAAA<br>CAGATAGACTGGGTGAGCGACCATGATGCTGACTTACACAATCCGTCT	[8]
pbtA	Codon optimized	ATGAACCTGAACGATTTACCTATGGACGTCTTTGAAATGGCAGACAGCGGTATGGAGGTGGAAAGCCTCACGGCT<br>GGCCATGGCATGCCAGAAGTTGGAGCTAGTTGCAACTGTGTGTGCGGGTTTTGCTGCAGCTGCAGTCCGAGCGCG	[38]
pcpA	Codon optimized	ATGTCGAGTAATATCCTCGAAAAAGTTAAGGAGTTTTTCGTCCGGCTGGTGAAGGATGATGCGTTTCAAAGCCAG<br>CTGCAGAACAACAGTATTGATGAAGTTCGAAATATCCTGCAGGAGGCCGGGTACATATTCAGCAAAGAAGAATTC<br>GAAACCGCAACCATTGAATTGCTGGATTTGAAGGAACGCGATGAATTCCACGAGCTGACAGAAGAGGAGCTTGTC<br>ACCGCTGTTGGCGGTGTTACGGGCGGGAGTGGTATATATGGCCCGATTCAAGCTATGTACGGTGCCGTCGTAGGT<br>GATCCAAAACCGGGTAAGGACTGGGGGTGGCGCTTTCCGAGCCCGCTGCCAAAACCGAGTCCGATTCCGAGTCCG<br>TGGAAACCCCCGGTTGATGTCCAGCCTATGTATGGTGTGGTAGTGTCAAACGATAGT	[19]
pgm2	Codon optimized	ATGGAGCGCGAAATCGTGTGGACAGAAATTGAGGAGTCGGATTTAGCCGCCGTCGTGTCGGCATCTAATGTCAAG<br>GATGGTCCAACCGTTAGCTCAAGTAATGTAAAGGACCGC	[47]
plpA1	Codon optimized	ATGAGCATTGAGAATGCCAAGAGCTTTTATGAACGCGTCAGTACAGATAAGCAGTTCCGCACTCAACTGGAAAAT<br>ACGGCCAGTGCTGAAGAACGGCAGAAAATCATTCAGGCAGCGGGCTTTGAGTTCACCAATCAGGAGTGGGAAATT<br>GCAAAAGAACAGATTCTTGCGACAAGTGAAAGTAATAACGGTGAACTGTCCGAGGCCGAACTGACCGCCGTCAGC<br>GGTGGGGTTGACTTAAGCATTTTCGAGCTGCTGGACGAAGAACCTTTATTCCCGATTCGTCCTTTGTACGGCCTG<br>CCTATT	[19]
plpA2	Codon optimized	ATGTCTATTGAGAGTGCAAAGGCTTTCTACCAGCGTATGACGGATGACGCATCTTTTCGTACCCCTTTTGAAGCG<br>GAACTGTCGAAAGAGGAGCGCCAACAATTAATCAAAGATAGCGGATATGACTTTACTGCAGAAGAATGGCAACAG<br>GCTATGACCGAGATCCAGGCGGCACGCTCAAACGAGGAACTGAATGAGGAAGAACTCGAGGCAATTGCCGGGGGC<br>GCTGTGGCCGCAATGTATGGTGTGGTTTTCCCATGGGACAACGAGTTCCCGTGGCCCCGCTGGGGCGGT	[19]
pqqA	Amplified from genome	ATGTGGAAGAAACCTGCTTTTATCGATTTACGTCTCGGTCTGGAAGTGACGCTGTACATTTCTAACCGT	*Klebsiella variicola* str. 342
*procA**	Codon optimized	ATGTCAGAAGAACAACTCAAGGCATTCATTGCCAAGGTTCAAGCAGACACTTCACTGCAGGAACAGCTCAAAGTA<br>GAAGGTGCTGATGTTGTTGCTATTGCTAAAGCCTCAGGGTTCGCGATTACCACAGAGGACCTCAATTCGCATCGC<br>CAAAATCTGTCTGATGATGAGCTGGAGGGAGTCGCGGGAGGCTTTTTCTGCGTACAGGGTACGGCCAACCGTTTC<br>ACTATCAACGTTTGC	[24]
procA1.7	Codon optimized	ATGTCAGAAGAACAACTCAAGGCATTCATTGCCAAGGTTCAAGCAGACACTTCACTGCAGGAACAGCTCAAAGTA<br>GAAGGTGCTGATGTTGTTGCTATTGCTAAAGCCTCAGGGTTCGCGATTACCACAGAGGACTTAAAAGCACATCAA<br>GCCAACTCACAAAAGAACCTGTCTGATGCTGAGCTGGAAGGTGTGGCTGGGCGAACCATTGGGGGAACCATTGTG<br>TCGATAACCTGTGAGACTTGCGATCTGCTTGTGGGGAAAATGTGC	[24]
psnA2	Codon optimized	ATGAGCAAAAATGAGAACAACAAGAAACAGCTGCGCGATCTTTTCATTGAAGATCTGGGCAAAGTTACTGGCGGT<br>AAAGGTGGCCCGTATACCACCTTAGCCATTGGCGAAGAAGATCCGATTACCACTTTGGCTATCGGAGAAGAGGAC<br>CCTGATCCAACGACACTTGCCTTAGGTGAAGAGGACCCAACTACGCTTGCAATCGGCGAAGAA	[1]
psnA2_tev	Codon optimized	ATGAGCAAAAATGAGAACAACAAGAAACAGCTGCGCGATCTTTTCATTGAAGATCTGGGCAAAGTTACTGGCGAG<br>AACTTGTATTTCCAGGGTAAAGGTGGCCCGTATACCACCTTAGCCATTGGCGAAGAAGATCCGATTACCACTTTG<br>GCTATCGGAGAAGAGGACCCTGATCCAACGACACTTGCCTTAGGTGAAGAGGACCCAACTACGCTTGCAATCGGC<br>GAAGAA	
raxX	Codon optimized	AACCACTCTAAGAAAAGTCCGGCAAAAGGGGCAGCGTCCCTGCAGCGTCCTGCTGGGGCAAAAGGCCGCCCTGAA<br>CCTCTGGATCAACGCTTGTGGAAACACGTCGGTGGTGGTGACTACCCACCCCCAGGAGCCAACCCAAAGCATGAT<br>CCACCACCCCGCAATCCGGGCCACCAT	[17, 18]
sboA	Amplified from genome	ATGAAAAAAGCTGTCATTGTAGAAAACAAAGGTTGTGCAACATGCTCGATCGGAGCCGCTTGTCTAGTGGACGGT<br>CCTATCCCTGATTTTGAAATTGCCGGTGCAACAGGTCTATTCGGTCTATGGGGA	ATCC 6633<br>[39]
sgbA	Codon optimized	TCTGGTCGCGGGCGCGATCCTGATGCTGCTGTACCTCCCTTGCCTCGTGTACCTCGCACTACTAATCATGAGCCA<br>CGTACGGCGTCCCGAGAACCAAGAGCAGCTCCAAGAACTGGACCTACACGTCCGCCTTCGTCGCGTCCATCTCCG<br>TGTGGTCACTCTCCTCAAACCCCTGGTGCAGGACGCAGTGGATGTCGTGTGGAGCGTCAAAAATCGGCTGCGGCT<br>TCGTCTGAGAAGGAAAAGACAATGGAGAACCAAGATTTGGAGTTATTAGCACGCCTGCATGCACTTCCTGAGACT<br>GAACCGGTGGGCGTCGACGGATTACCCTATGGCGAGACTTGTGAGTGCGTCGGGTTACTTACGTTGTTGAACACC<br>GTATGTATCGGCATTTCATGCGCT	[21]
strA	Codon optimized	ATGAGTAAGGAATTAGAAAAAGTTCTTGAATCCAGTTCAATGGCAAAGGGGGACGGCTGGAAGGTTATGGCTAAA<br>GGTGACGGTTGGGAG	[48]
stspA	Codon optimized	AAGAAATTCTATGAAGCGCCAGCTCTCATCGAACGTGGCGCCTTTGCGGCTGCTACAGCGGGGTTTGGACGTCTG<br>CTGGCGGATCAGCTGGTGGGACGCCTGATTCCG	[10]
tbtA	Codon optimized	ATGGACCTGAATGATCTGCCGATGGATGTTTTTGAACTGGCAGATAGCGGTGTTGCAGTTGAAAGCCTGACCGCA<br>GGTCATGGTATGACCGAAGTTGGTGCAAGCTGTAATTGCTTTTGTTATATTTGTTGTAGCTGCAGCAGCGCC	[49]
tfxA	Amplified from genome	ATGGATAACAAGGTTGCGAAGAATGTCGAAGTGAAGAAGGGCTCCATCAAGGCGACCTTCAAGGCTGCTGTTCTG<br>AAGTCGAAGACGAAGGTCGACATCGGAGGTAGCCGTCAGGGCTGCGTCGCT	ATCC 53912
*tgnA**	Codon optimized	TATCGACCTTATATTGCCAAGTATGTCGAAGAACAAACTCTGCAGAATTCAACCAACCTGGTATATGACGACATC<br>ACGCAGATCTCTTTTATCAATAAAGAAAAGAACGTGAAAAAAATTAATCTGGGTCCCGATACTACGATCGTGACT<br>GAAACCATCGAGAATGCGGACCCCGATGAGTATTTCTTA	[34]
thcoA	Codon optimized	CGCAAGAAAGAATGGCAGACACCAGAACTGGAAGTACTCGATGTACGCCTCACCGCAGCGGGCCCGGGTAAAGCT<br>AAACCGGATGCTGTGCAGCCAGACGAAGATGAAATAGTGCACTACTCA	[9]
*truE**	Codon optimized	ATGAACAAGAAGAACATTTTACCGCAGTTAGGACAACCAGTCATCCGCCTTACTGCCGGTCAACTGTCAAGCCAA<br>CTGGCGGAGCTTTCTGAGGAGGCTCTGGGAGGGGTCGATGCCTCGTACGCGGTGTTCTGGCCGATCTGTAGCTAT<br>GACGAC	

truE	Codon optimized	ATGAACAAGAAGAACATTTTACCGCAGTTAGGACAACCAGTCATCCGCCTTACTGCCGGTCAACTGTCAAGCCAA<br>CTGGCGGAGCTTTCTGAGGAGGCTCTGGGAGTCGATGCCTCGACCTTGCCGGTTCCGACGTTGTGTAGCTATGAC<br>GGGGTGGACGCTAGCACAGTCCCTACACTTTGTAGTTACGATGAC	[35]
truE_TEV	Codon optimized	AACAAGAAGAACATTTTACCGCAGTTAGGACAACCAGTCATCCGCCTTACTGCCGGTCAACTGTCAAGCCAACTG<br>GCGGAGCTTTCTGAGGAGGCTCTGGGAGAGAACTTGTATTTCCAGGGTGTCGATGCCTCGACCTTGCCGGTTCCG<br>ACGTTGTGTAGCTATGACGGGGTGGACGCTAGCACAGTCCCTACACTTTGTAGTTACGATGAC	

Plasmid origins

Name	Details	Sequence	Source
pSC101	var2 – maintains at p15A-level copy number	AGTAAGACGGGTAAGCCTGTTGATGATACCGCTGCCTTACTGGGTGCATTAGCCAGTCTGAATGACCTGTCACGG<br>GATAATCCGAAGTGGTCAGACTGGAAAATCAGAGGGCAGGAACTGCTGAACAGCAAAAAGTCAGATAGCACCACA<br>TAGCAGACCCGCCATAAAACGCCCTGAGAAGCCCGTGACGGGCTTTTCTTGTATTATGGGTAGTTTCCTTGCATG<br>AATCCATAAAAGGCGCCTGTAGTGCCATTTACCCCCATTCACTGCCAGAGCCGTGAGCGCAGCGAACTGAATGTC<br>ACGAAAAAGACAGCGACTCAGGTGCCTGATGGTCGGAGACAAAAGGAATATTCAGCGATTTGCCCGAGCTTGCGA<br>GGGTGCTACTTAAGCCTTTAGGGTTTTAAGGTCTGTTTTGTAGAGGAGCAAACAGCGTTTGCGACATCCTTTTGT<br>AATACTGCGGAACTGACTAAAGTAGTGAGTTATACACAGGGCTGGGATCTATTCTTTTTATCTTTTTTTATTCTT<br>TCTTTATTCTATAAATTATAACCACTTGAATATAAACAAAAAAAACACACAAAGGTCTAGCGGAATTTACAGAGG<br>GTCTAGCAGAATTTACAAGTTTTCCAGCAAAGGTCTAGCAGAATTTACAGATACCCACAACTCAAAGGAAAAGGA<br>CTAGTAATTATCATTGACTAGCCCATCTCAATTGGTATAGTGATTAAAATCACCTAGACCAATTGAGATGTATGT<br>CTGAATTAGTTGTTTTCAAAGCAAATGAACTAGCGATTAGTCGCTATGACTTAACGGAGCATGAAACCAAGCTAA<br>TTTTATGCTGTGTGGCACTACTCAACCCCACGATTGAAAACCCTACAAGGAAAGAACGGACGGTATCGTTCACTT<br>ATAACCAATACGCTCAGATGATGAACATCAGTAGGGAAAATGCTTATGGTGTATTAGCTAAAGCAACCAGAGAGC<br>TGATGACGAGAACTGTGGAAATCAGGAATCCTTTGGTTAAAGGCTTTTGGATTTTCCAGTGGACAAACTATGCCA<br>AGTTCTCAAGCGAAAAATTAGAATTAGTTTTTAGTGAAGAGATATTGCCTTATCTTTTCCAGTTAAAAAAATTCA<br>TAAAATATAATCTGGAACATGTTAAGTCTTTTGAAAACAAATACTCTATGAGGATTTATGAGTGGTTATTAAAAG<br>AACTAACACAAAAGAAAACTCACAAGGCAAATATAGAGATTAGCCTTGATGAATTTAAGTTCATGTTAATGCTTG<br>AAAATAACTACCATGAGTTTAAAAGGCTTAACCAATGGGTTTTGAAACCAATAAGTAAAGATTTAAACACTTACA<br>GCAATATGAAATTGGTGGTTGATAAGCGAGGCCGCCCGACTGATACGTTGATTTTCCAAGTTGAACTAGATAGAC<br>AAATGGATCTCGTAACCGAACTTGAGAACAACCAGATAAAAATGAATGGTGACAAAATACCAACAACCATTACAT<br>CAGATTCCTACCTACATAACGGACTAAGAAAAACACTACACGATGCTTTAACTGCAAAAATTCAGCTCACCAGTT<br>TTGAGGCAAAATTTTTGAGTGACATGCAAAGTAAGTATGATCTCAATGGTTCGTTCTCATGGCTCACGCAAAAAC<br>AACGAACCACACTAGAGAACATACTGGCTAAATACGGAAGGATCTGAGGTTCTTATGGCTCTTGTATCTATCAGT<br>GAAGCATCAAGACTAACAAACAAAAGTAGAACAACTGTTCACCGTTACATATCAAAGGGAAAACTGTCCATATGC<br>ACAGATGAAAACGGTGTAAAAAAGATAGATACATCAGAGCTTTTACGAGTTTTTGGTGCATTCAAAGCTGTTCAC<br>CATGAACAGATCGACAATGTAACAGATGAACAGCATGTAACACCTAATAGAACAGGTGAAACCAGTAAAACAAAG<br>CAACTAGAACATGAAATTGAACACCTGAGACAACTTGTTACAGCTCAACAGTCACACATAGACAGCCTGAAACAG<br>GCGATGCTGCTTATCGAATCAAAGCTGCCGACAACACGGGAGCCAGTGACGCCTCCCGTGGGGAAAAAATCATGG<br>CAATTCTGGAAGAAATAGCGCTTTCAGCCGGCAAACCGGCTGAAGCCGGATCTGCGATTCTGATAACAAACTAGC<br>AACACCAGAACAGCCCGTTTGCGGGCAGCAAAACCCGTAC	[50]
p15A		TTAATAAGATGATCTTCTTGAGATCGTTTTGGTCTGCGCGTAATCTCTTGCTCTGAAAACGAAAAAACCGCCTTG<br>CAGGGCGGTTTTTCGAAGGTTCTCTGAGCTACCAACTCTTTGAACCGAGGTAACTGGCTTGGAGGAGCGCAGTCA<br>CCAAAACTTGTCCTTTCAGTTTAGCCTTAACCGGCGCATGACTTCAAGACTAACTCCTCTAAATCAATTACCAGT<br>GGCTGCTGCCAGTGGTGCTTTTGCATGTCTTTCCGGGTTGGACTCAAGACGATAGTTACCGGATAAGGCGCAGCG<br>GTCGGACTGAACGGGGGGTTCGTGCATACAGTCCAGCTTGGAGCGAACTGCCTACCCGGAACTGAGTGTCAGGCG<br>TGGAATGAGACAAACGCGGCCATAACAGCGGAATGACACCGGTAAACCGAAAGGCAGGAACAGGAGAGCGCACGA<br>GGGAGCCGCCAGGGGAAACGCCTGGTATCTTTATAGTCCTGTCGGGTTTCGCCACCACTGATTTGAGCGTCAGAT<br>TTCGTGATGCTTGTCAGGGGGGCGGAGCCTATGGAAAAACGGCTTTGCCGCGGCCCTCTCACTTCCCTGTTAAGT<br>ATCTTCCTGGCATCTTCCAGGAAATCTCCGCCCCGTTCGTAAGCCATTTCCGCTCGCCGCAGTCGAACGACCGAG<br>CGTAGCGAGTCAGTGAGCGAGGAAGCGGAATATATCCTGTATCACATATTCTGCTGACGCACCGGTGCAGCCTTT<br>TTTCTCCTGCCACATGAAGCACTTCACTGACACCCTCATCAGTGCCAACATAGTAAGCCAGTATACACTCCGCTA	Unknown
