# Supplementary material for: Functional expression of diverse post-translational peptide-modifying enzymes in Escherichia coli under uniform expression and purification conditions
Source: PLoS One. 2022 Sep 19;17(9):e0266488. doi: 10.1371/journal.pone.0266488 (PMC9484694; doi:10.1371/journal.pone.0266488)
Supplement: S8 Table — (PDF) [file pone.0266488.s019.pdf]

S8 Table: Plasmid Sequences

Name[a]	Description	Sequence[b,c]
**N-term MBP-Tag**		
pEG3017	HIS_6-MBP-TruE*	CATTTCCCCGAAAAGTGCCACCTGACGTCTAAGAAACCATTATTATCATGACATTAACCTATAAAAATAGGCGTATCACGAGGCAGAATTTCAGATAAAA<br>AAAATCCTTAGCTTTCGCTAAGGATGATTTCTGGAATTCGCGGCCGCTTCTAGAGCGCATCCCGAAAATTTATCAAAAAGAGTATTGACTTAAAGTCTAA<br>CCTATAGGATACTTACAGCCATCGAGAGCTGCGAGATTCCACCATCAAAACACGGAGAGTAGCCCACATGTCATATTACCACCATCACCATCATCACGAC<br>TATGATATTCCCACAAGCATGAAAATCGAAGAAGGTAAACTGGTAATCTGGATTAACGGCGATAAAGGCTATAACGGATTGGCTGAAGTCGGTAAGAAAT<br>TCGAGAAAGATACCGGAATTAAAGTCACCGTTGAGCATCCGGATAAACTGGAAGAGAAATTCCCACAGGTTGCGGCAACTGGCGATGGCCCTGACATTAT<br>CTTCTGGGCACACGACCGCTTTGGTGGCTACGCTCAATCTGGCCTGTTGGCTGAAATCACCCCGGACAAAGCGTTCCAGGACAAGCTGTATCCGTTTACC<br>TGGGATGCCGTACGTTACAACGGCAAGCTGATTGCTTACCCGATCGCTGTTGAAGCGTTATCGCTGATTTATAACAAAGATCTGCTGCCGAACCCGCCAA<br>AAACCTGGGAAGAGATCCCGGCGCTGGATAAAGAACTGAAAGCGAAAGGTAAGAGCGCGCTGATGTTCAACCTGCAAGAACCGTACTTCACCTGGCCGCT<br>GATTGCTGCTGACGGGGGTTATGCGTTCAAGTATGAAAACGGCAAGTACGACATTAAAGACGTGGGCGTGGATAACGCTGGCGCGAAAGCGGGTCTGACC<br>TTCCTGGTTGACCTGATTAAAAACAAACACATGAATGCAGACACCGATTACTCCATCGCAGAAGCTGCCTTTAATAAAGGCGAAACAGCGATGACCATCA<br>ACGGCCCGTGGGCATGGTCCAACATCGACACCAGCAAAGTGAATTATGGTGTAACGGTACTGCCGACCTTCAAGGGTCAACCATCCAAACCGTTCGTTGG<br>CGTGCTGAGCGCAGGTATTAACGCCGCCAGTCCGAACAAAGAGCTGGCGAAAGAGTTCCTCGAAAACTATCTGCTGACTGATGAAGGTCTGGAAGCGGTT<br>AATAAAGACAAACCGCTGGGTGCCGTAGCGCTGAAGTCTTACGAGGAAGAGTTGGCGAAAGATCCACGTATTGCCGCCACCATGGAAAACGCCCAGAAAG<br>GTGAAATCATGCCGAACATCCCGCAGATGTCCGCTTTCTGGTATGCCGTGCGTACTGCGGTGATCAACGCCGCCAGCGGTCGTCAGACTGTCGATGAAGC<br>CCTGAAAGACGCGCAGACTCGTATCACCAAGTCGTACTACCATCACCATCACCATCACGGCGGTAGTGGCGAAAACCTGTATTTTCAGGGTATGAACAAG<br>AAGAACATTTTACCGCAGTTAGGACAACCAGTCATCCGCCTTACTGCCGGTCAACTGTCAAGCCAACTGGCGGAGCTTTCTGAGGAGGCTCTGGGAGGGG<br>TCGATGCCTCGTACGCGGTGTTCTGGCCGATCTGTAGCTATGACGACTAATAATTCAGCCAAAAAACTTAAGACCGCCGGTCTTGTCCACTACCTTGCAG<br>TAATGCGGTGGACAGGATCGGCGGTTTTCTTTTCTCTTCTCAACTGTACTAGTAGCGGCCGCTGCAGTCCGGCAAAAAAGGGCAAGGTGTCACCACCCTG<br>CCCTTTTTCTTTAAAACCGAAAAGATTACTTCGCGTTATGCAGGCTTCCTCGCTCACTGACTCGCTGCGCTCGGTCGTTCGGCTGCGGCGAGCGGTATCA<br>GCTCACTCAAAGGCGGTAATGACAGTAAGACGGGTAAGCCTGTTGATGATACCGCTGCCTTACTGGGTGCATTAGCCAGTCTGAATGACCTGTCACGGGA<br>TAATCCGAAGTGGTCAGACTGGAAAATCAGAGGGCAGGAACTGCTGAACAGCAAAAAGTCAGATAGCACCACATAGCAGACCCGCCATAAAACGCCCTGA<br>GAAGCCCGTGACGGGCTTTTCTTGTATTATGGGTAGTTTCCTTGCATGAATCCATAAAAGGCGCCTGTAGTGCCATTTACCCCCATTCACTGCCAGAGCC<br>GTGAGCGCAGCGAACTGAATGTCACGAAAAAGACAGCGACTCAGGTGCCTGATGGTCGGAGACAAAAGGAATATTCAGCGATTTGCCCGAGCTTGCGAGG<br>GTGCTACTTAAGCCTTTAGGGTTTTAAGGTCTGTTTTGTAGAGGAGCAAACAGCGTTTGCGACATCCTTTTGTAATACTGCGGAACTGACTAAAGTAGTG<br>AGTTATACACAGGGCTGGGATCTATTCTTTTTATCTTTTTTTATTCTTTCTTTATTCTATAAATTATAACCACTTGAATATAAACAAAAAAAACACACAA<br>AGGTCTAGCGGAATTTACAGAGGGTCTAGCAGAATTTACAAGTTTTCCAGCAAAGGTCTAGCAGAATTTACAGATACCCACAACTCAAAGGAAAAGGACT<br>AGTAATTATCATTGACTAGCCCATCTCAATTGGTATAGTGATTAAAATCACCTAGACCAATTGAGATGTATGTCTGAATTAGTTGTTTTCAAAGCAAATG<br>AACTAGCGATTAGTCGCTATGACTTAACGGAGCATGAAACCAAGCTAATTTTATGCTGTGTGGCACTACTCAACCCCACGATTGAAAACCCTACAAGGAA<br>AGAACGGACGGTATCGTTCACTTATAACCAATACGCTCAGATGATGAACATCAGTAGGGAAAATGCTTATGGTGTATTAGCTAAAGCAACCAGAGAGCTG<br>ATGACGAGAACTGTGGAAATCAGGAATCCTTTGGTTAAAGGCTTTTGGATTTTCCAGTGGACAAACTATGCCAAGTTCTCAAGCGAAAAATTAGAATTAG<br>TTTTTAGTGAAGAGATATTGCCTTATCTTTTCCAGTTAAAAAAATTCATAAAATATAATCTGGAACATGTTAAGTCTTTTGAAAACAAATACTCTATGAG<br>GATTTATGAGTGGTTATTAAAAGAACTAACACAAAAGAAAACTCACAAGGCAAATATAGAGATTAGCCTTGATGAATTTAAGTTCATGTTAATGCTTGAA<br>AATAACTACCATGAGTTTAAAAGGCTTAACCAATGGGTTTTGAAACCAATAAGTAAAGATTTAAACACTTACAGCAATATGAAATTGGTGGTTGATAAGC<br>GAGGCCGCCCGACTGATACGTTGATTTTCCAAGTTGAACTAGATAGACAAATGGATCTCGTAACCGAACTTGAGAACAACCAGATAAAAATGAATGGTGA<br>CAAAATACCAACAACCATTACATCAGATTCCTACCTACATAACGGACTAAGAAAAACACTACACGATGCTTTAACTGCAAAAATTCAGCTCACCAGTTTT<br>GAGGCAAAATTTTTGAGTGACATGCAAAGTAAGTATGATCTCAATGGTTCGTTCTCATGGCTCACGCAAAAACAACGAACCACACTAGAGAACATACTGG<br>CTAAATACGGAAGGATCTGAGGTTCTTATGGCTCTTGTATCTATCAGTGAAGCATCAAGACTAACAAACAAAAGTAGAACAACTGTTCACCGTTACATAT<br>CAAAGGGAAAACTGTCCATATGCACAGATGAAAACGGTGTAAAAAAGATAGATACATCAGAGCTTTTACGAGTTTTTGGTGCATTCAAAGCTGTTCACCA<br>TGAACAGATCGACAATGTAACAGATGAACAGCATGTAACACCTAATAGAACAGGTGAAACCAGTAAAACAAAGCAACTAGAACATGAAATTGAACACCTG<br>AGACAACTTGTTACAGCTCAACAGTCACACATAGACAGCCTGAAACAGGCGATGCTGCTTATCGAATCAAAGCTGCCGACAACACGGGAGCCAGTGACGC<br>CTCCCGTGGGGAAAAAATCATGGCAATTCTGGAAGAAATAGCGCTTTCAGCCGGCAAACCGGCTGAAGCCGGATCTGCGATTCTGATAACAAACTAGCAA<br>CACCAGAACAGCCCGTTTGCGGGCAGCAAAACCCGTACCGATTATCAAAAAGGATCTTCACCTAGATCCTTTTAAATTAAAAATGAAGTTTTAAATCAAT<br>CTAAAGTATATATGAGTAAACTTGGTCTGATTACGCCCCGCCCTGCCACTCATCACAGTACTGTTGTAATTCATTAAGCATGCGGCCGACATGGAAGCCA<br>TCACAAACGGCATGATGAACCTGGATCGCCAGCGGCATCAGCACCTTGTCGCCTTGCGTATAATATTTGCCCATCGTGAAAACGGGGGCGAAGAAGTTGT<br>CCATATTGGCCACGTTTAAATCAAAACTGGTGAAACTCACCCAGGGATTGGCTGAGACAAAAAACATATTCTCAATAAACCCTTTAGGGAAATAGGCCAG<br>GTTTTCACCGTAACACGCCACATCTTGCGAATATATGTGGAGAAACTGCCGGAAATCGTCGTGGTATTCACTCCAGAGGGACGAAAACGTTTCAGTTTGC<br>TCATGGAAAACGGTGTAACAAGGGTGAACACTATCCCAGATCACCAGCTCACCGTCTTTCATGGCCATACGAAACTCCGGGTGAGCGTTCATCAGGCGGG<br>CAAGAATGTGAATAAAGGCCGGATAAAACTTGTGCTTATTTTTCTTTACGGTCTTTAAAAAGGCCGTAATATCCAGCTGAACGGTCTGGTTATAGGTACA<br>TTGAGCAACTGACTGAAATGCCTCAAAATGTTCTTTACGATGCCATTGGGATATATCAACGGTGGTATATCCCGTGATTTTTTTCTCCATACTCTTCCTT<br>TTTCAATATTATTGAAGCATTTATCAGGGTTATTGTCTCATGAGCGGATACATATTTGAATGTATTTAGAAAAATAAACAAATAGGGGTTCCGCG
**N-term HIS_6-Tag with ATag-1**		
Backbone Sequence		CACATTTCCCCGAAAAGTGCCACCTGACGTCTAAGAAACCATTATTATCATGACATTAACCTATAAAAATAGGCGTATCACGAGGCAGAATTTCAGATAA<br>AAAAAATCCTTAGCTTTCGCTAAGGATGATTTCTGGAATTCGCGGCCGCTTCTAGAGGGAGAACGATCGTTGGCTGAATCATAAAAAATTTATTTGCTTT<br>GTGAGCGGATAACAATTATAATAGATTCAATTGTGAGCGGATAACAATTAGAACCCAACACCACCAGCAAGCCTAAGGAGGAGAAATATGTCATATTACC<br>ACCATCACCATCATCACGACTATGATATTCCCACAAGCGAGAACTTGTACTTTCAAGGGATGAGCAAAGGAGAAGAACTTTTCACTGGAGTTGTCCCAAT<br>TCTTGTTGAATTAGATGGTGATGTTAATGGGCACAAATTTTCTGTCCGTGGAGAGGGTGAAGGTGATGCTACAAACGGAAAACTCACCCTTAAATTTATT<br>TGCACTACTGGAAAACTACCTGTTCCGTGGCCAACACTTGTCACTACTCTGACCTATGGTGTTCAATGCTTTTCCCGTTATCCGGATCACATGAAACGGC<br>ATGACTTTTTCAAGAGTGCCATGCCCGAAGGTTATGTACAGGAACGCACTATATCTTTCAAAGATGACGGGACCTACAAGACGCGTGCTGAAGTCAAGTT<br>TGAAGGTGATACCCTTGTTAATCGTATCGAGTTAAAGGGTATTGATTTTAAAGAAGATGGAAACATTCTTGGACACAAACTCGAGTACAACTTTAACTCA<br>CACAATGTATACATCACGGCAGACAAACAAAAGAATGGAATCAAAGCTAACTTCAAAATTCGCCACAACGTTGAAGATGGTTCCGTTCAACTAGCAGACC<br>ATTATCAACAAAATACTCCAATTGGCGATGGCCCTGTCCTTTTACCAGACAACCATTACCTGTCGACACAATCTGTCCTTTCGAAAGATCCCAACGAAAA<br>GCGTGACCACATGGTCCTTCTTGAGTTTGTAACTGCTGCTGGGATTACACATGGCATGGATGAGCTCTACAAATAATTCAGCCAAAAAACTTAAGACCGC<br>CGGTCTTGTCCACTACCTTGCAGTAATGCGGTGGACAGGATCGGCGGTTTTCTTTTCTCTTCTCAACCAATGGCGGCGCGCCATCGAATGGCGCAAAACC<br>TTTCGCGGTATGGCATGATAGCGCCCGGAAGAGAGTCAATTCAGGGTGGTGAATATGAAACCAGTAACGTTATACGATGTCGCAGAGTATGCCGGTGTCT<br>CTTATCAGACCGTTTCCCGCGTGGTGAACCAGGCCAGCCACGTTTCTGCGAAAACGCGGGAAAAAGTGGAAGCGGCGATGGCGGAGCTGAATTACATTCC<br>CAACCGCGTGGCACAACAACTGGCGGGCAAACAGTCGTTGCTTATTGGCGTTGCCACCTCCAGTCTGGCCCTGCACGCGCCGTCGCAAATTGTCGCGGCG<br>ATTAAATCTCGCGCCGATCAACTGGGTGCCAGCGTGGTGGTGTCGATGGTAGAACGAAGCGGCGTCGAAGCCTGTAAAGCGGCGGTGCACAATCTTCTCG<br>CGCAACGCGTCAGTGGGCTGATCATTAACTATCCGCTGGATGACCAGGATGCCATTGCTGTGGAAGCTGCCTGCACTAATGTTCCGGCGTTATTTCTTGA<br>TGTCTCTGACCAGACACCCATCAACAGTATTATTTTCTCCCATGAGGACGGTACGCGACTGGGCGTGGAGCATCTGGTCGCATTGGGTCACCAGCAAATC<br>GCGCTGTTAGCGGGCCCATTAAGTTCTGTCTCGGCGCGTCTGCGTCTGGCTGGCTGGCATAAATATCTCACTCGCAATCAAATTCAGCCGATAGCGGAAC<br>GGGAAGGCGACTGGAGTGCCATGTCCGGTTTTCAACAAACCATGCAAATGCTGAATGAGGGCATCGTTCCCACTGCGATGCTGGTTGCCAACGATCAGAT<br>GGCGCTGGGCGCAATGCGCGCCATTACCGAGTCCGGGCTGCGCGTTGGTGCGGATATCTCGGTAGTGGGATACGACGATACCGAAGATAGCTCATGTTAT<br>ATCCCGCCGTTAACCACCATCAAACAGGATTTTCGCCTGCTGGGGCAAACCAGCGTGGACCGCTTGCTGCAACTCTCTCAGGGCCAGGCGGTGAAGGGCA<br>ATCAGCTGTTGCCAGTCTCACTGGTGAAAAGAAAAACCACCCTGGCGCCCAATACGCAAACCGCCTCTCCCCGCGCGTTGGCCGATTCATTAATGCAGCT<br>GGCACGACAGGTTTCCCGACTGGAAAGCGGGCAGTGATAATTGGTAACGAATCAGACAATTGACGGCTCGAGGGAGTAGCATAGGGTTTGCAGAATCCCT<br>GCTTCGTCCATTTGACAGGCACATTATGCATCGATGATAAGCTGTCAAACATGAGCACGCTTACTAGTAGCGGCCGCTGCAGTCCGGCAAAAAAGGGCAA<br>GGTGTCACCACCCTGCCCTTTTTCTTTAAAACCGAAAAGATTACTTCGCGTTATGCAGGCTTCCTCGCTCACTGACTCGCTGCGCTCGGTCGTTCGGCTG<br>CGGCGAGCGGTATCAGCTCACTCAAAGGCGGTAATGACAGTAAGACGGGTAAGCCTGTTGATGATACCGCTGCCTTACTGGGTGCATTAGCCAGTCTGAA<br>TGACCTGTCACGGGATAATCCGAAGTGGTCAGACTGGAAAATCAGAGGGCAGGAACTGCTGAACAGCAAAAAGTCAGATAGCACCACATAGCAGACCCGC<br>CATAAAACGCCCTGAGAAGCCCGTGACGGGCTTTTCTTGTATTATGGGTAGTTTCCTTGCATGAATCCATAAAAGGCGCCTGTAGTGCCATTTACCCCCA<br>TTCACTGCCAGAGCCGTGAGCGCAGCGAACTGAATGTCACGAAAAAGACAGCGACTCAGGTGCCTGATGGTCGGAGACAAAAGGAATATTCAGCGATTTG<br>CCCGAGCTTGCGAGGGTGCTACTTAAGCCTTTAGGGTTTTAAGGTCTGTTTTGTAGAGGAGCAAACAGCGTTTGCGACATCCTTTTGTAATACTGCGGAA<br>CTGACTAAAGTAGTGAGTTATACACAGGGCTGGGATCTATTCTTTTTATCTTTTTTTATTCTTTCTTTATTCTATAAATTATAACCACTTGAATATAAAC<br>AAAAAAAACACACAAAGGTCTAGCGGAATTTACAGAGGGTCTAGCAGAATTTACAAGTTTTCCAGCAAAGGTCTAGCAGAATTTACAGATACCCACAACT<br>CAAAGGAAAAGGACTAGTAATTATCATTGACTAGCCCATCTCAATTGGTATAGTGATTAAAATCACCTAGACCAATTGAGATGTATGTCTGAATTAGTTG<br>TTTTCAAAGCAAATGAACTAGCGATTAGTCGCTATGACTTAACGGAGCATGAAACCAAGCTAATTTTATGCTGTGTGGCACTACTCAACCCCACGATTGA<br>AAACCCTACAAGGAAAGAACGGACGGTATCGTTCACTTATAACCAATACGCTCAGATGATGAACATCAGTAGGGAAAATGCTTATGGTGTATTAGCTAAA

		GCAACCAGAGAGCTGATGACGAGAACTGTGGAAATCAGGAATCCTTTGGTTAAAGGCTTTTGGATTTTCCAGTGGACAAACTATGCCAAGTTCTCAAGCG<br>AAAAATTAGAATTAGTTTTTAGTGAAGAGATATTGCCTTATCTTTTCCAGTTAAAAAAATTCATAAAATATAATCTGGAACATGTTAAGTCTTTTGAAAA<br>CAAATACTCTATGAGGATTTATGAGTGGTTATTAAAAGAACTAACACAAAAGAAAACTCACAAGGCAAATATAGAGATTAGCCTTGATGAATTTAAGTTC<br>ATGTTAATGCTTGAAAATAACTACCATGAGTTTAAAAGGCTTAACCAATGGGTTTTGAAACCAATAAGTAAAGATTTAAACACTTACAGCAATATGAAAT<br>TGGTGGTTGATAAGCGAGGCCGCCCGACTGATACGTTGATTTTCCAAGTTGAACTAGATAGACAAATGGATCTCGTAACCGAACTTGAGAACAACCAGAT<br>AAAAATGAATGGTGACAAAATACCAACAACCATTACATCAGATTCCTACCTACATAACGGACTAAGAAAAACACTACACGATGCTTTAACTGCAAAAATT<br>CAGCTCACCAGTTTTGAGGCAAAATTTTTGAGTGACATGCAAAGTAAGTATGATCTCAATGGTTCGTTCTCATGGCTCACGCAAAAACAACGAACCACAC<br>TAGAGAACATACTGGCTAAATACGGAAGGATCTGAGGTTCTTATGGCTCTTGTATCTATCAGTGAAGCATCAAGACTAACAAACAAAAGTAGAACAACTG<br>TTCACCGTTACATATCAAAGGGAAAACTGTCCATATGCACAGATGAAAACGGTGTAAAAAAGATAGATACATCAGAGCTTTTACGAGTTTTTGGTGCATT<br>CAAAGCTGTTCACCATGAACAGATCGACAATGTAACAGATGAACAGCATGTAACACCTAATAGAACAGGTGAAACCAGTAAAACAAAGCAACTAGAACAT<br>GAAATTGAACACCTGAGACAACTTGTTACAGCTCAACAGTCACACATAGACAGCCTGAAACAGGCGATGCTGCTTATCGAATCAAAGCTGCCGACAACAC<br>GGGAGCCAGTGACGCCTCCCGTGGGGAAAAAATCATGGCAATTCTGGAAGAAATAGCGCTTTCAGCCGGCAAACCGGCTGAAGCCGGATCTGCGATTCTG<br>ATAACAAACTAGCAACACCAGAACAGCCCGTTTGCGGGCAGCAAAACCCGTACCGATTATCAAAAAGGATCTTCACCTAGATCCTTTTAAATTAAAAATG<br>AAGTTTTAAATCAATCTAAAGTATATATGAGTAAACTTGGTCTGACAGTTACCAATGCTTAATCAGTGAGGCACCTATCTCAGCGATCTGTCTATTTCGT<br>TCATCCATAGTTGCCTGACTCCCCGTCGTGTAGATAACTACGATACGGGAGGGCTTACCATCTGGCCCCAGTGCTGCAATGATACCGCGAGAACCACGCT<br>CACCGGCTCCAGATTTATCAGCAATAAACCAGCCAGCCGGAAGGGCCGAGCGCAGAAGTGGTCCTGCAACTTTATCCGCCTCCATCCAGTCTATTAATTG<br>TTGCCGGGAAGCTAGAGTAAGTAGTTCGCCAGTTAATAGTTTGCGCAACGTTGTTGCCATTGCTACAGGCATCGTGGTGTCACGCTCGTCGTTTGGTATG<br>GCTTCATTCAGCTCCGGTTCCCAACGATCAAGGCGAGTTACATGATCCCCCATGTTGTGCAAAAAAGCGGTTAGCTCCTTCGGTCCTCCGATCGTTGTCA<br>GAAGTAAGTTGGCCGCAGTGTTATCACTCATGGTTATGGCAGCACTGCATAATTCTCTTACTGTCATGCCATCCGTAAGATGCTTTTCTGTGACTGGTGA<br>GTACTCAACCAAGTCATTCTGAGAATAGTGTATGCGGCGACCGAGTTGCTCTTGCCCGGCGTCAATACGGGATAATACCGCGCCACATAGCAGAACTTTA<br>AAAGTGCTCATCATTGGAAAACGTTCTTCGGGGCGAAAACTCTCAAGGATCTTACCGCTGTTGAGATCCAGTTCGATATAACCCACTCGTGCACCCAACT<br>GATCTTCAGCATCTTTTACTTTCACCAGCGTTTCTGGGTGAGCAAAAACAGGAAGGCAAAATGCCGCAAAAAAGGGAATAAGGGCGACACGGAAATGTTG<br>AATACTCATACTCTTCCTTTTTCAATATTATTGAAGCATTTATCAGGGTTATTGTCTCATGAGCGGATACATATTTGAATGTATTTAGAAAAATAAACAA<br>ATAGGGGTTCCGCG
pEG3045	HIS6-MdnA	ATGGCATATCCCAACGATCAACAAGGTAAAGCACTTCCTTTCTTTGCTCGTTTCTTGTCCGTAAGCAAAGAGGAATCTTCCATCAAGTCTCCTTCCCCTG<br>AGCCTACCTACGGGGGCACCTTTAAATACCCTTCTGACTGGGAAGATTATTAATAA
pEG3046	HIS6-BmbC	ATGGGTCCGGTTGTTGTGTTCGATTGCATGACGGCCGACTTTCTGAACGACGATCCAAATAACGCGGAGTTGTCTGCCTTGGAAATGGAGGAGCTCGAGT<br>CCTGGGGCGCCTGGGACGGAGAGGCTACCAGCTAGTAA
pEG3047	HIS6-StrA	ATGAGTAAGGAATTAGAAAAAGTTCTTGAATCCAGTTCAATGGCAAAGGGGGACGGCTGGAAGGTTATGGCTAAAGGTGACGGTTGGGAGTAATAA
pEG3048	HIS6-PqqA	ATGTGGAAGAAACCTGCTTTTATCGATTTACGTCTCGGTCTGGAAGTGACGCTGTACATTTCTAACCGTTAATAA
pEG3049	HIS6-SboA	ATGAAAAAAGCTGTCATTGTAGAAAACAAAGGTTGTGCAACATGCTCGATCGGAGCCGCTTGTCTAGTGGACGGTCCTATCCCTGATTTTGAAATTGCCG<br>GTGCAACAGGTCTATTCGGTCTATGGGGATAA
pEG3051	HIS6-TfxA	ATGGATAACAAGGTTGCGAAGAATGTCGAAGTGAAGAAGGGCTCCATCAAGGCGACCTTCAAGGCTGCTGTTCTGAAGTCGAAGACGAAGGTCGACATCG<br>GAGGTAGCCGTCAGGGCTGCGTCGCTTAATAA
pEG3052	HIS6-ProcA1.7	ATGTCAGAAGAACAACTCAAGGCATTCATTGCCAAGGTTCAAGCAGACACTTCACTGCAGGAACAGCTCAAAGTAGAAGGTGCTGATGTTGTTGCTATTG<br>CTAAAGCCTCAGGGTTCGCGATTACCACAGAGGACTTAAAAGCACATCAAGCCAACTCACAAAAGAACCTGTCTGATGCTGAGCTGGAAGGTGTGGCTGG<br>GCGAACCATTGGGGGAACCATTGTGTCGATAACCTGTGAGACTTGCGATCTGCTTGTGGGGAAAATGTGCTGATAA
pEG3053	HIS6-TbtA	ATGGACCTGAATGATCTGCCGATGGATGTTTTTGAACTGGCAGATAGCGGTGTTGCAGTTGAAAGCCTGACCGCAGGTCATGGTATGACCGAAGTTGGTG<br>CAAGCTGTAATTGCTTTTGTTATATTTGTTGTAGCTGCAGCAGCGCCTAATAA
pEG3055	HIS6-Pgm2	ATGGAGCGCGAAATCGTGTGGACAGAAATTGAGGAGTCGGATTTAGCCGCCGTCGTGTCGGCATCTAATGTCAAGGATGGTCCAACCGTTAGCTCAAGTA<br>ATGTAAAGGACCGCTAATAA

Initial RST$_N$ (with Link-1) expression vector

Backbone Sequence	CACATTTCCCCGAAAAGTGCCACCTGACGTCTAAGAAACCATTATTATCATGACATTAACCTATAAAAATAGGCGTATCACGAGGCAGAATTTCAGATAA<br>AAAAAATCCTTAGCTTTCGCTAAGGATGATTTCTGGAATTCGCGGCCGCTTCTAGAGGGAGAACGATCGTTGGCTGAATCATAAAAAATTTATTTGCTTT<br>GTGAGCGGATAACAATTATAATAGATTCAATTGTGAGCGGATAACAATTAGAACCCAACACCACCAGCAAGCCTAAGGAGGAGAAATATGTCATATTACC<br>ACCATCACCATCATCACGGGTCCCTGCAGGACTCAGAAGTCAATCAAGAAGCTAAGCCAGAGGTCAAGCCAGAAGTCAAGCCTGAGACTCACATCAATTT<br>AAAGGTGTCCGATGGATCTTCAGAGATCTTCTTCAAGATCAAAAAGACCACTCCTTTAAGAAGGCTGATGGAAGCGTTCGCTAAAAGACAGGGTAAGGAA<br>ATGGACTCCTTAAGATTCTTGTACGACGGTATTAGAATTCAAGCTGATCAGGCCCCTGAAGATTTGGACATGGAGGATAACGATATTATTGAGGCTCACC<br>GCGAACAGATTGGAGGTCATCACCATCACCACCATGGATATGATATTAGCACAGGTATGAGCAAAGGAGAAGAACTTTTCACTGGAGTTGTCCCAATTCT<br>TGTTGAATTAGATGGTGATGTTAATGGGCACAAATTTTCTGTCCGTGGAGAGGGTGAAGGTGATGCTACAAACGGAAAACTCACCCTTAAATTTATTTGC<br>ACTACTGGAAAACTACCTGTTCCGTGGCCAACACTTGTCACTACTCTGACCTATGGTGTTCAATGCTTTTCCCGTTATCCGGATCACATGAAACGGCATG<br>ACTTTTTCAAGAGTGCCATGCCCGAAGGTTATGTACAGGAACGCACTATATCTTTCAAAGATGACGGGACCTACAAGACGCGTGCTGAAGTCAAGTTTGA<br>AGGTGATACCCTTGTTAATCGTATCGAGTTAAAGGGTATTGATTTTAAAGAAGATGGAAACATTCTTGGACACAAACTCGAGTACAACTTTAACTCACAC<br>AATGTATACATCACGGCAGACAAACAAAAGAATGGAATCAAAGCTAACTTCAAAATTCGCCACAACGTTGAAGATGGTTCCGTTCAACTAGCAGACCATT<br>ATCAACAAAATACTCCAATTGGCGATGGCCCTGTCCTTTTACCAGACAACCATTACCTGTCGACACAATCTGTCCTTTCGAAAGATCCCAACGAAAAGCG<br>TGACCACATGGTCCTTCTTGAGTTTGTAACTGCTGCTGGGATTACACATGGCATGGATGAGCTCTACAAATAATTCAGCCAAAAAACTTAAGACCGCCGG<br>TCTTGTCCACTACCTTGCAGTAATGCGGTGGACAGGATCGGCGGTTTTCTTTTCTCTTCTCAACCAATGGCGGCGCGCCATCGAATGGCGCAAAACCTTT<br>CGCGGTATGGCATGATAGCGCCCGGAAGAGAGTCAATTCAGGGTGGTGAATATGAAACCAGTAACGTTATACGATGTCGCAGAGTATGCCGGTGTCTCTT<br>ATCAGACCGTTTCCCGCGTGGTGAACCAGGCCAGCCACGTTTCTGCGAAAACGCGGGAAAAAGTGGAAGCGGCGATGGCGGAGCTGAATTACATTCCCAA<br>CCGCGTGGCACAACAACTGGCGGGCAAACAGTCGTTGCTTATTGGCGTTGCCACCTCCAGTCTGGCCCTGCACGCGCCGTCGCAAATTGTCGCGGCGATT<br>AAATCTCGCGCCGATCAACTGGGTGCCAGCGTGGTGGTGTCGATGGTAGAACGAAGCGGCGTCGAAGCCTGTAAAGCGGCGGTGCACAATCTTCTCGCGC<br>AACGCGTCAGTGGGCTGATCATTAACTATCCGCTGGATGACCAGGATGCCATTGCTGTGGAAGCTGCCTGCACTAATGTTCCGGCGTTATTTCTTGATGT<br>CTCTGACCAGACACCCATCAACAGTATTATTTTCTCCCATGAGGACGGTACGCGACTGGGCGTGGAGCATCTGGTCGCATTGGGTCACCAGCAAATCGCG<br>CTGTTAGCGGGCCCATTAAGTTCTGTCTCGGCGCGTCTGCGTCTGGCTGGCTGGCATAAATATCTCACTCGCAATCAAATTCAGCCGATAGCGGAACGGG<br>AAGGCGACTGGAGTGCCATGTCCGGTTTTCAACAAACCATGCAAATGCTGAATGAGGGCATCGTTCCCACTGCGATGCTGGTTGCCAACGATCAGATGGC<br>GCTGGGCGCAATGCGCGCCATTACCGAGTCCGGGCTGCGCGTTGGTGCGGATATCTCGGTAGTGGGATACGACGATACCGAAGATAGCTCATGTTATATC<br>CCGCCGTTAACCACCATCAAACAGGATTTTCGCCTGCTGGGGCAAACCAGCGTGGACCGCTTGCTGCAACTCTCTCAGGGCCAGGCGGTGAAGGGCAATC<br>AGCTGTTGCCAGTCTCACTGGTGAAAAGAAAAACCACCCTGGCGCCCAATACGCAAACCGCCTCTCCCCGCGCGTTGGCCGATTCATTAATGCAGCTGGC<br>ACGACAGGTTTCCCGACTGGAAAGCGGGCAGTGATAATTGGTAACGAATCAGACAATTGACGGCTCGAGGGAGTAGCATAGGGTTTGCAGAATCCCTGCT<br>TCGTCCATTTGACAGGCACATTATGCATCGATGATAAGCTGTCAAACATGAGCACGCTTACTAGTAGCGGCCGCTGCAGTCCGGCAAAAAAGGGCAAGGT<br>GTCACCACCCTGCCCTTTTTCTTTAAAACCGAAAAGATTACTTCGCGTTATGCAGGCTTCCTCGCTCACTGACTCGCTGCGCTCGGTCGTTCGGCTGCGG<br>CGAGCGGTATCAGCTCACTCAAAGGCGGTAATGACAGTAAGACGGGTAAGCCTGTTGATGATACCGCTGCCTTACTGGGTGCATTAGCCAGTCTGAATGA<br>CCTGTCACGGGATAATCCGAAGTGGTCAGACTGGAAAATCAGAGGGCAGGAACTGCTGAACAGCAAAAAGTCAGATAGCACCACATAGCAGACCCGCCAT<br>AAAACGCCCTGAGAAGCCCGTGACGGGCTTTTCTTGTATTATGGGTAGTTTCCTTGCATGAATCCATAAAAGGCGCCTGTAGTGCCATTTACCCCCATTC<br>ACTGCCAGAGCCGTGAGCGCAGCGAACTGAATGTCACGAAAAAGACAGCGACTCAGGTGCCTGATGGTCGGAGACAAAAGGAATATTCAGCGATTTGCCC<br>GAGCTTGCGAGGGTGCTACTTAAGCCTTTAGGGTTTTAAGGTCTGTTTTGTAGAGGAGCAAACAGCGTTTGCGACATCCTTTTGTAATACTGCGGAACTG<br>ACTAAAGTAGTGAGTTATACACAGGGCTGGGATCTATTCTTTTTATCTTTTTTTATTCTTTCTTTATTCTATAAATTATAACCACTTGAATATAAACAAA<br>AAAAACACACAAAGGTCTAGCGGAATTTACAGAGGGTCTAGCAGAATTTACAAGTTTTCCAGCAAAGGTCTAGCAGAATTTACAGATACCCACAACTCAA<br>AGGAAAAGGACTAGTAATTATCATTGACTAGCCCATCTCAATTGGTATAGTGATTAAAATCACCTAGACCAATTGAGATGTATGTCTGAATTAGTTGTTT<br>TCAAAGCAAATGAACTAGCGATTAGTCGCTATGACTTAACGGAGCATGAAACCAAGCTAATTTTATGCTGTGTGGCACTACTCAACCCCACGATTGAAAA<br>CCCTACAAGGAAAGAACGGACGGTATCGTTCACTTATAACCAATACGCTCAGATGATGAACATCAGTAGGGAAAATGCTTATGGTGTATTAGCTAAAGCA<br>ACCAGAGAGCTGATGACGAGAACTGTGGAAATCAGGAATCCTTTGGTTAAAGGCTTTTGGATTTTCCAGTGGACAAACTATGCCAAGTTCTCAAGCGAAA<br>AATTAGAATTAGTTTTTAGTGAAGAGATATTGCCTTATCTTTTCCAGTTAAAAAAATTCATAAAATATAATCTGGAACATGTTAAGTCTTTTGAAAACAA<br>ATACTCTATGAGGATTTATGAGTGGTTATTAAAAGAACTAACACAAAAGAAAACTCACAAGGCAAATATAGAGATTAGCCTTGATGAATTTAAGTTCATG<br>TTAATGCTTGAAAATAACTACCATGAGTTTAAAAGGCTTAACCAATGGGTTTTGAAACCAATAAGTAAAGATTTAAACACTTACAGCAATATGAAATTGG<br>TGGTTGATAAGCGAGGCCGCCCGACTGATACGTTGATTTTCCAAGTTGAACTAGATAGACAAATGGATCTCGTAACCGAACTTGAGAACAACCAGATAAA

		AATGAATGGTGACAAAATACCAACAACCATTACATCAGATTCCTACCTACATAACGGACTAAGAAAAACACTACACGATGCTTTAACTGCAAAAATTCAG<br>CTCACCAGTTTTGAGGCAAAATTTTTGAGTGACATGCAAAGTAAGTATGATCTCAATGGTTCGTTCTCATGGCTCACGCAAAAACAACGAACCACACTAG<br>AGAACATACTGGCTAAATACGGAAGGATCTGAGGTTCTTATGGCTCTTGTATCTATCAGTGAAGCATCAAGACTAACAAACAAAAGTAGAACAACTGTTC<br>ACCGTTACATATCAAAGGGAAAACTGTCCATATGCACAGATGAAAACGGTGTAAAAAAGATAGATACATCAGAGCTTTTACGAGTTTTTGGTGCATTCAA<br>AGCTGTTCACCATGAACAGATCGACAATGTAACAGATGAACAGCATGTAACACCTAATAGAACAGGTGAAACCAGTAAAACAAAGCAACTAGAACATGAA<br>ATTGAACACCTGAGACAACTTGTTACAGCTCAACAGTCACACATAGACAGCCTGAAACAGGCGATGCTGCTTATCGAATCAAAGCTGCCGACAACACGGG<br>AGCCAGTGACGCCTCCCGTGGGGAAAAAATCATGGCAATTCTGGAAGAAATAGCGCTTTCAGCCGGCAAACCGGCTGAAGCCGGATCTGCGATTCTGATA<br>ACAAACTAGCAACACCAGAACAGCCCGTTTGCGGGCAGCAAAACCCGTACCGATTATCAAAAAGGATCTTCACCTAGATCCTTTTAAATTAAAAATGAAG<br>TTTTAAATCAATCTAAAGTATATATGAGTAAACTTGGTCTGACAGTTACCAATGCTTAATCAGTGAGGCACCTATCTCAGCGATCTGTCTATTTCGTTCA<br>TCCATAGTTGCCTGACTCCCCGTCGTGTAGATAACTACGATACGGGAGGGCTTACCATCTGGCCCCAGTGCTGCAATGATACCGCGAGAACCACGCTCAC<br>CGGCTCCAGATTTATCAGCAATAAACCAGCCAGCCGGAAGGGCCGAGCGCAGAAGTGGTCCTGCAACTTTATCCGCCTCCATCCAGTCTATTAATTGTTG<br>CCGGGAAGCTAGAGTAAGTAGTTCGCCAGTTAATAGTTTGCGCAACGTTGTTGCCATTGCTACAGGCATCGTGGTGTCACGCTCGTCGTTTGGTATGGCT<br>TCATTCAGCTCCGGTTCCCAACGATCAAGGCGAGTTACATGATCCCCCATGTTGTGCAAAAAAGCGGTTAGCTCCTTCGGTCCTCCGATCGTTGTCAGAA<br>GTAAGTTGGCCGCAGTGTTATCACTCATGGTTATGGCAGCACTGCATAATTCTCTTACTGTCATGCCATCCGTAAGATGCTTTTCTGTGACTGGTGAGTA<br>CTCAACCAAGTCATTCTGAGAATAGTGTATGCGGCGACCGAGTTGCTCTTGCCCGGCGTCAATACGGGATAATACCGCGCCACATAGCAGAACTTTAAAA<br>GTGCTCATCATTGGAAAACGTTCTTCGGGGCGAAAACTCTCAAGGATCTTACCGCTGTTGAGATCCAGTTCGATATAACCCACTCGTGCACCCAACTGAT<br>CTTCAGCATCTTTTACTTTCACCAGCGTTTCTGGGTGAGCAAAAACAGGAAGGCAAAATGCCGCAAAAAAGGGAATAAGGGCGACACGGAAATGTTGAAT<br>ACTCATACTCTTCCTTTTTCAATATTATTGAAGCATTTATCAGGGTTATTGTCTCATGAGCGGATACATATTTGAATGTATTTAGAAAAATAAACAAATA<br>GGGGTTCCGCG
pEG3057	RST$_N$ (Link-1)-TruE*	ATGAACAAGAAGAACATTTTACCGCAGTTAGGACAACCAGTCATCCGCCTTACTGCCGGTCAACTGTCAAGCCAACTGGCGGAGCTTTCTGAGGAGGCTC<br>TGGGAGGGGTCGATGCCTCGTACGCGGTGTTCTGGCCGATCTGTAGCTATGACGACTAATAA
pEG3058	RST$_N$ (Link-1)-MdnA	ATGGCATATCCCAACGATCAACAAGGTAAAGCACTTCCTTTCTTTGCTCGTTTCTTGTCCGTAAGCAAAGAGGAATCTTCCATCAAGTCTCCTTCCCCTG<br>AGCCTACCTACGGGGGCACCTTTAAATACCCTTCTGACTGGGAAGATTATTAATAA
pEG3059	RST$_N$ (Link-1)-SboA	ATGAAAAAAGCTGTCATTGTAGAAAACAAAGGTTGTGCAACATGCTCGATCGGAGCCGCTTGTCTAGTGGACGGTCCTATCCCTGATTTTGAAATTGCCG<br>GTGCAACAGGTCTATTCGGTCTATGGGGATAA
pEG3060	RST$_N$ (Link-1)-PqqA	ATGTGGAAGAAACCTGCTTTTATCGATTTACGTCTCGGTCTGGAAGTGACGCTGTACATTTCTAACCGTTAATAA
pEG3061	RST$_N$ (with Link-1)-StrA	ATGAGTAAGGAATTAGAAAAAGTTCTTGAATCCAGTTCAATGGCAAAGGGGGACGGCTGGAAGGTTATGGCTAAAGGTGACGGTTGGGAGTAATAA
pEG3062	RST$_N$ (Link-1)-BmbC	ATGGGTCCGGTTGTTGTGTTCGATTGCATGACGGCCGACTTTCTGAACGACGATCCAAATAACGCGGAGTTGTCTGCCTTGGAAATGGAGGAGCTCGAGT<br>CCTGGGGCGCCTGGGACGGAGAGGCTACCAGCTAGTAA
pEG3063	RST$_N$ (Link-1)-TfxA	ATGGATAACAAGGTTGCGAAGAATGTCGAAGTGAAGAAGGGCTCCATCAAGGCGACCTTCAAGGCTGCTGTTCTGAAGTCGAAGACGAAGGTCGACATCG<br>GAGGTAGCCGTCAGGGCTGCGTCGCTTAATAA
pEG3064	RST$_N$ (Link-1)-ProcA1.7	ATGTCAGAAGAACAACTCAAGGCATTCATTGCCAAGGTTCAAGCAGACACTTCACTGCAGGAACAGCTCAAAGTAGAAGGTGCTGATGTTGTTGCTATTG<br>CTAAAGCCTCAGGGTTCGCGATTACCACAGAGGACTTAAAAGCACATCAAGCCAACTCACAAAAGAACCTGTCTGATGCTGAGCTGGAAGGTGTGGCTGG<br>GCGAACCATTGGGGGAACCATTGTGTCGATAACCTGTGAGACTTGCGATCTGCTTGTGGGGAAAATGTGCTGATAA
pEG3065	RST$_N$ (Link-1)-TbtA	ATGGACCTGAATGATCTGCCGATGGATGTTTTTGAACTGGCAGATAGCGGTGTTGCAGTTGAAAGCCTGACCGCAGGTCATGGTATGACCGAAGTTGGTG<br>CAAGCTGTAATTGCTTTTGTTATATTTGTTGTAGCTGCAGCAGCGCCTAATAA
pEG3067	RST$_N$ (Link-1)-Pgm2	ATGGAGCGCGAAATCGTGTGGACAGAAATTGAGGAGTCGGATTTAGCCGCCGTCGTGTCGGCATCTAATGTCAAGGATGGTCCAACCGTTAGCTCAAGTA<br>ATGTAAAGGACCGCTAATAA

RST$_N$ expression vector

Backbone Sequence	CACATTTCCCCGAAAAGTGCCACCTGACGTCTAAGAAACCATTATTATCATGACATTAACCTATAAAAATAGGCGTATCACGAGGCAGAATTTCAGATAA<br>AAAAAATCCTTAGCTTTCGCTAAGGATGATTTCTGGAATTCGCGGCCGCTTCTAGAGGGAGAACGATCGTTGGCTGAATCATAAAAAATTTATTTGCTTT<br>GTGAGCGGATAACAATTATAATAGATTCAATTGTGAGCGGATAACAATTAGAACCCAACACCACCAGCAAGCCTAAGGAGGAGAAATATGTCATATTACC<br>ACCATCACCATCATCACGGGTCCCTGCAGGACTCAGAAGTCAATCAAGAAGCTAAGCCAGAGGTCAAGCCAGAAGTCAAGCCTGAGACTCACATCAATTT<br>AAAGGTGTCCGATGGATCTTCAGAGATCTTCTTCAAGATCAAAAAGACCACTCCTTTAAGAAGGCTGATGGAAGCGTTCGCTAAAAGACAGGGTAAGGAA<br>ATGGACTCCTTAAGATTCTTGTACGACGGTATTAGAATTCAAGCTGATCAGGCCCCTGAAGATTTGGACATGGAGGATAACGATATTATTGAGGCTCACC<br>GCGAACAGATTGGAGGTTGCATGTCATATTACGACTCCATTCCCACAAGCGAGAACTTGTACTTTCAAGGGTGCATGAGCAAAGGAGAAGAACTTTTCAC<br>TGGAGTTGTCCCAATTCTTGTTGAATTAGATGGTGATGTTAATGGGCACAAATTTTCTGTCCGTGGAGAGGGTGAAGGTGATGCTACAAACGGAAAACTC<br>ACCCTTAAATTTATTTGCACTACTGGAAAACTACCTGTTCCGTGGCCAACACTTGTCACTACTCTGACCTATGGTGTTCAATGCTTTTCCCGTTATCCGG<br>ATCACATGAAACGGCATGACTTTTTCAAGAGTGCCATGCCCGAAGGTTATGTACAGGAACGCACTATATCTTTCAAAGATGACGGGACCTACAAGACGCG<br>TGCTGAAGTCAAGTTTGAAGGTGATACCCTTGTTAATCGTATCGAGTTAAAGGGTATTGATTTTAAAGAAGATGGAAACATTCTTGGACACAAACTCGAG<br>TACAACTTTAACTCACACAATGTATACATCACGGCAGACAAACAAAAGAATGGAATCAAAGCTAACTTCAAAATTCGCCACAACGTTGAAGATGGTTCCG<br>TTCAACTAGCAGACCATTATCAACAAAATACTCCAATTGGCGATGGCCCTGTCCTTTTACCAGACAACCATTACCTGTCGACACAATCTGTCCTTTCGAA<br>AGATCCCAACGAAAAGCGTGACCACATGGTCCTTCTTGAGTTTGTAACTGCTGCTGGGATTACACATGGCATGGATGAGCTCTACAAATAATTCAGCCAA<br>AAAACTTAAGACCGCCGGTCTTGTCCACTACCTTGCAGTAATGCGGTGGACAGGATCGGCGGTTTTCTTTTCTCTTCTCAACCAATGGCGGCGCGCCATC<br>GAATGGCGCAAAACCTTTCGCGGTATGGCATGATAGCGCCCGGAAGAGAGTCAATTCAGGGTGGTGAATATGAAACCAGTAACGTTATACGATGTCGCAG<br>AGTATGCCGGTGTCTCTTATCAGACCGTTTCCCGCGTGGTGAACCAGGCCAGCCACGTTTCTGCGAAAACGCGGGAAAAAGTGGAAGCGGCGATGGCGGA<br>GCTGAATTACATTCCCAACCGCGTGGCACAACAACTGGCGGGCAAACAGTCGTTGCTTATTGGCGTTGCCACCTCCAGTCTGGCCCTGCACGCGCCGTCG<br>CAAATTGTCGCGGCGATTAAATCTCGCGCCGATCAACTGGGTGCCAGCGTGGTGGTGTCGATGGTAGAACGAAGCGGCGTCGAAGCCTGTAAAGCGGCGG<br>TGCACAATCTTCTCGCGCAACGCGTCAGTGGGCTGATCATTAACTATCCGCTGGATGACCAGGATGCCATTGCTGTGGAAGCTGCCTGCACTAATGTTCC<br>GGCGTTATTTCTTGATGTCTCTGACCAGACACCCATCAACAGTATTATTTTCTCCCATGAGGACGGTACGCGACTGGGCGTGGAGCATCTGGTCGCATTG<br>GGTCACCAGCAAATCGCGCTGTTAGCGGGCCCATTAAGTTCTGTCTCGGCGCGTCTGCGTCTGGCTGGCTGGCATAAATATCTCACTCGCAATCAAATTC<br>AGCCGATAGCGGAACGGGAAGGCGACTGGAGTGCCATGTCCGGTTTTCAACAAACCATGCAAATGCTGAATGAGGGCATCGTTCCCACTGCGATGCTGGT<br>TGCCAACGATCAGATGGCGCTGGGCGCAATGCGCGCCATTACCGAGTCCGGGCTGCGCGTTGGTGCGGATATCTCGGTAGTGGGATACGACGATACCGAA<br>GATAGCTCATGTTATATCCCGCCGTTAACCACCATCAAACAGGATTTTCGCCTGCTGGGGCAAACCAGCGTGGACCGCTTGCTGCAACTCTCTCAGGGCC<br>AGGCGGTGAAGGGCAATCAGCTGTTGCCAGTCTCACTGGTGAAAAGAAAAACCACCCTGGCGCCCAATACGCAAACCGCCTCTCCCCGCGCGTTGGCCGA<br>TTCATTAATGCAGCTGGCACGACAGGTTTCCCGACTGGAAAGCGGGCAGTGATAATTGGTAACGAATCAGACAATTGACGGCTCGAGGGAGTAGCATAGG<br>GTTTGCAGAATCCCTGCTTCGTCCATTTGACAGGCACATTATGCATCGATGATAAGCTGTCAAACATGAGCACGCTTACTAGTAGCGGCCGCTGCAGTCC<br>GGCAAAAAAGGGCAAGGTGTCACCACCCTGCCCTTTTTCTTTAAAACCGAAAAGATTACTTCGCGTTATGCAGGCTTCCTCGCTCACTGACTCGCTGCGC<br>TCGGTCGTTCGGCTGCGGCGAGCGGTATCAGCTCACTCAAAGGCGGTAATGACAGTAAGACGGGTAAGCCTGTTGATGATACCGCTGCCTTACTGGGTGC<br>ATTAGCCAGTCTGAATGACCTGTCACGGGATAATCCGAAGTGGTCAGACTGGAAAATCAGAGGGCAGGAACTGCTGAACAGCAAAAAGTCAGATAGCACC<br>ACATAGCAGACCCGCCATAAAACGCCCTGAGAAGCCCGTGACGGGCTTTTCTTGTATTATGGGTAGTTTCCTTGCATGAATCCATAAAAGGCGCCTGTAG<br>TGCCATTTACCCCCATTCACTGCCAGAGCCGTGAGCGCAGCGAACTGAATGTCACGAAAAAGACAGCGACTCAGGTGCCTGATGGTCGGAGACAAAAGGA<br>ATATTCAGCGATTTGCCCGAGCTTGCGAGGGTGCTACTTAAGCCTTTAGGGTTTTAAGGTCTGTTTTGTAGAGGAGCAAACAGCGTTTGCGACATCCTTT<br>TGTAATACTGCGGAACTGACTAAAGTAGTGAGTTATACACAGGGCTGGGATCTATTCTTTTTATCTTTTTTTATTCTTTCTTTATTCTATAAATTATAAC<br>CACTTGAATATAAACAAAAAAAACACACAAAGGTCTAGCGGAATTTACAGAGGGTCTAGCAGAATTTACAAGTTTTCCAGCAAAGGTCTAGCAGAATTTA<br>CAGATACCCACAACTCAAAGGAAAAGGACTAGTAATTATCATTGACTAGCCCATCTCAATTGGTATAGTGATTAAAATCACCTAGACCAATTGAGATGTA<br>TGTCTGAATTAGTTGTTTTCAAAGCAAATGAACTAGCGATTAGTCGCTATGACTTAACGGAGCATGAAACCAAGCTAATTTTATGCTGTGTGGCACTACT

CAACCCCACGATTGAAAACCCTACAAGGAAAGAACGGACGGTATCGTTCACTTATAACCAATACGCTCAGATGATGAACATCAGTAGGGAAAATGCTTAT
GGTGTATTAGCTAAAGCAACCAGAGAGCTGATGACGAGAACTGTGGAAATCAGGAATCCTTTGGTTAAAGGCTTTTGGATTTTCCAGTGGACAAACTATG
CCAAGTTCTCAAGCGAAAAATTAGAATTAGTTTTTAGTGAAGAGATATTGCCTTATCTTTTCCAGTTAAAAAAATTCATAAAATATAATCTGGAACATGT
TAAGTCTTTTGAAAACAAATACTCTATGAGGATTTATGAGTGGTTATTAAAAGAACTAACACAAAAGAAAACTCACAAGGCAAATATAGAGATTAGCCTT
GATGAATTTAAGTTCATGTTAATGCTTGAAAATAACTACCATGAGTTTAAAAGGCTTAACCAATGGGTTTTGAAACCAATAAGTAAAGATTTAAACACTT
ACAGCAATATGAAATTGGTGGTTGATAAGCGAGGCCGCCCGACTGATACGTTGATTTTCCAAGTTGAACTAGATAGACAAATGGATCTCGTAACCGAACT
TGAGAACAACCAGATAAAAATGAATGGTGACAAAATACCAACAACCATTACATCAGATTCCTACCTACATAACGGACTAAGAAAAACACTACACGATGCT
TTAACTGCAAAAATTCAGCTCACCAGTTTTGAGGCAAAATTTTTGAGTGACATGCAAAGTAAGTATGATCTCAATGGTTCGTTCTCATGGCTCACGCAAA
AACAACGAACCACACTAGAGAACATACTGGCTAAATACGGAAGGATCTGAGGTTCTTATGGCTCTTGTATCTATCAGTGAAGCATCAAGACTAACAAACA
AAAGTAGAACAACTGTTCACCGTTACATATCAAAGGGAAAACTGTCCATATGCACAGATGAAAACGGTGTAAAAAAGATAGATACATCAGAGCTTTTACG
AGTTTTTGGTGCATTCAAAGCTGTTCACCATGAACAGATCGACAATGTAACAGATGAACAGCATGTAACACCTAATAGAACAGGTGAAACCAGTAAAACA
AAGCAACTAGAACATGAAATTGAACACCTGAGACAACTTGTTACAGCTCAACAGTCACACATAGACAGCCTGAAACAGGCGATGCTGCTTATCGAATCAA
AGCTGCCGACAACACGGGAGCCAGTGACGCCTCCCGTGGGGAAAAAATCATGGCAATTCTGGAAGAAATAGCGCTTTCAGCCGGCAAACCGGCTGAAGCC
GGATCTGCGATTCTGATAACAAACTAGCAACACCAGAACAGCCCGTTTGCGGGCAGCAAAACCCGTACCGATTATCAAAAAGGATCTTCACCTAGATCCT
TTTAAATTAAAAATGAAGTTTTAAATCAATCTAAAGTATATATGAGTAAACTTGGTCTGACAGTTACCAATGCTTAATCAGTGAGGCACCTATCTCAGCG
ATCTGTCTATTTCGTTCATCCATAGTTGCCTGACTCCCCGTCGTGTAGATAACTACGATACGGGAGGGCTTACCATCTGGCCCCAGTGCTGCAATGATAC
CGCGAGAACCACGCTCACCGGCTCCAGATTTATCAGCAATAAACCAGCCAGCCGGAAGGGCCGAGCGCAGAAGTGGTCCTGCAACTTTATCCGCCTCCAT
CCAGTCTATTAATTGTTGCCGGGAAGCTAGAGTAAGTAGTTCGCCAGTTAATAGTTTGCGCAACGTTGTTGCCATTGCTACAGGCATCGTGGTGTCACGC
TCGTCGTTTGGTATGGCTTCATTCAGCTCCGGTTCCCAACGATCAAGGCGAGTTACATGATCCCCCATGTTGTGCAAAAAAGCGGTTAGCTCCTTCGGTC
CTCCGATCGTTGTCAGAAGTAAGTTGGCCGCAGTGTTATCACTCATGGTTATGGCAGCACTGCATAATTCTCTTACTGTCATGCCATCCGTAAGATGCTT
TTCTGTGACTGGTGAGTACTCAACCAAGTCATTCTGAGAATAGTGTATGCGGCGACCGAGTTGCTCTTGCCCGGCGTCAATACGGGATAATACCGCGCCA
CATAGCAGAACTTTAAAAGTGCTCATCATTGGAAAACGTTCTTCGGGGCGAAAACTCTCAAGGATCTTACCGCTGTTGAGATCCAGTTCGATATAACCCA
CTCGTGCACCCAACTGATCTTCAGCATCTTTTACTTTCACCAGCGTTTCTGGGTGAGCAAAAACAGGAAGGCAAAATGCCGCAAAAAAGGGAATAAGGGC
GACACGGAAATGTTGAATACTCATACTCTTCCTTTTTCAATATTATTGAAGCATTTATCAGGGTTATTGTCTCATGAGCGGATACATATTTGAATGTATT
TAGAAAAATAAACAAATAGGGGTTCCGCG

pEG3121 MdnA*

ATGGCACTTCCTTTCTTTGCTCGTTTCTTGTCCGTAAGCAAAGAGGAATCTTCCATCAAGTCTCCTTCCCCTGAGCCTACCTACGGGGGCACCTTTAAAT
ACCCTTCTGACTGGGAAGATTATTAATAA

pEG3128 ProcA*

ATGTCAGAAGAACAACTCAAGGCATTCATTGCCAAGGTTCAAGCAGACACTTCACTGCAGGAACAGCTCAAAGTAGAAGGTGCTGATGTTGTTGCTATTG
CTAAAGCCTCAGGGTTCGCGATTACCACAGAGGACCTCAATTCGCATCGCCAAAATCTGTCTGATGATGAGCTGGAGGGAGTCGCGGGAGGCTTTTTCTG
CGTACAGGGTACGGCCAACCGTTTCACTATCAACGTTTGCTGATAA

pEG3132 PaaP

ATGATTAAATTTTCTACATTGTCTCAGCGCATCAGCGCCATCACGGAAGAAAACGCCATGTACACTAAGGGTCAAGTGATCGTATTGAGCTGATAA

pEG3248 SboA

ATGAAAAAAGCTGTCATTGTAGAAAACAAAGGTTGTGCAACATGCTCGATCGGAGCCGCTTGTCTAGTGGACGGTCCTATCCCTGATTTTGAAATTGCCG
GTGCAACAGGTCTATTCGGTCTATGGGGATAA

RST$_N$ expression vector (w/ flanking restriction sites)

Backbone Sequence

CACATTTCCCCGAAAAGTGCCACCTGACGTCTAAGAAACCATTATTATCATGACATTAACCTATAAAAATAGGCGTATCACGAGGCAGAATTTCAGATAA
AAAAAATCCTTAGCTTTCGCTAAGGATGATTTCTGGAATTCGCGGCCGCTTCTAGAGGGAGGGTCTCAGTGCAACGATCGTTGGCTGAATCATAAAAAAT
TTATTTGCTTTGTGAGCGGATAACAATTATAATAGATTCAATTGTGAGCGGATAACAATTAGAACCCAACACCACCAGCAAGCCTAAGGAGGAGAAATAT
GTCATATTACCACCATCACCATCATCACGGGTCCCTGCAGGACTCAGAAGTCAATCAAGAAGCTAAGCCAGAGGTCAAGCCAGAAGTCAAGCCTGAGACT
CACATCAATTTAAAGGTGTCCGATGGATCTTCAGAGATCTTCTTCAAGATCAAAAAGACCACTCCTTTAAGAAGGCTGATGGAAGCGTTCGCTAAAAGAC
AGGGTAAGGAAATGGACTCCTTAAGATTCTTGTACGACGGTATTAGAATTCAAGCTGATCAGGCCCCTGAAGATTTGGACATGGAGGATAACGATATTAT
TGAGGCTCACCGCGAACAGATTGGAGGTTGCATGTCATATTACGACTCCATTCCCACAAGCGAGAACTTGTACTTTCAAGGGTGCATGAGCAAAGGAGAA
GAACTTTTCACTGGAGTTGTCCCAATTCTTGTTGAATTAGATGGTGATGTTAATGGGCACAAATTTTCTGTCCGTGGAGAGGGTGAAGGTGATGCTACAA
ACGGAAAACTCACCCTTAAATTTATTTGCACTACTGGAAAACTACCTGTTCCGTGGCCAACACTTGTCACTACTCTGACCTATGGTGTTCAATGCTTTTC
CCGTTATCCGGATCACATGAAACGGCATGACTTTTTCAAGAGTGCCATGCCCGAAGGTTATGTACAGGAACGCACTATATCTTTCAAAGATGACGGGACC
TACAAGACGCGTGCTGAAGTCAAGTTTGAAGGTGATACCCTTGTTAATCGTATCGAGTTAAAGGGTATTGATTTTAAAGAAGATGGAAACATTCTTGGAC
ACAAACTCGAGTACAACTTTAACTCACACAATGTATACATCACGGCAGACAAACAAAAGAATGGAATCAAAGCTAACTTCAAAATTCGCCACAACGTTGA
AGATGGTTCCGTTCAACTAGCAGACCATTATCAACAAAATACTCCAATTGGCGATGGCCCTGTCCTTTTACCAGACAACCATTACCTGTCGACACAATCT
GTCCTTTCGAAAGATCCCAACGAAAAGCGTGACCACATGGTCCTTCTTGAGTTTGTAACTGCTGCTGGGATTACACATGGCATGGATGAGCTCTACAAAT
AATTCAGCCAAAAAACTTAAGACCGCCGGTCTTGTCCACTACCTTGCAGTAATGCGGTGGACAGGATCGGCGGTTTTCTTTTCTCTTCTCAACAAGTGAG
ACCATGGGCGGCGCGCCATCGAATGGCGCAAAACCTTTCGCGGTATGGCATGATAGCGCCCGGAAGAGAGTCAATTCAGGGTGGTGAATATGAAACCAGT
AACGTTATACGATGTCGCAGAGTATGCCGGTGTCTCTTATCAGACCGTTTCCCGCGTGGTGAACCAGGCCAGCCACGTTTCTGCGAAAACGCGGGAAAAA
GTGGAAGCGGCGATGGCGGAGCTGAATTACATTCCCAACCGCGTGGCACAACAACTGGCGGGCAAACAGTCGTTGCTTATTGGCGTTGCCACCTCCAGTC
TGGCCCTGCACGCGCCGTCGCAAATTGTCGCGGCGATTAAATCTCGCGCCGATCAACTGGGTGCCAGCGTGGTGGTGTCGATGGTAGAACGAAGCGGCGT
CGAAGCCTGTAAAGCGGCGGTGCACAATCTTCTCGCGCAACGCGTCAGTGGGCTGATCATTAACTATCCGCTGGATGACCAGGATGCCATTGCTGTGGAA
GCTGCCTGCACTAATGTTCCGGCGTTATTTCTTGATGTCTCTGACCAGACACCCATCAACAGTATTATTTTCTCCCATGAGGACGGTACGCGACTGGGCG
TGGAGCATCTGGTCGCATTGGGTCACCAGCAAATCGCGCTGTTAGCGGGCCCATTAAGTTCTGTCTCGGCGCGTCTGCGTCTGGCTGGCTGGCATAAATA
TCTCACTCGCAATCAAATTCAGCCGATAGCGGAACGGGAAGGCGACTGGAGTGCCATGTCCGGTTTTCAACAAACCATGCAAATGCTGAATGAGGGCATC
GTTCCCACTGCGATGCTGGTTGCCAACGATCAGATGGCGCTGGGCGCAATGCGCGCCATTACCGAGTCCGGGCTGCGCGTTGGTGCGGATATCTCGGTAG
TGGGATACGACGATACCGAAGATAGCTCATGTTATATCCCGCCGTTAACCACCATCAAACAGGATTTTCGCCTGCTGGGGCAAACCAGCGTGGACCGCTT
GCTGCAACTCTCTCAGGGCCAGGCGGTGAAGGGCAATCAGCTGTTGCCAGTCTCACTGGTGAAAAGAAAAACCACCCTGGCGCCCAATACGCAAACCGCC
TCTCCCCGCGCGTTGGCCGATTCATTAATGCAGCTGGCACGACAGGTTTCCCGACTGGAAAGCGGGCAGTGATAATTGGTAACGAATCAGACAATTGACG
GCTCGAGGGAGTAGCATAGGGTTTGCAGAATCCCTGCTTCGTCCATTTGACAGGCACATTATGCATCGATGATAAGCTGTCAAACATGAGCACGCTTACT
AGTAGCGGCCGCTGCAGTCCGGCAAAAAAGGGCAAGGTGTCACCACCCTGCCCTTTTTCTTTAAAACCGAAAAGATTACTTCGCGTTATGCAGGCTTCCT
CGCTCACTGACTCGCTGCGCTCGGTCGTTCGGCTGCGGCGAGCGGTATCAGCTCACTCAAAGGCGGTAATGACAGTAAGACGGGTAAGCCTGTTGATGAT
ACCGCTGCCTTACTGGGTGCATTAGCCAGTCTGAATGACCTGTCACGGGATAATCCGAAGTGGTCAGACTGGAAAATCAGAGGGCAGGAACTGCTGAACA
GCAAAAAGTCAGATAGCACCACATAGCAGACCCGCCATAAAACGCCCTGAGAAGCCCGTGACGGGCTTTTCTTGTATTATGGGTAGTTTCCTTGCATGAA
TCCATAAAAGGCGCCTGTAGTGCCATTTACCCCCATTCACTGCCAGAGCCGTGAGCGCAGCGAACTGAATGTCACGAAAAAGACAGCGACTCAGGTGCCT
GATGGTCGGAGACAAAAGGAATATTCAGCGATTTGCCCGAGCTTGCGAGGGTGCTACTTAAGCCTTTAGGGTTTTAAGGTCTGTTTTGTAGAGGAGCAAA
CAGCGTTTGCGACATCCTTTTGTAATACTGCGGAACTGACTAAAGTAGTGAGTTATACACAGGGCTGGGATCTATTCTTTTTATCTTTTTTTATTCTTTC
TTTATTCTATAAATTATAACCACTTGAATATAAACAAAAAAAACACACAAAGGTCTAGCGGAATTTACAGAGGGTCTAGCAGAATTTACAAGTTTTCCAG
CAAAGGTCTAGCAGAATTTACAGATACCCACAACTCAAAGGAAAAGGACTAGTAATTATCATTGACTAGCCCATCTCAATTGGTATAGTGATTAAAATCA
CCTAGACCAATTGAGATGTATGTCTGAATTAGTTGTTTTCAAAGCAAATGAACTAGCGATTAGTCGCTATGACTTAACGGAGCATGAAACCAAGCTAATT
TTATGCTGTGTGGCACTACTCAACCCCACGATTGAAAACCCTACAAGGAAAGAACGGACGGTATCGTTCACTTATAACCAATACGCTCAGATGATGAACA
TCAGTAGGGAAAATGCTTATGGTGTATTAGCTAAAGCAACCAGAGAGCTGATGACGAGAACTGTGGAAATCAGGAATCCTTTGGTTAAAGGCTTTTGGAT
TTTCCAGTGGACAAACTATGCCAAGTTCTCAAGCGAAAAATTAGAATTAGTTTTTAGTGAAGAGATATTGCCTTATCTTTTCCAGTTAAAAAAATTCATA
AAATATAATCTGGAACATGTTAAGTCTTTTGAAAACAAATACTCTATGAGGATTTATGAGTGGTTATTAAAAGAACTAACACAAAAGAAAACTCACAAGG
CAAATATAGAGATTAGCCTTGATGAATTTAAGTTCATGTTAATGCTTGAAAATAACTACCATGAGTTTAAAAGGCTTAACCAATGGGTTTTGAAACCAAT
AAGTAAAGATTTAAACACTTACAGCAATATGAAATTGGTGGTTGATAAGCGAGGCCGCCCGACTGATACGTTGATTTTCCAAGTTGAACTAGATAGACAA
ATGGATCTCGTAACCGAACTTGAGAACAACCAGATAAAAATGAATGGTGACAAAATACCAACAACCATTACATCAGATTCCTACCTACATAACGGACTAA
GAAAAACACTACACGATGCTTTAACTGCAAAAATTCAGCTCACCAGTTTTGAGGCAAAATTTTTGAGTGACATGCAAAGTAAGTATGATCTCAATGGTTC
GTTCTCATGGCTCACGCAAAAACAACGAACCACACTAGAGAACATACTGGCTAAATACGGAAGGATCTGAGGTTCTTATGGCTCTTGTATCTATCAGTGA
AGCATCAAGACTAACAAACAAAAGTAGAACAACTGTTCACCGTTACATATCAAAGGGAAAACTGTCCATATGCACAGATGAAAACGGTGTAAAAAAGATA
GATACATCAGAGCTTTTACGAGTTTTTGGTGCATTCAAAGCTGTTCACCATGAACAGATCGACAATGTAACAGATGAACAGCATGTAACACCTAATAGAA
CAGGTGAAACCAGTAAAACAAAGCAACTAGAACATGAAATTGAACACCTGAGACAACTTGTTACAGCTCAACAGTCACACATAGACAGCCTGAAACAGGC
GATGCTGCTTATCGAATCAAAGCTGCCGACAACACGGGAGCCAGTGACGCCTCCCGTGGGGAAAAAATCATGGCAATTCTGGAAGAAATAGCGCTTTCAG
CCGGCAAACCGGCTGAAGCCGGATCTGCGATTCTGATAACAAACTAGCAACACCAGAACAGCCCGTTTGCGGGCAGCAAAACCCGTACCGATTATCAAAA
AGGATCTTCACCTAGATCCTTTTAAATTAAAAATGAAGTTTTAAATCAATCTAAAGTATATATGAGTAAACTTGGTCTGACAGTTACCAATGCTTAATCA
GTGAGGCACCTATCTCAGCGATCTGTCTATTTCGTTCATCCATAGTTGCCTGACTCCCCGTCGTGTAGATAACTACGATACGGGAGGGCTTACCATCTGG
CCCCAGTGCTGCAATGATACCGCGAGAACCACGCTCACCGGCTCCAGATTTATCAGCAATAAACCAGCCAGCCGGAAGGGCCGAGCGCAGAAGTGGTCCT
GCAACTTTATCCGCCTCCATCCAGTCTATTAATTGTTGCCGGGAAGCTAGAGTAAGTAGTTCGCCAGTTAATAGTTTGCGCAACGTTGTTGCCATTGCTA
CAGGCATCGTGGTGTCACGCTCGTCGTTTGGTATGGCTTCATTCAGCTCCGGTTCCCAACGATCAAGGCGAGTTACATGATCCCCCATGTTGTGCAAAAA

		AGCGGTTAGCTCCTTCGGTCCTCCGATCGTTGTCAGAAGTAAGTTGGCCGCAGTGTTATCACTCATGGTTATGGCAGCACTGCATAATTCTCTTACTGTC<br>ATGCCATCCGTAAGATGCTTTTCTGTGACTGGTGAGTACTCAACCAAGTCATTCTGAGAATAGTGTATGCGGCGACCGAGTTGCTCTTGCCCGGCGTCAA<br>TACGGGATAATACCGCGCCACATAGCAGAACTTTAAAAGTGCTCATCATTGGAAAACGTTCTTCGGGGCGAAAACTCTCAAGGATCTTACCGCTGTTGAG<br>ATCCAGTTCGATATAACCCACTCGTGCACCCAACTGATCTTCAGCATCTTTTACTTTCACCAGCGTTTCTGGGTGAGCAAAAACAGGAAGGCAAAATGCC<br>GCAAAAAAGGGAATAAGGGCGACACGGAAATGTTGAATACTCATACTCTTCCTTTTTCAATATTATTGAAGCATTTATCAGGGTTATTGTCTCATGAGCG<br>GATACATATTTGAATGTATTTAGAAAAATAAACAAATAGGGGTTCCGCG
pEG2192	PapoA	AGCAAGAAAGAATGGCAAGAGCCCACGATCGAAGTGCTCGATATTAATCAGACTATGGCGGGTAAGGGCTGGAAACAGATAGACTGGGTGAGCGACCATG<br>ATGCTGACTTACACAATCCGTCTTAATAA
pEG2194	BamA	CTGAAAATCCGCAAGGTGAAAATTGTCAGAGCGCAGAACGGCCACTACACGAACTAATAA
pEG2195	EpiA	GAAGCAGTTAAAGAGAAGAACGATCTGTTCAACCTGGATGTTAAAGTCAACGCAAAAGAAAGTAACGATAGTGGCGCAGAACCACGCATAGCGTCGAAAT<br>TTATTTGCACACCAGGCTGCGCGAAAACGGGTTCGTTTAACAGCTATTGTTGTTAATAA
pEG2199	HalA1	ACGAACTTGCTGAAAGAATGGAAAATGCCCCTGGAACGTACGCATAATAACTCCAACCCGGCGGGAGACATTTTTCAGGAACTGGAAGATCAAGACATAC<br>TCGCCGGTGTGAATGGAGCAGAAAACTTATACTTTCAGGGTTGTGCGTGGTATAACATTAGCTGCCGTCTGGGCAACAAAGGAGCCTACTGCACCCTTAC<br>AGTTGAGTGCATGCCCTCCTGTAACTGATAA
pEG2200	HalA2	GTGAATTCCAAAGACCTGAGAAATCCAGAATTTCGCAAAGCTCAGGGTCTGCAGTTTGTAGATGAAGTTAATGAGAAGGAACTCTCGAGTTTAGCCGGCA<br>GCGAGAATCTTTACTTTCAAGGCACGACGTGGCCATGTGCGACCGTCGGCGTTTCAGTTGCCTTGTGCCCGACGACCAAATGCACTTCACAGTGCTGATA<br>A
pEG2312	PapA_tev	TTGAAACAGATCAATGTGATTGCTGGCGTAAAAGAGCCTATTCGCGCCTATGAGAACTTGTATTTCCAGGGTTGTTCGGCTAATGACGCATGCTATTTTT<br>GCGACACGCGTGACAACTGCAAAGCCTGTGATGCCAGTGATTTTTGTATCAAAAGTGATACG
pEG2571	TruE_tev	AACAAGAAGAACATTTTACCGCAGTTAGGACAACCAGTCATCCGCCTTACTGCCGGTCAACTGTCAAGCCAACTGGCGGAGCTTTCTGAGGAGGCTCTGG<br>GAGAGAACTTGTATTTCCAGGGTGTCGATGCCTCGACCTTGCCGGTTCCGACGTTGTGTAGCTATGACGGGGTGGACGCTAGCACAGTCCCTACACTTTG<br>TAGTTACGATGAC
pEG2575	PsnA2_tev	ATGAGCAAAAATGAGAACAACAAGAAACAGCTGCGCGATCTTTTCATTGAAGATCTGGGCAAAGTTACTGGCGAGAACTTGTATTTCCAGGGTAAAGGTG<br>GCCCGTATACCACCTTAGCCATTGGCGAAGAAGATCCGATTACCACTTTGGCTATCGGAGAAGAGGACCCTGATCCAACGACACTTGCCTTAGGTGAAGA<br>GGACCCAACTACGCTTGCAATCGGCGAAGAA
pEG3157	MibA	ATGCCAGCCGATATTCTGGAGACTCGTACCAGCGAAACGGAGGACTTACTGGATCTTGACCTGAGCATCGGTGTAGAAGAAATCACCGCAGGCCCGGCAG<br>TGACTTCTTGGTCACTGTGCACCCCTGGATGCACGAGTCCGGGCGGTGGCTCCAATTGTTCGTTCTGTTGCTAATAA
pEG3161	PlpA1	ATGAGCATTGAGAATGCCAAGAGCTTTTATGAACGCGTCAGTACAGATAAGCAGTTCCGCACTCAACTGGAAAATACGGCCAGTGCTGAAGAACGGCAGA<br>AAATCATTCAGGCAGCGGGCTTTGAGTTCACCAATCAGGAGTGGGAAATTGCAAAAGAACAGATTCTTGCGACAAGTGAAAGTAATAACGGTGAACTGTC<br>CGAGGCCGAACTGACCGCCGTCAGCGGTGGGGTTGACTTAAGCATTTTCGAGCTGCTGGACGAAGAACCTTTATTCCCGATTCGTCCTTTGTACGGCCTG<br>CCTATTTAATAA
pEG3162	PlpA2	ATGTCTATTGAGAGTGCAAAGGCTTTCTACCAGCGTATGACGGATGACGCATCTTTTCGTACCCCTTTTGAAGCGGAACTGTCGAAAGAGGAGCGCCAAC<br>AATTAATCAAAGATAGCGGATATGACTTTACTGCAGAAGAATGGCAACAGGCTATGACCGAGATCCAGGCGGCACGCTCAAACGAGGAACTGAATGAGGA<br>AGAACTCGAGGCAATTGCCGGGGGCGCTGTGGCCGCAATGTATGGTGTGGTTTTCCCATGGGACAACGAGTTCCCGTGGCCCCGCTGGGGCGGTTAATAA
pEG3165	PbtA	ATGAACCTGAACGATTTACCTATGGACGTCTTTGAAATGGCAGACAGCGGTATGGAGGTGGAAAGCCTCACGGCTGGCCATGGCATGCCAGAAGTTGGAG<br>CTAGTTGCAACTGTGTGTGCGGGTTTTGCTGCAGCTGCAGTCCGAGCGCGTAATAA
pEG3172	LtnA1	ATGAATAAAAACGAAATCGAAACCCAGCCAGTTACGTGGCTGGAGGAAGTTTCTGATCAGAATTTTGATGAGGATGTCTTTGGTGCGTGTAGCACAAACA<br>CCTTCTCGCTGAGCGATTACTGGGGTAACAACGGTGCTTGGTGTACACTCACGCACGAATGTATGGCATGGTGCAAGTAATAA
pEG3173	LtnA2	ATGAAGGAAAAGAATATGAAGAAAAACGACACCATCGAACTTCAGCTTGGAAAATACCTGGAAGATGATATGATCGAACTGGCTGAAGGGGATGAGTCCC<br>ATGGGGGTACTACCCCGGCTACCCCTGCGATTTCTATCCTCAGCGCGTATATCAGCACCAATACCTGCCCGACAACTAAGTGTACACGCGCGTGCTAATA<br>A
pEG3174	CrnA1	ATGTCCGAACTGAGTATGGAGAAAGTGGTCGGCGAAACATTTGAGGATCTGAGCATCGCGGAAATGACGATGGTGCAGGGCAGCGGCGACATTAACGGCG<br>AATTTACTACCTCGCCGGCATGTGTTTATTCCGTTATGGTTGTATCGAAAGCAAGCAGCGCTAAATGTGCGGCCGGTGCATCGGCAGTCTCGGGAGCCAT<br>TCTGAGTGCGATTCGTTGCTAATAA
pEG3175	CrnA2	ATGAGCGAATCCAACATGAAGAAGGTTGTTGGCGAAACCTTCGAAGATCTGAGCATCGCAGAAATGACGAAAGTTCAGGGCTCAGGGGACGTGATGCCGG<br>AATCTACCCCAATTTGTGCCGGCTTCGCAACCTTGATGAGTTCTATCGGTCTTGTTAAAACCATCAAAGGCAATGTCAAAAGTTTCTCCGTCTTAATTTA<br>ATAA
pEG3176	BsjA2	ATGACCAATGAAGAGATCATTGTCGCGTGGAAAAACCCTAAAGTCCGTGGCAAAAATATGCCAAGTCACCCGAGCGGCGTGGGATTCCAAGAGCTTTCCA<br>TCAACGAGATGGCCCAAGTGACCGGCGGAGCAGTAGAACAGCGTGCAACACCAACCCTGGCAACCCCGCTGACCCCGCATACCCCGTACGCAACCTATGT<br>GGTTAGCGGAGGCGTGGTTAGCGCGATTTCTGGTATCTTCAGCAACAATAAAACGTGTCTGGGCTAATAA
pEG3177	BsjA3	ATGACCAATGAGGAAATTATCGTTGCGTGGAAAAACCCGAAGGTGCGCGGCAAAAACATGCCTTCCCATCCGTCCGGTGTGGGCTTCCAGGAATTATCTA<br>TTAATGAAATGGCACAGGTGACTGGTGGCGCGGTTGAACAGCGCGCGACGCCGGCAACCCCAGCAACACCATGGCTGATTAAAGCGTCTTATGTGGTGAG<br>TGGGGCGGGAGTTTCTTTTGTCGCAAGCTATATCACTGTAAACTAATAA
pEG3178	CinA	ATGACGGCGAGTATTCTTCAGTCTGTCGTTGATGCGGACTTTCGTGCGGCCCTGATTGAAAACCCAGCCGCATTCGGCGCGAGCACCGCAGTTTTGCCGA<br>CCCCAGTCGAACAGCAGGATCAGGCATCACTGGATTTTTGGACAAAAGATATTGCTGCCACTGAGGCGTTTGCTTGCAAACAGTCTTGCTCATTTGGGCC<br>GTTCACCTTTGTGTGCGACGGGAATACCAAATAATAA
pEG3180	LasA	ATGGACAAACGTGTGCGTTACGAAAAACCGAGCCTGGTGAAAGAGGGTACGTTTCGCAAAACTACCGCTGGCCTGCGGCGTCTGTTCGCTGACCAGCTGG<br>TTGGCCGCCGTAACATTTAATAA
pEG3181	AlbsA	ATGGATTCACTGCTGTCAACAGAAACCGTCATTAGTGATGACGAACTGCTTCCGATTGAAGTTGGTGGTACCGCGGAATTGACAGAGGGGCAGGGCGGCG<br>GTCAGTCCGAGGATAAACGTCGCGCTTATAACTGCTAATAA
pEG3182	McbA	ATGGAATTAAAAGCGAGTGAATTTGGTGTAGTTTTGTCCGTTGATGCTCTTAAATTATCACGCCAGTCTCCATTAGGTGTTGGCATTGGTGGTGGTGGCG<br>GCGGCGGCGGCGGCGGCGGTAGCTGCGGTGGTCAAGGTGGCGGTTGTGGTGGTTGCAGCAACGGTTGTAGTGGTGGAAACGGTGGCAGCGGCGGAAGTGG<br>TTCACATATCTAATAA
pEG3194	PsnA2	ATGAGCAAAAATGAGAACAACAAGAAACAGCTGCGCGATCTTTTCATTGAAGATCTGGGCAAAGTTACTGGCGGTAAAGGTGGCCCGTATACCACCTTAG<br>CCATTGGCGAAGAAGATCCGATTACCACTTTGGCTATCGGAGAAGAGGACCCTGATCCAACGACACTTGCCTTAGGTGAAGAGGACCCAACTACGCTTGC<br>AATCGGCGAAGAATAATAA
pEG3197	AMdnA	ATGCCGGAAAATCGGCAGGAAGATCTCAACGCTCAGGCTGTACCATTCTTCGCGCGTTTCTTGGAGGGTCAAAACTGCGAGGACCTTACTGATGAGGAAT<br>CGGAGGCGGTTAGCGGTGGAAAACGCGGCCAAACCCGTAAATATCCAAGCGACTGCGAAGATGGGAATGGCGTGACCGGTAAACTGCGCGATGAAGATAT<br>TGCAGTGACCTTGAAGTACCCATCCGACAATGAAGATAATGGCGGCGGTGAAATTGTGACTCTGAAGTTTCCAAGTGATGATGATGATCAACCAGTAGGC<br>TAATAA
pEG3283	PapA	ATGTTGAAACAGATCAATGTGATTGCTGGCGTAAAAGAGCCTATTCGCGCCTATGGTTGTTCGGCTAATGACGCATGCTATTTTTGCGACACGCGTGACA<br>ACTGCAAAGCCTGTGATGCCAGTGATTTTTGTATCAAAAGTGATACGTAATAA

pEG3286	PcpA	ATGTCGAGTAATATCCTCGAAAAAGTTAAGGAGTTTTTCGTCCGGCTGGTGAAGGATGATGCGTTTCAAAGCCAGCTGCAGAACAACAGTATTGATGAAG<br>TTCGAAATATCCTGCAGGAGGCCGGGTACATATTCAGCAAAGAAGAATTCGAAACCGCAACCATTGAATTGCTGGATTTGAAGGAACGCGATGAATTCCA<br>CGAGCTGACAGAAGAGGAGCTTGTCACCGCTGTTGGCGGTGTTACGGGCGGGAGTGGTATATATGGCCCGATTCAAGCTATGTACGGTGCCGTCGTAGGT<br>GATCCAAAACCGGGTAAGGACTGGGGGTGGCGCTTTCCGAGCCCGCTGCCAAAACCGAGTCCGATTCCGAGTCCGTGGAAACCCCCGGTTGATGTCCAGC<br>CTATGTATGGTGTGGTAGTGTCAAACGATAGTTAATAA
pEG3563	PadeA	AAAAAGCAATATAGCAAACCTAGCCTGGAGGTTCTGGACGTCCACCAGACCATGGCTGGCCCGGGCACTAGTACGCCAGACGCGTTTCAGCCAGATCCAG<br>ATGAAGATGTTCACTATGATTCGTAATAA
pEG3564	ThcoA	CGCAAGAAAGAATGGCAGACACCAGAACTGGAAGTACTCGATGTACGCCTCACCGCAGCGGGCCCGGGTAAAGCTAAACCGGATGCTGTGCAGCCAGACG<br>AAGATGAAATAGTGCACTACTCATAATAA
pEG3565	StspA	AAGAAATTCTATGAAGCGCCAGCTCTCATCGAACGTGGCGCCTTTGCGGCTGCTACAGCGGGGTTTGGACGTCTGCTGGCGGATCAGCTGGTGGGACGCC<br>TGATTCCGTAATAA
pEG3567	LcnA	ACTAAAGGCCTGGACAAAATGCTTTTAACCAAAAAGAAGAAGGATAGTATGGGTCTGCTGAACGAAATCGACGTTACCACCCTGGATGAACAGTTAGGCG<br>GTAAAATGAGCAAAGCATGGTGCCGATCCATGGTGGTGTCCTGCGTGTATAACCTGGTTGATTTTTCGTCGTCGAGTGACGGGAAAAAGACATGTGCTCT<br>GTACCGCAAATATTGTTAATAA
pEG3568	PalA	AAAGATCTTCTGAAGGAACTGATGTATGAAGTAGACCTCGAAGAGATGGAGAATCTTCAGGGTAGCGGGTACTCAGCCGCCCAGTGTGCCTGGATGGCGC<br>TGAGCTGCGTCAATTACATCCCGGGAGTGGGATTCGGTTGTGGCGGCTACAGCGCATGTGAACTCTACAAGCGTTATTGTTAATAA
pEG3570	RaxX	AACCACTCTAAGAAAAGTCCGGCAAAAGGGGCAGCGTCCCTGCAGCGTCCTGCTGGGGCAAAAGGCCGCCCTGAACCTCTGGATCAACGCTTGTGGAAAC<br>ACGTCGGTGGTGGTGACTACCCACCCCCAGGAGCCAACCCAAAGCATGATCCACCACCCCGCAATCCGGGCCACCATTAATAA
pEG3571	ComX	CAAGATCTGATTAATTACTTCCTGAATTATCCTGAGGCTCTGAAGAAACTCAAGAATAAGGAAGCCTGCTTAATTGGGTTTGACGTCCAGGAAACCGAAA<br>CGATTATCAAAGCCTATAACGATTACTACCGCGCTGATCCGATCACGCGTCAATGGGGTGATTAATAA
pEG3572	KgpE	AAGAACCCGACGCTGTTGCCCAAACTGACCGCGCCGGTCGAACGTCCGGCCGTAACTTCGTCGGATTTAAAGCAAGCCTCAAGCGTCGATGCTGCATGGT<br>TAAATGGCGATAATAACTGGTCAACCCCATTCGCCGGTGTGAACGCGGCATGGTTAAATGGGGACAACAACTGGTCCACGCCTTTTGCGGGCGTGAATGC<br>TGCATGGCTTAATGGCGACAATAACTGGAGCACTCCATTTGCCGCCGATGGCGCTGAGTAATAA
pEG3574	TgnA*	TATCGACCTTATATTGCCAAGTATGTCGAAGAACAAACTCTGCAGAATTCAACCAACCTGGTATATGACGACATCACGCAGATCTCTTTTATCAATAAAG<br>AAAAGAACGTGAAAAAAATTAATCTGGGTCCCGATACTACGATCGTGACTGAAACCATCGAGAATGCGGACCCCGATGAGTATTTCTTATAATAA
pEG3871	SgbA	TCTGGTCGCGGGCGCGATCCTGATGCTGCTGTACCTCCCTTGCCTCGTGTACCTCGCACTACTAATCATGAGCCACGTACGGCGTCCCGAGAACCAAGAG<br>CAGCTCCAAGAACTGGACCTACACGTCCGCCTTCGTCGCGTCCATCTCCGTGTGGTCACTCTCCTCAAACCCCTGGTGCAGGACGCAGTGGATGTCGTGT<br>GGAGCGTCAAAAATCGGCTGCGGCTTCGTCTGAGAAGGAAAAGACAATGGAGAACCAAGATTTGGAGTTATTAGCACGCCTGCATGCACTTCCTGAGACT<br>GAACCGGTGGGCGTCGACGGATTACCCTATGGCGAGACTTGTGAGTGCGTCGGGTTACTTACGTTGTTGAACACCGTATGTATCGGCATTTCATGCGCTT<br>AATAA
pEG3905	TruE	ATGAACAAGAAGAACATTTTACCGCAGTTAGGACAACCAGTCATCCGCCTTACTGCCGGTCAACTGTCAAGCCAACTGGCGGAGCTTTCTGAGGAGGCTC<br>TGGGAGTCGATGCCTCGACCTTGCCGGTTCCGACGTTGTGTAGCTATGACGGGGTGGACGCTAGCACAGTCCCTACACTTTGTAGTTACGATGAC

RST_C expression vector

Backbone Sequence	CACATTTCCCCGAAAAGTGCCACCTGACGTCTAAGAAACCATTATTATCATGACATTAACCTATAAAAATAGGCGTATCACGAGGCAGAATTTCAGATAA<br>AAAAAATCCTTAGCTTTCGCTAAGGATGATTTCTGGAATTCGCGGCCGCTTCTAGAGGGAGAACGATCGTTGGCTGAATCATAAAAAATTTATTTGCTTT<br>GTGAGCGGATAACAATTATAATAGATTCAATTGTGAGCGGATAACAATTCGAACCCAACACCACCAGCAAGCCTAAGGAGGAGAAATATGTCATATTACC<br>ACCATCACCATCATCACATGAGCAAAGGAGAAGAACTTTTCACTGGAGTTGTCCCAATTCTTGTTGAATTAGATGGTGATGTTAATGGGCACAAATTTTC<br>TGTCCGTGGAGAGGGTGAAGGTGATGCTACAAACGGAAAACTCACCCTTAAATTTATTTGCACTACTGGAAAACTACCTGTTCCGTGGCCAACACTTGTC<br>ACTACTCTGACCTATGGTGTTCAATGCTTTTCCCGTTATCCGGATCACATGAAACGGCATGACTTTTTCAAGAGTGCCATGCCCGAAGGTTATGTACAGG<br>AACGCACTATATCTTTCAAAGATGACGGGACCTACAAGACGCGTGCTGAAGTCAAGTTTGAAGGTGATACCCTTGTTAATCGTATCGAGTTAAAGGGTAT<br>TGATTTTAAAGAAGATGGAAACATTCTTGGACACAAACTCGAGTACAACTTTAACTCACACAATGTATACATCACGGCAGACAAACAAAAGAATGGAATC<br>AAAGCTAACTTCAAAATTCGCCACAACGTTGAAGATGGTTCCGTTCAACTAGCAGACCATTATCAACAAAATACTCCAATTGGCGATGGCCCTGTCCTTT<br>TACCAGACAACCATTACCTGTCGACACAATCTGTCCTTTCGAAAGATCCCAACGAAAAGCGTGACCACATGGTCCTTCTTGAGTTTGTAACTGCTGCTGG<br>GATTACACATGGCATGGATGAGCTCTACAAACGACTGGTTCCGCGTGGTAGCTATTACGACTCCATTCCCACAAGCGAGAACGACTCAGAAGTCAATCAA<br>GAAGCTAAGCCAGAGGTCAAGCCAGAAGTCAAGCCTGAGACTCACATCAATTTAAAGGTGTCCGATGGATCTTCAGAGATCTTCTTCAAGATCAAAAAGA<br>CCACTCCTTTAAGAAGGCTGATGGAAGCGTTCGCTAAAAGACAGGGTAAGGAAATGGACTCCTTAAGATTCTTGTACGACGGTATTAGAATTCAAGCTGA<br>TCAGGCCCCTGAAGATTTGGACATGGAGGATAACGATATTATTGAGGCTCACCGCGAACAGATTGGAGGCTCCATTACAAGCCACCATCACCATCATCAC<br>GGTTAATACTTTCAGCCAAAAAACTTAAGACCGCCGGTCTTGTCCACTACCTTGCAGTAATGCGGTGGACAGGATCGGCGGTTTTCTTTTCTCTTCTCAA<br>CAAGTGAGACCATGGGCGGCGCGCCATCGAATGGCGCAAAACCTTTCGCGGTATGGCATGATAGCGCCCGGAAGAGAGTCAATTCAGGGTGGTGAATATG<br>AAACCAGTAACGTTATACGATGTCGCAGAGTATGCCGGTGTCTCTTATCAGACCGTTTCCCGCGTGGTGAACCAGGCCAGCCACGTTTCTGCGAAAACGC<br>GGGAAAAAGTGGAAGCGGCGATGGCGGAGCTGAATTACATTCCCAACCGCGTGGCACAACAACTGGCGGGCAAACAGTCGTTGCTTATTGGCGTTGCCAC<br>CTCCAGTCTGGCCCTGCACGCGCCGTCGCAAATTGTCGCGGCGATTAAATCTCGCGCCGATCAACTGGGTGCCAGCGTGGTGGTGTCGATGGTAGAACGA<br>AGCGGCGTCGAAGCCTGTAAAGCGGCGGTGCACAATCTTCTCGCGCAACGCGTCAGTGGGCTGATCATTAACTATCCGCTGGATGACCAGGATGCCATTG<br>CTGTGGAAGCTGCCTGCACTAATGTTCCGGCGTTATTTCTTGATGTCTCTGACCAGACACCCATCAACAGTATTATTTTCTCCCATGAGGACGGTACGCG<br>ACTGGGCGTGGAGCATCTGGTCGCATTGGGTCACCAGCAAATCGCGCTGTTAGCGGGCCCATTAAGTTCTGTCTCGGCGCGTCTGCGTCTGGCTGGCTGG<br>CATAAATATCTCACTCGCAATCAAATTCAGCCGATAGCGGAACGGGAAGGCGACTGGAGTGCCATGTCCGGTTTTCAACAAACCATGCAAATGCTGAATG<br>AGGGCATCGTTCCCACTGCGATGCTGGTTGCCAACGATCAGATGGCGCTGGGCGCAATGCGCGCCATTACCGAGTCCGGGCTGCGCGTTGGTGCGGATAT<br>CTCGGTAGTGGGATACGACGATACCGAAGATAGCTCATGTTATATCCCGCCGTTAACCACCATCAAACAGGATTTTCGCCTGCTGGGGCAAACCAGCGTG<br>GACCGCTTGCTGCAACTCTCTCAGGGCCAGGCGGTGAAGGGCAATCAGCTGTTGCCAGTCTCACTGGTGAAAAGAAAAACCACCCTGGCGCCCAATACGC<br>AAACCGCCTCTCCCCGCGCGTTGGCCGATTCATTAATGCAGCTGGCACGACAGGTTTCCCGACTGGAAAGCGGGCAGTGATAATTGGTAACGAATCAGAC<br>AATTGACGGCTCGAGGGAGTAGCATAGGGTTTGCAGAATCCCTGCTTCGTCCATTTGACAGGCACATTATGCATCGATGATAAGCTGTCAAACATGAGCA<br>CGCTTACTAGTAGCGGCCGCTGCAGTCCGGCAAAAAGGGCAAGGTGTCACCACCCTGCCCTTTTTCTTTAAAACCGAAAAGATTACTTCGCGTTATGCA<br>GGCTTCCTCGCTCACTGACTCGCTGCGCTCGGTCGTTCGGCTGCGGCGAGCGGTATCAGCTCACTCAAAGGCGGTAATGACAGTAAGACGGTAAGCCTG<br>TTGATGATACCGCTGCCTTACTGGGTGCATTAGCCAGTCTGAATGACCTGTCACGGGATAATCCGAAGTGGTCAGACTGGAAAATCAGAGGGCAGGAACT<br>GCTGAACAGCAAAAAGTCAGATAGCACCACATAGCAGACCCGCCATAAAACGCCCTGAGAAGCCCGTGACGGGCTTTTCTTGTATTATGGGTAGTTTCCT<br>TGCATGAATCCATAAAAGGCGCCTGTAGTGCCATTTACCCCCATTCACTGCCAGAGCCGTGAGCGCAGCGAACTGAATGTCACGAAAAAGACAGCGACTC<br>AGGTGCCTGATGGTCGGAGACAAAAGGAATATTCAGCGATTTGCCCGAGCTTGCGAGGGTGCTACTTAAGCCTTTAGGGTTTTAAGGTCTGTTTTGTAGA<br>GGAGCAAACAGCGTTTGCGACATCCTTTTGTAATACTGCGGAACTGACTAAAGTAGTGAGTTATACACAGGGCTGGGATCTATTCTTTTTATCTTTTTTT<br>ATTCTTTCTTTATTCTATAAATTATAACCACTTGAATATAAACAAAAAAAACACACAAAGGTCTAGCGGAATTTACAGAGGGTCTAGCAGAATTTACAAG<br>TTTTCCAGCAAAGGTCTAGCAGAATTTACAGATACCCACAACTCAAAGGAAAAGGACTAGTAATTATCATTGACTAGCCCATCTCAATTGGTATAGTGAT<br>TAAAATCACCTAGACCAATTGAGATGTATGTCTGAATTAGTTGTTTTCAAAGCAAATGAACTAGCGATTAGTCGCTATGACTTAACGGAGCATGAAACCA<br>AGCTAATTTTATGCTGTGTGGCACTACTCAACCCCACGATTGAAAACCCTACAAGGAAAGAACGGACGGTATCGTTCACTTATAACCAATACGCTCAGAT<br>GATGAACATCAGTAGGGAAAATGCTTATGGTGTATTAGCTAAAGCAACCAGAGAGCTGATGACGAGAACTGTGGAAATCAGGAATCCTTTGGTTAAAGGC<br>TTTTGGATTTTCCAGTGGACAAACTATGCCAAGTTCTCAAGCGAAAAATTAGAATTAGTTTTTAGTGAAGAGATATTGCCTTATCTTTTCCAGTTAAAAA<br>AATTCATAAAATATAATCTGGAACATGTTAAGTCTTTTGAAAACAAATACTCTATGAGGATTTATGAGTGGTTATTAAAAGAACTAACACAAAAGAAAAC<br>TCACAAGGCAAATATAGAGATTAGCCTTGATGAATTTAAGTTCATGTTAATGCTTGAAAATAACTACCATGAGTTTAAAAGGCTTAACCAATGGGTTTTG<br>AAACCAATAAGTAAAGATTTAAACACTTACAGCAATATGAAATTGGTGGTTGATAAGCGAGGCCGCCCGACTGATACGTTGATTTTCCAAGTTGAACTAG<br>ATAGACAAATGGATCTCGTAACCGAACTTGAGAACAACCAGATAAAAATGAATGGTGACAAAATACCAACAACCATTACATCAGATTCCTACCTACATAA<br>CGGACTAAGAAAAACACTACACGATGCTTTAACTGCAAAAATTCAGCTCACCAGTTTTGAGGCAAAATTTTTGAGTGACATGCAAAGTAAGTATGATCTC<br>AATGGTTCGTTCTCATGGCTCACGCAAAAACAACGAACCACACTAGAGAACATACTGGCTAAATACGGAAGGATCTGAGGTTCTTATGGCTCTTGTATCT<br>ATCAGTGAAGCATCAAGACTAACAAACAAAAGTAGAACAACTGTTCACCGTTACATATCAAAGGGAAAACTGTCCATATGCACAGATGAAAACGGTGTAA<br>AAAAGATAGATACATCAGAGCTTTTACGAGTTTTTGGTGCATTCAAAGCTGTTCACCATGAACAGATCGACAATGTAACAGATGAACAGCATGTAACACC<br>TAATAGAACAGGTGAAACCAGTAAAACAAAGCAACTAGAACATGAAATTGAACACCTGAGACAACTTGTTACAGCTCAACAGTCACACATAGACAGCCTG<br>AAACAGGCGATGCTGCTTATCGAATCAAAGCTGCCGACAACACGGGAGCCAGTGACGCCTCCCGTGGGAAAAAATCATGGCAATTCTGGAAGAAATAGC

		GCTTTCAGCCGGCAAACCGGCTGAAGCCGGATCTGCGATTCTGATAACAAACTAGCAACACCAGAACAGCCCGTTTGCGGGCAGCAAAACCCGTACCGAT<br>TATCAAAAAGGATCTTCACCTAGATCCTTTTAAATTAAAAATGAAGTTTTAAATCAATCTAAAGTATATATGAGTAAACTTGGTCTGACAGTTACCAATG<br>CTTAATCAGTGAGGCACCTATCTCAGCGATCTGTCTATTTCGTTCATCCATAGTTGCCTGACTCCCCGTCGTGTAGATAACTACGATACGGGAGGGCTTA<br>CCATCTGGCCCCAGTGCTGCAATGATACCGCGAGAACCACGCTCACCGGCTCCAGATTTATCAGCAATAAACCAGCCAGCCGGAAGGGCCGAGCGCAGAA<br>GTGGTCCTGCAACTTTATCCGCCTCCATCCAGTCTATTAATTGTTGCCGGGAAGCTAGAGTAAGTAGTTCGCCAGTTAATAGTTTGCGCAACGTTGTTGC<br>CATTGCTACAGGCATCGTGGTGTCACGCTCGTCGTTTGGTATGGCTTCATTCAGCTCCGGTTCCCAACGATCAAGGCGAGTTACATGATCCCCCATGTTG<br>TGCAAAAAAGCGGTTAGCTCCTTCGGTCCTCCGATCGTTGTCAGAAGTAAGTTGGCCGCAGTGTTATCACTCATGGTTATGGCAGCACTGCATAATTCTC<br>TTACTGTCATGCCATCCGTAAGATGCTTTTCTGTGACTGGTGAGTACTCAACCAAGTCATTCTGAGAATAGTGTATGCGGCGACCGAGTTGCTCTTGCCC<br>GGCGTCAATACGGGATAATACCGCGCCACATAGCAGAACTTTAAAAGTGCTCATCATTGGAAAACGTTCTTCGGGGCGAAAACTCTCAAGGATCTTACCG<br>CTGTTGAGATCCAGTTCGATATAACCCACTCGTGCACCCAACTGATCTTCAGCATCTTTTACTTTCACCAGCGTTTCTGGGTGAGCAAAAACAGGAAGGC<br>AAAATGCCGCAAAAAAGGGAATAAGGGCGACACGGAAATGTTGAATACTCATACTCTTCCTTTTTCAATATTATTGAAGCATTTATCAGGGTTATTGTCT<br>CATGAGCGGATACATATTTGAATGTATTTAGAAAAATAAACAAATAGGGGTTCCGCG
pEG3212	CapA	ATGGTGCGTTTCCTGGCTAAGCTGCTGCGTTCAACGATCCATGGCTCTAATGGCGTGAGCCTCGACGCCGTCAGTTCCACGCATGGTACTCCGGGGTTTC<br>AGACACCTGATGCACGTGTTATTTCACGCTTTGGCTTTAAT
pEG3213	LasA	ATGGACAAACGTGTGCGTTACGAAAAACCGAGCCTGGTGAAAGAGGGTACGTTTCGCAAAACTACCGCTGGCCTGCGGCGTCTGTTCGCTGACCAGCTGG<br>TTGGCCGCCGTAACATT
pEG3214	AlbsA	ATGGATTCACTGCTGTCAACAGAAACCGTCATTAGTGATGACGAACTGCTTCCGATTGAAGTTGGTGGTACCGCGGAATTGACAGAGGGGCAGGGCGGCG<br>GTCAGTCCGAGGATAAACGTCGCGCTTATAACTGC
pEG3215	AtxA1	CCGATCATTAGCGAAACGGTCCAGCCTAAAACGGCTGGCCTGATTGTTCTGGGCAAGGCAAGCGCGGAAACGCGCGGATTGAGCCAAGGCGTGGAACCGG<br>ACATTGGTCAGACGTACTTCGAAGAAAGCCGTATTAATCAGGAT
pEG3553	Cln1A1	ACTCCCATTCAATCCAAATTCTGCCTCCTGCGCGTGGGCAGTGCCAAACGGCTGACGCAGTCATTCGACGTGGGAACTATTAAGGAAGGTTTAGTCAGCC<br>AGTATTATTTTGCG
pEG3554	Cln1A2	ACCCAGGTGAGCCCATCACCGCTGCGCCTGATTCGCGTCGGGAGAGCCTTGGACCTGACCCGCTCTATCGGGGATAGTGGGCTGCGTGAGTCCATGTCAA<br>GCCAGACGTACTGGCCC
pEG3555	Cln2A1	AACACTTTAAAAACGCGTCTTATTCGCTTTGGGTCGGCTAAACGTCTGACGCGCGCAGGTACGGGCGTGCTGTTACCTGAAACCAACCAGATTAAGCGCT<br>ACGATCCAGCA
pEG3556	Cln2A2	ACCACACCCAAATTTCGACTGATTCGGTTAGGTTCAGCTAAGCGATTGACCCGGTCGGGAATCGGGGATGTGTTTCCGGAGCCAAACATGGTTCGCCGCT<br>GGGAT
pEG3557	Cln3A1	CAGCGTATAATAGATGAAACCACCGATGGTCTGATTGAACTGGGGGCGGCCAGCGTACAGACACAGGGCGATGTTTTGTTTGCTCCGGAGCCTGGCGTGG<br>GCCGACCTCCAATGGGCCTTTCCGAAGAT
pEG3558	Cln3A2	GAACGCATTGAAGATCATATTGATGATGAACTGATTGACCTGGGAGCTGCTTCGGTTGAAACCCAGGGAGATGTGCTGAATGCACCGGAGCCTGGTATCG<br>GTCGTGAACCGACAGGCTTGAGCCGCGAT
pEG3559	Cln3A3	GAATTTGAAGGTATCCCATCACCGGATGCGCGTATTGATTTGGGTCTGGCGTCGGAAGAAACCTGTGGTCAGATTTATGATCACCCGGAAGTAGGCATCG<br>GTGCGTACGGGTGCGAGGGCCTGCAGCGT
pEG3560	CsegA1	ACCAAGAAAAACGCAACACAGGCCCCACGTTTAGTACGTGTAGGCGATGCTCATCGTTTGACCCAAGGTGCTTTCGTTGGACAGCCGGAAGCCGTAAATC<br>CACTTGGACGTGAAATTCAAGGA
pEG3561	CsegA2	ACCAAAACACACAGACTGATCAGATTGGGCGACGCGCAACGCTTGACCCAGGGCACATTGACTCCGGGCTTACCGGAGGACTTTCTGCCGGGCCATTACA<br>TGCCGGGG
pEG3562	CsegA3	ACTTCACGTTTCCAACTCCTGCGCCTGGGAAAAGCCGATCGTTTGACGCGTGGCGCGCTGGTCGGGCTCCTGATCGAAGATATTACTGTCGCTCGCTACG<br>ACCCTATG
**Lux Mod Backbone**		
pEG1128	TruD	AACACCCCTTGTATTACTGTTTATGTAAGCAGACAGTTTTATTGTTCATGATGATATATTTTTATCTTGTGCAATGTACATCAGAGATTTTGAGACACAA<br>CCAATTATTGAAGGCCTCCCTAACGGGGGGCCTTTTTTTGTTTCTGGTCTCCACGACGCTTAACGATCGTTGGCTGACCTGTAGGATCGTACAGGTTTAC<br>GCAAGAAAATGGTTTGTTACAGTCGAATAAACAGCCCCATAGGGTGGTGTGTACCACCCCTGATGAGTCCAAAAGGACGAAATGGGGCCTCTACAAATAA<br>TTTTGTTTAAAGCCACACTCGAATTACTCAAAGGACCTCTAGCAATGCAACCAACCGCCCTCCAAATTAAGCCCCACTTCCACGTTGAGATAATTGAGCC<br>GAAGCAAGTGTATCTCCTGGGCGAACAGGGCAACCACGCTCTCACCGGGCAGCTCTACTGCCAAATTCTGCCTTTCTTAAACGGCGAATACACCCGAGAA<br>CAAATTGTGGAAAAGCTCGATGGGCAGGTCCCGGAGGAATATATCGACTTCGTACTCAGTCGTCTGGTGGAGAAGGGCTATCTAACTGAGGTGGCTCCAG<br>AACTATCCCTGGAAGTGGCAGCATTTTGGAGCGAATTGGGAATTGCCCCTTCTGTAGTGGCAGAAGGGCTAAAGCAGCCAGTGACAGTGACAACGGCGGG<br>CAAGGGCATTAGGGAAGGGATAGTGGCTAACCTGGCAGCAGCGCTGGAGGAAGCTGGCATTCAGGTGTCAGACCCAAGGGACCCAAAGGCCCCAAAGGCA<br>GGGGATTCTACTGCCCAGCTTCAGGTGGTGCTGACCGATGACTATTTACAGCCGGAACTTGCAGCGATCAACAAGGAAGCCTTAGAGCGCCAACAACCCT<br>GGTTGCTGGTTAAGCCTGTGGGCAGTATCCTCTGGTTGGGACCGTTGTTCGTTCCTGGGGAAACCGGATGTTGGCACTGTCTTGCTCAACGATTGCAAGG<br>CAACCGGGAAGTTGAAGCATCGGTATTGCAACAAAAGCGAGCGCTGCAGGAGCGCAACGGTCAAAATAAAAATGGTGCAGTGAGTTGCTTGCCCACAGCA<br>CGGGCAACCCTACCTTCTACTCTACAAACAGGTTTACAGTGGGCTGCCACTGAGATTGCTAAGTGGATGGTCAAGCGGCACCTCAATGCCATAGCACCGG<br>GAACGGCTCGTTTTCCCACTCTAGCTGGCAAGATATTTACATTCAACCAGACGACTCTGGAGTTGAAAGCTCATCCTCTGAGCCGACGACCGCAATGTCC<br>CACCTGTGGCGATCGGGAAACTCTCCAACGGCGCGGGTTTGAACCACTGAAGCTAGAGTCGCGCCCCAAACACTTCACCTCCGATGGCGGTCATCGCGCC<br>ATGACCCCAGAACAAACGGTGCAGAAGTACCAACACCTCATCGGGCCCATAACGGGGGTAGTGACGGAACTGGTGCGAATTTCTGACCCTGCCAATCCCT<br>TGGTGCATACCTACCGGGCTGGGCATAGCTTTGGCAGTGCTACGTCTCTGCGGGGGCTGCGCAATGTCCTACGCCACAAGAGTTCTGGTAAAGGCAAGAC<br>CGATAGCCAATCTCGGGCCAGCGGACTTTGCGAGGCGATCGAGCGCTATTCGGGCATTTTTCAGGGAGACGAACCCCGCAAGCGGGCAACTTTGGCTGAG<br>TTGGGAGATTTGGCGATTCATCCAGAACAGTGTTTGCACTTTAGCGACAGGCAGTATGACAACCGGGAAAGCTCGAACGAGCGAGCAACAGTGACTCACG<br>ACTGGATTCCCCAACGGTTCGATGCAAGTAAGGCTCACGACTGGACTCCCGTGTGGTCCCTAACGGAGCAAACCCATAAGTATCTGCCTACAGCCCTGTG<br>CTATTACCGATACCCCTTCCCCCCAGAACACCGTTTCTGCCGTAGTGACTCCAACGGAAACGCGGCGGGAAATACCCTGGAAGAGGCGATTTTGCAAGGA<br>TTTATGGAACTGGTGGAACGGGATAGCGTGTGCCTGTGGTGGTACAATCGCGTTAGCCGTCCGGCTGTGGATTTGAGTAGCTTTGACGAGCCTTATTTTT<br>TGCAGTTGCAGCAGTTCTATCAAACTCAAAATCGCGATCTGTGGGTACTGGATTTAACAGCAGATTTGGGCATTCCGGCTTTTGTAGGGGTATCGAATCG<br>GAAAGCCGGCAGCTCGGAAAGAATAATTCTCGGCTTTGGAGCGCACCTGGACCCGACAGTTGCCATCCTTCGCGCTCTTACGGAGGTCAACCAAATAGGC<br>TTGGAATTGGATAAAGTTTCTGATGAGAGCCTCAAGAACGATGCCACGGATTGGTTAGTGAATGCTACATTGGCAGCTAGTCCCTATCTCGTTGCCGATG<br>CTAGCCAACCCCTCAAGACTGCGAAGGATTATCCCCGGCGTTGGAGTGACGATATTTACACCGATGTGATGACTTGTGTAGAAATAGCCAAGCAAGCAGG<br>TCTAGAGACTTTGGTACTGGATCAGACCAGACCCGACATAGGTTTAAATGTGGTTAAAGTCATTGTGCCAGGAATGCGTTTTTGGTCGCGATTTGGCTCC<br>GGTCGGCTCTATGACGTGCCAGTGAAGTTGGGATGGCGAGAGCAACCACTTGCTGAGGCACAAATGAACCCTACACCGATGCCATTTTAATAAGATACGA<br>ATTTATGTATAGACTCGGTACCAAAAAAAAAAAAAAAAGACGCTGAAAAGCGTCTTTTTTTTTTTTTGGTCCTACTATCCTTAAACGCATATCGTGGTACAG<br>GAGACCGTCCAATGGCGGCGCGCCATCGAATGGCGCAAAACCTTTCGCGGTATGGCATGATAGCGCCCGGAAGAGAGTCAATTCAGGGTGGTGAATATGA<br>AAAACATAAATGCCGACGACACATACAGAATAATTAATAAAATTAAAGCTTGTAGAAGCAATAATGATATTAATCAATGCTTATCTGATATGACTAAAAT<br>GGTACATTGTGAATATTATTTACTCGCGATCATTTATCCTCATTCTATGGTTAAATCTGATATTTCAATCCTAGATAATTACCCTAAAAAATGGAGGCAA<br>TATTATGATGACGCTAATTTAATAAAATATGATCCTATAGTAGATTATTCTAACTCCAATCATTCACCAATTAATTGGAATATATTTGAAAACAATGCTG<br>TAAATAAAAAATCTCCAAATGTAATTAAAGAAGCGAAAACATCAGGTCTTATCACTGGGTTTAGTTTCCCTATTCATACGGCTAACAATGGCTTCGGAAT<br>GCTTAGTTTTGCACATTCAGAAAAAGACAACTATATAGATAGTTTATTTTTACATGCGTGTATGAACATACCATTAATTGTTCCTTCTCTAGTTGATAAT<br>TATCGAAAAATAAATATAGCAAATAATAAATCAAACAACGATTTAACCAAAAGAGAAAAAGAATGTTTAGCGTGGCATGCGAAGGAAAAAGCTCTTGGG<br>ATATTTCAAAAATATTAGGTTGCAGTGAGCGTACTGTCACTTTCCATTTAACCAATGCGCAAATGAAACTCAATACAACAAACCGCTGCCAAAGTATTTC<br>TAAAGCAATTTTAACAGGAGCAATTGATTGCCCATACTTTAAAAATTGATAAGGATCCTAATTGGTAACGAATCAGACAATTGACGGCTCGAGGGAGTAG<br>CATAGGGTTTGCAGAATCCCTGCTTCGTCCATTTGACAGGCACATTATGCATCGATGATAAGCTGTCAAACATGAGCAGATCCTCTACGCCGGACGCATC<br>GTGGCCGGCATCACCGGCGCCACAGGTGCGGTTGCTGGCGCCTATATCGCCGACATCACCGATGGGGAAGATCGGGCTCGCCACTTCGGGCTCATGAGCA<br>AATATTTTATCTGAGGTGCTTCCTCGCTCACTGACTCGCTGCACGAGGCAGACCTCAGCGCTAGCGGAGTGTATACTGGCTTACTATGTTGGCACTGATG<br>AGGGTGTCAGTGAAGTGCTTCATGTGGCAGGAGAAAAAAGGCTGCACCGGTGCGTCAGCAGAATATGTGATACAGGATATATTCCGCTTCCTCGCTCACT

GACTCGCTACGCTCGGTCGTTCGACTGCGGCGAGCGGAAATGGCTTACGAACGGGGCGGAGATTTCCTGGAAGATGCCAGGAAGATACTTAACAGGGAAG
TGAGAGGGCCGCGGCAAAGCCGTTTTTCCATAGGCTCCGCCCCCCTGACAAGCATCACGAAATCTGACGCTCAAATCAGTGGTGGCGAAACCCGACAGGA
CTATAAAGATACCAGGCGTTTCCCCCTGGCGGCTCCCTCGTGCGCTCTCCTGTTCCTGCCTTTCGGTTTACCGGTGTCATTCCGCTGTTATGGCCGCGTTT
GTCTCATTCCACGCCTGACACTCAGTTCCGGGTAGGCAGTTCGCTCCAAGCTGGACTGTATGCACGAACCCCCCGTTCAGTCCGACCGCTGCGCCTTATC
CGGTAACTATCGTCTTGAGTCCAACCCGGAAAGACATGCAAAAGCACCACTGGCAGCAGCCACTGGTAATTGATTTAGAGGAGTTAGTCTTGAAGTCATG
CGCCGGTTAAGGCTAAACTGAAAGGACAAGTTTTGGTGACTGCGCTCCTCCAAGCCAGTTACCTCGGTTCAAAGAGTTGGTAGCTCAGAGAACCTTCGAA
AAACCGCCCTGCAAGGCGGTTTTTTCGTTTTCAGAGCAAGAGATTACGCGCAGACCAAAACGATCTCAAGAAGATCATCTTATTAAGGGGTCTGACGCTC
AGTGGAACGAAAAATCAATCTAAAGTATATATGAGTAAACTTGGTCTGACAGTTACCTTAGAAAAACTCATCGAGCATCAAATGAAACTGCAATTTATTC
ATATCAGGATTATCAATACCATATTTTTGAAAAAGCCGTTTCTGTAATGAAGGAGAAAACTCACCGAGGCAGTTCCATAGGATGGCAAGATCCTGGTATC
GGTCTGCGATTCCGACTCGTCCAACATCAATACAACCTATTAATTTCCCCTCGTCAAAAATAAGGTTATCAAGTGAGAAATCACCATGAGTGACGACTGA
ATCCGGTGAGAATGGCAAAAGCTTATGCATTTCTTTCCAGACTTGTTCAACAGGCCAGCCATTACGCTCGTCATCAAAATCACTCGCATCAACCAAACCG
TTATTCATTCGTGATTGCGCCTGAGCGAGACGAAATACGCGATCGCTGTTAAAAGGACAATTACAAACAGGAATCGAATGCAACCGGCGCAGGAACACTG
CCAGCGCATCAACAATATTTTCACCTGAATCAGGATATTCTTCTAATACCTGGAATGCTGTTTTCCCGGGGATCGCAGTGGTGAGTAACCATGCATCATC
AGGAGTACGGATAAAATGCTTGATGGTCGGAAGAGGCATAAATTCCGTCAGCCAGTTTAGTCTGACCATCTCATCTGTAACATCATTGGCAACGCTACCT
TTGCCATGTTTCAGAAACAACTCTGGCGCATCGGGCTTCCCATACAATCGATAGATTGTCGCACCTGATTGCCCGACATTATCGCGAGCCCATTTATACC
CATATAAATCAGCATCCATGTTGGAATTTAATCGCGGCCTCGAGCAAGACGTTTCCCGTTGAATATGGCTCAT

Cym Mod Backbone without SapI sites around CDS

Backbone Sequence

AACACCCCTTGTATTACTGTTTATGTAAGCAGACAGTTTTATTGTTCATGATGATATATTTTTATCTTGTGCAATGTACATCAGAGATTTTGAGACACAA
CCAATTATTGAAGGCCTCCCTAACGGGGGGCCTTTTTTTGTTTCTGGTCTCCCAAGCGCTTAACGATCGTTGGCTGAACAAACAGACAATCTGGTCTGTT
TGTATTATGGAAAATTTTTCTGTATAATAGATTCAACAAACAGACAATCTGGTCTGTTTGTATTATCAGCGGTCAACGCATGTGCTTTGCGTTCTGATGA
GACAGTGATGTCGAAACCGCCTCTACAAATAATTTTGTTTATCACACAGGAAAGTACTAGATGAGCAAAGGAGAAGAACTTTTCACTGGAGTTGTCCCAA
TTCTTGTTGAATTAGATGGTGATGTTAATGGGCACAAATTTTCTGTCCGTGGAGAGGGTGAAGGTGATGCTACAAACGGAAAACTCACCCTTAAATTTAT
TTGCACTACTGGAAAACTACCTGTTCCGTGGCCAACACTTGTCACTACTCTGACCTATGGTGTTCAATGCTTTTCCCGTTATCCGGATCACATGAAACGG
CATGACTTTTTCAAGAGTGCCATGCCCGAAGGTTATGTACAGGAACGCACTATATCTTTCAAAGATGACGGGACCTACAAGACGCGTGCTGAAGTCAAGT
TTGAAGGTGATACCCTTGTTAATCGTATCGAGTTAAAGGGTATTGATTTTAAAGAAGATGGAAACATTCTTGGACACAAACTCGAGTACAACTTTAACTC
ACACAATGTATACATCACGGCAGACAAACAAAAGAATGGAATCAAAGCTAACTTCAAAATTCGCCACAACGTTGAAGATGGTTCCGTTCAACTAGCAGAC
CATTATCAACAAAATACTCCAATTGGCGATGGCCCTGTCCTTTTACCAGACAACCATTACCTGTCGACACAATCTGTCCTTTCGAAAGATCCCAACGAAA
AGCGTGACCACATGGTCCTTCTTGAGTTTGTAACTGCTGCTGGGATTACACATGGCATGGATGAGCTCTACAAATAAGGATGAGCTCTACAAATAAGCAG
AGGTGGTTGTGTTGCGAAAAAAAAAAAAAAACACCCTAACGGGTGTTTTTTTTTTTTTGGTGTCCCCACGTGTGGCGCTGGAGACCGTCCAATGGCGGCG
CGCCATCGAATGGTGCAAAACCTTTCGCGGTATGGCATGATAGCGCCCGGAAGAGAGTCAATTCAGGGTGGTGAATATGAGCCCGAAACGTCGTACCCAG
GCAGAACGTGCAATGGAAACCCAGGGTAAACTGATTGCAGCAGCACTGGGTGTTCTGCGTGAAAAAGGTTATGCAGGTTTTCGTATTGCAGATGTTCCGG
GTGCAGCCGGTGTTAGCCGTGGTGCACAGAGCCATCATTTTCCGACCAAACTGGAACTGCTGCTGGCAACCTTTGAATGGCTGTATGAGCAGATTACCGA
ACGTAGCCGTGCACGTCTGGCAAAACTGAAACCGGAAGATGATGTTATTCAGCAGATGCTGGATGATGCAGCAGATTTTTTTCTGGATGATGATTTTAGC
ATCGGCCTGGATCTGATTGTTGCAGCAGATCGTGATCCGGCACTGCGTGAAGGTATTCTGCGTACCGTTGAACGTAATCGTTTTGTTGTTGAAGATATGT
GGCTGGGTGTGCTGGTGAGCCGTGGTCTGAGCCGTGATGATGCCGAAGATATTCTGTGGCTGATTTTTAACAGCGTTCGTGGTCTGACAGTTCGTAGCCT
GTGGCAGAAAGATAAAGAACGTTTTGAACGTGTGCGTAATAGCACCCTGGAAATTGCACGTGAACGTTATGCAAAATTCAAACGTTGATAAGGATCCTAA
TTGGTAACGAATCAGACAATTGACGGCTCGAGGGAGTAGCATAGGGTTTGCAGAATCCCTGCTTCGTCCATTTGACAGGCACATTATGCATCGATGATAA
GCTGTCAAACATGAGCAGATCCTCTACGCCGGACGCATCGTGGCCGGCATCACCGGCGCCACAGGTGCGGTTGCTGGCGCCTATATCGCCGACATCACCG
ATGGGGAAGATCGGGCTCGCCACTTCGGGCTCATGAGCAAATATTTTATCTGAGGTGCTTCCTCGCTCACTGACTCGCTGCACGAGGCAGACCTCAGCGC
TAGCGGAGTGTATACTGGCTTACTATGTTGGCACTGATGAGGGTGTCAGTGAAGTGCTTCATGTGGCAGGAGAAAAAAGGCTGCACCGGTGCGTCAGCAG
AATATGTGATACAGGATATATTCCGCTTCCTCGCTCACTGACTCGCTACGCTCGGTCGTTCGACTGCGGCGAGCGGAAATGGCTTACGAACGGGGCGGAG
ATTTCCTGGAAGATGCCAGGAAGATACTTAACAGGGAAGTGAGAGGGCCGCGGCAAAGCCGTTTTTCCATAGGCTCCGCCCCCCTGACAAGCATCACGAA
ATCTGACGCTCAAATCAGTGGTGGCGAAACCCGACAGGACTATAAAGATACCAGGCGTTTCCCCCTGGCGGCTCCCTCGTGCGCTCTCCTGTTCCTGCCTT
TCGGTTTACCGGTGTCATTCCGCTGTTATGGCCGCGTTTGTCTCATTCCACGCCTGACACTCAGTTCCGGGTAGGCAGTTCGCTCCAAGCTGGACTGTAT
GCACGAACCCCCCGTTCAGTCCGACCGCTGCGCCTTATCCGGTAACTATCGTCTTGAGTCCAACCCGGAAAGACATGCAAAAGCACCACTGGCAGCAGCC
ACTGGTAATTGATTTAGAGGAGTTAGTCTTGAAGTCATGCGCCGGTTAAGGCTAAACTGAAAGGACAAGTTTTGGTGACTGCGCTCCTCCAAGCCAGTTA
CCTCGGTTCAAAGAGTTGGTAGCTCAGAGAACCTTCGAAAAACCGCCCTGCAAGGCGGTTTTTTCGTTTTCAGAGCAAGAGATTACGCGCAGACCAAAAC
GATCTCAAGAAGATCATCTTATTAAGGGGTCTGACGCTCAGTGGAACGAAAAATCAATCTAAAGTATATATGAGTAAACTTGGTCTGACAGTTACCTTAG
AAAAACTCATCGAGCATCAAATGAAACTGCAATTTATTCATATCAGGATTATCAATACCATATTTTTGAAAAAGCCGTTTCTGTAATGAAGGAGAAAACT
CACCGAGGCAGTTCCATAGGATGGCAAGATCCTGGTATCGGTCTGCGATTCCGACTCGTCCAACATCAATACAACCTATTAATTTCCCCTCGTCAAAAAT
AAGGTTATCAAGTGAGAAATCACCATGAGTGACGACTGAATCCGGTGAGAATGGCAAAAGCTTATGCATTTCTTTCCAGACTTGTTCAACAGGCCAGCCA
TTACGCTCGTCATCAAAATCACTCGCATCAACCAAACCGTTATTCATTCGTGATTGCGCCTGAGCGAGACGAAATACGCGATCGCTGTTAAAAGGACAAT
TACAAACAGGAATCGAATGCAACCGGCGCAGGAACACTGCCAGCGCATCAACAATATTTTCACCTGAATCAGGATATTCTTCTAATACCTGGAATGCTGT
TTTCCCGGGGATCGCAGTGGTGAGTAACCATGCATCATCAGGAGTACGGATAAAATGCTTGATGGTCGGAAGAGGCATAAATTCCGTCAGCCAGTTTAGT
CTGACCATCTCATCTGTAACATCATTGGCAACGCTACCTTTGCCATGTTTCAGAAACAACTCTGGCGCATCGGGCTTCCCATACAATCGATAGATTGTCG
CACCTGATTGCCCGACATTATCGCGAGCCCATTTATACCCATATAAATCAGCATCCATGTTGGAATTTAATCGCGGCCTCGAGCAAGACGTTTCCCGTTG
AATATGGCTCAT

pEG7172 HalM1

CCAATCAAGGAGGTAGAAAACATAATGCGCGAACTCCAAAATGCGCTTTACTTTAGCGAAGTGGTTTTTGGACCGAATCTTGAGAAGATTGTAGGAGAAA
AGCGCCTCAATTTTTGGCTCAAACTTATAGGTGAGGACCCGGAAAACCTGAAGGAGTTTCTCTCGAGAAAGGGCAATTCTTTCGAAGAACAAACCTTACC
GGAAAAGGAAGCTATCGTTCCGAACCGCTTAGGTGAAGAGGCGCTGGAAAAAGTCCGCGAAGAACTTGAGTTCCTCAATACTTACAGCACTAAACATGTG
CGTCGCGTTAAAGAGTTGGGAGTGCAGATCCCTTTCGAAGGGATTCTGCTGCCATTCATTAGCATGTATATCGAAAAATTTCAGCAGCAGCAACTTCGCA
AAAAGATAGGGCCGATTCACGAAGAGATCTGGACGCAGATTGTTCAAGATATCACCTCCAAATTAAATGCGATTCTGCACCGTACCCTGATCCTGGAACT
GAATGTAGCTCGTGTTACCTCCCAACTTAAAGGTGATACTCCGGAAGAAAGATTCGCCTACTACTCGAAAACCTATTTAGGCAAACGTGAAGTAACTCAC
CGTCTGTATAGCGAATATCCGGTGGTTCTGCGGTTGCTGTTCACCACCATTTCACACCACATTTCGTTCATTACGGAAATCCTTGAACGCGTTGCAAATG
ACCGTGAAGCCATTGAAACCGAATTTTCACCGTGTTCCCCGATTGGTACCCTCGCCTCTCTCCACTTAAACTCGGGAGATGCTCACCATAAACAGCGTAC
TGTGACGATTTTGGAATTCTCCTCCTCGCTGAAACTTGTCTACAAACCTCGCTCCCTCAAAGTTGATGGGGTGTTCAACGGTTTACTCGCTTTCCTGAAC
GATAGAACGGGGGAAGTCATTAAGGACCAGTATTGCCCTAAGGTGTTACAGCGCGATGGCTACGGCTATGTGGAATTTGTCACTCACCAGTCTTGTCAAT
CCCTTGAGGAAGTGTCAGACTTCTACGAGAGACTCGGCTCTCTGATGAGTCTGTCCTACGTACTGAATAGTTCTGACTTTCATTTCGAGAACATTATAGC
TCATGGTCCCTATCCTGTCCTGATCGATCTTGAAACCATCATTCATAATACAGCGGATAGCAGCGAGGAAACGTCTACCGCTATGGATCGCGCGTTCCGT
ATGTTGAACGATTCGGTGCTGTCCACTGGTATGCTTCCCTCCTCTATTTATTATCGCGATCAGCCGAATATGAAGGGTCTGAACGTCGGAGGTGTGAGCA
AATCAGAAGGTCAGAAAACACCGTTCAAAGTTAATCAAATCGCCAATCGCAACACCGATGAGATGCGTATCGAAAAAGATCACGTTACCCTGAGCAGCCA
GAAAAATCTGCCCATTTTTCAGTCTGCCGCAATGGAGAGCGTACATTTCTTAGATCAGATCCAGAAAGGCTTTACCTCCATGTATCAGTGGATCGAGAAG
AACAAACAAGAATTTAAAGAACAGGTGCGTAAGTTTGAAGGTGTGCCGGTTCGTGCTGTTCTTCGGAGCACGACTCGCTATACCGAACTGCTGAAATCTT
CCTACCACCCTGACCTGCTCCGCAGCGCGTTGGACCGTGAAGTACTGCTGAACCGTTTGACTGTTGACTCGGTAATGACCCCGTATCTCAAAGAGATTAT
TCCACTCGAGGTGGAAGATCTGCTGAACGGTGACGTGCCATACTTCTACACCCTGCCGGAAGAACGCGCCCTGTATCAGGAAGCGTCTGCGATCAATAGT
ACGTTCTTTACCACTTCGATTTTCCATAAGATTGACCAGAAAATCGATAAGCTGGGTATCGAGGACCATACCCAGCAAATGAAGATCTTACACATGAGTA
TGCTTGCCTCTAACGCTAACCATTACGCCGATGTTGCCGACTTGGATATTCAGAAAGGACACACCATTAAAAACGAACAGTACGTTGAGATGGCCAAAGA
CATCGGTGATTACCTGATGGAGTTATCGGTCGAGGGTGAAAATCAAGGGGAACCAGATCTGTGTTGGATTTCGACCGTCCTGGAAGGGAGCTCTGAAATC
ATTTGGGACATCAGCCCAGTGGGCGAAGATTTATACAACGGCAGCGCTGGCGTCGCTCTCTTTTATGCGTACCTGTTCAAAATTACAGGTGAAAAGCGTT
ACCAAGAGATCGCATACAAAGCCCTGGTTCCGGTTCGCCGCAGTGTGGCCCAATTCCAGCACCATCCGAATTGGAGCATTGGTGCGTTTAACGGAGCGTC
AGGCTATCTGTACGCGATGGGTACGATAGCGGCCCTGTTTAATGATGAACGTTTGAAGCATGAAGTAACCCGCAGCATTCCGCACATTGAACCGATGATC
CACGAGGATAAGATCTATGATTTCATTGGCGGTTCCGCAGGGGCGCTGAAGGTGTTCCTGAGCCTGTCGGGGCTGTTTGACGAGCCGAAGTTTTTGGAAC
TTGCCATTGCATGCAGCGAACATCTGATGAAAAACGCCATTAAAACGGATCAAGGTATCGGCTGGAAACCACCGTGGGAGGTCACCCCACTGACCGGTTT
CAGCCATGGGGTTAGCGGCGTCATGGCATCCTTCATCGAACTGTACCAGCAAACCGGTGATGAGCGCTTGCTCAGTTACATTGATCAGAGTTTAGCCTAT
GAACGTTCCTTCTTCAGCGAACAAGAGGAGAACTGGCTGACTCCGAACAAAGAAACACCCGTGGTAGCTTGGTGCCACGGCGCGCCGGGAATTTTGGTAT
CACGACTGCTTCTGAAGAAATGCGGCTATTTGGATGAAAAAGTCGAAAAAGAAATTGAGGTGGCATTATCCACAACTATCCGTAAAGGCCTTGGTAACAA
TCGCAGTCTTTGCCATGGTGATTTCGGCCAGCTGGAAATTCTTCGCTTTGCGGCGGAAGTGTTAGGCGATAGCTATCTCCAGGAAGTTGTCAACAATCTG
TCCGGCGAGTTGTATAATCTTTTCAAAACGGAGGGATATCAGAGCGGAACCAGCCGCGGTACTGAATCCGTGGGCCTGATGGTAGGTCTGTCCGGGTTTG
GGTATGGTTTACTTTCAGCGGCATATCCATCTGCTGTCCCCTCAATCTTAACATTGGATGGTGAGATCCAGAAGTACCGGGAGCCTCATGAAGCCTGA

pEG7173 HalM2

TAAAACCGCTCGTAAGGAGGTCTTATGAAAACGCCGCTGACCTCGGAACATCCTTCAGTGCCGACGACGCTGCCGCATACTAACGACACCGATTGGCTCG
AGCAATTACATGACATTTTGTCCATTCCTGTTACGGAAGAAATCCAGAAATATTTCCACGCCGAAAATGATCTGTTCTCGTTTTTCTATACACCGTTCCT
GCAGTTTACGTACCAGAGCATGTCGGACTACTTTATGACCTTCAAGACCGATATGGCCCTGATCGAAAGACAGAGCCTCCTGCAAAGCACGCTGACCGCG
GTACATCACCGACTCTTCCACTTAACGCATCGCACCCTTATTAGTGAAATGCATATTGATAAACTTACCGTTGGCCTGAATGGCTCTACGCCGCACGAGC

GCTACATGGATTTCAACCACAAATTCAACAAAACCTCGAAGTCGAAGAACCTGTTTAACATCTACCCAATTTTGGGAAAATTGGTCGTTAACGAAACTCT
GCGCACTATTAACTTCGTCAAGAAAATCATTCAGCACTACATGAAGGACTACCTGCTCCTGTCGGACTTCTTCAAAGAGAAGGACTTGCGTCTTACCAAC
CTGCAATTAGGCGTGGGGGATACACACGTTAATGGGCAATGCGTCACCATTCTGACGTTTGCATCAGGCCAAAAAGTGGTATACAAACCTAGATCATTGT
CGATAGATAAACAGTTCGGAGAATTCATCGAGTGGGTAAACTCGAAAGGTTTTCAGCCTTCCTTGCGTATCCCTATTGCGATTGATCGTCAAACCTATGG
TTGGTATGAATTCATCCCTCATCAAGAGGCCACCAGCGAAGATGAAATAGAACGCTACTATTCTCGCATCGGTGGTTATCTGGCGATCGCCTACTTGTTC
GGGGCAACCGACCTGCACCTGGATAACCTGATCGCCTGCGGCGAACATCCGATGCTTATTGATTTGGAAACACTCTTTACCAACGATCTCGACTGCTATG
ACAGTGCGTTTCCGTTCCCGGCGCTGGCCCGCGAATTAACCCAATCCGTTTTTGGCACCCTTATGCTTCCCATCACCATCGCGTCGGGGAAACTGCTGGA
TATAGACCTGTCAGCAGTAGGAGGCGGTAAAGGTGTGCAGTCCGAAAAGATCAAAACCTGGGTCATCGTGAATCAGAAAACTGATGAGATGAAGCTGGTC
GAGCAGCCGTATGTTACCGAGAGTTCCCAGAATAAACCAACAGTTAATGGAAAGAGGCGAACATTGGCAATTATATTCCTCATGTCACAGATGGCTTTC
GTAAAATGTACCGCCTGTTTCTGAATGAAATTGATGAGTTAATGGATCATAACGGGCCAATCTTTGCGTTTGAGAGTTGTCAGATTCGTCATGTTTTTCG
AGCTACCCACGTGTATGCGAAATTTTTGGAGGCAAGTACCCACCCAGATTACTTGCAAGAACCTACCAGACGTAATAAACTGTTCGAGTCCTTTTGGAAC
ATCACGTCGCTGATGGCACCGTTCAAGAAAATTGTACCGCACGAAATCGCGGAGTTGGAGAACCATGATATTCCGTACTTCGTCCTGACTTGTGGCGGCA
CCATTGTTAAAGATGGATACGGCCGGGATATCGCAGACCTGTTTCAAAGTAGCTGCATCGAACGTGTAACTCATCGTCTGCAGCAGCTGGGAAGCGAGGA
TGAGGCGCGTCAAATTCGCTACATTAAAAGCAGCCTGGCGACGTTGACCAACGGTGATTGGACCCCATCCCATGAGAAAACCCCGATGTCTCCGGCCTCG
GCCGACCGTGAAGATGGTTACTTCCTGCGCGAGGCTCAGGCCATCGGCGACGACATTTTGGCGCAGCTGATTTGGGAGGATGACCGTCACGCCGCTTACC
TTATTGGCGTAAGCGTGGGCATGAACGAAGCCGTCACTGTGTCACCCCTGACGCCTGGCATCTACGACGGCACACTTGGCATAGTGCTGTTCTTCGATCA
GCTGGCCCAGCAGACCGGCGAAACCCATTATCGCCACGCCGCCGACGCTTTACTGGAAGGAATGTTCAAACAGCTGAAACCTGAACTGATGCCGTCTAGC
GCTTACTTCGGACTGGGTAGCCTGTTCTATGGCCTGATGGTGTTGGGCCTCCAGCGTTCCGACTCGCATATCATTCAGAAAGCGTATGAGTATCTGAAAC
ATTTGGAAGAGTGTGTGCAGCATGAGGAAACGCCAGATTTTGTCTCGGGTTTGTCTGGTGTACTGTATATGCTCACGAAAATTTATCAGCTCACGAATGA
ACCGAGAGTTTTCGAAGTGGCCAAAACCACAGCTTCGCGTCTGTCTGTGCTGCTTGACAGCAAGCAGCCCGACACTGTGCTCACCGGGTTATCCCATGGC
GCCGCAGGATTCGCCCTTGCATTACTGACCTACGGAACCGCTGCAAATGATGAACAGTTGCTGAAACAGGGCCACTCCTATCTGGTGTACGAACGTAATC
GGTTTAACAAACAGGAAAACAACTGGGTTGATTTACGTAAAGGCAACGCGTATCAAACATTTTGGTGCCATGGCGCCCCGGGTATTGGCATCTCACGCCT
CCTGTTAGCGCAATTTTACGATGACGAACTGCTGCATGAAGAGTTAAACGCAGCACTGAACAAGACTATTTCGGACGGCTTCGGCCACAATCACTCACTG
TGTCATGGCGATTTCGGCAACCTCGATCTGTTATTGCTTTATGCCCAATATACGAATAACCCAGAACCAAAGGAACTCGCTCGCAAACTGGCCATAAGCA
GTATCGATCAAGCGCACACGTATGGCTGGAAACTCGGGCTCAATCATAGCGATCAACTGCAGGGTATGATGTTAGGGGTGACTGGTATCGGCTATCAGCT
CCTTCGTCATATAAATCCGACAGTCCCCAGCATTTTGGCACTGGAACTGCCCAGCTCCACGTTAACTGAAAAAGAGCTGAGAATCCATGATCGTTGATAA

Cym Mod Backbone

Backbone Sequence

AACACCCCTTGTATTACTGTTTATGTAAGCAGACAGTTTTATTGTTCATGATGATATATTTTTATCTTGTGCAATGTACATCAGAGATTTTGAGACACAA
CCAATTATTGAAGGCCTCCCTAACGGGGGGCCTTTTTTTGTTTCTGGTCTCCCAAGCGCTTAACGATCGTTGGCTGAACAAACAGACAATCTGGTCTGTT
TGTATTATGGAAAATTTTTCTGTATAATAGATTCAACAAACAGACAATCTGGTCTGTTTGTATTATCAGCGGTCAACGCATGTGCTTTGCGTTCTGATGA
GACAGTGATGTCGAAACCGCCTCTACAAATAATTTTGTTTAAGCTCTTCATCACACAGGAAAGTACTAGATGAGCAAAGGAGAAGAACTTTTCACTGGAG
TTGTCCCAATTCTTGTTGAATTAGATGGTGATGTTAATGGGCACAAATTTTCTGTCCGTGGAGAGGGTGAAGGTGATGCTACAAACGGAAAACTCACCCT
TAAATTTATTTGCACTACTGGAAAACTACCTGTTCCGTGGCCAACACTTGTCACTACTCTGACCTATGGTGTTCAATGCTTTTCCCGTTATCCGGATCAC
ATGAAACGGCATGACTTTTTCAAGAGTGCCATGCCCGAAGGTTATGTACAGGAACGCACTATATCTTTCAAAGATGACGGGACCTACAAGACGCGTGCTG
AAGTCAAGTTTGAAGGTGATACCCTTGTTAATCGTATCGAGTTAAAGGGTATTGATTTTAAAGAAGATGGAAACATTCTTGGACACAAACTCGAGTACAA
CTTTAACTCACACAATGTATACATCACGGCAGACAAACAAAAGAATGGAATCAAAGCTAACTTCAAAATTCGCCACAACGTTGAAGATGGTTCCGTTCAA
CTAGCAGACCATTATCAACAAAATACTCCAATTGGCGATGGCCCTGTCCTTTTACCAGACAACCATTACCTGTCGACACAATCTGTCCTTTCGAAAGATC
CCAACGAAAAGCGTGACCACATGGTCCTTCTTGAGTTTGTAACTGCTGCTGGGATTACACATGGCATGGATGAGCTCTACAAATAATGAAGAGCGCAGAG
GTGGTTGTGTTGCGAAAAAAAAAAAAAACACCCTAACGGGTGTTTTTTTTTTTTTGGTGTCCCCACGTGTGGCGCTGGAGACCGTCCAATGGCGGCGCGC
CATCGAATGGTGCAAAACCTTTCGCGGTATGGCATGATAGCGCCCGGAAGAGAGTCAATTCAGGGTGGTGAATATGAGCCCGAAACGTCGTACCCAGGCA
GAACGTGCAATGGAAACCCAGGGTAAACTGATTGCAGCAGCACTGGGTGTTCTGCGTGAAAAAGGTTATGCAGGTTTTCGTATTGCAGATGTTCCGGGTG
CAGCCGGTGTTAGCCGTGGTGCACAGAGCCATCATTTTCCGACCAAACTGGAACTGCTGCTGGCAACCTTTGAATGGCTGTATGAGCAGATTACCGAACG
TAGCCGTGCACGTCTGGCAAAACTGAAACCGGAAGATGATGTTATTCAGCAGATGCTGGATGATGCAGCAGATTTTTTTCTGGATGATGATTTTAGCATC
GGCCTGGATCTGATTGTTGCAGCAGATCGTGATCCGGCACTGCGTGAAGGTATTCTGCGTACCGTTGAACGTAATCGTTTTGTTGTTGAAGATATGTGGC
TGGGTGTGCTGGTGAGCCGTGGTCTGAGCCGTGATGATGCCGAAGATATTCTGTGGCTGATTTTTAACAGCGTTCGTGGTCTGACAGTTCGTAGCCTGTG
GCAGAAAGATAAAGAACGTTTTGAACGTGTGCGTAATAGCACCCTGGAAATTGCACGTGAACGTTATGCAAAATTCAAACGTTGATAAGGATCCTAATTG
GTAACGAATCAGACAATTGACGGCTCGAGGGAGTAGCATAGGGTTTGCAGAATCCCTGCTTCGTCCATTTGACAGGCACATTATGCATCGATGATAAGCT
GTCAAACATGAGCAGATCCTCTACGCCGGACGCATCGTGGCCGGCATCACCGGCGCCACAGGTGCGGTTGCTGGCGCCTATATCGCCGACATCACCGATG
GGGAAGATCGGGCTCGCCACTTCGGGCTCATGAGCAAATATTTTATCTGAGGTGCTTCCTCGCTCACTGACTCGCTGCACGAGGCAGACCTCAGCGCTAG
CGGAGTGTATACTGGCTTACTATGTTGGCACTGATGAGGGTGTCAGTGAAGTGCTTCATGTGGCAGGAGAAAAAAGGCTGCACCGGTGCGTCAGCAGAAT
ATGTGATACAGGATATATTCCGCTTCCTCGCTCACTGACTCGCTACGCTCGGTCGTTCGACTGCGGCGAGCGGAAATGGCTTACGAACGGGGCGGAGATT
TCCTGGAAGATGCCAGGAAGATACTTAACAGGGAAGTGAGAGGGCCGCGGCAAAGCCGTTTTTCCATAGGCTCCGCCCCCCTGACAAGCATCACGAAATC
TGACGCTCAAATCAGTGGTGGCGAAACCCGACAGGACTATAAAGATACCAGGCGTTTCCCCCTGGCGGCTCCCTCGTGCGCTCTCCTGTTCCTGCCTTTCG
GTTTACCGGTGTCATTCCGCTGTTATGGCCGCGTTTGTCTCATTCCACGCCTGACACTCAGTTCCGGGTAGGCAGTTCGCTCCAAGCTGGACTGTATGCA
CGAACCCCCCGTTCAGTCCGACCGCTGCGCCTTATCCGGTAACTATCGTCTTGAGTCCAACCCGGAAAGACATGCAAAAGCACCACTGGCAGCAGCCACT
GGTAATTGATTTAGAGGAGTTAGTCTTGAAGTCATGCGCCGGTTAAGGCTAAACTGAAAGGACAAGTTTTGGTGACTGCGCTCCTCCAAGCCAGTTACCT
CGGTTCAAAGAGTTGGTAGCTCAGAGAACCTTCGAAAAACCGCCCTGCAAGGCGGTTTTTTCGTTTTCAGAGCAAGAGATTACGCGCAGACCAAAACGAT
CTCAAGAAGATCATCTTATTAAGGGGTCTGACGCTCAGTGGAACGAAAAATCAATCTAAAGTATATATGAGTAAACTTGGTCTGACAGTTACCTTAGAAA
AACTCATCGAGCATCAAATGAAACTGCAATTTATTCATATCAGGATTATCAATACCATATTTTTGAAAAAGCCGTTTCTGTAATGAAGGAGAAAACTCAC
CGAGGCAGTTCCATAGGATGGCAAGATCCTGGTATCGGTCTGCGATTCCGACTCGTCCAACATCAATACAACCTATTAATTTCCCCTCGTCAAAAATAAG
GTTATCAAGTGAGAAATCACCATGAGTGACGACTGAATCCGGTGAGAATGGCAAAAGCTTATGCATTTCTTTCCAGACTTGTTCAACAGGCCAGCCATTA
CGCTCGTCATCAAAATCACTCGCATCAACCAAACCGTTATTCATTCGTGATTGCGCCTGAGCGAGACGAAATACGCGATCGCTGTTAAAAGGACAATTAC
AAACAGGAATCGAATGCAACCGGCGCAGGAACACTGCCAGCGCATCAACAATATTTTCACCTGAATCAGGATATTCTTCTAATACCTGGAATGCTGTTTT
CCCGGGGATCGCAGTGGTGAGTAACCATGCATCATCAGGAGTACGGATAAAATGCTTGATGGTCGGAAGAGGCATAAATTCCGTCAGCCAGTTTAGTCTG
ACCATCTCATCTGTAACATCATTGGCAACGCTACCTTTGCCATGTTTCAGAAACAACTCTGGCGCATCGGGCTTCCCATACAATCGATAGATTGTCGCAC
CTGATTGCCCGACATTATCGCGAGCCCATTTATACCCATATAAATCAGCATCCATGTTGGAATTTAATCGCGGCCTCGAGCAAGACGTTTCCCGTTGAAT
ATGGCTCAT

pEG7034 TruD

AGACACACTCGAATTACTCAAAGGACCTCTAGCAATGCAACCAACCGCCCTCCAAATTAAGCCCCACTTCCACGTTGAGATAATTGAGCCGAAGCAAGTG
TATCTCCTGGGCGAACAGGGCAACCACGCTCTCACCGGGCAGCTCTACTGCCAAATTCTGCCTTTCTTAAACGGCGAATACACCCGAGAACAAATTGTGG
AAAAGCTCGATGGGCAGGTCCCGGAGGAATATATCGACTTCGTACTCAGTCGTCTGGTGGAGAAGGGCTATCTAACTGAGGTGGCTCCAGAACTATCCCT
GGAAGTGGCAGCATTTTGGAGCGAATTGGGAATTGCCCCTTCTGTAGTGGCAGAAGGGCTAAAGCAGCCAGTGACAGTGACAACGGCGGGCAAGGGCATT
AGGGAAGGGATAGTGGCTAACCTGGCAGCAGCGCTGGAGGAAGCTGGCATTCAGGTGTCAGACCCAAGGGACCCAAAGGCCCCAAAGGCAGGGGATTCTA
CTGCCCAGCTTCAGGTGGTGCTGACCGATGACTATTTACAGCCGGAACTTGCAGCGATCAACAAGGAAGCCTTAGAGCGCCAACAACCCTGGTTGCTGGT
TAAGCCTGTGGGCAGTATCCTCTGGTTGGGACCGTTGTTCGTTCCTGGGGAAACCGGATGTTGGCACTGTCTTGCTCAACGATTGCAAGGCAACCGGGAA
GTTGAAGCATCGGTATTGCAACAAAAGCGAGCGCTGCAGGAGCGCAACGGTCAAAATAAAAATGGTGCAGTGAGTTGCTTGCCCACAGCACGGGCAACCC
TACCTTCTACTCTACAAACAGGTTTACAGTGGGCTGCCACTGAGATTGCTAAGTGGATGGTCAAGCGGCACCTCAATGCCATAGCACCGGGAACGGCTCG
TTTTCCCACTCTAGCTGGCAAGATATTTACATTCAACCAGACGACTCTGGAGTTGAAAGCTCATCCTCTGAGCCGACGACCGCAATGTCCCACCTGTGGC
GATCGGGAAACTCTCCAACGGCGCGGGTTTGAACCACTGAAGCTAGAGTCGCGCCCCAAACACTTCACCTCCGATGGCGGTCATCGCGCCATGACCCCAG
AACAAACGGTGCAGAAGTACCAACACCTCATCGGGCCCATAACGGGGGTAGTGACGGAACTGGTGCGAATTTCTGACCCTGCCAATCCCTTGGTGCATAC
CTACCGGGCTGGGCATAGCTTTGGCAGTGCTACGTCTCTGCGGGGGCTGCGCAATGTCCTACGCCACAAGAGTTCTGGTAAAGGCAAGACCGATAGCCAA
TCTCGGGCCAGCGGACTTTGCGAGGCGATCGAGCGCTATTCGGGCATTTTTCAGGGAGACGAACCCCGCAAGCGGGCAACTTTGGCTGAGTTGGGAGATT
TGGCGATTCATCCAGAACAGTGTTTGCACTTTAGCGACAGGCAGTATGACAACCGGGAAAGCTCGAACGAGCGAGCAACAGTGACTCACGACTGGATTCC
CCAACGGTTCGATGCAAGTAAGGCTCACGACTGGACTCCCGTGTGGTCCCTAACGGAGCAAACCCATAAGTATCTGCCTACAGCCCTGTGCTATTACCGA
TACCCCTTCCCCCCAGAACACCGTTTCTGCCGTAGTGACTCCAACGGAAACGCGGCGGGAAATACCCTGGAAGAGGCGATTTTGCAAGGATTTATGGAAC
TGGTGGAACGGGATAGCGTGTGCCTGTGGTGGTACAATCGCGTTAGCCGTCCGGCTGTGGATTTGAGTAGCTTTGACGAGCCTTATTTTTTGCAGTTGCA
GCAGTTCTATCAAACTCAAAATCGCGATCTGTGGGTACTGGATTTAACAGCAGATTTGGGCATTCCGGCTTTTGTAGGGGTATCGAATCGGAAAGCCGGC
AGCTCGGAAAGAATAATTCTCGGCTTTGGAGCGCACCTGGACCCGACAGTTGCCATCCTTCGCGCTCTTACGGAGGTCAACCAAATAGGCTTGGAATTGG
ATAAAGTTTCTGATGAGAGCCTCAAGAACGATGCCACGGATTGGTTAGTGAATGCTACATTGGCAGCTAGTCCCTATCTCGTTGCCGATGCTAGCCAACC
CCTCAAGACTGCGAAGGATTATCCCCGGCGTTGGAGTGACGATATTTACACCGATGTGATGACTTGTGTAGAAATAGCCAAGCAAGCAGGTCTAGAGACT
TTGGTACTGGATCAGACCAGACCCGACATAGGTTTAAATGTGGTTAAAGTCATTGTGCCAGGAATGCGTTTTTGGTCGCGATTTGGCTCCGGTCGGCTCT
ATGACGTGCCAGTGAAGTTGGGATGGCGAGAGCAACCACTTGCTGAGGCACAAATGAACCCTACACCGATGCCATTTTAATAA

pEG7035 AlbA

AGAATCAAGCAAGTCAAAGGAGTTAACCCGAATGTTTATAGAGCAGATGTTTCCATTTATTAATGAAAGTGTAAGAGTTCACCAGCTTCCTGAGGGCGGC
GTGTTAGAAATCGACTACTTGCGCGATAATGTCTCCATTTCTGACTTTGAGTATTTGGATCTCAACAAAACGGCTTACGAGCTCTGCATGCGCATGGATG

GCCAAAAAACAGCTGAGCAGATTTTAGCTGAGCAATGTGCAGTGTATGATGAATCACCGGAAGATCATAAAGATTGGTATTACGACATGCTCAACATGCT
CCAGAACAAGCAGGTTATTCAGCTTGGAAACCGGGCCAGCCGCCATACAATCACCACGAGCGGAAGCAATGAATTTCCGATGCCCCTGCACGCCACCTTT
GAACTGACGCACCGCTGTAATTTGAAATGCGCCCACTGTTATTTGGAAAGCTCACCTGAAGCGCTCGGCACCGTGTCGATTGAGCAATTCAAAAAAACGG
CTGATATGCTGTTTGATAACGGTGTATTGACATGCGAAATCACAGGTGGAGAAATTTTTGTCCATCCAAACGCCAATGAGATTCTTGACTATGTGTGTAA
AAAGTTCAAAAAAGTCGCTGTCTTAACAAACGGAACACTCATGCGAAAAGAGAGCCTGGAGCTTTTGAAAACTTACAAGCAAAAAATCATCGTCGGCATT
TCTCTAGATAGTGTCAATTCCGAGGTCCATGACTCCTTTAGAGGGAGAAAAGGCTCTTTTGCCCAAACTTGTAAAACGATAAAATTGTTGAGTGACCACG
GTATATTTGTCAGAGTCGCTATGTCTGTATTCGAAAAAAACATGTGGGAAATCCACGATATGGCCCAAAAGGTTCGGGATCTCGGGGCGAAGGCGTTTTC
TTACAATTGGGTTGACGATTTCGGAAGAGGCAGGGATATTGTCCATCCAACGAAAGACGCCGAGCAGCACCGCAAGTTTATGGAATACGAGCAACATGTG
ATTGATGAGTTTAAAGATCTGATTCCGATTATTCCCTATGAGAGAAAACGCGCGGCAAATTGCGGCGCTGGCTGGAAGTCCATTGTGATCAGTCCGTTCG
GCGAAGTACGTCCTTGCGCCCTCTTTCCAAAGGAATTTTCATTGGGAAATATTTTTCATGATTCCTATGAAAGCATCTTTAACTCCCCTCTCGTCCATAA
ACTGTGGCAAGCGCAAGCGCCGCGGTTCAGCGAACATTGCATGAAAGACAAATGCCCGTTCAGCGGCTATTGCGGAGGCTGTTACTTAAAAGGGCTGAAC
TCTAACAAATATCACCGGAAAAACATTTGCTCTTGGGCGAAAAATGAACAATTAGAAGATGTGGTCCAGCTTATTTAGTAA

pEG7037 MdnC

AGACGCCCGCAACATTTTATTTTAAGGACGACCCAATGACCGTTTTAATTGTTACTTTTAGCCACGATAATGAAAGTATTCCTCTGGTAATCAAAGCCAT
AGAAGCCATGGGTAAAAAAGCCTTCCGTTTTGATACTGATCGCTTCCCTACAGAGGTGAAAGTTGATCTTTACTCAGGCGGTCAAAAAGGCGGAATTATT
ACCGATGGAGAACAAAAATTAGAGCTAAAAGAAGTTTCTTCTGTCTGGTATCGACGCATGAGATACGGACTAAAATTACCCGATGGGATGGATAGTCAAT
TTCGCGAAGCTTCTCTTAAGGAATGTCGGTTAAGTATTCGAGGAATGATTGCTAGTTTATCTGGCTTTCATCTTGATCCAATTGCTAAGGTAGATCATGC
TAATCATAAACAATTGCAGTTACAAGTGGCGCAACAATTAGGTTTATTAATTCCGGGGACTTTAACTTCTAATAATCCTGAAGCTGTCAAGCAATTTGCT
CGGGAGTTTGAAGCGACGGGAATTGTGACTAAAATGCTTTCTCAATTTGCTATTTATGGAGACAAGCAAGAGGAAATGGTTGTTTTTACCAGTCCTGTTA
CAAAGGAAGATCTAGATAATTTGGAAGGTTTGCAATTTTGTCCAATGACTTTTCAGGAAAACATTCCTAAAGCTTTGGAATTACGCATCACTATCGTCGG
TGAACAAATATTTACGGCGGCGATTAATTCCCAACAATTAGACGGTGCTATCTACGATTGGCGAAAAGAGGGACGCGCGCTCCATCAACAATGGCAACCC
TACGATTTACCGAAAACTATTGAAAAACAACTACTAGAATTAGTGAAATATTTCGGTCTTAATTATGGTGCAATTGATATGATTGTCACACCAGATGAAC
GTTATATCTTTTTAGAAATTAATCCCGTTGGCGAGTTTTTCTGGCTAGAACTTTATCCTCCTTATTTTCCTATCTCCCAGGCGATCGCTGAAATCCTAGT
TAACTCATAATAA

pEG7043 ProcM

AGAAATCACATTACGCATAGGGGGAGGTAGACACATGGAGAGTCCTAGCTCATGGAAAACATCGTGGCTGGCCGCCATCGCTCCGGATGAACCCCACAAA
TTCGACCGCCGCTTAGAATGGGACGAGCTTTCAGAGGAGAACTTCTTCGCAGCACTGAACTCAGAACCTGCATCGTTGGAAGAGGATGATCCATGTTTTG
AAGAAGCACTGCAAGACGCCCTGGAGGCCTTGAAGGCAGCATGGGATTTACCCCTTCTTCCCGTCGATAATAATCTTAATCGTCCCTTCGTAGATGTCTG
GTGGCCCATTCGCTGTCACTCTGCGGAGAGCTTGCGTCAAAGCTTCGTCAGTGATAGTGCTGGACTTGCGGACGAGATTTTTGATCAGCTGGCCGATTCG
TTACTGGACCGTCTGTGCGCCCTGGGAGATCAGGTGTTGTGGGAGGCGTTTAACAAGGAGCGTACACCAGGAACGATGTTGTTAGCCCACTTAGGAGCCG
CAGGCGACGGCTCCGGACCCCCTGTACGTGAGCATTACGAACGTTTTATTCAGTCTCACCGCCGTAATGGATTAGCGCCTTTGCTTAAGGAATTCCCTGT
ACTGGGCCGCCTTATTGGAACAGTTTTGTCCCTTTGGTTCCAAGGGAGCGTGGAAATGCTGCAACGTATCTGCGCTGACCGCACCGTTCTGCAACAGTGT
TTCGCTATCCCTTGCGGGCATCACCTGAAAACTGTAAAGCAGGGACTTTCTGATCCACACCGCGGCGGTCGCGCTGTGGCAGTTTTGGAATTTGCGGACC
CAAATTCCACCGCTAATTCAAGTATGCACGTAGTGTATAAACCGAAGGATATGGCTGTGGATGCAGCTTACCAGGCCACCTTAGCAGATCTTAATACTCA
TAGCGACCTTTCCCCGTTGCGCACGCTTGCCATTCATAACGGCAACGGATATGGTTACATGGAACATGTGGTTCACCATCTTTGCGCTAACGACAAAGAG
CTGACAAATTTCTATTTCAACGCTGGGCGTTTAACCGCGCTTCTGCATCTTCTTGGATGTACTGACTGTCACCATGAAAATTTGATTGCATGTGGTGATC
AATTACTGTTGATCGATACAGAAACATTATTGGAGGCGGATTTACCCGATCACATTTCGGATGCTTCGAGCACCACGGCGCAACCAAAGCCTAGTAGCCT
TCAAAAGCAATTTCAGCGTTCTGTTTTGCGTAGCGGGTTACTTCCTCAATGGATGTTCCTGGGGGAGTCGAAGTTGGCCATCGACATCTCGGCTCTGGGA
ATGTCCCCACCCAATAAGCCTGAGCGTATTGCACTTGGCTGGTTAGGATTCAATTCTGACGGGATGATGCCTGGGCGTGTATCCCAACCAGTTGAGATTC
CTACATCCTTGCCCGTTGGGATTGGTGAGGTTAATCCCTTTGATCGTTTTTAGAGGATTTTTGTGATGGCTTTTCCATGCAATCAGAGGCCCTTATTAA
GCTTCGCAACCGTTGGCTGGACGTTAATGGGGTTCTTGCTCATTTCGCGGGTCTGCCCCGCCGTATCGTTCTTCGCGCGACTCGCGTATACTTCACTATC
CAGCGTCAGCAGTTAGAGCCTACGGCACTGCGCTCTCCACTTGCACAGGCCTTGAAACTTGAGCAGCTTACTCGTTCTTTCTTGTTGGCAGAGTCAAAGC
CTCTTCACTGGCCCATTTTCGCAGCTGAAGTAAAGCAGATGCAGCACCTTGACATTCCTTTCTTCACACACTTAATCGACGCTGACGCTCTGCAGCTGGG
CGGCCTGGAACAAGAATTACCAGGCTTCATCCAGACTAGTGGCTTGGCAGCTGCTTACGAGCGTTTGCGTAATTTAGATACGGACGAGATTGCTTTCCAA
CTTCGTCTGATCCGCGGTGCAGTAGAGGCTCGCGAGTTGCATACTACGCCGGAGTCGAGCCCGACGTTGCCGCCGCCTGCCACCCCCGAGGCTCTTATGT
CCTCTTCAGCCGAGACTAGTTTAGAAGCTGCTAAGCGCATCGCTCACCGCTTACTGGAGTTGGCAATTCGTGATTCTCAAGGGCAAGTAGAATGGCTGGG
CATGGATCTGGGGGCAGATGGAGAGAGCTTCTCCTTTGGCCCAGTTGGCTTGAGCCTTTATGGGGGCTCAATCGGTATCGCTCACCTTCTGCAACGTTTG
CAGGCGCAGCAAGTTTCCTTGATGGACGCAGACGCTATCCAAACGGCAATTTTACAGCCCCTTGTGGGACTGGTTGATCAACCTAGCGACGACGGACGTC
GCCGTTGGTGGCGTGATCAGCCGCTGGGCTTAAGTGGATGTGGCGGTACCTTGCTTGCACTTACACTTCAAGGTGAACAAGCGATGGCTAATTCCCTGCT
GGCCGCTGCTTTGCCCCGTTTTATCGAGGCTGATCAGCAACTTGACCTGATTGGTGGCTGCGCTGGACTGATCGGTTCGTTGGTACAATTAGGTACTGAA
AGTGCCTTACAATTAGCTTTGCGTGCGGGCGACCATCTTATTGCGCAACAGAATGAAGAGGGGGCGTGGTCTAGCTCGTCATCACAGCCCGGTTTGTTGG
GCTTTAGTCATGGTACTGCAGGTTACGCAGCAGCCTTAGCACACTTACATGCATTTTCCGCTGATGAGCGTTACCGCACCGCAGCCGCTGCCGCTTTAGC
ATACGAACGCGCACGTTTTAATAAAGATGCCGGCAACTGGCCAGACTACCGCTCGATCGGACGTGACTCTGATTCAGATGAACCGTCCTTTATGGCTTCC
TGGTGTCACGGCGCACCCGGCATTGCCCTGGGCCGCGCCTGTTTGTGGGGTACGGCGCTTTGGGACGAAGAATGCACCAAGGAGATCGGAATTGGGTTAC
AGACCACAGCTGCTGTTTCGTCTGTTAGTACTGACCACCTGTGTTGTGGTTCACTTGGCCTTATGGTATTATTAGAGATGCTGTCAGCAGGACCCTGGCC
CATCGACAATCAATTACGTTCCCATTGCCAGGACGTAGCATTCCAGTACCGCCTGCAGGCTTTGCAGCGCTGTTCAGCCGAGCCGATTAAGCTTCGTTGC
TTCGGTACAAAAGAGGGCCTTTTAGTCCTGCCTGGATTTTTCACTGGCTTATCAGGAATGGGTTTAGCACTGCTTGAGGATGATCCATCTCGCGCCGTGG
TTTCTCAACTGATCAGTGCGGGCTTATGGCCGACAGAGTGATAA

pEG7047 MibHS

AGAGCACATCAGACCTAAGGAAAATATAAATGGCACGTAGTGAGGAATCGAACACTCTGGCACGTCTGTTTGACGTGTTGGGTGACGATGCCGCTGCCGC
ACGTGAATGGGTAACGGAACCCCATCGTCTGATCGCTAGCAATGAGCGCCTGGGCACAGCTCCGGAAGCCCCGGCGGATGACGATCCGGAGGCCATTCGG
ACGGTTGGAGTGATCGGAGGGGGCACAGCCGGGTATTTAACGGCGTTGGCTCTGAAGGCTAAACGCCCTTGGTTGGATGTGGCGCTCGTCGAAAGTGCGG
ATATCCCGATCATTGGGGTAGGAGAGGCGACGGTGTCTTATATGGTGATGTTTCTGCACCATTATCTGGGCATTGATCCGGCGGAGTTTTACCAACATGT
GCGCCCTACTTGGAAACTGGGCATCCGTTTTGAATGGGGGTCACGTCCGGAGGGCTTTGTTGCGCCATTCGATTGGGGGACCGGATCTGTTGGCCTGGTT
GGGAGCCTGCGTGAAACGGGCAATGTCAACGAAGCTACGTTACAGGCGATGCTCATGACGGAGGATCGCGTTCCGGTATATCGTGGCGAAGGTGGGCATG
TTAGTCTGATGAAATATCTGCCATTCGCATATCATATGGATAACGCTCGCCTGGTTCGCTACCTGACGGAACTCGCCACTCGTCGTGGCGTGCATCATGT
CGATGCGACTGTAGCTGAAGTTCGCCTGGATGGTCCTGACCACGTTGGGGACCTGATTACTACGGACGGTCGTCGCCTGCACTATGACTTTTACGTCGAT
TGTACTGGATTTCGTTCCCTGCTGCTGGAAAAAGCCCTGGGTATCCCGTTCGAATCTTATGCGTCAAGCCTGTTTACCGACGCGGCAATTACCGGTACCC
TTGCACATGGGGGTCATCTTAAACCTTACACTACGGCAACTACCATGAATGCGGGCTGGTGTTGGACGATCCCTACTCCTGAGTCCGATCACCTGGGGTA
CGTTTTCAGTAGTGCCGCGATCGATCCAGACGATGCAGCAGCAGAAATGGCCCGCCGTTTCCCGGGCGTTACCCGCGAAGCATTAGTTCGCTTTCGCTCC
GGCCGTCACCGTGAAGCTTGGCGCGGCAATGTCATCGCGGTAGGAAACAGCTATGCTTTCGTGGAACCTCTGGAGAGTTCGGGACTCCTGATGATTGCTA
CCGCAGTCCAGATCCTGGTGAGTTTGCTGCCGAGTAGTCGTCGTGACCCGCTGCCTAGCAATGTGGCGAATCAGGCGTTAGCTCACCGGTGGGACGCGAT
TCGTTGGTTTCTGAGTATTCATTACCGTTTCAACGGCCGCCTCGATACTCCGTTCTGGAAGGAAGCCCGTGCCGAAACAGATATTAGCGGTATTGAACCG
TTGCTTCGTCTGTTCAGTGCCGGTGCCCCTCTGACCGGTCGCGATAGCTTTGCGCGCTATTTGGCCGACGGAGCAGCCCCGTTGTTCTATGGCCTGGAGG
GTGTTGATACCTTACTGCTGGGACAGGAAGTGCCTGCGCGTCTGTTACCACCGCGTGAATCTCCTGAGCAGTGGCGTGCCCGTGCTGCAGCAGCCCGCTC
ATTAGCCTCGCGTGGCTTACGTCAGAGCGAAGCTCTGGATGCTTACGCTGCGGACCCCTGTCTCAATGCGGAACTGCTGTCTGATAGCGACTCATGGGCG
GGTGAACGCGTCGCGGTACGTGCAGGTCTGCGTTAATAAACCCTGCCATTTTTTTAGCCCAAAGAACACGGAGCATCTTTATGACGACTGGCACCACGGT
AGCGCATGCTGTAGAACCAGACGGTTTCCGCGCCGTGATGGCCACACTGCCGGCCGCTGTGGCGATCGTTACGGCAGCTGCGGCAGATGGGCGCCCGTGG
GGTATGACCTGCAGTTCGGTTTGCTCAGTGACCTTGACCCCGCCGACCCTTCTGGTCTGCCTTCGGACGGCGTCCCCGACTCTGGCCGCAGTCGTGTCAG
GTCGTGCATTTAGCGTGAACCTTCTGTGTGCGCGGGCCTATCCTGTGGCGGAATTGTTTGCATCTGCGGCAGCAGACCGGTTTGATCGCGTTCGTTGGCG
TCGCCCGCCGGGTACAGGCGGTCCACATCTTGCCGATGATGCACGTGCAGTGTTAGACTGTCGCCTGAGCGAAAGCGCAGAAGTAGGCGACCATGTGGTC
GTATTTGGCCAAGTCCGGGCGATTCGTCGCCTGAGTGATGAACCACCACTGATGTATGGTTATCGTCGTTACGCACCTTGGCCGGCAGATCGTGGTCCGG
GTGCGGCAGGCGGCTAATAA

pEG7048 MibD

AGATAACCCAATCCGTAAGGACACACGTCAAGGAGGCGATTTATGACGGCACACAGCGACGCAGGAGGTGACCCACGCCCGCCTGAACGCTTACTGTTGG
GGGTGTCAGGAAGTGTCGCTGCACTGAACTTACCGGCGTACATTTATGCCTTTCGGGCAGCCGGTGTGGCACGTCTTGCGGTCGTGCTGACACCAGCGGC
TGAAGGGTTCCTTCCAGCGGGTGCGTTACGCCCGATTGTGGATGCCGTTCATACGGAACATGACCAAGGCAAAGGTCACGTAGCGCTGTCACGCTGGGCG
CAACACTTACTCGTGCTGCCGGCAACAGCGAATTTGCTTGGCTGTGCAGCGTCAGGACTTGCGCCGAACTTTTTAGCGACCGTTCTGCTCGCGGCAGATT
GCCCAATCACATTCGTCCCGGCGATGAATCCGGTCATGTGGCGTAAACCAGCCGTACGCCGGAACGTTGCAACCTTACGCGCAGATGGTCATCACGTGGT
GGATCCTCTGCCGGGCGCTGTGTACGAAGCTGCCTCACGTTCTATCGTGGAAGGTCTTGCTATGCCGCGCCCTGAAGCGTTAGTCCGTTTACTGGGTGGC
GGTGATGACGGTTCTCCAGCAGGACCGGCAGGTCCGGTTGGACGCGCAGAGCATGTTGGGGCTGTTGAGGCTGTTGAAGCCGTGGAAGCAGTTGAGGCCG
TTGAGGCTGCGGAAGCACTTGCGTAATAA

pEG7056 PlpXY

AGAGCCACCATTTATAAGGAGAACCTACCGATGACCAAAAAGTATCGGCGTGTATCCTACGCAGTGTGGGAAATCACCCTGAAATGCAATCTGGCATGCT
CTCATTGTGGCAGCCGCGCCGGCCAAGCCCGTACGAAAGAGCTGAGTACCGAAGAAGCGTTCAACCTGGTCCGCCAGCTGGCCGACGTGGGCATTAAGGA
AGTCACCCTGATCGGTGGTGAAGCCTTTATGCGTTCGGATTGGCTGGAAATCGCGAAAGCCGTCACTGAAGCCGGCATGATCTGTGGCATGACCACAGGG
GGCTTCGGGGTCAGTCTGGAAACGGCGCGTAAAATGAAAGAAGCGGGCATTAAAACGGTGAGCGTTAGCATTGACGGTGGTATTCCTGAAACCCACGACC
GCCAGCGCGGTAAAAAGGGTGCGTGGCATAGTGCATTCCGGACTATGAGCCATCTGAAAGAAGTCGGGATCTACTTCGGTTGCAACACTCAAATCAATCG

TTTATCGGCGTCAGAATTCCCGATTATCTATGAACGTATTCGCGATGCTGGGGCACGTGCGTGGCAAATTCAGCTGACGGTTCCGATGGGCAACGCCGCG
GATAACGCAGATATGCTGCTGCAACCGTATGAATTGCTCGACATCTATCCGATGTTAGCCCGCGTTGCCAAACGTGCGAAACAGGAAGGCGTGCGTATTC
AGGCAGGTAACAACATCGGGTACTATGGACCGTATGAGCGTCTGCTGCGTGGCAGCGACGAATGGACGTTTTGGCAAGGATGTGGTGCGGGCCTTAACAC
CCTCGGCATCGAAGCCGACGGCAAAATCAAAGGCTGTCCATCCCTGCCGACCGCCGCGTACACCGGCGGTAACATTCGCGATCGCCCGCTGCGGGAAATC
GTCGAACAGACCGAAGAACTGAAATTTAACTTAAAAGCTGGTACAGAACAAGGTACGGACCATATGTGGGGCTTTTGTAAAACCTGCGAATTCGCGGAAC
TCTGTCGCGGCGGATGCAGCTGGACTGCGCATGTGTTCTTTGACCGGCGCGGCAATAATCCGTACTGCCACCATCGGGCTCTGAAACAAGCCCAAAAAGA
CATTCGCGAACGCTTTTATTTAAAAGTGAAAGCAAAGGGCAACCCGTTCGACAATGGTGAATTTGTTATCATTGAAGAACCTTTTAACGCTCCGTTACCC
GAGAATGACCTGCTGCACTTTAACAGTGATCACATTCAATGGCCAGAAAACTGGCAAAATAGTGAAAGCGCGTACGCATTGGCCAAGTAATAAATATAAA
GTTAAGGAGTTGCACATGAACAGTAATCAGATCCCTAACAAAGTTGCAACCGCGGCACAGAAATCTGACGACAGCAGCAGCGTATTACCGCGCCAGGGGT
GGCAAGACAAACAAGCCTTTATTAAGGCACTCATTAAAGCCAAACAGTCTCTCGAAATTGCCGAAATTAGCAACTTTTTAACC

pEG7058 PbtO

AGACGTCACTATCAAACACACTAATACCACATAAGGAGCGAACAATGACCCAGTATCCCCTGTCGCGTCCAGAACCGCTGGGCGTGCACCCAGATTATCG
TCGCCTGCGTGAGACTTGCCCGGTTGCACGTGTGGGTAGCCCGTATGGCCCAGCGTGGCTTGTCACCCGTTACGCCGATGTGGCCGCAGTTCTGACCGAT
GCCCGTTTTAGTCGTGCAGCCGCTCCGGAAGATGATGGTGGCATCCTGCTGAACACCGATCCGCCGGAACATGATCGTCTGCGTAAACTGATTGTAGCAC
ACACAGGCACCGCTCGCGTGGAACGGCTGCGTCCGCGTGCTGAAGAGATCGCTGTTGCGTTAGCGCGCCGTATCCCGGGCGAAGGCGAATTCATTAGTGC
ATTTGCCGAGCCCTTCAGCCATCGCGTTTTGTCTTTATTTGTTGGCCATCTTGTTGGGTTACCAGCGCAGGACCTGGGCCCCTTAGCGACCGTAGTGACT
CTGGCACCCGTTCCCGACCGCGAACGTGGCGCGGCATTTGCAGAGCTGTGTCGTCGGCTGGGTCGTCAGGTGGATCGCGAAACGCTTGCAGTAGTTTTAA
ACGTGGTCTTTGGCGGACATGCGGCTGTAGTGGCCGCGCTGGGTTATTGCCTGTTAGCTGCATTAGATGCGCCACTGCCACGTCTGGCCGGTGACCCAGA
GGGCATTGCCGAACTGGTGGAAGAAACCCTTCGTTTGGCTCCACCGGGAGATCGTACACTGTTGCGTCGTACTACAGAACCTGTGGAACTTGGCGGTCGC
ACATTACCAGCGGGTGCGCTTGTAATCCCGTCCATTGCAGCCGCAAACCGTGATCCGGATCGCCCTGTGGGCCGTCGTATGCCACGTCATCTTGCATTTG
GACGTGGAGCGCATGCCTGTTTAGGCATGGCGCTGGCGCGCATGGAACTCCAGGCAGCACTGAAAGCGTTAGCGGAACACGCGCCAGACGTACGGTTGCC
GGCTGGTACAGGCGCGCTGGTCCGCACACACGAAGAACTCTCGGTGAGCCCGCTCGCAGGAATCCCAATTCAACGCTAATAA

pEG7059 PbtM1

AGAGGAACGGATAAGGAGGTCAATATATGCTGTCTAGCGCGCTGGAGGTGGATATCGATGAAGCTGCGGTGGCGGCGGACTTACGCGAATTGGCCGCAGC
TCTGGATCGCAGTGGTTATGGTGAAATCCTCACCTGTTTTCTGCCTCAGAAGGCACAGGCGCATATCTGGGCTCAGACCGCTGCAAAAATTGATGGGCCG
TTGCGTACCCTGATGGAATTATTCCTTCTGGGTCGGGCGGTTCCCCAGGATGATCTCCCGCCTCGCATCGCGGCCGTGATTCCCGGTTTAGTTAGCGCAG
GTCTGGTTAAGACTGGACAGGGCGCGGTTTGGCTGCCGAACTTGATTCTGCTGCGTCCTATGGGCCAGTGGTTATGGTGTCAGCGGCCTCACCCCTCACC
GACCATGTACTTTGGTGACGATAGCCTGGCGCTGGTTCACCGGATGGTAACATATCGTGGCGGCCGTGCCCTGGATTTATGTGCAGGTCCGGGTGTTCAG
GCCCTTACCGCAGCCCTCCGCTCAGAGCACGTTACCGCGGTTGAGATCAATCCGGTCGCGGCAGCCCTTTGCCGCACCAACATTGCCATGAACGGTCTGT
CCGACCGCATGGAGGTTCGCCTGGGCTCACTGTACGACGTCGTGCGCGGTGAGGTTTTTGATGATATTGTATCAAACCCGCCGCTGCTGCCTGTTCCGGA
GGATGTGCAATTCGCCTTTGTGGGAGATGGCGGACGCGATGGTTTCGATATTTCTTGGACGATTCTGGATGGCCTGCCTGAACATCTGTCCGACCGTGGT
GCGTGTCGCATCGTTGGTTGTGTTCTGTCCGATGGCTATGTGCCTGTTGTGATGGAAGGCTTGGGAGAATGGGCCGCTAAACACGATTTCGACGTGCTTC
TTACAGTGACCGCACATGTCGAGGCGCATAAAGATAGTAGTTTTCTGCGTTCAATGAGCCTGATGAGTTCGGCGATCTCAGGCCGCCCAGCGGAGGAGCT
GCAAGAACGGTACGCAGCTGATTATGCCGAACTGGGCGGTTCCCACGTTGCGTTCTATGAACTGTGTGCCCGCCGTGGTGGGGGTTCTGCACGTCTGGCC
GACGTGAGCGCTACAAAACGCAGTGCGGAAGTGTGGTTTGTTTAATAA

pEG7060 PaaA

AGATCATTTCCAATAAGGGGGACACTATGAGCCTGACGAATGTCAAGCCGTTGATTAAAGAATCCCACCACATCATTTTAGCTGACGATGGTGACATTTG
CATTGGGGAAATTCCGGGGGTGTCTCAGGTAATCAATGACCCGCCGTCGTGGGTTCGTCCTGCCCTGGCAAAGATGGATGGCAAGCGTACTGTCCCCCGT
ATTTTCAAAGAACTGGTCAGTGAAGGCGTACAGATCGAATCCGAACATCTGGAAGGCCTGGTAGCCGGGCTTGCCGAACGCAAACTTCTCCAGGATAACA
GTTTCTTTTCCAAGGTGTTAAGCGGTGAAGAAGTGGAGCGCTATAACCGCCAGATTCTGCAGTTCAGCCTTATCGATGCGGATAACCAGCACCCTTTCGT
TTACCAAGAGCGGCTGAAACAGTCTAAAGTCGCTATCTTCGGTATGGGTGGCTGGGGCACGTGGTGTGCATTGCAGCTGGCCATGTCAGGCATTGGTACA
CTGCGGCTGATCGACGGCGATGATGTGGAACTGTCGAACATTAACCGCCAAGTTCTGTATCGCACGGATGATGTAGGTAAAAACAAAGTTGATGCCGCCA
AAGACACTATCCTGGCATACAACGAAAACGTGCATGTTGAAACCTTCTTTGAATTCGCCAGCCCGGACCGTGCCCGGCTTGAAGAACTTGTGGGTGATTC
TACCTTTATTATCCTGGCTTGGGCCGCGTTGGGTTACTACCGTAAAGATACGGCAGAGGAAATTATCCATTCGATTGCGAAAGATAAAGCGATCCCTGTA
ATTGAACTCGGCGGTGATCCTTTGGAAATCTCTGTCGGTCCTATTTACCTGAATGATGGCGTACACAGCGGCTTCGACGAGGTGAAAAATTCCGTTAAAG
ATAAATACTACGACAGCAACAGCGATATCCGCAAATTTCAAGAGGCGCGGTTGAAACACAGCTTCATCGATGGCGATCGTAAAGTGAACGCGTGGCAATC
AGCGCCCAGCCTGAGTATTATGGCTGGTATCGTAACGGATCAGGTTGTGAAAACCATTACCGGGTACGACAAGCCACATCTCGTTGGCAAGAAATTTATC
TTGAGTCTGCAAGATTTCCGCAGCCGCGAGGAGGAGATCTTTAAATAATAA

pEG7066 CinX

AGAAATTTTTCATACCGAGGGAGGAAAATATGGCTCTCAAAACCTGCGAAGAATTTCTGCGCGATGCGTTAGATCCGGATCGCTTCGGCCGCGAGATGAA
GGCAGTAACAGAAATTCCCGAGATCGTTAAACTCGGCCATCGTCATGGTTATGGATTTACTGCCGAAGAATTTCTGACCAAAGCTATGAGTTTTGGTGCT
CCGCCGGCAGGAGCAGCAGCACCTGGCGAATCAGCCAGCGTTCCTGGCCAGAACGGTTCCTCCCCCGGACACGCTGCGCGTGCAGCTATGGCTGGTCCAG
AAGCAGGGGCCACCAGCTTTGCCCACTATGAATACCGTCTGGATGAGCTGCCGGAATTCGCCCCCGTTGTGGCCGAGCTTCCGAAACTGAAAGTCATGCC
GCCTTCCGTGGGACCTGATCGGTTTGCAGCACGCTACCGTGATGAAGATATGCGCACAATTTCAATGAGTCCGGCGGATCCGGCTTACCAGGCTTGGCAC
CAGGAACTGGCGGGTCGTGGTTGGCGCGATGCAGAAGATACGGCTGCTGCTCCAGATGCCCCACGGCGCGATTTTCATCTGCTGAACCTCGATGAGCATG
TAGATTACCCAGGTTATGAAGAATATTTTGCGGCCAAGACCCGTGTCGTCGCGGCACTCGAAAACCTGTTTGGTGGTGACGTGCGTTGCTCAGGCTCTAT
GTGGTATCCGCCGTCGAGCTATCGCTTATGGCATACAAATGCCGATCAACCGGGGTGGCGTATGTACCTGGTAGATGTAGATCGCCCATTCGCGGACCCC
GACCGTACCTCCTTCTTTCGCTACCTGCATCCACGTACCCGTGAAATCGTCACGCTGCGCGAAAGCCCTCGTATTGTCCGTTTCTTTAAAGTCGAACAGG
ATCCCGAGAAGCTGTTCTGGCACTGTATCGCGAACCCCACCGATCGCCATCGCTGGTCGTTTGGTTACGTTGTTCCGGAAAACTGGATGGACGCCCTCCG
TCACCATGGCTAATAA

pEG7067 CapBC

AGAGGCCTACAGATATTCCAGACTAACACTAAGGAGGAAAACGATGCAGCCAGACCTGGAGGTTGTTGATGTTCGTCGCGGCGAGTCGTTCAAGGCATGG
TCGCATGGGTACCCATATCGCACTGTTCGCTGGCACTTCCATCCTGAGTTTGAAGTACATCTGATCGTGGAAACCACCGGCCAGATGTTTGTGGGTGATT
ATGTCGGAGGCTTTGGTCCGGGTAATCTGGTCCTGATGGGTCCCAATCTGCCTCATAATTGGGTGTCTGACGTTCCTGAGGGTAAAACCGTTGCAGAGCG
TAACCTTGTTGTTCAATTTGGGCAAGCGTTCGTTTCCCGTTGCGAGGATTCCTTAACGGAGTGGCGTCACGTGGAAACGTTACTGGCGGATGCGCGGCGT
GGCGTGCAATTTGGGCCGCGCACCTCTGAGGCCATTAAACCTCTGTTCGCGGAACTGATTCACGCGCGCGGCCTGCGTCGCATTGTGCTGTTTCTGTCTA
TGCTGCAAATCCTCGTCGATGCAACGGATCGCGAACTGCTGGCATCTCCAGCTTATCAGGCGGATCCTTCGACATTTGCAAGCACGCGCATTAATCATGC
GCTGGCCTACATTGGAAAGAATCTGGCGAACGAGCTTCGTGAAACAGATTTAGCACGGCTGGCCGGACAGTCTGTTTCCGCCTTCTCTCATTATTTTCGT
CGTCATACCGGCCTGCCTTTCGTGCAGTACGTTAATCGCATGCGTATCAACCTGGCCTGTCAGCTTCTGATGGACGGGGACGCATCGGTGACAGATATTT
GTTTCCGTAGCGGTTTTAACAACCTGTCCAATTTTAACCGTCAGTTTCTGGCAGTGAAAGGTATGTCACCCAGTCGGTTCCGTCGCTACCAGGCTCTCAA
CGACGCGTCACGTGATGCGAGTGAAGCGGCTGCAAAACGCGGCGCAGGTATTGCAGGTGCACCGGCAATCGTTCCAGCGGCTCAAGCACGTGGCGAGGCA
CGCCCAATTCCTGAAGTGCTGCTTAGCGGCTAATAATGGCTTCCGTTTTTCACCACTTGTTAAGGAGTACTTTATGATGCTGACGGCGAGCTCCACACCG
GCATCCGGTAATCCAGCTGCCCGTGCATTGCGCGCCGCTGCCTTTGCACTGGCCTTAGGCGGAGCATGCGTTGCGCATGCCGCACCTCTGCGGATTGGCA
TGACATTCCAAGAATTGAATAACCCGTATTTTGTGACCATGCAGAAAGCACTGAACGAAGCCGCGGCGAGCATTGGCGCGCAAGTGATTGTAACAGACGC
ACATCACGACGTGTCAAAACAGGTATCAGACGTTGAGGATATGCTGCAGAAGAAAATTGATATTTTACTGGTGAATCCAACCGACTCCACGGGCATCCAG
AGTGCGATTGTTTCCGCAAAGAAGGCTGGCGCCGTGGTCGTGGCGGTCGATGCCAATGCCAATGGCCCGGTGGATTCCTTCGTAGGGTCCAAGAATTTTG
ATGCCGGCGCTATGTCATGCGAGTACCTTGCGAAAGCGATCAACGGCGGCGGCGAAGTGGCCATTCTGGATGGCATCCCGGTCGTCCCAATCCTGGAACG
TGTCCGCGGCTGCCGCGCGGCACTGGCCAAATTCCCGAATGTGAAAATTGTCGACGTTCAGAATGGAAAACAGGAACGTGCGACAGCGTTAACGGTAACC
GAGAATATGATCCAGGCGCACCCGAAACTGAAAGGTGTGTTTAGTGTAAACGACGGCGGGTCAATGGGCGCTTTGAGCGCCATTGAAGCGAGCGGCAAAG
ATATCCGCCTCACGTCCGTAGATGGTGCCCCAGAGGCGGTGGCGGCGATTCAAAAGCCGAACTCCAAATTTATTGAAACAAGCGCTCAATTTCCGCGCGA
CCAGATCCGTTTAGCGATTGGTATTGGCCTGGCCAAGAAATGGGGCGCGAACGTGCCAAAAGCGATTCCAGTCGACGTGAAACTGATTGACAAAGGGAAC
GCGAAAACCTTTAGTTGGTAATAA

pEG7068 LasBCD

AGACAATTCATAAGGAGGTTAAGGTATGAAAGGCGAGGAAATGTTGGGACATCCACAGACCGGTTTTGTTGTACTGCCAGACAACGATGCCACCGGCGAC
GTGACGGGCCGCCTGTTACCTTGGGGTGATGTAGTTACAGTGTATCCGTCTGGCCGTCCATGGATCATCGGCAACTGCTGGGATCGCCCAGTCCTCGTCC
ATGATGGCGTGATCGTCTTGGGTCATACCAGCGTCACGCGTGATCAAATTGCCCGTCATGGGAACGATCCGCATCGCTTACTGGACGAGGCCGACGGCGC
ATTTCATGCGGCGGTCCTGATCGGACACGAAGTTCATGTTCGCGGCTCCGCCTACGGTGTCTGTCGTCTGTATACATGCGTTGTTGACGGTGTGACCTTA
GTGAGTGATCGTACAGACGTCCTGCAGCGTCTGGCAGGTACTGATGTGGACGTCGACGTGCTGGCTGGCCACTTGTTAGAGCCGATCCCGCACTGGTTAG
GCGAACAACCGTTATTGACGTCCGTGGAGCCCGTGCCACCGACACATCACGTTATTTTAACTCCGGACGCACGTAGTCGTTTACGGCCATCACGTCGTCG
TCGGCCTGAACCGTCGCTGGGTTTGCGGGACGGTGCGGAACTTGTCCGGGAGCGTCTGGCCGCAGCTGTGGCTACCCGTGTGGACAGTCCAGCGTTAATT
ACCAGTGAACTGAGTGGCGGCTATGATTCCACTAGTGTGTCATACTTGGCAGCGCGCGGTAAAGCCGAGGTGGTGCTGGTCACGGCCGCGGACGTGACA
GCACAAGCGAGGATCTGTGGTGGGCTGAACGCGCAGCCGCAGGGCTCCCGGAACTCGATCACGTAGTGTTACCTGCGGATGAATTACCGTTTACGTACGC
CGGCCTGACGGAGCCTGGTGCACTTTTGGATGAACCGTGTACGGCTGTTGCCGGCCGTGAGCGTGTACTGGCGCTGGTACGTAAAGCCGCGGCCCGCGGC
TCTACACTTCATCTGACTGGCCATGGTGGCGATCACCTGTTTACTTCACTGCCGACACCGTTTCATGACCTGTTTCGTACGCGTCCAGTCGCCGCGCTCC
GCCAGTTGCGTGCATTTGGCGCGTTGGCTGCGTGGCCGACCCGTAAGCTGATGCGCGAACTCGCGGACCGCCGCGATCATAGCACCTGGTGGCGCGCGCA
CGCACGTCCTCAGAATGGCCAGCCGGATCCGCACAGCCCCATGTTAGGCTGGGCAATTCCCCCGACTGTCCCGGCGTGGGTTACTGCTGACGGCGTGCGC
GCGATCGAACTTGGGATTTTAGAAATGGCAGAACGCGCGGAGCCCCTTGGTCATGCGCGCGGAGAACACGCTGAGCTGGATTCAATCTTTGAAGGGGCGC
GTATGGCCCGTGGCCTCAATCGTATGGCTACGCATGCCGGAGTCCCGCTTGCAGCCCCGTTCCATGACGATCGGGTCGTGGAAGCGTGTCTGTCGATCCG

GCCGGAGGAACGCATTTCTGCATGGCAGTACAAACCCTTACTGAACGCCGCAATGCAGGGTGTGGTGCCGAGCACCGTTCTTGATCGTAGCGCTAAAGAT
GACGGGAGTATTGATGTGGCCTATGGGCTGCAGGAACACCGTGATGAACTGGTAGCGCTGTGGGAATCATCACGTCTGGCGGAAACCGGTCTGATTGATG
CGGGTATGCTGCGGCGTTTATGCGCGCAGCCGTCCTCCCACGAGCTCGAGCATGGATCCTTGTACGCTACTATCGCTTGTGAGTTGTGGCTGCGTGGTTT
AGATCAGGATCGTACCCAACGCTACTAATAACCTACTACTCTGATCCCCATAAGGAGGTTTTTTATGCCGGTGCAGCTGCGTCGGCATGTGTCTTTTACG
GCTACGGAATACGGCGGCGTGCTGCTGGATGAAACCAAAGGCGCATACTGGCGTCTGAACACCACAGGCGCCGAAGTTGTTCGCGCCATGGGGGAAGCCG
AGCGGGATGAGATTGTACGGCATGTGGTGGCGACCTTCGATGTTGATGCGCAAACCGCAGCCCAGGATGTCGATGTCCTGCTGGCAGAACTTCGTGATGC
CGGCCTTGTGGCCTCGTAATAACAACCTAATCTTAGGCGAGGTCATTTTTTATGTCTGTGAATATGGCTCTCCGTGGCCATGGTATGTCCGGTCGCCGTC
GTCGCTTAGATGCCACGCGTGCTCGCCTGGCCGTTGTGGTTGCCCGTGTCCTGAATCTCTTACCGCCGCGCTTAATCCGTCGTTGTTTGCGTGTACTGAG
TCGCGGAGCCCGCCCTGCCTCGATTGAGGCAGCAGAAGCTGCTCGTCGTACTGTGGTTGCGGTGAGTCCAGCTGCCGCCGGTGCGTACGGCTGTTTAATC
CGCAGCATTGCCACCACCCTGGTTCTTCGTTCACGCGGGCAATGGCCAACCTGGTGTTGGTGTACGTGCGGAGCCTCCTTTTGGTGCCCATGCCTGGA
TTGAAGCAGAGGAGCGGCTGGTGGATGAACCTGGTACTATGCATACTTACCGTCGTCTTATCACCGTTGGTCCACTGTCTCGCAAAGTTCGTTAATAA

pEG7069 LasF

AGAGCCATCAGATTTAAGGAACATAAAAAATGTCTATCGAACTGACGCCTAGTTTGGCCGATCTGGTCGATCCACTTCCAGGTCACGCACTGCGCGCTGC
GGCGACATTACGTCTGGCAGATCTGATTGCGGCTGGTGCAGATACTGCACCGGCATTAGCAGCGGCGGCACGCATTGATGCTGACGCGATCGCGCGTCTT
ATGCGGTATCTGTGCAGTCGCGGGATTTTTCAAGCACATGAAGGCCGGTACGCGTTGACTGAATTTAGCGAATTGCTGCTGGATGAAGATCCATCTGGCC
TGCGTAAAACCTTAGATCAGGATAGCTATGGGGATCGTTTCGACCGCGCGGTTGCGGAACTGGTGGACGTTGTACGGTCCGGTGAACCTTCTTATCCTCG
CCTTTACGGCTCGACGGTTTATGATGACCTGGCAGCCGATCGTCCCTCGGCGAGGTGTTCGCGGATGTTCGTGGCTTGCACTCCGCAGGGTATGGGGAA
GATGTCGCGGCAGTGGCGGGTTGGTCCTCATGCCTGCGCGTTGTCGATCTGGGTGGAGGGACTGGCTCCGTCCTGCTTGCTGTGTTAGAGCGTCACCCGT
CCCTGTCAGGCGCAGTACTGGATCTGCCATACGTCGCCCCGCAGGCAAAGAAAGCTCTGCAGGCCTCAGCGTTTGCCCAACGTTGTGAATTTATCAAAGG
GAGCTTCTTCGATCCGTTACCTCCGGCAGACCGTTACCTGTTGTGTAACGTGCTGTTCAACTGGGATGACGCGCAAGCAGGCGCTATTTTGGCACGCTGT
GCGCAGGCGGGCCCTGTGGCCGGAGTAGTGGTAGCCGAACGTTTGATCGATCCGGATGCGGAAGTGGAACTCGTAGCAGCTCAAGATCTGCGTCTGTTGG
CTGTTTGCGGCGGTCGGCAGCGTGGCACCGCTGAATTCGAAGCGCTTGGGGCAGCCCATGGCCTGGCGTTAACCAGCGTTACCCTCACGGCATCTGGTAT
GAGCCTGCTCCGTTTCGATGTGTGTCGTGCCGGGAGTGCTGGCGGGGAAGTTGTGGAAAAATCTTAATAA

pEG7070 AlbsBC

AGAGTTTAGGAGAAAGACATAAGGAAATATTAAATGCCTGAGCTTCCCCGTTTCGCGACGGCCCCTCGTCACGTGCGTGCCCTGGATTTCGGTCATGTTC
TGGTCCTGATCGATTACCGTTCCAATCACGTCCAGTGCCTGCTTCCGGCAGCCGCAGCCCATTGGACAGCCACAGCGCGTACCGGCCGCTTGGACACCAT
GCCGGCAGCGCTGGCCACCCAGTTACTGACATCGGCGTTATTAGTACCGCGGCCGACCGCAACACCGTGGACGGCACCTGTAGCGGCACCACCTGCTCCA
CCGTCATGGGGTGGATCCGAGCATCCTGCCGGGACATCACGCCCTCGGGCACGTCATCGGCACTCAACCACGGCTGCGGCGGCGCTGGCATGTGTGCTGG
CGATTAAGGCAGCAGGCCCAACCCGCTATGCTATGCAGCGCTTGACCACGGTCGTGAAGGCAGCCGCTTCTACGTGCCGTCGCCCGGCAACGCCAGCACA
AGCGACGGCTGCTGCGCTTGCGGTCCGTCAGGCGGCTGGTACTCGCCAGCGCGTACAGCCTGTCTGGAAGAATCCGCCGCGACTGTCATTTTACTCGCT
ACCCGGCGTTTGAGTTCGACATGGTGCCATGGAGTAGCTCCCGATCCGATTCGCCTCCATGCCTGGGTGGAAACTGAGGATGGGACACCTGTAGCAGAGC
CAGCCTCGACCCTTGCGTACACCCCGGCCTTAACCATTGGAGGCCACCATCAACACCAGCCTTAATAAGGAAGCAGCCGTAAAAGGTAGGTTTTTTTAT
GATCTTTGGTGGATTTTCGACGACCCGTGAAGTTCGTCAACGCCCTGGTAATGCCGAGTTTATTGCTACGGACTCGCCTATTTGGCGCCTCGGTCGTAGT
CCAGCTCGTTGCGTGGCTGCGGACCATGGACAGCGTCGCCTGGTAGTGTTGGGAGAATGCGGGGCAACGGATGGCGAATTATCTCGCCTGGCGACCGCGG
GGCTGCCCACGGATATTACCTGGCGCTGGCCAGGCGTGTACGTGGTGGTCGAAGAACAACCGGAACGTACGGTGCTGCACACTGATCCAGCAGCTGCACT
CCCGGTATACGCAACCCCTTGGCAAGGCGGCTGGGCATGGTCAACCAGCGCGCGCATCCTGGCACGTTTAACAGAAGCTCCAATTGATGGTCAACGCCTG
GCATGTTCAGTGCTGGCCCCGTCTGTTCCGGCTCTGAGCGGTACCCGCACATTCTTTGCGGGTATCGAACAATTGGCCCTGGGTTCGCGTATTGAACTGC
CGGTGGATGGGTCCCGTCTGCGTGTTACGGTACGTTGGCGCCCGGATCCAGTCCCGGGAGAACCATATCATCGCTTGCGCACAGCGTTGACCGAGGCGGT
CGCCCTGCGTGTCAACCGCGCACCAGACCTGTCATGCGACCTCTCGGGCGGCCTCGATTCCACGTCACTGGCGGCAGTCCTGGCGGCTGTGTGCTTACCGGAG
TCCCACCATCTGAATGCTATCACGATTCATCCGGAGGGCGATGAAAGTGGCGCGGACTTACGGTATGCGCGCTTGGCAGCTGCGCACCACGGGCGTATTC
GCCACCACCTTCTCCCCCTTGCGGCAGAACACCTGCCGTATACTGAAATTACGGCGGTGCCCCCTACCACCGAACCGGCACCTTCAACATTAACGCGTGC
ACGCCTCGCGTGGCAGTTAGATTGGATGCGCCAGCACTTAGGCAGCCGCACCCATATGACTGGCGATGGAGGCGACAGCGTACTGTTCCAACCGCCGGCA
CATCTGGCGGATCTCCTGCGGCATCGGCAGTGGCGTCGGACTTTGTCGGAAAGTTTGGGATGGGCACGCCTTCGCCATACGTCTGTTTTACCCTTACTGC
GTGGAGCAGCAACTCTTGCACGTACATCACGTCGGTCGGGCCTCCAGGATCTCGCACGCGCATTGGCGGGTGCAGGTCAGCAGGGCGATGGTCGTGGCAA
TGTGAGCTGGTTCGCACCATTACCGCTGCCTGGCTGGGCGACCCCAACCGCTCGTCGCTTACTGCTTGATGCAGCCGATGAAGCTATCTCGACCGCGGAT
CCGTTACCGGGACTGGATACGTCGCTGCGCGTACTGATCGATGAAATTCGCGAAGTCGCCCGCACGGCAGCGGCAGATGCCGAACTGGCGGATGCTCACG
GAACGACTCTGCATAACCCATTTCTCGATCCGCGCACTATTGATGCAGTCCTGCGCACGCCAATCGCACATCGCCCGGCGGTCCACTCGTATAAGCCAGC
GCTGGGGCATGCAATGCAGGATTTGCTCCCGGGTGCAGTCGCTCGGCGCTCAACTAAAGGCTCTTTTAACGCCGATCATTATGCGGGGATGCGTGCAAAT
CTGCCAGCATTGACAGCGCTGGCAGATGGCCACCTGGCCGACCTGGGTTTGTTGGAGCCGACGCGCTTCCGCAGTCATCTTCGCCAAGCCGCCGCGGGCA
TTCCGATGCCGCTTGCGGCGATCGAACAGGCGCTGTCTGCCGAAGCATGGTGTCATGCACATCACGCCACCCCAAGCCCTGCCTGGACAACGCAGCCACC
GGAACACCCGCATGCCTAATAA

pEG7071 AlbsT

AGACGCTTGAACCAGCAATAAGGAGAGTAATTATGAGCACGTCCCCCGAACAGACCCTCTGGATCTCAACTGATACCTGTGGTCTGGGGCCGTATCGCGC
TGACTTGGTGGATACCTATTGGCAGTGGGAACAAGACCCAACATTGCTTGTAGGCTACGGTCGTCAGTCACCGCAGTCACTGGAGGCCCGCACGGAAGGT
ATGGCCCACCAATTGCGTGGCGATAACATCCGTTTCACTATCTATGATCTGTGCAGCAGTACACCTACCCCGGCGGGCGTGGCAACGCTGCTGCCCGATC
ATAGCGTCCGTACTGCCGAGTATGTTATTATGCTTGCGCCTGAAGCACGTGGGCGTGGCTTAGGAACCACCGCCACGCAGCTGACGTTAGATTATGCGTT
TCACATCACCAATCTGCGGATGGTCTGGTTGAAAGTACTGGCGCCGAACACCGCGGGCATCCGTGCGTATGAGAAAGCTGGCTTTCGTACAGTTGGAGCG
CTTCGCGAAGCCGGCTATTGGCTGGGGAAGGTCTGCGATGAGGTACTGATGGATGCCTTAGCGAAAGACTTCACGGGTCCAAGTGCAGTCCACGCAGCAT
TAACTGGCGCCAGCGGTCGCCAGCTGCGCCGTGCACCTTAATAA

pEG7073 McbCD

AGAGCTTCACCCTACAAGGAGGATATAGAATGTCAAAACACGAACTCTCTTTAGTGGAAGTAACGCATTACACAGATCCTGAAGTTCTGGCCATTGTTAA
AGATTTTCATGTCAGAGGTAACTTTGCTTCCCTCCCCGAATTTGCTGAACGAACTTTCGTGTCCGCGGTACCTCTTGCCCATCTGGAGAAATTTGAAAAT
AAAGAAGTTCTCTTCAGGCCAGGTTTCAGCTCCGTAATAAACATATCCTCATCACATAATTTTAGTCGTGAAAGGCTCCCATCAGGAATAAACTTTTGCG
ACAAAAATAAACTTTCCATTCGTACTATTGAAAAGTTATTAGTCAATGCATTCAGCTCACCTGATCCTGGCTCTGTAAGGCGGCCTTATCCTTCTGGGGG
GGCATTGTACCCGATTGAAGTTTTTTTATGCAGATTATCTGAAAATACAGAAAACTGGCAGGCAGGAACTAATGTTTATCACTACCTGCCGCTAAGTCAG
GCACTAGAACCTGTTGCTACATGTAATACTCAGTCACTCTACCGAAGCCTGTCCGGTGGGGATTCGGAACGTCTTGGTAAACCCCATTTTGCTCTCGTCT
ATTGCATTATTTTTGAAAAAGCTTTGTTCAAATATCGCTACAGGATATACCGGATGGCCTTAATGGAAACAGGTTCGATGTATCAGAACGCAGTATTGGT
TGCAGATCAAATAGGACTGAAAAACCGGGTATGGGCGGGATATACCGATTCATACGTAGCAAAAACAATGAATCTGGATCAGAGGACTGTAGCGCCACTG
ATCGTTCAGTTTTTTGGAGATGTAAACGATGATAAATGTCTACAGTAACCTTATGTCCGCATGGCCGGCCACAATGGCCATGAGTCCAAAACTGAACAGA
AATATGCCAACGTTTTCTCAGATATGGGACTATGAGCGTATTACACCAGCCAGCGCGGCCAGCTGGTGAAACTCTGAAGTCAATTCAGGGGGCAATAGGTGAAT
ATTTTGAACGCCGTCATTTTTTTAATGAGATAGTCACCGGTGGTCAGAAAACATTATATGAGATGATGCCTCCATCTGCTGCAAAGGCTTTTACCGAAGC
ATTTTTTCAGATCTCATCACTGACCCGCGATGAAATCATAACCCATAAATTTAAAACGGTCAGAGCCTTTAATCTGTTTAGCCTTGAACAACAAGAAATA
CCTGCAGTCATAATTGCACTCGACAATATAACCGCTGCAGATGATCTGAAATTTTATCCTGACAGAGATACATGCGGATGTAGCTTTCATGGTAGTTTGA
ACGATGCCATAGAAGGTTCCTTGTGTGAATTTATGGAGAGACAGTCCCTCCTTCTTTACTGGTTACAGGGAAAAGCCAATACTGAAATATCCAGTGAAAT
AGTAACAGGCATAAATCATATAGATGAGATTTTACTGGCTCTCAGGTCAGAAGGAGATATCAGGATTTTCGATATCACCCTGCCCGGAGCTCCTGGACAC
GCAGTACTAACCCTGTATGGCACAAAAAACAAAATCAGTCGAATAAAATACAGTACCGGATTATCCTATGCTAATAGTCTGAAAAAAGCACTTTGTAAAT
CCGTAGTGGAATTGTGGCAATCGTATATATGCCTGCACAACTTTCTTATTGGCGGTTATACTGATGATGACATTATTGATAGTTACCAGCGTCACTTTAT
GTCATGCAACAAGTACGAGTCGTTTACGGATTTGTGTGAAAATACGGTACTACTGTCTGATGATGTCAAGTTAACGTTTGAGGAAAATATTACGTCAGAC
ACAAATTTATTAAACTATCTTCAACAAATTTCTGATAATATTTTTGTTTACTATGCCAGGGAAAGAGTAAGTAACAGCCTTGTCTGGTACACAAAAAATAG
TAAGCCCTGATTTTTTCCTTCATATGAATAACTCAGGTGCAATAAACATTAATAATAAAATTTACCATACCGGGGACGGTATTAAAGTCAGAGAATCAAA
GATGGTACCATTCCCATAATAA

pEG7074 MibO

AGAGTTCATCAGTTTATTAGGAAAATATGATTTTTGGCCCGGATTTTCATCGCGATCCGTATCCAGTGTATCGTCGTCTGCGTGATGAGGCTCCGTGCCA
CCATGAACCAGCGTTAGGTCTGTATGCGTTGAGCCGCTACGAGGACGTTCTGGCTGCCCTTCGTCAGCCCACCGTGTTCAGCTCAGCAGCGCGTGCGGTA
GCCTCCAGTGCAGCGGGAGCAGGTCCATACCGCGGTGCCGACACCGTTAGTCCGGAGCGGGAAACTGCGGCTGAAGGGCCCGCCCGTAGCCTGTTGTTCC
TGGATCCGCCAGAGCACCAGGTGCTGCGTCAGGCGGTGTCCCGTGGCTTTACGCCGCAGGCAGTATTGCGCCTTGAGCCGGCCGTCCGCGACATTGCGGC
GGGTCTTGCTGATCGTATCCCCGATCGCGGTGGTGGCGAGTTCGTTACCGAATTTGCGGCTCCGCTGGCAATCGCGGTGATTCTGCGGTTACTTGGTGTA
CCGGAAGCAGATCGTGCCCGCGTAAGCGAACTTTTATCGGCATCAGCCCTGTCGGGGGCGGAAGCAGAACTGCGCTCCTATTGGCTGGGCCTTTCGGCAC
TCCTCCGCGATCGTGAAGATGCAGGCGAAGGTGACGGAGAGGATCGTGGTGTGGTGGCGGCTCTGGTCCGTCCTGATGCTGGACTGCGCGACGCGGATGT
TGCCGCAGGACCTGCCGTGCGTGCACCGCTGACGGATGAGCAGGTTGCAGCATTCTGCGCCTTAGTGGCAATCGCGGCAATCGGTGGGGCAAGCCGGCACTGAAAGTGTGGCAATGGCG
CTCTCCAACGCATTGGTCCTGTTCGGGCGTCACCATGACCAGTGGCGCACACTGTGTGCGCGTCCGGATGCGATTCCAGCAGCATTCGAAGAGGTCCTCC
GCTATTGGGCACCTACGCAGCATCAAGGTCGGACGTTAACCGCGGCGGTACGTTTACATGGCCGTCTGCTGCCGGCCGGTGCGCATGTACTGCTGCTGAC
CGGTTCAGCCGGCCGGGATGAACGTGCGTACCCAGACCCCGATGTATTTGACATCGGTCGCTTCCACCCGGATCGTCGTCCGTCGACCGCGCTGGGTTTT
GGTCTGGGCGCACACTTTTGTTTAGGCGCTGCTCTCGCTCGTCTGCAGGCACGCGTAGCGCTGCGCGAACTGACACGCCGGTTCCCGCGTTATCGTACGG
ACGAGGAACGCACTGTGCGTTCGGAAGTGATGAACGGGTTCGGCCACAGCCGTGTACCATTTTCCACGTAATAA

pEG7076 LtnM1

AGACAATTGAAGCAGGCTAGCCAGGAGTTCCATATGAAGTTTAACAAGAACGTGTTCCCAGAGATCAATGAAACGGATTTCGATAACAATATCAAGCCCC
TGCTGGATGAACTGGAATCTCGTATTACCATTCCGCAGGAGGAACTGAGCTTTTCAAGCATTAACGATGATTTATTTCGCGAGTTAACCCGCAACGAGGA

GTACCCTTACCAGAGCATTTGTACGATCGTTGCAAACATCGTGATGGATGACGGCAGTGAGATTTGGCGCAAAGATATTTTTGTTGATTCCAATAGTGTG
CGCGAAGCCGTATGCGACATTCTGAGCCAAACGTTATTCCTCTATTTCATCCGCTGCTTCTCCGAACAAATTAAAGACATTCGCAAAACTGATGAGGATA
AAGAGTCCACCTACAACCGCTACATTAACCTCCTGTTCAGCTCCAACTTCAAAATCTTCTCCGACGAATACCCTGTCCTGTGGTATCGGACCATTCGCAT
CATCAAAAATCGCTGGTATTCTATCAAGAAATCGTTACTGCTGACTCAAAAACACCGTGTGGAGATCGATAAGCAGTTGGACATCCCGCACAAGATGAAG
ATTAAAGGCCTGAAAATCGGGGGAGACACGCATAACGGCGGTGCCACAGTGACCACGATCTTCTTTGAGAAAGGGTATAAACTGATTTATAAGCCGCGGA
GCACATCCGGCGAATTCTCGTACAAGAAATTTATCGAAAAGATTAACCCGTACCTGAAGAAAGACATGGGAGCGATTAAAGCGATCGATTTCGGTGAATA
CGGCTTTTCTGAGTATATTGAGTGTAACACGGATGAAGAGGACATGAAACAGGTCGGTCAGCTTGCATTTTTCATGTACCTGTTGAATGCATCAGATATG
CATTATAGCAATGTCATTTGGACCAAACAGGGCCCTGTGCCGATTGATTTAGAAACCTTGTTCCAGCCGGATCGTATTCGCAAAGGCCTGAAGCAGTCGG
AAACTAACGCGTACCACAAAATGGAGAAAAGTGTATACGGAACGGGAATTATTCCAATTTCCCTGAGCGTTAAAGGCAAAAAGGGTGAGGTCGACGTCGG
CTTTAGTGGAATCCGTGATGAGCGCTCTAGTTCGCCGTTTCGCGTTCTGGAAATTTTGGATGGGTTTTCGAGCGACATCAAAATCGTGTGGAAAAAGCAG
CAGAAGTCTAGCTCCAGCAAAAACAATCTGATTGTCGATCACAAAAAGGAGCGCGAAATCCTTCAGCGTGCCCAGTCCGTCGTAGAAGGTTTCCAGGAAA
CCTCTAAAATCTTCATGAAACATCGTGAGGAATTCATCTCCATTATCTTAGACTCATTCGAGAACATCAAAATTCGCTACATCCATAACATGACGTTTCG
CTACGAACAGTTGCTGCGCACTCTGACGGATGCCGAGCCGGCCCAGAAGATTGAGTTAGACCGTCTGCTGCTGAGTCGTACCGGAATTCTGTCCATCTCG
TCTAGTCCCTACATCTCGCTCTCCGAATGTCAACAGATGTGGCAGGGTGACGTGCCGTACTTCTACTCGAAGTTTTCGAGCAAAAGTATCTTTGATACCA
ATGGCTTCGTTGATGAAATCGAGCTGACGCCCCGCCAGGCATTTATCATCAAAGCCGAAAGTATCACCAACGATGAAGTCGATTTTCAGTCCAAGATCAT
TAAACTGGCGTTCATGGCACGCTTAAGTGACCCGCACACAACCAACGACAACAAACTGAATAAAAAGGTGATTATCGAAAGCAACCAGCAGAGCAACAGC
AGTGAATCAGGTAACAAAGCCATTTTGTTCCTGAGCGATCTGCTGAAAAATAACGTACTGGAAGATCGTTATAGTCATCTGCCGAAAACTTGGATTGGCC
CTGTAGCACGTGATGGCGGTTTGGGTTGGGCGCCGGGCGTGCTGGGATACGATCTGTACTCGGGCCGTACAGGACCTGCGTTAGCATTGGCTGCGGCCGG
GCGCGTTTTGAAAGATAAAGACAGTATCGAACTTAGCGCCGACATTTTTAATAAATCGTCCCAGATTCTGCAGGAAAAGACTTACGACTTTCGTAACCTG
TTCGCATCAGGTATCGGCGGTTTTAGCGGATTACCGGTCTGTTTTGGGCGCTGAACGCGGCAGGGAATATTCTGAACAATGATGACTGGATTAAAACCT
CGAATCAGAGTATGCTGCTGCTGAATGAGAACATGCTGAAAGTGGACAAAAATTTCTTTGACCTGATTAGCGGCAACTCGGGAGCGATCGGTATGATGTA
CCTGACCAATCCAAATTTCTATTTGTCTCGCTCGAAAATTAACGACATTCTGCTGACCACGGACTGCTTGATTACTGAAATGGAAAAAGACGAAACGAGC
GGACTGGCCCATGGCGTGTCTCAGATCCTGTGGTTCCTTAGCATTATGATGCAACGTCAGCCCTCAAGTGAAATCAAAATCCGCGCGACGATTGTCGACA
ACATCATCAAGAAGAAGTATACGAATTCCTATGGCGAAATCGAATGCTACTATCCGACTGATGGGCACTCCAAATCCACCTCGTGGTGCAACGGGACAAG
TGGGATTCTGGTCGCCTATATTGAGGGGTATAAAGCTAATATCGTGGACAAATCCTCGGTGTATCATATTATTAATCAGATCAACGTCGAACAACTTCAG
CATGATAACATTCCGATCATGTGCCATGGTAGCCTTGGTGTGTATGAATCGCTTAAATATGCGTCAAAGTACTTTGAAATCGAAACCAAGTACCTTCTGG
ATGTGATGCGCAATGGCGGCTGCTCCTCCCAAGAAGTATTAAAGTACTATGGCAAGGGTAACGGCCGTTACCCGCTGTCACCAGGTTTAATGGCGGGTCA
GTCGGGCGCGTTGCTGCACTGTTGCAAACTGGAGGATAACGATATCAGCGTGAGCCCCATTTCACTGATGACGTAATAA

pEG7077 LtnM2

AGAATTCCACCCCCCACTAAGGAGGTTTTTTATGGATCCGAGTATCAAAAAGCTCGTGGATTCTATCATCGAATTCTACAAAAAGGACATCTACCTGGCA
TACAAAGAGCTGGAACGCGAAATCAAAAACATCGATAAGACCATCTACAACACTTCAAATGACGAGATCTTGCGGATTTTTAAAGAGAGCCTGATCAGCA
TCATCACCGATGATATTTACCGCCTCTCGATTAAAACCTTCATCTATGAGTTTCACAAGTTTCGTATCGATAACGGGTTTCCGGCTGTCAAAGATAGCGA
AAGCGCCTTCAATTATTACATCAGTACCTTTGACGTGAAAACGATCGCTCGCTGGTTTGAGAAATTCCCAATGCTGGAATCCATCATCTCCAGTAGCATC
AAAAACGATTGCACATTTATGGTGGATGTATGTGTCAATTTCATCTTAGACCTGTCGGAATGCGAGAAGATTAATCTGATCTCAGAGGATAGCCGGCTCA
TCACGATCTCATCCAGCAACTCTGACCCGCACAACGGTGGCACGCGTGTCTTGTTCTTTCGTTTCCACAACGGTGATACCATTCTTTACAAACCCCGCAG
CCTGACCGTGGACAAGCTGATCTCTAATATTTTCGAAGAGGTATTCGAATTCGATGCGACGAACTCGAAAAATCCTATTCCCAAGGTGCTGGATCGGGGT
ACCTATGGCTGGCAGGAATTCATTGAGAAGAAATCGATCTCTTCCTCAGAGATTAAGCAGGCCTACTATAACCTGGGTATCTTTAGCAGTATCTTTACAG
TGTTAGGGTCTACTGATATCCACGATGAAAACTTGATTTTTAAAGGTACGACCCCGTATTTCATCGATCTGGAAACAGCCCTCTCTCCGCGTATCCGGTA
TGAAGGTAATGAGGAAAACCTGTTCTATCGGATGAGCTCATCGTTGTTCACTTCTATCGTGGGGACGACTATTATTCCTGCAAAACTTGCTGTCCATTCC
CAGGAAATTATGATCGGCGCAATTAACACCCCTGCGAAACAGAAAACCAAGAAGGATGGCTTTAACATCATCAACTTCGGCACGGATGCCGTCGATATCG
CAAAACAGAATATTGAGGTGGAGCGTATTGCTAACCCTATGCGCATTAAAAATAACATCGTGAACGATCCGCTGCCGTACCAGAACATCTTTACGCGCGG
CTTCAAAGAGGGGATCAAATCCATCATCCTGAAGAAAGGCTCGATCATTTCCATTCTGAACAACTTCAACAGCCCGATTCGTTACATCATGCGGCCGACG
GCAAAATATTATTTGATTCTGGATGCCGCGGTATTTCCCGAAAACCTGTATTCGGAACAGACACTGAACAAAACCCTGAATTACTTAAAGCCGCCAAAAA
TCGTGGAAAATTCCCTGATTTCTAAACAGCTCTTTCTTGCCGAAAAACGCATTCTGTCCGAAGGCGATATTCCGAGCTTCTATGTGCTGGGCAAAGAGAA
AAATATCCGTGCGCAGAACTTCATTAGCGAACAGATCTTCGAGGAAACCGCGGTCGATAACGCGATTCAAATTCTGGAATCCATTTCGCAAGACTGGGTG
AATTTTAATGAGCGCCTGATTGCGGAGGGCTTCTCCTATATTCGTGAACAGAGTCGTGGCTATCTGTCCAGTGATTTTGAGAACTCTGATATTTTCAAAA
GCTCACTGACCGAAACAAAGAAGTCCGGTTATACCGCAATGCTGAAAACAATTATCTCCATGTCGGTCAAGACCTCGGAAAACAAAAAGATCGGTTGGCT
GCCAGGCATTTATGATGATTATCCGATCAGCTATATGAGTGCCGCGTTTTGTTCGTTCCATGATTCCGGCGGTATCATCACTTTGCTTGAACACCACTTT
GGGCACTGCTCCCCCGAATATAACGAGATGAAGCGCGGGCTGCTGGAACTGGGCAAAATGTTGAAAATTAACAATAGTAACCTGAGCATCATCTCCGGCT
CAGAGTCTCTGGAATTTCTGTATACGCACCGCGAAGTCGAATGCCTGGAACTGGAATACATTTTAAACAATTCAGCGGAAATCATGGGCGACGTGTTCCT
GGGGAAATTAGGCCTTTATCTTATCCTGGCGAGCTACCTGAAAACAGACCTGAAAATTTTCCAAGATTTCAGTATCATCTGCCAGAAAAACCTCGAGTTT
AAAAAGTTCGGGATCGCGCACGGTGAATTAGGGTATCTGTGGACCATCTTCCGTATTCAAAACAAACTGAAGAACAAAAATGCGTGTCTGAGCATCTATC
ATGAAGTGTTGAACATTTATAAAGGTAAGCGCATTGAATCCGTGGGATGGTGCAACGGTTTATCGGGTATTCTGATGATTTTGTCAGAAATGAGCACCGT
ATTAGAGAAAAATCAAGACTATCTGTTCAAGCTGGCAAATCTGAGCACTAAACTGAATGAGGAATCCGTTGACCTGAGTGTGTGCCACGGCGCCAGCGGG
GTGCTTCAAACACTGCTTTTCGTCTATAGCAACACGAACGATAAACGTTATCTCAGCCTGGCCAATAAGTATTGGAAGAAAGTGCTGGATAACAGCATTA
AGTACGGTTTCTACAATGGAGAACGCGATAAGGATTATCTGTTGGGATATTTCCAGGGTTGGTCAGGCTTCACGGACAGCGCACTCCTGCTGGATAAATA
CAATAACAATGAGCAAGTGTGGATTCCGATCAACCTGAGCTCCGATATCTATCAGCATAATCTGAACAACTGCAAAGAGAAGAATTATGAGGGCGATGGC
TGCCATAAATCTTAATAA

pEG7078 CrnM

AGATCACCCATACCAAGTATAACGAGAACCTCCATGAATGATATCAACAAAAACAAAACTAAAACCATTAACGAAAAGATTAAAATTTTCACCAAAGAAG
AGGTGATTGATATCAGTTACTTTGAAGAATGGCGCAGCGTTCGTACTCTGCTTAACGAAAACTACTTTAAAATTATGCTCGAGGAAATGAATATTTCCAA
AAACCAATTTTCGTATGCGCTGCAACCGTTAAACGACGAGTTCAAACTGCATACTAACGTTAAAAATGAAGAATGGATCAAATGCTTTAATCGCGTCATT
AACAATTTTAACTATAAAAATATTAACTATAAAGTTGGTTTGTACCTGCCTATTCAGCCTTTCTCCGTTTATTTACAGGAGAAACTGAAAGAGATCCTGA
AGAAGCTGAACAACATTAAGATTAATGATAAAATTATCGACGCCTTTATCGAAGCTCACCTGATCGAAATGTTCGACCTCGTCGGTAAAGTAATCGCCCT
TAAATTTGAAGATTATAAACAGATCAACTTCCTGAAAAACACAAATAATGGCACCCGCTTGGAGGAATTCTTGCGTAGCACCTTTTATTCTCGGAAGTCA
TTTCTGAAACTGTTTAACGAGTTTCCGGTACTCGCGCGGGTTTGCACCGTACGTACGATCTATTTGATCAATAACTTTAGTGCTATCATCCAGAACATCA
ATAGCGACTACCTGGAAATCCAGGAATTTCTGAACGTCGATTTCCTGAACTTGACAAACATCACTCTTTCGACGGGTGATTCCCACGAACAGGGTAAAAG
TGTGTCCATCCTCTATTTTGATGAAAAAAAGCTGATTTATAAACCGAAAAATCTGAAGATTTCAGAAATTTTCGAGAGCTTCATCGACTGGTACACCAAC
GTCTCTAACCATAAGCTGCTCGACCTGAAAATCCCGAAAGGAATTTTTAAAGACGATTACACTTATAACGAATTTATTGAGCCAAACTACTGCGAGAATA
AGCGCGAAATTGAAAATTACTATAACCGTTATGGGTACCTGATCGCAATCTGTTATCTGTTCAACCTGAATGACCTGCATGTAGAAAATGTGATCGCCCA
TGGCGAGTACCCGGTTATTGTTGATATTGAAACGAGCTTTCAAGTCCCTGTGCAAATGGAGGACGATACTTTATATGTGAAGCTGTTGCGCGAGCTGGAA
TTGGAAAGCGTTTCATCGTCGTTTCTGTTACCTACCAATCTGTCGTTTGGTATGGACGATAAAGTGGACCTGTCCGCGCTGAGCGGAACCATGGTCGAGC
TGAATCAGCAAATTCTGGCGCCTGTCAACATTAATATGGACAACTTTCATTACGAGAAATCACCGAGCTATTTTCCAGGCGGAAACAATATCCCTAAAAA
CAACAAATCAGTGACTGTTGATTATAAAAAATACTTGCTCAATATTGTGACTGGTTTCGACGAATTTATGAAGTATACCCAAGAAAATCAGCTGGAATTT
ATTGAGTTCCTGAAAAAATTCTCAGATAAAAAAATCCGGGTGCTGGTGAAGGGTACGGAAAAATATGCGTCCATGATTCGCTACAGCAACCATCCGAACT
ACAACAAAGAAATGAAATATCGCGAGCGTCTCATGATGAACTTGTGGGCGTACCCTTACAAAGACAAGCGTATTGTTAATAGCGAAGTACAGGACCTGTT
ATTTAACGATATCCCGATCTTTTACTCCTTTCCAAATAGCCGTGACCTCATTGATAGTCGCGGCTTGGTGTATAAAGATTACCTTCCTGTGACAGGACTG
CAGAAAGCAATTGATCGCGTGAAAGATACCTCGGTAAAAAGCTTGTTCGACCAGAAGCTGATTCTTCAGAGTAGCTTAGGTCTGTGGGATGAGATTCTCA
ACAAGCCGGTCCAGAAAAGGAACTGCTCTTTGAAAAGCAGAACTTTAACTATGTGAAAGAGGCGATCAATATTGCGGAATTGCTGATTGGCTATTTAAT
CGAAACGGACGACCAGAGCACCATGCTGAGCATTGATTGTTCTGAAGATAAACACTGGAAGATTGTTCCTTTAGACGAATCCCTGTATGGTGGGCTGTCC
GGCATTGCATTATTTTTTCTCGATATTTATAAAATTACCAAAGATGAAAAATATTTTAATTACTATGATAAAATCATTTCCACGGCCATTAAACAATGTA
AAGCGACCATCTTCTCGTCAAGCTTCACGGGTTGGCTGAGTCCCATTTATCCGTTGATTCTGGAAAAGAAATACTTTGGTACCATGAAAGATAAGAAATT
CTTTGACTACACGATGGAAAAGCTGTCGAATATGACTGAAGAACAAATTAACAACATGGATGGTATGGACTATATCAGTGGCAAGGCGGGTATTGTCAAA
CTGCTGATTAGCGCGTACCGGGAATCGAAGAACAATGAAAACATCGGACTGGCCCTGAGTAAATTCAGCAACGATCTGATTCAAAATATTGGCACCGGCA
AAGTCAGTGAATTACAAAACGTGGGCCTGGCGCACGGCATTTCTGGTATTATGGTCGTAGTAGCCTCACTGGACACGTTTAAAAGTGAATATATTCGCGA
GCAGCTGGCAATTGAATATGAGATGTTCTGTTTGCGTGAAGATTCATACAAATGGTGTTGGGGCATCTCTGGAATGATTCAAGCCCGTCTCGAAATTCTG
AAACTGAGCCCGGAGTGTGTGGATAAAAAAGAGCTGAACTTGCTTATTAAGCGTTTTAAAAACATCTTGAATCAGATGATTAACGAAGATTCCCTTTGTC
ACGGCAACGGTTCGATCATTACTACGATGAAGATGATCTATATGTACACCCAAGACACCGAGTGGAACTCTCTGATTAATCTGTGGTTATCAAATGTAAG
TATCTATTCGACCTTACAAGGCTATAGCATTCCAAAGCTGGGCGATGTAACAATTAAGGGGTTGTTTGATGGCATTTGTGGTATTGGCTGGTTATACCTG
TATTCGAACTTTAGCATTGAAAACGTGCTGCTCCTCGAGGTCTAATAA

pEG7079 BsjM

AGAGACGGGCGGCCACCAGGAGGAACGAGAATGATCAAAAATGTAAACCTCAAAGAGGCCATTAAAGGTTTGACCGTATCAGAACGTTATGACACTCTGA
AAAATTCGGGAGTCAACCTGAATCTGAACATTTCGGCTTTGGAAGAGTGGCGCAACCGTAAGAATCTTTTAGCCGATGAGGACTTTACGGAGATGCTGAC
GGTGCTGGAATATGACCCGGTGTATTTTAGCCACGCGATTAACGAGAACATCGAAGAACATATCGATATCTACAAGAGCAAAATTCTGGGGGAAAACTGG
TTTATCGTGCTGAACGATATTCTGGACGAGCTCGATAATCCCATCGAATACAAGAAAGAGATGAATCACAGCTACCTCCTGCGTCCGTTCTTGCTCTACG
CCGAAAAGGAGATGAACAAATACATTGTCAATCGTAAGGAGTTACTTCCGGTGGAACCCCAGGTCATCCAACAGATCATGGAAAATTTGGCCTCCAAACT
GTTCGCCGTTTCTGTGAAAAGCTTTGTCCTGGAGCTGAATATTTCGAAATTGAAGGACGAACTGGCCGGCGAAACACCGGACGAACGCTTTCACTCATTT
ATTCGTTTGATGGGTGAGAAAACGCGCCTGGTGGACTTTTACAACGAATATATCGTTCTGAGTCGTATTCTGGTGAACATCACGATCTTATTCGTCAACA

ACATTATTGAGCTGTTTGAGCGCCTGCAGGAATCCAAGCTGGATATTGTTAAGAAACTTGGCGTGCAGGAGGAGTTCAAAATCAGTAATATTAGCATTGG
CGAAGGTGATACACATCAGCAAGGACGCTCGGTTATCGTTCTTACGTTCGTGAGTGGAAAGAAAGTGGTGTATAAACCAAAAAATCTGAAAGTTGTTTCT
GCTTATAATTCTTTAATTGACTGGATCAACAATAAAAATAATATTCTGAAAATGCCTTCGTATAACACATTGATTTATGATGATTTCGTGATCGAGGAGT
TTGTCGAGAAACGTGACTGCAAAAGTATCGAGGAGGTCAAAAAATATTATATTCGTTATGGGCAAATTTTGGGGATTATGTATATCTTAAATGGGAACGA
TTTTCATATGGAAAACCTGATTGCCTCGGGTGAATATCCGATCATTGTTGACTTGGAAACGCTGCTTCAGAACATTATCAATTTTAAAAACAAACCATCA
GCGGACTTGATCACCACCAAAAAGATGCTTAACCTGGTAAACAGTACTCTGCTGCTCCCTGAAAAACTTCTGAAGGGCGACATCACGGACGAAGGAATCG
ACATGTCAGCCTTGGCAGGGAAAGAACAACACTTGGAACGCCGCGAATACCAGTTGAAAAACCTGTTCACCGACAACATGGTTTTTGATCTCGAAAAAGT
GAAAATCGAAGGTGCGAACAACATCCCGAAATTAAACGGTGAAAACGTTGACTACAGCACCTATATTGATGAGATTGTGGTTGGGTTCGAAAATATCTGT
AACCTGTTCATTCAATATCGCGACGAGTTACTGCATTCCGGCATCCTGGAGGAGTTTAAAGATGTGAAGGTTCGTCATGTGCTTCGCAATACGGTTGTTT
ATGCTAAGATGCTGGCGAATACATATCATCCAGATTACCTGCGTGATTCGTTGAATCGCGAACAGGTTCTTGAAAACATTTGGGTGCATCCGTTTGAGCG
CAAAGAATTCATTAAGAGCGAGATGGAAGATATCCTCAACAACGACATCCCGATCTTTTTCTCATACGCGTCGTCTAAGGATATTATCGATTCGAATGGC
AAACTGCACAAAAACGTTATGGAAATTTCGGGTTACGAACGTTTTACCACCAAACTGAAGGAACTGAATCCCTTTCTGATTGAACAGCAGGTGAGCGTTA
TTAATATTAAAACCGGCCGCTATGGGGATAAGAAATTCGAAAAAAATTATAGCGTGCGCGACGTTGCAACGGAGAAAAAAGATAATCCGATTGATTTCCT
GCAGGAGGCAATGAATATCGGCGATAAAATTTTGGAACATGCTATCATCTGTGATGAGACCAAAACGATTTCGTGGCTTACCATTAACAACCATCATGAT
AAAAATTGGGAAATTGGGCCTATTTCCGGTGAATTTTATGATGGTCTGGCGGGAATTTCACTCTTCTACCACTACCTCTATAAAAAATCCCACAATGTCG
AGTATAAAAAAATTCGTGATTACGCGTTCAACATGGCGAAAGTCAAAGCCCTGTCACTGAAATACGATAGTGGCTTGACCGGTTACGCTTCCTTGCTGTA
TACGGCACACAAGATTGTTCAGGATGAACCGCGGAAGCAATACAAAGACGTGATCAACGAAGTGTTCAAGTACATTGATGAGAGCAAAGTCGTGACCGCT
AAGTATAACTGGTTGCATGGCACTGCCTCTATTATTCATGTGTTATTGAACCTCTACGAGGACTCTCGTGATATGGCGTACCTGACTAAATGTATTCAGT
ACGGCAAATATTTGGTCAAGCAAATCAAAGAACACAAGGATATGCTTGCGCCTGGCTTTAGCCAGGGCATCTCTTCGGTCATTATGGTTCTGGTGCGCTT
AAGTAAAAAGTGTGAAGTCGAAGAATTTCTCGAATTAGCTCTGGAATTAATGGAAATGGAACGCAACAAACTGGGAAACCTTTCTGAATCAAACTGGCTG
AACGGCTTGGTGGGCATTGGCTTATCACGTATCAAACTGAAAGGACTGGATTCCAACTTACAGGTCGACAACGACATCGAACTCGTCCTGGATGGCGTCA
TGAACAGCTTGTACTCAAAAGATGATACTTTGAGCTGTGGTAACTCTGGCACAGTGGAATTGTTCCTGAGTCTGTTTGAACAGACGAAAAAGAAAGAGTA
TCTGGATATGGCGAAAGCAATCTGCGGGAAAATGATCGAAGAGAGTCGCATCTCCTTTGAGTATCAGACAAAGAGTCTGCCGGGTTTAGAACTGGTGGGC
CTCTACTCTGGCTTAGCCGGAATTGGTTATCAATTCTTACGTATCTCGGACGTTGAGGATATTGCGAGCATTGCTACCTTAGATTAATAA

pEG7127 PsnB

AGACGAATATAAGGAATAAAATAATGACGAATTTAGACACGAGCATTGTGGTCGTAGGAAGTCCGGATGATCTTCACGTCCAGTCAGTGACGGAGGGTCT
GCGTGCACGCGGTCACGAGCCTTACGTGTTTGACACCCAACGTTTTCCGGAAGAGATGACAGTGTCACTTGGTGAACAGGGTGCCTCTATTTTTGTCGAT
GGCCAGCAAATTGCACGTCCGGCGGCGGTGTACCTCCGTTCACTGTACCAGAGCCCCGGCGCGTATGGGGTGGATGCCGACAAAGCGATGCAGGATAACT
GGCGCCGCACATTGCTCGCTTTTCGCGAGCGTAGTACCCTGATGAGCGCTGTGCTTCTGCGTTGGGAAGAAGCGGGGACTGCAGTGTATAATTCGCCACG
CGCGTCGGCGAATATCACTAAACCGTTTCAGCTGGCGCTGCTGCGCGACGCTGGTCTGCCGGTACCACGTAGCTTGTGGACAAACGACCCTGAAGCAGTG
CGGCGGTTTCATGCGGAAGTGGGTGACTGTATTTACAAACCGGTCGCCGGGGGAGCGCGTACACGCAAACTGGAAGCGAAAGATCTCGAAGCGGACCGCA
TCGAACGCCTGAGTGCAGCGCCGGTGTGTTTTCAAGAACTGCTCACAGGAGATGATGTGCGTGTTTACGTGATAGATGACCAGGTAATATGCGCCCTGCG
CATCGTAACTGATGAGATCGATTTCCGCCAAGCAGAGGAACGTATCGAGGCCATCGAAATTTCAGATGAAGTAAAAGACCAATGTGTACGTGCCGCCAAA
CTTGTTGGCCTGCGCTACACCGGTATGGATATCAAAGCCGGCGCCGATGGTAACTATCGTGTTCTCGAACTGAACGCGAGTGCGATGTTTCGCGGTTTCG
AAGGCCGTGCGAATGTGGATATCTGTGGACCGCTGTGTGATGCATTGATCGCTCAGACCAAACGTTAATAA

pEG7130 AMdnC

AGAGGCTATATAGGATAGGGGGGTCCCCATGAACGTTCTGATTATAACGCATTCCCACGATAACGAGAGCATTTCATTGGTAACCCAAGCCATTGAATCC
CAGGGTGGTAAAGCATTTCGCTTCGATACCGATCGTTTTCCGACGGAAGTCCAGCTGGACATCTATTACTCAAATACAGAGAAATGCGTGCTGGTGGCTG
ACGATCAAAAACTGGATTTAAATGAAGTAACCGCGGTCTGGTATCGCCGCATTGCGATCGGTGGCAAAATCCCGCCCACGATGGATAAGCAACTTCGTCA
GGCCTCGATTCAGGAGAGTCGTGCTACAATTCAAGGCATGATAGCGAGCATTCGCGGCTTTCACCTTGACCCAGTGCCGAACATTCGTCGCGCTGAAAAT
AAGCAACTGCAGCTGCAGGTTGCCCGCAAAATCGGACTGGATACCCCACGCACTCTCACCACTAATAATCCGCAGGCCGTGAAGGAATTTGCGGCAGAAT
GCCAGCAGGACGTAATCACCAAAATGCTGAGTAGTTTTGCGATTTATGATGAGAAAGGCGGAGAACAGGTGGTTTTCACCAATCCCGTGAAATCTGAGGA
TCTGGAAAATTTAGAAGGTCTGCGCTTTTGCCCTATGACGTTTCAAGAGAAAATCGCAAAGGTTCTGGAGCTCCGGATCACCATCGTGGGTAAGTCAATT
TTAACGGCTGCGGTGAATTCACAGGCCCTGGACAAATCCCGTTATGATTGGCGCAAGCAGGGCGTAGCATTACTGGATGCATGGCAGACCCATACGTTAC
CCCAGGACGTGGCTGATAAATTGCTTCAACTGATGGCCCATTTCGGGTTAAACTATGGAGCCATTGACGTGATTCTGACCCCGGATAATCGCTATGTGTT
CTTGGAGGTCAATCCGGTGGGCGAATTCTTTTGGCTTGAGCGTTGCCCAGGTCTGCCGATTAGTCAAGCTATTGCTAAAGTGCTGCTTTCTCATATATAA
TAA

pEG7132 AtxBC

AGAGCTGTTAGTCGCTGCCAGGAGGTCCCGTATGTACGAGCTGAATGATGGCGTAGGTTTGGCCCTCGTGGATCAGCATCCGATTTTTCTGGACCTGAAA
ACAGACCGTTACCTGTCGTTGAGTCCAGATGGGGCAGCAGTCCTGCTGGGAGCAGCGCCAGCCACCAAAGAGAGTCCACTGTTTCTCGGATTAGAATCCA
TTGGCTTGGTCAAAAACGGTCCGTCAGGCCTTAAGCCTTGCCAAATTGCCGTAGCCACTGGGTCTGCACCGCCCCGTAAGGTGCAATTCGAGTCGTTGTC
ACTCCTGCTTTTGCGCTTAATTCGTGCACGTCTGGATCAACGTGCTCTTTTGAAGCGTGTGACCGACTTAAAGAAGGCCGGCACCATTGCCCAGACGAAG
AACCGTGACTGCGCCTTGTCATTATTAGGTAGCGTGGAGACTGAGGCAAAGGCTTGTCGTACCCTTTTAAGTAGTACAGACAAATGCCTGCCCGACGCAT
TCGCAATTGCAACGCACCTGCGCCGTCGCGGAGTAGACGCCAAGTTAGTTTTCGGTGTGCGCCTGCCATTCGCGGCACATGCCTGGGTCCAGGTAGATGA
TATTGTAGTGGGTGATCGTCCCGACCGTATCCTTGCGTTCACCCCCATCTTAGTCGTTTAATAACTTTTAACATCCCTTCTCATAAGGAGGTTTTAATGC
GCTATGTCGCGTCTTTCTTTGTTCGCGGACATGTCAGCACACCAGCACTGCGTCACCCAGAGCCAAAGGGTTTCGCTTATGCAAAAGTCAGTGGCGGACT
GAGCGTATGGAGCGATGCGCCGATTCGTCACCGTGCGCCCCTTATTACAGTGGGCGCGGTGTTCGATCGCGCGTCTTTTAAAGGGCTGGATTGCGACTTA
TCAGGTCTGCGTCAGGATGGTCTTAATACATTGAAAGCGGAAACGTTCGGACCCTACCTGGCGTTAGAGGTTGCCGATAACGGCACCCTTCGCGTTTATC
GCGATCCGTCAGGCGGCGCGCCTTGCTATTACCTGCAGACCGAGGACGGCTTCTGGCTTGCAAGCGATGCTGATTTGTTATTCACTCATTCGGGCGTACA
TCCATCAGTAAGCTTACCGGGACTGATTGAACACTTGCGTCGTCCAGAGTTCCAAAATGAGGGCACATGCTTAAACGTCAAGCAAGTACGCCCTGGGGAG
CAGGTTGATTTATCGCTCTCGGGCGAGGTCCGTGCCTGTTTGTTCCCGCCTGCATCATCCCTGCGCCCGCCTGAGTTGCACCGCGCATACGATGACATTA
AGGCTGAGCTGCGCGCTCTGATTTTACGCAGCATTAAGGCCTATGCCAGTGATTTCCCTCACGTTGTTGTTAGCTTCAGCGGTGGTCTGGATAGCAGTGT
TGTTGCGGCCGGCTTAGCGCAAACTTCCACTAAGGTCCTGCTTCACACCTTTAAGGGCCCAGATGCCAAAGGGGACGAGACTGCCTTCGCCGCAGAATGC
GCGGCATATCTGGGTTTAAGCTTAGAGATTGATACTCTCAGTATCGATGACGTTGATCTGTCGGCAACTATTTCCCCGCACCTGCCGCGCCCCAGCACAT
CATTCTTCTTGCCATCACTGCTGCGCGGTTTCTCTACCTCGAGCCAAACGCGCACAGGCGGGGCAATCTTTTCGGGAAACGGCGGTGACTCGGTCTTTTG
TTTCATGCATAGCGCGACCCCGCTGGCCGATTTGATGTGTCGTCCGTCAGGTCTTACGCCGTTCATGCAAACATGGGCCGACGTGCAAAAGCTTACCCGT
GCCTCAGCGACCGAAGTGCTGCGTCGCGCGTTAAAGACAGCCATGGCGCGTGGCTACATCTGGCCTGAATCCAATCTCCTCTTGTCCCGCGACACAAGCT
CGAGCCGTTTAACACCTGACTCCGTTCTGTCGAGCCTTGAGGGGATTCTGCCCGGTCGCTTGCGTCACCTCGCCCTGATTCGTCGTGCTCACAACACCTT
CGAGCCATTCGCCCCTTGGCGTACGCCGCCAGTCGTTCACCCTCTCATGGCCAAGCCGATTCAAGCCTTCTGCCTTTCTCTTCCTTCATGGATGTGGGTC
AGCGGTGGTAAAGACCGCTCGCTCGTGCGTGACGCGTTCGAAGGATTACTTCCAGATTCAGTGCGCCTTCGTAAATCAAAGGGAAGTCCTGCAGGCTTTC
TGCATGCGCTGTACCGCGCCAAGGGTCGTCAAATGATTGAGCGTATCCGTCACGGTTACCTGCGTCGTGAGGGGATCATCGATATCTCTACTGGCCCGGA
CGCATTGTTCTCGGAAGGGTTCCGCAATCCGCGTGTAATGCACCGTTTCTTTGAGCTCGCCGCAACTGAGGTGTGGATCGATCACTGGCGCAACTGGCGC
CGCCCCCGCACATAATAA

pEG7133 Cln1BC

AGACAGTAGTATAAAGGAGGGTTCAAGTATGCCTTTATGGTTAGCGCAGGACGTCCACGCGGTCGCTCTGGACGAAGATATCGTGGTGCTGGATGCGGTG
AGCGACGCATACCTGTGTTTAGTTGGTGCCAGCGCTCTGATCAGCTTGGGCAGCGAGCGTTCCGTCAGTGCAGATCCGGTGGCCGCTGAGACACTTCGTG
AGGCTGGTCTGGTGGGTCCACATCCTAGCGGCGCCACCCGACCAATACCTCCGAAGCCGACGATTGACTTACCTGATGCAGCCCGTCAGGCGCAAGGTCG
TGAATTACGTGCCGCCGCGTGGGCTGGCGCGGCAACCGCAATCGATTTCCGCCGGCGTTCATTTAGACAACTCCTCGCGAGAGCAGGGCAACGCCCGCCG
GGTCAAGCAGCTGCTCCGGCTGATGAGGTATTGGCAGCAGCCGCAGTGTTCATGCGGTTACGTCCATGGTCACCCGTTGGAGGCGCGTGCCTTATGCGTT
CGTATTACTTATTACGGCATTTGCGCATCCTCGGTTTCGATGCCGATTGGATCATTGGTGTGCGTACGTGGCCATTTATGGCCCATTGCTGGCTGCAGGT
CGGTGCCGTCGCACTCGACGATGACGTCGAGAGATTAACAGCATACACACCGATTCTGGCGGTGTAATAATTCAATAAATTAAGGAATTTTGATGGGCGA
CTACCTGGCTCTGTACTGGCCGCGCGGCATGCCCGGTGTAGCTGCAGACGCAATGCGGGCCGCCATCGAAGCTGAGGGCGCCTGGACCCTGGCGTTCGAG
GCCTACCAGCTGGTAGTGTATGTCAAAGGGCCCCGAGCACCTAAAGTGCGTGCCCTGCCGGATCAGGGCGGGGTGGTCATTGGGGAACTGTTTGATACTG
CAGCAACCCGCGAAGGACGCGTGCAGGACTTTCCTATAGCGCTGATCAAAGACGTCGCAGCTCAGGATGCCGCACGTATTCTTGCTACCCATGCGTGGGG
TCGTTATGTGGCTGTATTAAAAGCCGGTGATCGTCCGCCATGGATCTTTCGCGATCCAAGCGGGGCGGTGGAATGTCTGGCGTGGGTCCGCGATGAAGTG
ACCATCATTAGCAGCGATGTTGCAGCGCAACGAGCTTGGTCCCCTGATCGGCTGGCGATTGACTGGTCGGGACTGGGACGTGTACTGGCACGCGGAAACT
TATGGGGAGAAATTTGCCCGCTGGCTGGCGTCACGGCGATTGCGCCAGGTACCGCACGGTGTGATCTCGGTGATGCAGCTCTGAGCCTGTGGCGCCCAGG
AGATCATGCACGTCGTAGTCGTCATGATGTTTCCCCACGTGATTTGGCAAGAGTGGTGGATGCTAGCGTTGCAGCCCTGGCTAGAGATCGCAGCGCTATT
CTGGTCGAAATCAGCGGGGGACTGGATTCCGCTATCGTTGCCACGTCGCTGGCTCGTTGTGGAGCCCCAGTTGTTGCTGGAATTAACCATTACTGGCCCG
AACCGGAGGGTGATGAACGTCGCTGGGCCCAGGACATCGCAGATCGGTGCGGTTTTCGCCTGATCGCGGGCCAACGTCAGCGGCTGTTGCTGGACGAGGC
AAAGCTGCTGAGACATGCACAGGGCCCGCGACCTGGTCTGAATGCGCAGGACCCGGACCTCGATCACGATCTGGCGGAACAGGCTAAAGCGTTGGGTGCC
GATGCACTGTTCTCAGGGCAAGGTGGCGATGGTGTGTTCTATCAAATGGCAAATGCTGCACTGGCAGCCGATATCCTCATGGGGAAACCTGCTCCTATGG
GTAGAGCCGCGTCTTTAGCCGCTGTGGCTCGTCGGGCACGAGCCACGGTCTGGAGTTTGTGCGGCCAGGCTATGTTTCCGTCGCGCGCATTTGCCGCTGG
TATGCCGCCGCCAAGTTTCTTGAGCGCCGGTTTGGCGCCGCCACCCGTGCACCCGTGGATTGCAGACCAGCGCGGTGTTTCACCGGCGAAACGTATTCAA
ATTCGGGGGCTGACCAATATTCAATGTGCTTTCGGCGATAGCTTACGGGGCCGAGCAGCAGATCTTTTATATCCGCTTATGGCCCAACCGGTCATGGAAC
TGTGTCTGTCTATCCCTGCACCGCTGTTGGCAGTAGGCGCATTGGATCGCCCTTTCGCACGTGCGGCGTTCGCAGATCGATTACCTCCTCGTTCACTCGT
TCGACGCTCAAAAGGTGATGTTACCGTGTTTTTCAGCAAAAGCCTTGCAGCAAGCCTGCCGGCCCTTCGTCCTTTCCTGCTGGACGGGCGCCTTGCAGAA

CAGGGTCTGATCGATCGAGCAAAACTGGAACCTCTGCTGCACCCCGAACCGATGATTTGGCGCGACTCAGTCGGCGAGGTAATGCTGGCAGCGTATCTTG
AAGCCTGGGTGCGCGCATGGGAAGCCAAGTTGCGTGTTAGCTAATAA

pEG7134 Cln2BC

AGAACCACTATAAGGAACGATTTATGACTCTGACCTGGCGCCCGGGTGTTCACGCGGTAATGGTCGAAGATGATCTGGTTCTGCTGGATGAAGCAGCGGA
CGCTTATGTCTGTTTGTTGGATGGCGCCAAAGTGGTTAGCGTCCGGGCTGACGGTGCTCTGAGCTTCAATCCCCCACATGCAGCAGAAGATATGATCGCG
GGTGGCCTCGTCGAACCTTCATCAAGTGCCGCGGCGTCAGCAAACCCGCCGGCAAAACTCCCATGTACTCCGCTGGCGCGCTTATCGCGCCCGCGGCATG
TAAAAGTGCGTCCGGCTGAAGCGGCCTTGTTCCTGATCCAAGCCTGGGGTGTTGCGCGTGCGGTACGTCGTTGGCCAATGGCTAGATTATTAGAAGCATT
ACGTGGAGATCGTGCCGCAGAACCGGCGAAAGGCCGCCGATCGATGGCGGAGGCGTGCGCTGTTTTTGATGCGCTTCTGGCCTGGAGCCCTTTTGACGGT
GAATGTTTGTTTCGCTCAGTATTACGACGTAGATTTTTAATGGCACTGGGCCATTCGCCGGACTTGGTGATAGGCGTGCGTACCTGGCCGTTCCGCGCAC
ATTGCTGGCTGCAGAGCGGAGTGGATGCCCTGGATGATTGGCCGGAACGGCTCTGCGCATATCGCCCGATTCTGGCAGCTTCTGCAAGCCAGGGTAGATA
ATAACAGTATAACTAGAACAACAAGGAGTCAGATAATGAGTTACCTGCTGATGACCTGGCCGCCGGGGCAGCCGAGCGTAGAAGCTGATGCACTTCACGC
AGCCTTTAACGGGCAGGGTGGATGGAGCCTGGTTTTGGAACGATTCTGCCTGCGCGTATACGTGCGTGGCGCGGCAGCCCCTGCAGTTACCCTTACCCCG
AAAGGAGGCGTGCTCATTGGTGAGATGTTTGATCGGGCTGCCACAGAAACGGGCGCCGTTGCCGCTTATGATCTGAGCCGCCTGGGAGATGACGACGGTA
TGGCCGTAGCCCGGCGTGTGGTGGACGAAGCGTGGGGGAGATATGTGTTGGTGCTGCCAGTTAAAGAACGCCGTCCAGTGGTTTTGCGAGAACCACTGGG
CGCGCTGGATGCGCTGATCTGGCGCAAAGGCGATGTCTGGTGCGTGGGGGCAGACGTACCCCCGGGTCTTGAACCAAAAGATCTGGGTGTGGAAGAGACT
AGACTGACGCACCTGATCGCGGAACCGGATCTGGCATCTGCGAGCCTGCCCTTAACCGGCGTCGCGGCAGTGATGCCAGGTACTGCGGTCGATGAAACCG
GCCAGGTGCACCGTCTGTGGACCCCCGCGCGTTTTGCTCGCTCCCCTCGCACTGACGCGTGGACTGCAGCCGAACGTATTCCGCTGGTTACCCGTGCGTG
CATCGCGGCGCTGTCTGCGAATCGAAGTGGTATTCTGTGCGAGATTTCGGGCGGCCTGGATAGCGCTATTGTTGCGACCTCTCTGAAAGCGGAAGGTGCG
AAGATTAGTAGCGGGATCAACTTCCATTGGCCCCAGGCTGAAGCAGATGAGCGCCCGTACGCACGCGCTGTTGCGAAAAGCGTGCGAACCCGGTTACAGG
TGGTAGCGAGTCGTGTAGCGCCCGTTGACCCGGAAACGTTTGATGAGATCGTGGTCGCGCGACCAAGTTTTAATGCCATTGATCCAGTCTATGATACCGT
ACTGGCCCAACGTCTGATTCAGGGCGGTGAAGGAGCCCTGTTTACCGGACAAGGTGGTGACGCAGTTTTCTATCAGATGCCAGCACCACAACTTTCGTTG
GATTTGTTGGCTCGTGGCCCCCGCCGCCGCGGTCTTATGGGATTATCACGCCGCACCAACCGCAGTGTCTGGTCGTTGCTGCGCATGGGCTTACGTGCAC
CCGTACGAGCAACCTTTCCCTACGGTGCGAGAGGTGCCGATCGTCCTCCGATGCACCCGTGGCTGGAGGACGCGCGTGGTGTTGGGGCCGCGAAACGGAT
TCAGATCGAAGCGCTGGTTGCTAACCAGGCCGTGTTTGAAGCATCTCGTCGCGGTGCGGCGGCTCATTTGGTGCACCCACTGCTGTCGCAACCGCTTGTG
GAGCTGTGCCTTTCAACCCCAGCGGCCGTGCTGGCGGGTGCCGAACAAGATAGAGCATTCGTGCGTAGCGCTTTTCGTGCGCAACTGCCACGCCTGGTCT
TAGATCGTCAAAGCAAAGGAGATCTGAGCGTTTTCTTTGCTAAAGGTGTGGCGCGGAGCTTGCCGGGCTTGCGTCCGCGTCTGCTCGAAGGACGCTTAGC
GGCACGTGGCCTGATCGACGTGGAAGCGTTATCACAAGCGATGCAGCCAGAAGCGATGATTTGGCGTGACGGTTCGGCCGAAATCCTGTGCCTTGCTGTT
CTGGAATCATGGCTCCGCTCTTGGGAGGCTCGTGGTGCATAATAA

pEG7135 Cln3BC

AGATCCCGATAAAGGAGGTCCTAATGCGCGTTGCAGTGCCGGATCATTTAGCGTATTGCGTAAAACAAGGTGGAGTTACGTTTCTGGACGTCCGCGGGGA
TCGTTACTTCGGCCTGCCGCCGGTGCTGGAACACGCGTTCGTTGCCATTGCCGAGGCGGATTTTCTGCTGAAAGAACCAAATTCACTTCTGGAGCCACTC
GAAGCACTGGGTGTCTTAGTGCGAGGCCAAGCCCGCCGTGCCGATCTGACAATTCCGTCTGCAAATCTGTCATGGGTGGATGAGGTCAGCCCGACCCCAC
CACGTCTTGACCCTGCGTCACTCGTCGCAACCGTCACGTCTGTTATTCGAACGCGTCTGAGCCAAAAGAGTAAGTCCTTGCAGGCTCTCTTGGAAGAGGT
CCGTACCCGCCGTCCGGGATCGCCGGCCCATAATTGGCAGCTGATGCGTCGTCTGACGGCTGGATTCCGTGCATCGCGTGCTTGGGCGCCGATAGAACCC
ATCTGCCTCCTGGACAGCTTGGCGTTACTGGATTTTCTGCATCGCCGTGGCCTGTATCCGCATATTGTTTTCGGTGTGATCCGCCAACCGTTTGCCGCTC
ATTGTTGGGTGCAAGCTGATGATGTAGTCCTGAATGACCGGCTGGATCATGTCGGTGAATATACACCGATCCTGGTGGTCTAATAATAACATAAGGAGGG
TTTCTAAATGGAAGATTACGTGGTCCTCATTTGGCCGGCACTCGCTGAAGCTCCTGCACGCGACTTGATTCGTCGCCTGCCGAAACTCAAAACCGTCATT
GAAACTAGCGGATTGGTGGTACTGCGCCCCGAAAATGGTGCGGGTCTGCGGGTAGGCGGGAACGGTGTGGTCCTGGGTAGCGTCTTTCGCACCGGCGGTG
ATCGCGAAACTGTTGCGGAATTTTCGGAATCGGAAGCATCCGCGATCGCCACGAGTCGTGGTCAGCAGTTAGTGACAGAGTTCTGGGGTGGCTACCTGGC
TGTTCTTGGAGATGCTTCGCGTTCCGAAGTGATGGTCCTGCGAGATCCTTCAGGTGCAATGCCGGCTTATTGTTTAGTTCATGGCGAAGTCCAGATCATC
TGCTCTCGCTTGGAGGTCCTGGAGGACGCAGGACTGGGGCAGCAGGCGCTGAACTGGGACGTGGTGGCGCAATTACTGGCCTTCCCAAACCTTCGAGGTC
GCTCAACGGGTCTTAAAGGCGTGGAAGAATTACTTCCCGGTTGCCGTCTGACATTTACGGGAGGACTGAAAACCGAAACGCTGACCTGGAACCCGTGGCT
TTTTGCCCGCCCATCTGCGCAAGCGCCTGAACGTGGAGTTGCGGCGACCGCCGTGCGTCAGGCGGTGGAAGTAAGCGTTCGAAAATGGGCTGATCAGAGT
TCACCGGTACTTTTGGAATTGTCAGGCGGGCTGGATAGTAGTATCATCGCCTGCTGTCTGGACGAACCGCGCACCGCGGCCACCTTCGTGAACTTTGTCA
CACCGACGGCCGAAGGCGATGAACGAGGATATGCACGTCTGGTTGCCAAGGCAGCAGATAAACAACTGATCGAGCAGGACATCCGGGCTGACGAAGTAGA
TGTTACCCGTCCAAGACCTGGCCGCCATCCTCGTCCGGCCAGTCAGGCGCTGTTACAGCCGCTGGAACAGGCTTGCGCTGAACTGGCACCTCAGTTGGGT
GCGAGAAGTTTCTTCTCCGGTCTGGGAGGAGACAACGTGTTTTGTAGCATTGCAACCGCAAGCCCGGCTGCGGATGCACTTTTGACTAGCGGTCTGGGCC
GACAGTTCTGGGCCGCAATCGGGGACCTGTGTGCACGTCATAACTGCACCGTATGGGCAGCCTTAAGCGCCACGCTGAAGAAACTGCTCCGCTCAGATCG
TCGTCTGGTGATCAAACCAAACCTGGATTTTCTGTCCTTTCGGGAGGACGCCATAGACCGTCCGGATCACCCATGGCTTGAAGTGGCCGCCGATCGTCTG
CCGGGGAAACGCGAACATGTCGCAAGCATTCTGTTGGCGCAAGGCTTCCTGGATCGTTATGAGCACGCTCAGGTTGCTGCCGTCCGCTTTCCCTTGTTAA
CGCAACCGGTTATGGAGGCTTGTCTGCGCGTGCCGACCTGGATGGCAAACCACCAGGGTCGCAATCGGGCGGTCGCACGCGATGCCTTCTTTGATCGCTT
GCCCCCGAGAGTACGTGATCGGCAGACAAAAGGAGGTTTGAACGCGTTTATGGGTGTTGCGTTCGAACGCAACCGTCAGGCCTTAGCTCGTCATCTGTTA
GACGGGCGCCTGGTACAGCGTGGCCTGATAGATGCAGTGGCAATAAAATCGGCGCTGGCCTCACCAGTCCTGGAAGGAGGAGCCATGAACCGCTTACTGT
ACCTGGCCGATGTCGAATCCTGGGTACGCTCATGGGAAGATGTGTAATAA

pEG7136 CsegBC

AGATCACTGCAATAGTAAGGAGGTATATAATGGACCTGTGGTTGAGCGCCGGGGTCTATGCTGTCATGATCGATGATGATGTAGTTTTCCTGGACGTCGC
CACCAATGCATACTTCTGCCTCCCAGCCGTTGGGAGCGTGTTGGCACTCGAAGGTCGTTCGCTGCGTGTGGCGGCTCGCGAACTGGCAGAAGATCTTATT
CAGGCAGGCTTAGCATCCGCGGCTGCGGCAATCGAACCCCCACCGAGCACACCAGCCCCAGTTCGCACTGCGCGTGCGGTATTGGAAGCTCTGCCGGCGC
GTGAAAGACCACGTCCACGTCTTGCCCACTGGCGTCAGGCGATTATGGCTGGCTTGGCGTCCCGTGCCGCTGAACGTCGACCATTCGCGCAGAGACTGCC
GCCGCCTTCAACGGGGGTTTCACCTCCGGCATCAGAAGGCCTGCTTGCCGATCTGGATGCGTTCCGTCGACTTCAGCCATGGTTGCCGTTCGACGGTGCT
TGTCTGTTCCGTAGCCAAATGCTGCGCGATTATCTCCTTGCGCTGGGTCACCGCGTTGACTGGATTTTCGGTGTACGTACGTGGCCGTTTGGTGCCCACT
GTTGGTTGCAGGCCGGCGACCTGGTGCTGGATGATGAGGCCGAACGTCTGATTGCGTATCACCCCATTATGGTACGTTAATAAAGCACCGAGGGGTCAAT
AATAAGGAGGTAAACATGGGGTATGCCGCATTGACTTATCCGGGTGGTTTAGCGGCAGCAGCGTTTGATGAGATGGTAGAAGCACTGATCGATGCTGGAT
GGACCTTGGCGTTGCGTGCGTTCAGACTCGCCGTTCTCACCGATGGTCAGGCTCCAGCCGTGTCGCCGCTGATGGGCAGAGGCGGCGTAGCAGGCGTTCT
CATCGGCGAAGCGTTTGATCGTCGCGCCACATTAGGTGGCGCGGTCGCACGTGCCGCGCTGGATGGTTTGGCTGACATCGATCCGCTGGAAGCAGGTCGC
CATCTGATTGAAACCGCGTGGGGCGGCTACGTGGGTATGTGGATTGGTCGGGCCGAAGCTGGTCCGACACTGCTGCGCGATCCTAGTGGCGCGCTCGAAG
CCTTAGCGTGGCGCCGTGACGGTGTAACCGTTATGTCAGCGCGCCCGTTGACGGGGCGCGCAGGCCCAGCTGATTTAGCAATCGATTGGCCACGTATCGT
GCAGATTCTGGCCGATCCCATTTCCGCGGCTCTCGGCCCGCCCCCTCTGACTGGCTTAGCGACCATAGACCCGGGCGCGGCGGTTCATGGCGCGGATGGC
CAAGAACGCTCAGTGCTGTGGACCCCAGCTGCAGTTGTCCGTGGTGCTCGTCACCGTCCTTGGCCAAGCCGTCAGGATCTGCGTCGCACCATCGATGCGA
CTGTCGCGGCACTGGCCTCGGATGCGGGCCCGATTGTCTGCGAAATTTCAGGAGGTCTGGACTCGGCCATAGTTGCGACTAGCCTTGCGGCGTCCGGTCT
GGGTCCGCAGCTGACAGTGAATTTTTACGGTGACCAGCCTGAAGCTGATGAACGCGGATACGCTCAAGCCGTCGCCGAACGTATCGGTGCGCCTCTGCGG
ACCCTTCGTCGAGAGCCGTTCGCGTTCGATGAAACCGTGCTGGCAGCCGCTGGACAGGCCGCACGTCCGAATTTTAACGCCCTCGATCCTGGATACGATG
CCGGGCTCGTGGGTGCCCTGGAAGCTATCGATGCTCGTGCATTATTTACGGGCCATGGCGGTGATACCGTGTTTTATCAAGTGGCGGCCAGTGCCTTGGC
CGCAGACTTACTGGGCGGCGCACCATGTGAAGGTAGCCGCCGTGCACGTTTAGAGGAAGTAGCTCGGCGGACCCGACGCTCGATTTGGAGTCTTGCATGG
GAAGCGTTTTCTGGTCGACCCAGCACTGTAAGCATTGAAGGTCAGTTGCTTCGACAGGAAGCAGAGAGAATTCGGCGCGTCGGCCTGACCCATCCGTGGG
TTGGAGGCCTGTCGTCTGTGACCCCTGCGAAACGCCAGCAAATCCGCGCGCTGGTCAGTAACCTGAACGCGCATGGCGCCACTGGTCGCGCCGAACGCGC
TAGAATCGTGCACCCGCTTTTAGCTCAGCCGGTGGTTGAAGCCTGCCTGGCGATTCCTGCCCCTATCCTCAGTGCGGGCGAAGGAGAACGCTCATTTGCG
AGAGAAGCCTTTGCAGACCGTTTGCCACCGAGCATTGTGGGCCGCCGAAGCAAAGGGGAAATTAGTGTGTTTCTTAACAGATCTTTAGCAGCCAGCGCCC
CCTTTCTGCGTGGCTTTTTACTTGAAGGACGGCTGGCGGCTCGCGGGCTGATTGATCGTGACGAACTTGCAGCCGCGCTGGAACCGGAAGCAATCGTCTG
GAAGGATGCGTCACGCGACCTGCTTACTGCGGCGGCCCTGGAGGCGTGGGTCAGACATTGGGAAGCACGTATTGGCGAGGGGGAAGCAGCGGAAGGTGAG
CGTGCTGCCGGTCGTGGTACCGCAGCGACGGGACCGCGTACAAGCGCGCGGAAGGCGAACACCCGTTAATAA

pEG7137 PadeK

AGACACCGAAACCTAAGGAGGGATATATGACCGAACGTGCCGCAGTGCGTACCGACCATTATAAAGCCTTTGGGTTTAGAATTGAAAGCGATTTCGTGCT
CCCGGAACTTCCGCCCGCAGGCGAACGCGAACCGCTCGATAATATTACGGTTCGTCGTACCGACCTGCAGCCGCTCTGGAATTCTAGTATCCATTTTTAC
GGAAACTTTGCCATTCTGGATCACGGACGCACGGTTATGTTTCGAGTTCCGGGTGCTGCTATCTATGCGGTACAGGATGCTAGCAGCATATTAGTGTCCC
CATTCGATCAGGCAGAAGAAAACTGGGTACGTCTTTTTATTCTGGGTACCTGTATTGGGATCATCCTGCTGCAGCGTAAGATTATGCCGCTGCACGGTAG
CGCCGTTGCCATTGATGGCAAAGCCTACGCGATTATCGGCGAATCTGGTGCCGGCAAAAGCACTCTTGCACTGCATCTTGTCAGTAAGGGTTATCCATTG
CTTTCGGATGATGTGATTCCGGTCGTTATGACCCAGGGCTCCCCCTGGGTGGTGCCGTCGTACCCGCAACAAAAACTTTGGGTGGACACTCTGAAGCACA
TGGGAATGGATAATGCAAACTATACGCCGCTGTACGAACGTAAAACGAAGTTCGCGGTGCCCGTGGGCAGTAATTTCCACGAAGAACCGCTGCCGTTAGC
TAGCATTTTCGAGCTTGTCCCGTGGGATGCGGCAACGCACATTGCCCCGATCCAAGGGATGGAACGCTTTCGTGTCCTGTTCCACCACACTTATCGGAAC
TTTCTGGTTCAGCCGCTGGGTCTTATGGAATGGCATTTTAAAACTCTGAGCTCGTTCGTTCACCAAATTGGAATGTATCGTCTGCATAGACCTATGGTCG
GATTCAGTACCTTAGATTTAACGTCGCACATTCTGAATATAACGCGTCAGGGAGAGAACGATCAATAATAA

pEG7138 ThcoK

AGAGCATTCCATAAGGAGAAATTTTATGACGAGAACCAACACCGGCTATCGTTATCGCGCGTTCGGCCTGCGCATAGACTCAGATATTCCGCTGCCAGAA
TTAGGGGACGGTACGCGCCCTGATGGTGACGCGGATCTGACGGTCGTCCGGTGTGGGGAAGCGGAGCCGGAATGGGCTGAAGGTGGTGGCGGGGGTCGTC
TGTATGCCGCTGAAGGCATTGTATCTTTTCGCGTGCCGCAGACGGCAGCGTTCCGTATTACTAATGGAAATCGCATCGAGGTGCATGCCTACTCGGGGGC
TGATGAGGATCGAATACGCCTGTACGTGTTAGGGACCTGTATGGGAGCGCTGTTACTGCAACGTAGAATCTTACCGCTTCATGGTTCGGTCGTCGCCCGT
GATGGTCGTGCGTATGCCATAGTTGGCGAAAGCGGAGCGGGCAAATCCACGATGAGTGCAGCACTTCTCGAACGTGGATTCCGCCTCGTTACGGATGACG

TGGCCGCCATCGTGTTCGATGAGCGTGGGACCCCACTGGTTATGCCGGCTTATCCACAGCAAAAACTGTGGCAGGATTCCCTGGACCGTCTGCAAATTGC
GGGCTCGGGCCTTCGTCCGCTGTTCGAACGCGAAACGAAATACGCTGTACCCGCGGATGGGGCATTCTGGCCCGAACCGGTTCCATTGGTGCACATTTAC
GAACTGGTTCATAGCGATGGTCAAACGCCTGAACTGCAGCCGATTGCCAAATTAGAGCGTTGCTATACCTTGTATCGCCACACATTTCGTAGAAGCCTGA
TCGTCCCCAGCGGCTTAAGCGCCTGGCATTTTGAAACGGCAGTGAAACTTGCGGAGAAAACGGGGATGTACCGTCTTATGCGCCCGGCCAAAGTTTTCGC
GGCTCGCGAATCTGCTCGGCTGATTGAAACTCACGCCGATGGTGAAGTGTCACGTTAATAA

pEG7139 StspM

AGAGGCGGTATTAAGGGGGCCAGAGATGGCGGATCATATTGCGGCCGGTCATGACACCGTCCTGAGCCTGGCCGAACGGACAGGTACCGATCCAGATCTG
CTGGGCCGTGTGTTGCGCTTCCTCGCTTGTCGTGGTGTTTTCGCCGAGCCTCGCCCAGGTACTTATGCCTTGACCCCTCTGAGCTTAACTTTACTGGAAG
GCCATCCGTCCGGTTTAAGAGAATGGTTGGATGCGTCGGGTGCGGGAGCGCGCATGGACGCGGCAGTTGGAGATCTGCTTGGCGCCCTCCGCTCGGGTGA
ACCGAGCTATCCACGTCTGCATGGTCGTCCGTTTTATGAAGATCTGGCGCTGCACAGCCGAGGCCCTGCTTTTGATGGACTGCGTCATACGCACGCCGAA
TCGTATGTTGCCGACCTGCTGGCAGCCTACCCGTGGGAACGCGTTCGTCGCGTGGTTGATGTAGGCGGTGGGACCGGCGTATTGGTCGAGGCGCTTATGA
GAACTCATGCGACCCTCCGTACAGTACTGGTCGATCTTCCAGGCGCGGTGGCTACCGCTACCGCTCGAATTGCGGCTGCGGGTTTTGGCAATAGATATAC
ACCGGTCACGGGTTCCTTCTTTGATCCGCTGCCTGCGGGGGCGGATGTTTACACCCTGGTTAACGTGGTTCACAACTGGAACGATGAGCGTGCCTCAGCT
CTGCTGCGTCGGTGTGCGGATGCGGGTCGCCGCGACAGTACGTTTGTTATCGTGGAACGCTTAGCGGACGATGCAGACCCTCGTGCCATCACCGCCATGG
ACCTCCGTATGTTCCTTTTTCTGGGCGGTAAAGAGCGCACGGCCGCACAGATTCGCGAAGTAGCTAGTGCGGCTGGCATGGCCCACCAAAGCACCATTAA
AACACCGTCTGGCCTCCACTTACTTGTTTTCCGTAAGAAACGTTTCGCTGCTCGCGGTCACGGTCGTCGCATGGTGACCTAATAA

pEG7141 LcnG

AGACTATCGATAATAGGAGGTAGACCATGGACGGAACCAACAAGCGCCTGGAGGACAAGTGGTTTGATATTAACTTCCTGGAAATGTATACACGCAGCTG
CCTGAAAACTTTTGGCTACTTCGACGAAATTCTGATCGTGAAGAAACGCATCGAGGTCCTGAAGAACGTGCTTGAAAAACAGTACTTGTCTACCAATGAT
TATGCTGAGGAGTTTTTCGAGCTGAATACCACCTTGGAGAGCATAAAAGAATACATCAAACTGAATCTGGTCATCGAGAAAGAACCGATCTCAATTTGCA
TTATGGTCAAAAACGAAGAACGTTGCATCAAGCGCTGCATTGATAGCGTTGAAATCCTCGCCGAGGAGATAATCATTATCGATACCGGCTCTACGGATAA
TACCATTAACATTATTGAGGAATGCGCAAACGACAAAATTAAAGTGTTCTCAAAAGAATGGCGTAACGATTTTTCCGAAATTCGGAACTATGCCATCGAG
AAAGCGAGTAGCGAATGGCTGGTGTTTATAGATGCCGATGAATATCTGGACGAAGCCTCGGTGCTCAACCTGCTCAGTACGCTCAACATCTTTAACAATC
ATAAGCTCAAAGACTCTATTGTCCTGTGCCCCATGATCAACGAAGCCAATAACACCATCCATTTCCGTACCGGGAAATTTTTCAGAAAAGACTCCGGGAT
TAAATTCTTTGGTACCTGCCATGAGGAGCCCCGCATTAAAGGCATGCCGAATTCTACCCTGCTGATTCCGATCAAGGTTGATTATCTGCATGACGGCTAC
CTGGCAAAAGTACAATCAAATAAAGACAAGAAAACCCGTAACATCGAACTGTTAGAAGGTATGGTGGAACTGGAACCGGATAATCCTCGTTGGGCGTATA
TGTTTGTGCGCGACGGATTTGCAATCCTCGATAACGAATACATTGAGAAAACTTGTTTGCGGTTTTTACTGCTGGACAAAAACGTACGCATCTGCGTCAA
CAACCTGCAAGACCATAAATTCACTTTGTCACTCCTGACGATCCTGGGCCGCCTCTATCTGCGCGAGTGCGAATTCGAGAAAAGCAATCTGATAATTCGC
ATTCTTGACGAACTCATCCCTAATAGTCTGGATGGTAAATTTCTGGCATTCATGGAGCGATTCAGCAAACTGAAAATTGAGATTAATACGCTGTTAACGG
AGGTCATCGAATATCGTCGTAACCACGAAGTAGATGAAACCAGTTTAATCAACACACAAGGCTACCATATCGACTATGTTCTGTCGATTTTGCTGTTCGA
AACGGGTAATTACGCGCAAAGTAAGAAATACTTCGATTTCCTGCAGGAGAACCATTTTCTGGAAGAACTGTTTCAAGACAGCTCTTATTCTATCATACTG
AAAATGCTCGAGTCAGTAGAAGATTAATAA

pEG7142 PalS

AGACCAAACAATTAGGAGGACAAATATGGGGAATTTGCGTGATTTCTACCAACTGATGAAAGATAACTATGCGGACTCTAATCTGTTCAAGGATTTGAAT
CTGATCCACAATATCTCCAACGACATCCAAATTGGAATTAATTGCGATTTCTCTGAAATGCTGGGAGAACTGGTAGGTAATTACGATTCCCTGAACTATC
CGTCAATCACCTGTGGTATTCTGACGTATAATGAAGAACGCTGCATTAAACGTTGTCTGGAAAGTGTGGTGAACGAATTCGATGAGATTATTGTCTTGGA
TAGTGTATCCGAGGACAATACCGTGAAAATTATCAAGGAGAATTTCAACGATGTCAAAGTCTACGTCGAGCCATGGAAGAACGATTTTTCATTTCACCGC
AACAAGATCATTAATCTCGCAACGTGCGACTGGATCTACTTTATCGACGCGGATAATTATTATGATTCGAAGAACAAGGGTAAAGCCATGCGCATCGCTA
AGGTTATGGATTTCTTGAAAATCGAAGGCGTTGTGAGCCCAACGGTCATTGAGCATGACAATAGCATGAGCCGTGATACCCGTAAGATGTTTCGTCTGAA
AGATAACATTCTGTTTAGCGGTAAAGTTCATGAAGAACCGGTGTATGCCAATGGTGAGATCCCCCGGAACATCATAGTAGACATCAACGTGTTTCACGAC
GGCTATAACCCAAAGATTATCAACATGATGGAAAAGAACGAGCGCAATATCACCCTGACTAAAGAGATGATGAAGATCGAACCGAACAATCCGAAATGGC
TGTACTTCTATAGCCGCGAACTCTATCAGACGCAACGTGACATTGCCCTTGTGCAAAGTGTACTGTTCAAGGCACTGGAACTGTATGAAAACAGTTCATA
TACGCGTTATTATGTTGACACCATTGCCTTACTGTGCCGAGTGCTGTTCGAATCTAAAAACTACCAGAAACTTACGGAATGTCTGAACATCCTGGAGAAC
AATACGCTTAACTGTTCCGATATCGATTACTATAATTCAGCGCTGCTGTTCTACAACCTGTTACTGCGCATCAAGAAAATTAGCTCCACCCTGAAGGAGA
ACATTGATATGTACGAACGTGACTATCATAGCTTTATCAACCCCTCGCATGATCACATTAAGATTCTGATATTAAATATGCTCCTGCTGCTCGGCGATTA
CCAGGATGCCTTTAAGGTTTACAAGGAGATCAAGTCCATTGAGATTAAAGATGAGTTTCTGGTGAACGTGAACAAATTCAAAGACAATCTTCTGAGCTTC
ATTGACTCCATTAACAAAATTTAATAA

pEG7143 SgbL

AGAAAAACGAGGAGGTAATAGATGACAAGCCATGCAACCGAGGTTGAATGGGAGGACCTTCTGCGCCAAGCATTACACGCAACTGGTACAGGTGCTCGTT
GGGCTGTAGAGGCGGACGAGATGTGGTGCCGTGTCGCCCCGGTGCCTGGAACTCGCCGCGAGCAAGGATGGAAGCTTCATGTAAGCGCGACGACCGCGAG
TGCGCCCGAAGTCTTAACTCGTGCATTAGGCGTACTTCTGCGTGAAAAGTCCGGGTTCAAATTTGCCCGCTCACTTGAACAAGTCTCGGCCTTGAATAGT
CGTGCTACGCCCCGTGGTAGTTCGGGTAAATTTATCACAGTATACCCCCGCTCAGACGCCGAAGCCGTCGCACTGGCTCGCGACCTGCATGCGGCAACGG
CCGGCTTGGCTGGGCCCCGTATTCTTTCCGATCAACCATACGCCGCGCACAGCCTGGTGCATTATCGTTATGGGGCTTTCGTGGGACGTCGTCGCCTTTC
AGATGACGGGCTTTTAGTTTGGTTTATTGAGGACCCAGATGGCAATCCCGTGGAGGATAAACGCACCGGACGTTATGCGCCGCCTCCCTGGGCTGTATGT
CCGTTTCCTGCGAGCGTCCCCGTTGCGCCCCATGACGGCGAAGCTACGAGTCGTCCTGTTGTCTTAGGTGGTCGCTTCGCGGTTCGTGAAGCCATCCGTC
AAACGAATAAAGGGGGCGTCTATCGCGGGTCGGACACACGCACTGGCACCGGCGTGGTTATCAAAGAGGCGCGCCCACATGTTGAAGGAGACGCCAGTGG
GGGCGATGTTCGTGACTGGCTTCGCGCAGAGGCGCGTACGCTTGAAAAATTAAAAGGTACCGGCTTGGCACCAGAAGCGGTGGCGTTGTTTGAGCACGCT
GGCCACTTGTTCTTAGCCCAAGACGAGGTCCCGGGGGTTACGTTACGCACCTGGGTAGCGGAACACTTCCGTGACGTTGGAGGAGAGCGCTATCGTGCCG
ACGCCCTGGCTCAGGTGGCTCGTTTAGTTGATTTAGTCGCGGCTGCTCATGCACGTGGCTTGGTCCTGCGCGATTTTACACCAGGGAACGTGATGGTCCG
TCCAGACGGCGAATTGCGCCTTATTGATTTAGAGCTGGCGGTTCTTGAGGATGAGGCCGCATTGCCTACCCACGTCGGTACCCCGGGGTTTTCGGCACCC
GAACGCCTTGCAGACGCTCCAGTGCGTCCTACTGCTGACTACTATTCTCTGGGAGCCACAGCTTGTTTTGTCTTGGCCGGTAAAGTCCCTAATTTACTTC
CTGAAGAACCCGTGGGTCGCCCATCGGAGGAGCGTCTTGCTGCCTGGTTGACTGCATGTACACGTCCGCTGCGCCTGCCAGATGGAGTCGTTGACATGAT
CTTGGGGTTAATGCGCGATGATCCTGCAGAGCGCTGGGACCCATCCCGCGCGCGTGAAGCACTGCGCAAAGCTGACCCGACAGCACGCCCCGGGGATGCT
GATCGCACTGCAGTACGTCGTACGGGTTCGTCGGCAGTGGCCGGGCCAGTTCCTGACTCACGTACAGCAGATGGTCGTACAGCGGACGGCCGTTCCGCGG
ATGAAGTTGTGGCAGGTCTTGTCGATCACTTAGTCGATAGTATGACCCCGGCAGATGATCGTCTGTGGCCGGTAAGCACTCTTACGGGAGAATCGGATCC
ATGTACAGTCCAGCAAGGCGCTGCTGGGGTGCTTGCGGTGTTGACCCGCTACTTCGAATTGACGGGCGATCCGCGCTTACCAGGCTTATTGTCGACAGCC
GGACGTTGGATCGCAGACCGCACGGATGTTCGTTCACCTCGTCCGGGATTACATTTCGGGGGACGCGGAACAGCCTGGGCCTTATACGACGCGGGGCGTG
CAGTCGACGATCGTCGCTTGGTGGAACATGCTCTGGACTTAGCATTAGCCCCGCCCCAAGCGACTCCTCATCACGATGTCACGCATGGGACTGCGGGCTC
AGGCTTAGCCGCCTTGCACCTGTGGCAGCGTACTGGAGATACTCGTTTCGCGGATTTAGCAGTAGAGGCAGCTGATCGCTTAACAGCTGCAGCTCGTCGC
GAGCCTTCGGGTGTTGGATGGGCAGTACCTGCAGAGGCCGACTCCCCAGAAGGAGGCAAGCGTTACCTGGGCTTCGCTCATGGCGCAGCTGGGATTGGGT
GCTTCTTATTGGCTGCGGCGGAACTTAGTCGTCAACCCGATCATCGTGCAACTGCTTTGGAAGTTGGCGAAGGCCTGGTTGCTGATGCTGTTCGCATCGG
AGAGGCGGCACAGTGGCCTGCGCAATCCGGGGACTTGCCGACAGCGCCTTACTGGTGCCATGGGGCGGCAGGTATCGGGACATTTCTTGTACGCTTATGG
CAGGCGACCGGGGACGATCGCTTCGGTGATCTGGCCCGCGGGAGTGCTCACGCTGTGGCCGAACGTGCTAGTCGCGCCCCATTGGCGCAATGTCACGGTT
TGGCTGGAAACGGAGATTTCTTGTTGGATTTGGCAGACGCGACAGGCGATCCTGTGCATCGCGACACCGCGGAAGAGTTAGCAGGGTTGATCTTGGCCGA
AGGAACCCGTCGTCAGGGACATGTCGTTTTCCCTAATGAGTATGGGGAAGTATCATCTTCATGGTCCGACGGTAGTGCGGGGATTCTTGCGTTCCTTCTG
CGTACGCGTCATACGGGCCCTCGCCATTGGATGGTAGAACAACGTGGGTAATAA

pEG7144 RaxST

AGAGCCTTCCACAAACTAAGGAGCACAATTATGGATTATCATTTCATCAGCGGACTGCCTCGTGCGGGGAGTTCATTACTGGCTGCGTTACTGCGTCAAA
ATCCGCAGCTGCATGCCGATGTTACATCTCCGGTGGCGCGCCTTTACGCGGCCATGCTGATGGGTATGAGTGAAGAACACCCGAGCAACGTGCAGATTGA
CGATGCCCAACGTGTCCGTCTGTTACGTGCAGTATTTGATGCGTATTATCAGAACCGTCAGGAACTGGGGACAGTGTTCGATACTAACCGCGCATGGTGC
TCTCGCCTCACGGGCCTGGCGCGTCTGTTTCCGCGTAGTCGCATGATCTGCTGTGTACGCGATGTGGGCTGGATTGTTGATTCTTTTGAACGCCTGGCGC
AGTCGCAGCCGTTACGCCTTTCGGCCCTGTTCGGTTACGACCCCGAGGATTCGGTTAGCATGCACGCTGACTTACTCACTGCGCCTCGCGGGGTAGTGGG
CTACGCCCTGGATGGTTTACGTCAAGCGTTTTATGGAGATCACGCGGATCGTCTGCTGTTGTTACGTTATGATACGCTGGCACAGCGTCCTGCACAAGCC
ATGGAACAGGTATATGCATTCCTGCAGCTCCCTGCCTTTGCACATGATTATGCCGGTGTTCAGGCCGAAGCGGAACGCTTTGATGCCGCCCTGCAAATGC
CTGGTTTGCACCGCGTGCGTCGTGGTGTTCACTATGTTCCGCGACGTTCGGTTTTACCGCCTGCCCTGTTTGACCAGCTGCAGGAACTTGCATTCTGGGA
AAGTGCACCCAGCCATGGAGCGCTGCTCGTGTAATAA

pEG7145 ComQ

AGAGGAACGAGAAATAAGGACACAGATATATGAAGGAAATCGTGAAACAGAATATCAGTAACAAAGACCTGTCGCAACTCCTGTGTTCCTTCATTGATTC
AAAGGAAACTTTCAGTTTTGCCGAGAGCGCTATACTGCATTATGTAGTATTCGGCGGTGAGAACCTGGACGTAGCTACCTGGCTGGGCGCCGGAATTGAA
ATTCTGATCCTGAGCAGCGATATCATGGACGACCTGGAGGACGAGGATAACCATCATGCGTTGTGGATGAAAATTAACCGCAGCGAGAGCTTGAATGCGG
CCCTGTCCTTATACACCGTCGGCTTAACGAGCATCTATTCCCTGAACACAAATCCGTTGATATTTAAGTATGTGCTGCGCTACGTCAATGAGGCCATGCA
GGGTCAGCATGATGATATAACCAATAAAAGCAAAACCGAAGATGAATCGCTTGAAGTGATTCGCCTTAAATGCGGCAGCCTGATCGCCCTGGCAAATGTC
GCGGGCGTGCTGTTAGCCACGGGCGAGTACAATGAAACAGTTGAACGTTACTCTTATTACAAAGGCATCGTGGCGCAAATTTCCGGCGACTATCACGTGC
TGCTGTCAGGAAACCGGAGCGATATCGAGAAAAACAAACAGACACTGATTTACCTGTATCTGAAACGCCTGTTTAACAACGCGAGCGAGGAATTGCTGTA
TCTGTTCTCCCATAAAGATTTGTACTATAAAGCCCTGCTCGACCGTGAAAAGTTTGAAGAAAAACTGATCCAGGCCGGGGTGACGCAGTACATCAGCGTT
CTGCTCGAAATATATAAGCAGAAGTGCTTCTCCACCATAGAACAGCTGAACTTAGATAAAGAAAAGAAAGAGCTGATCAAGGAGAGCCTGCTGTCATATA
AGAAAGGCGACACCCGTTGCAAGACCTAATAA

pEG7146	KgpF	AGAACGCAGACAATTTCATAGGAGGTCCCGATGATCAATTATGCTAATGCGCAGCTCCATAAGAGTAAAAACTTGATGTATATGAAAGCCCACGAAAACA<br>TCTTCGAAATCGAGGCGCTGTACCCGCTGGAATTGTTCGAGCGTTTTATGCAGTCCCAAACCGATTGCTCCATCGATTGTGCCTGTAAAATTGATGGTGA<br>CGAATTGTATCCCGCCCGTTTTAGTCTGGCCCTGTATAACAACCAGTATGCCGAAAAGCAAATTCGCGAAACCATCGACTTCTTCCATCAGGTAGAGGGT<br>CGGACCGAGGTGAAACTGAACTATCAGCAACTGCAGCACTTCCTGGGTGCTGACTTCGATTTTAGCAAAGTGATTCGAAACCTGGTGGGTGTGGATGCAC<br>GCCGCGAACTGGCTGATTCCCGGGTTAAACTGTATATTTGGATGAACGATTACCCAGAGAAAATGGCGACCGCCATGGCATGGTGCGATGATAAGAAGGA<br>ATTGTCGACGTTGATAGTAAATCAGGAGTTTCTGGTCGGGTTCGATTTTTATTTCGATGGTCGCACGGCAATAGAATTATACATTAGTCTGTCATCCGAA<br>GAATTTCAGCAGACACAAGTTTGGGAACGCCTCGCAAAGGTAGTGTGCGCCCCAGCGCTGCGCCTTGTTAATGATTGCCAGGCGATCCAGATTGGCGTGA<br>GCCGTGCCAATGATAGTAAGATCATGTATTACCATACCCTTAATCCGAACTCGTTTATCGACAATCTGGGCAATGAAATGGCAAGCAGAGTTCACGCGTA<br>TTACCGACATCAACCGGTTCGCTCTCTGGTAGTATGCATACCAGAACAGGAGTTGACCGCCCGGTCCATACAGCGCTTAAACATGTATTACTGTATGAAC<br>TAATAA
pEG7147	TgnB	AGAAATATTACAACGAGGTAAAGGCATGAAAACCATTCTGATTATTACCAATACCCTGGATCTGACCGTGGATTATATTATTAATCGCTATAATCATACC<br>GCTAAATTTTTCGTCTGAATACCGATCGTTTTTTGATTATGATATTAATATTACCAATAGCGGTACCAGCATTCGTAATCGTAAATCTAATCTGATTA<br>TTAATATTCAGGAAATTCATAGCCTGTATTATCGCAAAATTACCCTGCCGAATCTGGATGGCTATGAAAGTAAATATTGGACCCTGATGCAGCGCGAAAT<br>GATGAGTATTGTTGAAGGCATTGCAGAAACCGCTGGCAATTTTGCACTGACCCGTCCGTCTGTGCTGCGCAAAGCTGATAATAAAATTGTGCAGATGAAA<br>CTGGCAGAAGAAATTGGTTTTATTCTGCCGCAGAGTCTGATTACCAATTCAAATCAGGCGGCAGCCTCATTTTGCAATAAAAATAATACCAGCATTGTGA<br>AACCGCTGAGTACCGGCCGCATTCTGGGTAAAAATAAAATTGGCATTATTCAGACCAATCTGGTTGAAACCCATGAAAATATTCAGGGCCTGGAACTGTC<br>TCCGGCTTATTTTCAGGATTATATTCCGAAAGATACCGAAATTCGTCTGACCATTGTTGGTAATAAACTGTTTGGCGCCAATATTAAATCAACCAATCAG<br>GTTGATTGGCGCAAAAATGATGCACTGCTGGAATATAAACCGGCCAATATTCCGGATAAAATTGCCAAAATGTGTCTGGAAATGATGGAAAAACTGGAAA<br>TTAATTTTGCGGCGTTTGATTTTATTATTCGTAATGGTGATTATATTTTTCTGGAACTGAATGCCAATGGTCAGTGGCTGTGGCTGGAAGATATTCTGAA<br>ATTTGATATTTCAAATACCATTATTAATTATCTGCTGGGTGAACCGATTTAATAATAA
pEG7149	PapB	AGAACTAAGGAGGTTAGAGGATGGCAAACCTGATCCAGGACCGCGAGGACGAACTGATTCATTTCCATCCGTACAAACTGTTCGAGGTGGATTCAAAAAC<br>CTTCTTCTATAACGTAGTCACCAACGCGATTTTTGAAATTGATAGCCTGATAATCGACATTCTTCACTCAAAAGGTAAAAATGAGGAGCACGTTGTGAAA<br>GATTTGGCTGAACGCTATGAGCTGTCTCAGGTTCGCGAAGCGATCCAGAACATGAAAGAGGCATACATTATAGCAACCGATGCTAACATCTCCGACGTAG<br>AGAAGATGGGTATCTTAGATAACTCGCAGCGCGTTTTTAAACTGTCTAGCCTGACGCTCTTTATGGTGCAGGAATGCAACCTGCGGTGTACGTATTGTTA<br>CGGCGAAGAAGGAGAATACAACCAGAAAGGTAAAATGACGTCCGAAATCGCCCGGAGCGCAGTGGATTTTCTGATTCAACAGAGTGGTGAAATCGAACAG<br>TTGAACATCACATTCTTTGGAGGCGAACCGCTGCTCAACTTTCCATTAATACAAGAAACCGTGCAGTATGTGCACGAACAGAGCGAGATCCATAACAAGA<br>AATTTAGCTTTTCCATCACCACCAATGGCACGCTCATTACCCCCAAAATCAAAAACTTCTTCTATAAACACCACTTTGCAGTCCAGACTTCTATCGATGG<br>TGATGAAAAGACGCACAATTTCAATCGCTTCTTCAAAGGAGGCCAGGGCTCTTATGATCTGCTGTTAAAGCGGACGGAAGAAATGCGCAATGACCGTAAA<br>ATTGGTGCACGTGGAACCGTGACCCCTGCCGAGCTGGACCTCTCAAAATCATTTGACCACTTAGTTAAACTCGGCTTTCGCAAAATCTACTTATCACCCG<br>CTTTATATAGTCTCTCTGACGATCACTACGACACCCTGAGCAAAGAGATGGTCAAACTTGTTGAACAATTCCGTGAGCTGCTGGAGCGTGAAGATTACGT<br>CACCGCGAAGAAAATGTCTAATGTTCTGGGTATGTTATCGAAGATTCACTCCGGTGGCCCGCGCATTCATTTTTGCGGTGCCGGCACTAATGCTGCCGCT<br>GTCGATGTCCGCGGCAACCTTTTCCCGTGTCATCGTTTCGTGGGTGAAGATGAATGTTCAATCGGTAACCTGTTCGACGAGGACCCGCTGTCAAAACAGT<br>ACAACTTTATAGAGAATTCTACAGTACGCAACCGTACTACGTGTTCGAAATGCTGGGCGAAGAATCTGTGCGGCGGTGGTTGTCACCAAGAAAATTTCGC<br>CGAGAATGGTAATGTGAACCAGCCAGTGGGCAAATTATGCAAAGTGACCAAAAACTTCATCAACGCGACCATCAATCTGTACTTGCAACTTACTCAAGAA<br>CAACGCAGCATTCTGTTCGGCTAATAA
pEG7152	PcpXY	AGACACAGGGAGGTCTTTATATGACATACCGTCGCACCTCCTATGCGGTATGGGAGATCACGCTGAAATGCAATCTGGCATGTTCGCACTGTGGAAGTCG<br>TGCCGGGCACACGCGAGCAAAAGAACTGTCCACACAGGAAGCGCTGGATCTGGTCCGTCAGATGGCTGATGTCGGCATTATCGAAGTTACTCTGATTGGG<br>GGTGAAGCGTTCCTGCGTCCAGACTGGCTGCAGATTGCCGAGGCGATAACGAAAGCCGGGATGCTGTGCAGCATGACTACGGGCGGTTATGGCATATCGC<br>TGGAAACCGCCCGCAAAATGAAAGCGGCAGGAATCGCGAGCGTGAGCGTTAGCATCGATGGCTTGGAGGAAACCCATGATCGCTTACGCGGTCGCAAAGG<br>CTCTTGGCAGGCTGCGTTTAAAACAATGAGCCATTTGAGAGAAGTGGGCATCTTCTTTGGCTGTAACACCCAGATTAACCGTCTGTCGGCCCCTGAATTT<br>CCGCTGATATATGAACGCATCCGTGACGCCGGGGCACGTGCCTGGCAGATCCAGCTTACGGTGCCGATGGGCCGCGCTGCCGATAACGCAAATATCCTTC<br>TGCAACCGTACGAACTGCTTGATCTGTATCCGATGATTGCTCGAGTGGCCCGCCGGGCCCGTCAAGAGGGCGTGCAAATCCAGCCAGGTAATAATATTGG<br>GTATTACGGCCCTTACGAACGTCTTTTACGTGGCCGGGGGAGCGATAGTGAGTGGGCATTTTGGCAGGGCTGTGCCGCGGGCTTAAGTACCCTGGGTATT<br>GAAGCGGATGGTGCTATAAAAGGTTGTCCCTCACTGCCAACGAGCGCGTATACCGGCGGTAACATTCGCGAACATAGTCTGCGAGAAATAGTGGAAGAAT<br>CGGAACAGCTGCGTTTTAACCTCGGTGCAGGGACGAGCCAAGGGACCGCCCACTTGTGGGGCTTTTGCCAGACGTGTGAATTTAGTGAATTGTGCAGAGG<br>TGGTTGTACGTGGACAGCTCACGTGTTCTTTAACCGCCGTGGGAATAACCCGTATTGTCATCATCGGGCGCTTTTCCAAGCGGAGCAGGGTATCAGAGAA<br>CGTGTCGTGCCAAAGGTCGAAGCTCAGGGCCTGCCGTTTGACAACGGTGAATTTGAACTTATCGAAGAACCTATTGACGCGCCTCTGCCCGAAAATGATC<br>CACTGCACTTTACCAGCGACTTAGTGCAGTGGTCAGCGAGTTGGCAGGAAGAATCGGAATCTATAGGCGCAGTGGTAGACTAATAACACAAGGGGGTAGT<br>AGTATGGTGGAAAACATTGATAATGAACGTGAGAAAAGTGCGAACGAAATTGAACCGGAAAGCCTGCTTCTGCCGCGCCAGGCTTGGCAGTCGCAGATCG<br>CCTATCTTAAAGCGATTCTGAAAGCCAAACAGGCGCTTGACCGGATCGAAAAACGTTATCTGCGGTAATAA
pEG7160	LynD	CTAAATTCCCCCGAGGTCAATAATGCAATCTACACCATTACTGCAAATACAACCACATTTCCATGTAGAGGTCATTGAACCAAAGCAAGTCTACTTGTTG<br>GGTGAACAAGCTAATCATGCATTGACAGGCCAATTATACTGCCAAATTTTGCCATTGTTAAACGGACAATACACATTGGAACAAATCGTTGAAAAACTAG<br>ACGGAGAAGTACCACCTGAATACATTGATTATGTGCTGGAGAGACTAGCTGAGAAGGGCTATCTGACTGAAGCAGCACCTGAATTATCTAGTGAAGTGGC<br>CGCTTTCTGGTCTGAGCTGGGGATTGCACCTCCTGTCGCGGCCGAAGCATTACGTCAACCTGTGACTTTAACACCTGTTGGAAACATCAGCGAAGTAACA<br>GTAGCAGCCTTAACCACAGCCCTACGTGATATCGGTATTTCCGTTCAAACACCTACAGAAGCTGGATCGCCAACTGCATTGAACGTTGTACTTACCGATG<br>ATTATCTCCAACCAGAACTCGCTAAGATCAATAAGCAAGCCTTAGAAAGTCAACAAACTTGGCTACTTGTCAAACCAGTTGGCTCCGTGTTATGGTTGGG<br>TCCGGTATTCGTGCCAGGAAAAACAGGTTGCTGGGATTGTTTGGCTCACAGATTAAGGGGGAATAGAGAGGTAGAGGCCTCTGTATTGAGACAAAAACAA<br>GCTCAACAACAACGTAATGGACAAAGCGGGTCTGTAATAGGATGCCTTCCCACGGCTAGAGCGACACTGCCCTCAACACTCCAAACTGGGCTGCAGTTCG<br>CTGCTACCGAAATTGCTAAATGGATAGTTAAGTATCATGTTAATGCCACAGCGCCTGGCACCGTATTCTTCCCTACATTGGATGGTAAGATAATTACGCT<br>AAATCACTCCATACTGGATTTGAAGTCACATATTCTGATCAAGCGTTCTCAATGTCCCACCTGTGGTGACCCAAAAATCTTACAGCACCGTGGTTTCGAA<br>CCTTTAAAACTTGAGTCAAGGCCTAAACAGTTCACCTCAGACGGCGGACATCGTGGTACTACCCCTGAACAAACTGTCCAGAAATATCAACATTTAATCT<br>CGCCTGTTACCGGTGTAGTTACTGAATTGGTCAGGATAACTGATCCGGCCAATCCACTAGTTCACACATATAGAGCTGGTCATAGCTTCGGGAGCGCTAC<br>ATCGCTGAGAGGGCTGCGTAATACCTTAAAGCATAAGAGTTCAGGTAAGGGTAAGACTGATTCTCAAAGTAAAGCCTCGGGCCTGTGTGAGGCGGTAGAA<br>CGTTACTCAGGAATCTTTCAAGGTGACGAACCGAGAAAACGCGCCACATTGGCTGAATTGGGAGATTTGGCAATTCACCCTGAGCAATGCTTGTGTTTTT<br>CCGACGGTCAGTACGCTAATAGAGAAACTTTAAACGAACAGGCAACGGTGGCACATGATTGGATACCTCAACGTTTTGATGCATCACAAGCTATTGAATG<br>GACTCCAGTCTGGTCCCTAACTGAACAGACCCATAAATATTTGCCCACCGCATTGTGTTACTACCATTATCCTCTACCCCCAGAACACAGATTCGCACGT<br>GGAGATTCGAATGGTAATGCTGCCGGAAATACGTTGGAAGAGGCTATACTCCAAGGCTTCATGGAATTAGTCGAGAGAGATGGTGTGGCTTTATGGTGGT<br>ATAACAGGCTACGCAGACCCGCTGTAGACTTAGGCTCATTTAACGAGCCATACTTCGTTCAGTTGCAACAATTCTACAGAGAAAACGATAGAGATTTGTG<br>GGTTTTGGACTTGACAGCTGATTTAGGTATCCCGGCTTTCGCGGGCGTTTCTAATAGAAAAACTGGTAGTTCGGAGAGGTTGATATTAGGATTCGGTGCA<br>CACCTCGATCCTACTATTGCAATTCTGAGAGCAGTTACAGAAGTTAACCAGATTGGCCTTGAATTAGATAAAGTTCCAGACGAGAACCTTAAGAGCGACG<br>CAACAGATTGGCTAATTACTGAAAAATTAGCTGACCACCCTTATTTGTTACCAGATACAACTCAACCTCTAAAAACTGCTCAAGATTATCCTAAAAGGTG<br>GTCTGACGATATATACACGGACGTAATGACTTGCGTTAATATTGCTCAACAAGCAGGACTTGAAACTCTAGTTATTGATCAAACACGTCCGGACATTGGT<br>TTGAATGTTGTTAAGGTGACAGTCCCGGGGATGAGGCACTTTTGGTCAAGATTTGGAGAGGGGAGGCTTTATGACGTGCCCGTCAAATTAGGTTGGCTTG<br>ACGAACCTTTGACCGAAGCGCAAATGAACCCCACGCCGATGCCTTTTTAATAA
pEG7166	PapoK	TTCAATCGTTAAGGAGGTACATAAATGCACGATCGTAGCGCGAATGTTAGCTGGACCAAATACATCGCGTTTGGTCTGCGCATTGCCAGCGAACTCAACT<br>TACCGGAACTGATATTGGCGGCTCCCGAAGCCGTTGAGGATGTTGTCATACGCCAGGCAGATCTCACGGCCTGGTCTGGCCAACTTGAACAGGCAAATTT<br>TGTCATGTTGGACGAACGTTTCATGTTTCAGATCCCGGGGACCGCCATTTATGCGGTACGCGAAGGCAAAGAGATTGAAGTGAGCATCTTCTCTGGGGCC<br>GACCCGGACACCGTGCGCCTTTTCGTGCTGGGGACGTGCATGGGCGTGCTCTTGATGCAGCGCCGCATTCTGCCTATCCACGGCTCCGCCGTCGTTATCG<br>GTGGCCGCGCGTATGCCTTTGTTGGTGAATCAGGCACAGGTAAATCGACCTTAGCTGCAGCATTTCGGCAGGCCGGTTACCAAATGGTTAGCGATGATGT<br>CATTGCCGTCAAAGCGACCGCATCTAGCGCTATTGTTTACCCTGCGTATCCACAGCAAAAACTGGGTTTAGATTCGCTGTTGCAGCTTGAAGCGCTCCGT<br>GAGAATAAGCACGCCCGCAAGCGTAACAACATCCGTTCTCTGACGGATGGCAATAGTGTGATGCCGCAGTACAGCGATCTGCGCATGCTGGCGGGGGAAC<br>TGAATAAATATGCAGTTCCAGCCGTCGATGAATTCTTTAATGACCCGCTGCCGTTGGGCGGTGTTTTCGAACTGGTAGCAGACAGTCCGATTCGAGCATT<br>AATGCGCGAAGGCGAACTCGTCGCTGTGACCGAGCAACCGCTGAACGTTCTGGAATGTTTACATACTCTTCTGCAACACACGTACCGTCGGGTAATCATC<br>CCTCGAATGGGACTGAGCGAGTGGAGCTTCGATACTGCGGCCCGAATGGCACGCAAGGTCGAGGGCTGGCGACTCCTCCGTGATAGCTCCGTGTTCACGG<br>CTAGTGAAGTCGTCCAGCGCGTCCTCGACATCATCCGTAAGGAGGAAAAGAGCTACGGATCACACTAATAA
pEG7169	EpiD	ACTGAACTATAAGGTAGGTATATTATGCACGGTAAACTGCTGATCTGCGCAACTGCTTCGATCAACGTCATCAATATCAACCATTATATTGTGGAGCTGA<br>AACAGCACTTCGATGAGGTGAATATCCTGTTTTCACCTTCCTCGAAGAACTTTATCAACACCGATGTCCTGAAGCTGTTTTGCGATAATCTGTATGACGA<br>GATCAAAGATCCGCTGCTGAACCACATCAACATAGTGGAGAACCACGAGTATATCTTGGTGCTGCCTGCCAGTGCCAATACGATCAACAAAATCGCGAAC<br>GGTATATGCGATAACCTCTTGACGACCGTATGCTTAACCGGGTACCAGAAACTGTTTATCTTTCCGAATATGAACATCCGCATGTGGGGAAATCCGTTCT<br>TACAGAAAAATATTGACCTGCTTAAAAGCAACGACGTGAAGGTGTATTCCCCCGACATGAACAAATCTTTTGAGATAAGCTCAGGCCGCTACAAAAATAA<br>CATCACGATGCCGAATATCGAAAACGTGCTGAATTTTGTCCTGAACAATGAGAAACGCCCGCTGGATTAATAA

pEG7171	BamB	GCCCCGTCAGACACCTTCTAAGGAGGACATATATGGAAGGGTTGTATCAGCTGAAAGTGCATAGTCGTATACACAAACTGCAAAATAATATCGCAATAGG<br>TAGCATGCCGCCTCACGCGCTGATCATCGAGGATGCCCCCGAATATTTGTCAAACGTTCTGCGCTTCTTTAGTAGCAAAAAGACTATAAAAGAAGCTGAA<br>GTGTACCTGTCGGATAATACGAATCTGAGCTCCAATGAGATCAACCTGTTGTTAGGTGATCTGATTGAGAACGAGATTATCGTAAAGCAAAACTACGACT<br>CGAATAATCGGTACAGTCGACACAGTCTGTATTACGAGATGATTGATGCCAACGCTGAAAACGCGCAGAAAATTCTGGCAGAGAAAACAGTGGGCCTCGT<br>TGGGATGGGCGGGATTGGTTCCAATGTAGCCATGAATCTCGCAGCCGCCGGTGTTGGCAAACTGATCTTTAGTGATGGCGATACCATAGAACTGTCTAAT<br>TTAACGCGACAGTATCTTTACAAAGAGGATCAGGTGGGCTTGAGCAAAGTAGAGAGCGCCAAAGAACAACTGCAATTACTGAATAGCGAAGTCGAGCTTA<br>TCCCGGTTTGCGAAAGTATCTCTGGTGAGGAACTGTTCGACAACCATTTCTCCGAATGCGATTTCGTCGTACTGTCCGCCGACTCTCCGTTCTTTGTTCA<br>CGAATGGATTAACAATGCCGCGTTGAAATATGGCTTCTCCTACTCTAACGCAGGATATATCGAAACCTATGGCGCGATCGGTCCACTGGTGATACCTGGG<br>GAAACTGCCTGCTACGAATGCTATAAAGACAAGGGCGATCTTTACTTGTACTCCGACAACAAGGAAGAATTTTCTGTGAACCTGAATGAATCATTCCAAG<br>CACCGAGCTATGGACCGCTTAATGCGATGGTTAGTTCCATTCAGGCGAATGAAGTGATACGCCACCTCCTCGGACTTAAAACCAAAACGTCCGGCAAACG<br>GCTGCTGATCAACAGTGAAATCTACAAAATCCACGAAGAGAACTTCGAGAAGAAGAACAACTGCCTGTGCTCGGATATTAAGGGCGAGAAGCTGTCGAAG<br>AACACCCTTAACTCCGATAAAGAGCTGCACGAAGTGTATATCGAAGAACGCGAATCGGATTCTTTCAACTCCATTCTCTTGGATAAAACCATGAGCAAGC<br>TGGTAAAAATTAACAAAGAGGAGACAAAAATCCTCGACATTGGTTGCGCTACCGGCGAACAGGCTCTGTATTTCGCGAATAAAGGTGCTAAGGTGACCGC<br>TGTCGACATTTCAGACGATATGTTGAAGGTGCTGGACAAGAAAGCAAGCAACATTAACGCGGGGAGTATCAAAACCATGCGTGGTAATATCGAATCCATC<br>GAGGTGAATGACACTTTTAATTACATCGTCTGTAACAACATCCTTGATTACCTGCCGGAGATCGACCGCACGCTGAGAAAACTTAACATGTTTTTGAAAA<br>ATGACGGGACGCTGATTGTGACGATTCCCCACCCCGTGAAGGATGGTGGAGGGTGGCGGAAAGATTATTATAACGGCAAATGGAACTACGAAGAGTTTAT<br>CCTGAAGGATTACTTCAACGAGGGTCTGATCGAAAAGAGCCGCGAGGACAAAAATGGGGAAACGGTGATCAAAAGCATTAAAACGTACCACAGAACCACC<br>GAAACCTATTTCAATAGCTTTACTGACGCTGGCTTCAAGGTAGTATCTCTGCTGGAACCGCAACCGCTTTCAACTGTTTCAGAGACTCATCCAATTCTGT<br>TCGAAAAGTGTTCGCGCATTCCGTACTTTCAAGTTTTTGTGCTCAAGAAAGAGGATCGCCACGCCATTTAATAA

a. In each backbone sequence (labeled “Backbone Sequence”), the relevant part (peptide or RBS+enzyme) has GFP as a placeholder (RBS+GFP for enzyme plasmid) and is highlighted grey. Replace this region with the insert DNA listed for each plasmid (below the relevant backbone) to get each plasmids sequence.
b. Text colors correspond to promoters (orange), ribozyme insulators (blue), RBSs (green), protein coding sequences (red), terminators (purple), and plasmid backbone and spacers (black).
