# Supplementary material for: Functional expression of diverse post-translational peptide-modifying enzymes in Escherichia coli under uniform expression and purification conditions
Source: PLoS One. 2022 Sep 19;17(9):e0266488. doi: 10.1371/journal.pone.0266488 (PMC9484694; doi:10.1371/journal.pone.0266488)
Supplement: S1 File — References from Supporting information figures/tables/notes. (PDF) [file pone.0266488.s020.pdf]

S1 File. References

1. Lee H, Park Y, Kim S. Enzymatic Cross-Linking of Side Chains Generates a Modified Peptide with Four Hairpin-like Bicyclic Repeats. Biochemistry. 2017;56(37):4927-30. doi: 10.1021/acs.biochem.7b00808.
2. Gavrish E, Sit S, Clarissa, Cao S, Kandror O, Spoering A, Peoples A, et al. Lassomycin, a Ribosomally Synthesized Cyclic Peptide, Kills Mycobacterium tuberculosis by Targeting the ATP-Dependent Protease ClpC1P1P2. Chemistry & Biology. 2014;21(4):509-18. doi: 10.1016/j.chembiol.2014.01.014.
3. Knappe TA, Linne U, Robbel L, Marahiel MA. Insights into the Biosynthesis and Stability of the Lasso Peptide Capistruin. Chemistry & Biology. 2009;16(12):1290-8. doi: 10.1016/j.chembiol.2009.11.009.
4. Zong C, Cheung-Lee WL, Elashal HE, Raj M, Link AJ. Albusnodin: an acetylated lasso peptide fromStreptomyces albus. Chemical Communications. 2018;54(11):1339-42. doi: 10.1039/c7cc08620b.
5. Zimmermann M, Hegemann D, Julian, Xie X, Marahiel A, Mohamed. The Astexin-1 Lasso Peptides: Biosynthesis, Stability, and Structural Studies. Chemistry & Biology. 2013;20(4):558-69. doi: 10.1016/j.chembiol.2013.03.013.
6. Zimmermann M, Hegemann JD, Xie X, Marahiel MA. Characterization of caulonodin lasso peptides revealed unprecedented N-terminal residues and a precursor motif essential for peptide maturation. Chem Sci. 2014;5(10):4032-43. doi: 10.1039/c4sc01428f.
7. Hegemann JD, Zimmermann M, Xie X, Marahiel MA. Caulosegnins I–III: A Highly Diverse Group of Lasso Peptides Derived from a Single Biosynthetic Gene Cluster. Journal of the American Chemical Society. 2013;135(1):210-22. doi: 10.1021/ja308173b.
8. Zhu S, Hegemann JD, Fage CD, Zimmermann M, Xie X, Linne U, et al. Insights into the Unique Phosphorylation of the Lasso Peptide Paeninodin. J Biol Chem. 2016;291(26):13662-78. Epub 2016/05/05. doi: 10.1074/jbc.M116.722108. PubMed PMID: 27151214; PubMed Central PMCID: PMCPMC4919450.
9. Zhu S, Fage CD, Hegemann JD, Yan D, Marahiel MA. Dual substrate-controlled kinase activity leads to polyphosphorylated lasso peptides. FEBS Letters. 2016;590(19):3323-34. doi: 10.1002/1873-3468.12386.
10. Su Y, Han M, Meng X, Feng Y, Luo S, Yu C, et al. Discovery and characterization of a novel C-terminal peptide carboxyl methyltransferase in a lassomycin-like lasso peptide biosynthetic pathway. Applied Microbiology and Biotechnology. 2019;103(6):2649-64. doi: 10.1007/s00253-019-09645-x.
11. Ren H, Biswas S, Ho S, Van Der Donk WA, Zhao H. Rapid Discovery of Glycocins through Pathway Refactoring in Escherichia coli. ACS Chemical Biology. 2018;13(10):2966-72. doi: 10.1021/acschembio.8b00599.
12. Kaunietis A, Buivydas A, Čitavičius DJ, Kuipers OP. Heterologous biosynthesis and characterization of a glycocin from a thermophilic bacterium. Nature Communications. 2019;10(1). doi: 10.1038/s41467-019-09065-5.
13. Serebryakova M, Tsibulskaya D, Mokina O, Kulikovsky A, Nautiyal M, Van Aerschot A, et al. A Trojan-Horse Peptide-Carboxymethyl-Cytidine Antibiotic from Bacillus amyloliquefaciens. Journal of the American Chemical Society. 2016;138(48):15690-8. doi: 10.1021/jacs.6b09853.

14. Ansaldi M, Marolt D, Stebe T, Mandic-Mulec I, Dubnau D. Specific activation of the Bacillus quorum-sensing systems by isoprenylated pheromone variants. Molecular Microbiology. 2002;44(6):1561-73. doi: 10.1046/j.1365-2958.2002.02977.x.
15. Ghodge SV, Biernat KA, Bassett SJ, Redinbo MR, Bowers AA. Post-translational Claisen Condensation and Decarboxylation en Route to the Bicyclic Core of Pantocin A. Journal of the American Chemical Society. 2016;138(17):5487-90. doi: 10.1021/jacs.5b13529.
16. Jin M, Liu L, Wright SAI, Beer SV, Clardy J. Structural and Functional Analysis of Pantocin A: An Antibiotic from Pantoea agglomerans Discovered by Heterologous Expression of Cloned Genes. Angewandte Chemie International Edition. 2003;42(25):2898-901. doi: 10.1002/anie.200351053.
17. Luu DD, Joe A, Chen Y, Parys K, Bahar O, Pruitt R, et al. Biosynthesis and secretion of the microbial sulfated peptide RaxX and binding to the rice XA21 immune receptor. Proceedings of the National Academy of Sciences. 2019;116(17):8525-34. doi: 10.1073/pnas.1818275116.
18. Shuguo H, Wei Z, Chao Z, Daoji W. One-Step Expression and Tyrosine O-Sulfonation of Ax21 in Escherichia coli. Applied Biochemistry and Biotechnology. 2012;166(5):1368-79. doi: 10.1007/s12010-011-9525-3.
19. Morinaka BI, Lakis E, Verest M, Helf MJ, Scalvenzi T, Vagstad AL, et al. Natural noncanonical protein splicing yields products with diverse β-amino acid residues. Science. 2018;359(6377):779-82. doi: 10.1126/science.aao0157.
20. Lohans CT, Li JL, Vederas JC. Structure and Biosynthesis of Carnolysin, a Homologue of Enterococcal Cytolysin with d-Amino Acids. Journal of the American Chemical Society. 2014;136(38):13150-3. doi: 10.1021/ja5070813.
21. Hegemann JD, Van Der Donk WA. Investigation of Substrate Recognition and Biosynthesis in Class IV Lanthipeptide Systems. Journal of the American Chemical Society. 2018;140(17):5743-54. doi: 10.1021/jacs.8b01323.
22. Huo L, Van Der Donk WA. Discovery and Characterization of Bicereucin, an Unusual d-Amino Acid-Containing Mixed Two-Component Lantibiotic. Journal of the American Chemical Society. 2016;138(16):5254-7. doi: 10.1021/jacs.6b02513.
23. Mu D, Montalbán-López M, Deng J, Kuipers OP. Lantibiotic Reductase LtnJ Substrate Selectivity Assessed with a Collection of Nisin Derivatives as Substrates. Applied and Environmental Microbiology. 2015;81(11):3679-87. doi: 10.1128/aem.00475-15.
24. Tang W, Van Der Donk WA. Structural Characterization of Four Prochlorosins: A Novel Class of Lantipeptides Produced by Planktonic Marine Cyanobacteria. Biochemistry. 2012;51(21):4271-9. doi: 10.1021/bi300255s.
25. Li Y-M, Milne JC, Madison LL, Kolter R, Walsh CT. From Peptide Precursors to Oxazole and Thiazole-Containing Peptide Antibiotics: Microcin B17 Synthase. Science. 1996;274(5290):1188-93. doi: 10.1126/science.274.5290.1188.
26. Foulston LC, Bibb MJ. Microbisporicin gene cluster reveals unusual features of lantibiotic biosynthesis in actinomycetes. Proceedings of the National Academy of Sciences. 2010;107(30):13461-6. doi: 10.1073/pnas.1008285107.
27. ÖKesli AE, Cooper LE, Fogle EJ, Van Der Donk WA. Nine Post-translational Modifications during the Biosynthesis of Cinnamycin. Journal of the American Chemical Society. 2011;133(34):13753-60. doi: 10.1021/ja205783f.

28. Caetano T, Barbosa J, Möesker E, Süssmuth RD, Mendo S. Bioengineering of lanthipeptides in Escherichia coli: assessing the specificity of lichenicidin and haloduracin biosynthetic machinery. Research in Microbiology. 2014;165(7):600-4. doi: 10.1016/j.resmic.2014.07.006.
29. Mcclerren AL, Cooper LE, Quan C, Thomas PM, Kelleher NL, Van Der Donk WA. Discovery and in vitro biosynthesis of haloduracin, a two-component lantibiotic. Proceedings of the National Academy of Sciences. 2006;103(46):17243-8. doi: 10.1073/pnas.0606088103.
30. Kupke T, Kempter C, Jung G, Götz F. Oxidative Decarboxylation of Peptides Catalyzed by Flavoprotein EpiD. Journal of Biological Chemistry. 1995;270(19):11282-9. doi: 10.1074/jbc.270.19.11282.
31. Schnell N, Engelke G, Augustin J, Rosenstein R, Ungermann V, Gotz F, et al. Analysis of genes involved in the biosynthesis of lantibiotic epidermin. European Journal of Biochemistry. 1992;204(1):57-68. doi: 10.1111/j.1432-1033.1992.tb16605.x.
32. Zhang Y, Li K, Yang G, Mcbride JL, Bruner SD, Ding Y. A distributive peptide cyclase processes multiple microviridin core peptides within a single polypeptide substrate. Nature Communications. 2018;9(1). doi: 10.1038/s41467-018-04154-3.
33. Weiz R, Annika, Ishida K, Makower K, Ziemert N, Hertweck C, Dittmann E. Leader Peptide and a Membrane Protein Scaffold Guide the Biosynthesis of the Tricyclic Peptide Microviridin. Chemistry & Biology. 2011;18(11):1413-21. doi: 10.1016/j.chembiol.2011.09.011.
34. Roh H, Han Y, Lee H, Kim S. A Topologically Distinct Modified Peptide with Multiple Bicyclic Core Motifs Expands the Diversity of Microviridin-Like Peptides. ChemBioChem. 2019;20(8):1051-9. doi: 10.1002/cbic.201800678.
35. Donia MS, Ravel J, Schmidt EW. A global assembly line for cyanobactins. Nature Chemical Biology. 2008;4(6):341-3. doi: 10.1038/nchembio.84.
36. Sardar D, Pierce E, Mcintosh JA, Schmidt EW. Recognition Sequences and Substrate Evolution in Cyanobactin Biosynthesis. ACS Synthetic Biology. 2015;4(2):167-76. doi: 10.1021/sb500019b.
37. Parajuli A, Kwak DH, Dalponte L, Leikoski N, Galica T, Umeobika U, et al. A Unique Tryptophan C-Prenyltransferase from the Kawaguchipeptin Biosynthetic Pathway. Angewandte Chemie International Edition. 2016;55(11):3596-9. doi: 10.1002/anie.201509920.
38. Tocchetti A, Maffioli S, Iorio M, Alt S, Mazzei E, Brunati C, et al. Capturing Linear Intermediates and C-Terminal Variants during Maturation of the Thiopeptide GE2270. Chemistry & Biology. 2013;20(8):1067-77. doi: 10.1016/j.chembiol.2013.07.005.
39. Himes PM, Allen SE, Hwang S, Bowers AA. Production of Sactipeptides in Escherichia coli: Probing the Substrate Promiscuity of Subtilosin A Biosynthesis. ACS Chemical Biology. 2016;11(6):1737-44. doi: 10.1021/acschembio.6b00042.
40. Hudson GA, Burkhart BJ, Dicaprio AJ, Schwalen CJ, Kille B, Pogorelov TV, et al. Bioinformatic Mapping of Radical S-Adenosylmethionine-Dependent Ribosomally Synthesized and Post-Translationally Modified Peptides Identifies New Cα, Cβ, and Cγ-Linked Thioether-Containing Peptides. Journal of the American Chemical Society. 2019. doi: 10.1021/jacs.9b01519.
41. Meyer AJ, Segall-Shapiro TH, Glassey E, Zhang J, Voigt CA. Escherichia coli "Marionette" strains with 12 highly optimized small-molecule sensors. Nature Chemical Biology. 2019;15(2):196-204. doi: 10.1038/s41589-018-0168-3.

42. Chen Y-J, Liu P, Nielsen AAK, Brophy JAN, Clancy K, Peterson T, et al. Characterization of 582 natural and synthetic terminators and quantification of their design constraints. Nature Methods. 2013;10(7):659-64. doi: 10.1038/nmeth.2515.

43. Gruber AR, Lorenz R, Bernhart SH, Neubock R, Hofacker IL. The Vienna RNA Websuite. Nucleic Acids Research. 2008;36(Web Server):W70-W4. doi: 10.1093/nar/gkn188.

44. Stanton BC, Nielsen AAK, Tamsir A, Clancy K, Peterson T, Voigt CA. Genomic mining of prokaryotic repressors for orthogonal logic gates. Nature Chemical Biology. 2014;10(2):99-105. doi: 10.1038/nchembio.1411.

45. Lou C, Stanton B, Chen Y-J, Munsky B, Voigt CA. Ribozyme-based insulator parts buffer synthetic circuits from genetic context. Nature Biotechnology. 2012;30(11):1137-42. doi: 10.1038/nbt.2401.

46. Rocco CJ, Dennison KL, Klenchin VA, Rayment I, Escalante-Semerena JC. Construction and use of new cloning vectors for the rapid isolation of recombinant proteins from Escherichia coli. Plasmid. 2008;59(3):231-7. doi: 10.1016/j.plasmid.2008.01.001.

47. Noike M, Matsui T, Ooya K, Sasaki I, Ohtaki S, Hamano Y, et al. A peptide ligase and the ribosome cooperate to synthesize the peptide pheganomycin. Nature Chemical Biology. 2015;11(1):71-6. doi: 10.1038/nchembio.1697.

48. Schramma KR, Bushin LB, Seyedsayamdost MR. Structure and biosynthesis of a macrocyclic peptide containing an unprecedented lysine-to-tryptophan crosslink. Nature Chemistry. 2015;7(5):431-7. doi: 10.1038/nchem.2237.

49. Hudson GA, Zhang Z, Tietz JI, Mitchell DA, Van Der Donk WA. In Vitro Biosynthesis of the Core Scaffold of the Thiopeptide Thiomuracin. Journal of the American Chemical Society. 2015;137(51):16012-5. doi: 10.1021/jacs.5b10194.

50. Segall-Shapiro TH, Sontag ED, Voigt CA. Engineered promoters enable constant gene expression at any copy number in bacteria. Nature Biotechnology. 2018;36(4):352-8. doi: 10.1038/nbt.4111.
